# Supplementary material for: Enhancing Temporomandibular Disorders Education for Initial Care Clinicians Through Interprofessional Education
Source: MedEdPORTAL. 2024 Nov 19;20:11467. doi: 10.15766/mep_2374-8265.11467 (PMC11575917; doi:10.15766/mep_2374-8265.11467)
Supplement: Supplementary file 1 — Facilitator Guide.docxLearner Guide and Clinical Tools.pdfModule 1 - TMD Pathophysiology.pptxModule 2 - TMD Assessment.pptxModule 3 - TMD Diagnosis.pptxModule 4 - TMD Management.pptxSample Patient Education Tools.pptx [file mep_2374-8265.11467-s001.zip › _Educational Summary Report_11467.pdf]

MedEdPORTAL® | The AAMC Journal of Teaching and Learning Resources

Original Publication

Open Access

Enhancing Temporomandibular Disorders Education for Initial Care Clinicians Through Interprofessional Education

James Hawkins, DDS, MS, MEd-HPE*, Ronald Cervero, PhD, Steven J. Durning, MD, PhD

*Corresponding author: james.m.hawkins77.mil@health.mil

Abstract

Introduction: Temporomandibular disorders (TMDs) are common musculoskeletal pain conditions that can significantly impact daily activities such as eating, talking, breathing, intimacy, and expressing emotion. TMDs are often complex and multifactorial, and many patients experience overlapping pain conditions, sleep difficulties, and mental health challenges. The National Academies of Sciences, Engineering and Medicine (NASEM) has called for improved TMD education and training for health professionals, as current training opportunities are limited. **Methods:** To prepare health professionals to care for patients who have a TMD, we designed a 5-hour, interactive, module-based curriculum aligned with the 2020 NASEM recommendations. The four-part module set addresses TMD physiology and pathophysiology, assessment, diagnosis, and management. Instructional methods are founded on the Cognitive Theory of Multimedia Learning and include engaging videos, clinical evaluation and education tools, and interactive digital simulation scenarios. **Results:** Thirty learners from diverse health professional backgrounds (medicine, dentistry, physician assistant, nursing, physical therapy) participated. Multiple-choice question assessments and pre/post retrospective survey scores demonstrated enhanced knowledge ($M = 2.9$ vs. $M = 4.2$, $p < .001$) and perceived competence ($M = 1.9$ vs. $M = 3.4$, $p < .001$), respectively. Encouragingly, all participants indicated applicability to their clinical practice. **Discussion:** Our modules offer educators and clinicians a valuable resource to improve TMD knowledge and facilitate best practices. Supplementary resources included in the curriculum are conducive to clinical implementation, fostering improved clinician assessment and patient education.

Keywords
Temporomandibular Disorders, Pain Medicine, Interprofessional Education, Interdisciplinary Medicine, Primary Care, Military & Veterans' Health, Case-Based Learning, Multimedia Learning

Educational Objectives

By the end of this activity, learners will be able to:

1. Examine the prevalence, impact, and risk factors associated with temporomandibular disorders (TMDs), including common comorbidities.
2. Discuss the neuroanatomical and physiological factors related to temporomandibular joints and masticatory muscle function, as well as the implications for TMD.
3. Demonstrate proficiency in obtaining a comprehensive TMD history, performing a thorough TMD examination, and ordering relevant diagnostic tests and images.
4. Differentiate between arthrogenous and myogenous TMD diagnoses, and recognize common signs and symptoms associated with TMD mimickers.
5. Apply first-line management strategies for TMD, including basic self-care techniques, sleep hygiene recommendations, and appropriate pharmacotherapy.
6. Recognize when to refer patients with complex or secondary TMD diagnoses for further evaluation and management.

Citation:
Hawkins J, Cervero R, Durning SJ. Enhancing temporomandibular disorders education for initial care clinicians through interprofessional education. *MedEdPORTAL*. 2024;20:11467.
https://doi.org/10.15766/mep_2374-8265.11467

Introduction

Temporomandibular disorders (TMDs) are a group of over 30 musculoskeletal conditions associated with the masticatory muscles, temporomandibular joints (TMJ), and associated structures. TMDs are the second most common musculoskeletal pain condition (after low back pain) and impact between 4% to 18% of the US adult population.[1,2] TMDs often significantly impact a patient's quality of life. For example, given the anatomic

US Government work in the public domain. This is an open-access publication distributed under the terms of the Creative Commons Public Domain Mark.

locations that are affected by a TMD, normal daily activities such as eating, talking, brushing the teeth, smiling, and breathing may be impaired. A patient's family may also suffer, as activities such as intimacy and communication may be impacted by a TMD. Lastly, job performance may decline, as chronic pain often leads to absenteeism or presenteeism.[3] Health care utilization and care costs are also high for TMD patients, with many spending thousands of dollars for care not covered by insurance.[3,4]

TMDs are often complex and multifactorial, and they rarely occur in isolation. Many patients experience overlapping pain conditions, sleep difficulties, and mental health challenges that complicate diagnosis and management and compromise the prognosis.[1,3] Additionally, up to 50% of patients develop chronic pain after TMD onset,[1] indicating a need for early detection and prevention, as well as knowledge of chronic pain management.[3]

Despite the widespread occurrence and profound impact of TMDs, education and training within the health professions has historically been insufficient.[3,5] Most health professions schools spend little to no time teaching about TMDs or chronic pain care in predoctoral or postdoctoral programs, and there are few evidence-based advanced education resources available.[3,5,6] This leads many students to feel dissatisfied with their training and not confident in their ability to manage TMDs or chronic pain.[7,8] Educational disparities persist after graduation, as many continuing education courses prioritize outdated, nonevidence based, and aggressive treatment approaches to TMDs.[3] Moreover, accessible and comprehensive learning resources are sparse and often limited to only one facet of TMD care.[9] Collectively, this educational gap contributes to fragmented TMD care characterized by a lack of interprofessional collaboration and insufficient access to quality care. This can potentially lead to ineffective care or even iatrogenic harm, ultimately resulting in dissatisfaction among both patients and providers.[3]

Based on this gap, the National Institutes of Health commissioned the National Academies of Sciences, Engineering, and Medicine (NASEM) to address the current state of knowledge regarding TMD education and training, research, treatments, burden, and costs. A comprehensive report by NASEM was published in 2020, which made multiple recommendations to improve TMD care moving forward. Recommendation nine advocated for improved interprofessional education and training on TMDs for health professionals at undergraduate, graduate, postgraduate, and continuing education levels.[3]

To address this gap, we created a clinically oriented, interactive curriculum targeted to health professionals who are likely to be the initial evaluators of individuals with a TMD, henceforth referred to as initial care clinicians (ICCs). ICCs include, but are not limited to, physicians, physician assistants, dentists, physical therapists, and nurse practitioners. This four-module curriculum parallels recommendations by the NASEM report and emphasizes improving knowledge and attitudinal gaps related to TMD prevalence and impact, physiology, and pathophysiology (module 1); patient assessment (module 2); diagnosis (module 3); and management (module 4). The modules differ from traditional modes of didactic TMD instruction by utilizing brief, engaging videos and interactive, case-based scenarios designed using Mayer's Cognitive Theory of Multimedia Learning (CTML).[10] It also provides tools that can be incorporated clinically to optimize patient assessment, diagnosis, and management. The curriculum is scalable and adaptable to a variety of audiences, enhances interprofessional collaborative practice, and it may be used by health professional educators during various stages of student training or by practicing clinicians for continuing education.[11]

Methods

Curriculum Design

A team composed of orofacial pain experts, clinician educators, and educational specialists convened in 2023 to design a curriculum that would help address the gap in TMD education and care. Using information from the 2020 NASEM report[3] and 2023 American Academy of Orofacial Pain guidelines for assessment, diagnosis, and management,[12] as well as our team's clinical experience, we developed an interactive modular curriculum to enable ICCs across multiple health professions fields to more effectively care for patients with a TMD. The curriculum was created in a digital format to enable implementation using either an asynchronous or synchronous approach. Only the asynchronous approach was evaluated by the authors.

In developing this curriculum for ICCs, we utilized Kern's six-step model for curriculum development.[13] Building upon the recognized need to enhance provider TMD knowledge and education that was elaborated on in the 2020 NASEM report (step 1), the local needs assessment (step 2) revealed a significant gap in TMD knowledge and confidence of learners across the health professions locally. This data was predominantly gathered through discussions the primary author had with numerous department chiefs and other health professionals throughout medicine, dentistry, and the allied health professions. These conversations highlighted a clear demand for a structured educational program focused on TMD. For step 3, we established goals and objectives for the curriculum

US Government work in the public domain. This is an open-access publication distributed under the terms of the Creative Commons Public Domain Mark.

aimed at enhancing foundational knowledge and clinical skills in assessing, diagnosing, and managing TMD. Steps 4 through 6 are discussed throughout the remainder of the Methods.

Facilitators/Learners

We developed a facilitator guide (Appendix A), which provided course setup instructions and timing guidance for both asynchronous and synchronous implementation strategies. For asynchronous implementation, our team created an online course using Google Classroom Learning Management System (LMS). The Google Classroom was divided into four distinct yet interconnected modules, described below. We also created online assessments, including surveys, for each module using Google Forms software and placed links for these within the respective modules. Google LMS and software was chosen due to its no-cost model and ease of access by learners at different institutions that did not have access to another standard LMS or survey software. A course invitation was emailed to participants for enrollment. A learner guide (Appendix B) was provided to participants containing a course overview, course instructions, recommended course timing, and sample clinical evaluation tools.

Curriculum Materials

Our team designed four modules that discussed high-yield topics essential to TMD care: TMD pathophysiology (Appendix C), TMD assessment (Appendix D), TMD diagnosis (Appendix E), and TMD management (Appendix F). Each module featured a variety of learning tools aimed at facilitating participants' comprehension and clinical application of the subject matter. We introduced information in each module through brief videos (range: 5-27 minutes; average: 15 minutes) that covered critical content within its respective topical domain, with each module taking approximately 60 minutes to complete. To enhance learner comprehension, we designed the videos based on principles from Mayer's Cognitive Theory of Multimedia Learning.[10] This approach involved utilizing simple visuals (graphics, animations, callouts) and verbal explanations (via voice-over and onscreen text) to highlight key learning objectives (multimedia, signaling, and coherence principles). Additionally, we divided the videos into concise and manageable segments with user-paced control to mitigate cognitive load (segmenting principle).

After reviewing the videos in the diagnosis (Appendix E) and management (Appendix F) modules, learners completed six interactive digital simulation scenarios to facilitate the application of knowledge in a simulated clinical setting. These scenarios provided learners with explanatory feedback based on their decisions, thereby reinforcing their learning (feedback principle). Learners had unlimited opportunities to interact with the scenarios, and scores were not recorded from these. Finally, sample clinical evaluation tools (Appendix B) and patient education tools (Appendix G) were included to equip learners with the tools necessary to help immediately translate the knowledge acquired in the course into clinical practice. Learners were encouraged to practice using these tools when completing the digital simulation scenarios. The total time to complete the curriculum was approximately 5 hours.

Implementation Logistics

Using a snowball sampling approach, we recruited 30 ICCs to participate in this curriculum from September to December 2023. There was no prerequisite knowledge to participate, and most learners had minimal knowledge of TMDs. Learners consented to participate and enrolled in a Google Classroom containing course materials hosted on the facilitator's secured Google Drive account. Each learner supplied relevant practice data, including health professional category and years of experience (<1, 1-5, 6-10, >10). Learners were able to complete modules at their own pace within a 12-week window, but we recommended completing at least one module per week in sequential order.

Evaluation Strategy

Using the Google Forms links embedded in the Google Classroom modules, learners completed multiple-choice question (MCQ) assessments before and after each module to measure the change in knowledge (Appendix A). These questions were aligned with the learning objectives and adhered to the formatting guidelines outlined in the National Board of Medical Examiners (NBME) Item-Writing Guide.[14] Questions were scored as single best answer, per NBME recommendation. There were no pass/fail scores or classification of success level established.

After completing each module, learners also completed a pre/post retrospective survey (Appendix A) to measure perceived competence change based on the module's learning objectives. This approach mitigated the potential for learners to over-or-underestimate their knowledge before engaging with the learning material.[15,16] Furthermore, the postmodule assessments included questions to evaluate whether participants thought their awareness of patients at risk for developing a TMD or already having TMD increased after module completion. All assessments were completed via Google Forms, and the data was stored on the facilitator's Google Drive account. Matched quantitative data were analyzed using paired *t* tests with a significance set at $p < .05$. The Figure illustrates the curriculum structure and implementation process. This project was certified exempt by the Uniformed Services University of the Health Sciences

US Government work in the public domain. This is an open-access publication distributed under the terms of the Creative Commons Public Domain Mark.

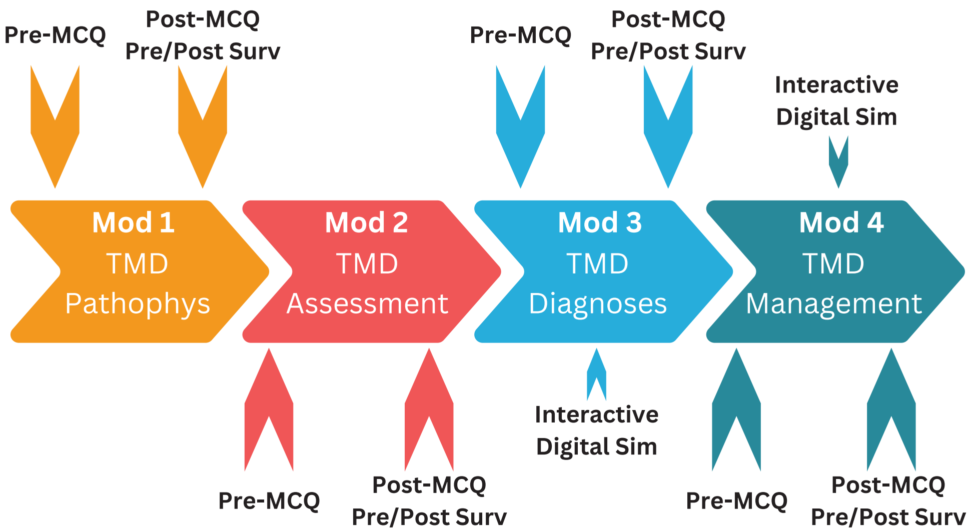

Figure. Flowchart illustrating the curriculum's modular structure, implementation process, and assessment timeline. Abbreviations: MCQ, multiple-choice questions; Surv, retrospective survey; Sim, simulation; Mod, module; TMD, temporomandibular disorders; Pathophys, pathophysiology.

Human Research Protection Program Institutional Review Board, reference number 954299, on October 13, 2022.

Results

A total of 30 learners from multiple institutions throughout the Department of Defense and the US Department of Veterans Affairs participated. Learners encompassed a spectrum of health professions: dentistry (DDS and DMD, $n = 13$), medicine (MD and DO, $n = 5$), physical therapy (DPT, $n = 5$), physician assistant ($n = 4$), and nursing (BSN and DNP, $n = 3$). There was a diverse duration of professional experience: 17% ($n = 5$) of learners recently graduated from professional school and had been practicing for less than 1 year, 30% ($n = 9$) of learners had been practicing between 1 and 5 years, 20% ($n = 6$) of learners had been practicing between 6 to 10 years, and 33% ($n = 10$) of learners had been practicing for over 10 years.

Response rates for both pre- and postmodule MCQ knowledge checks, as well as postmodule retrospective surveys, were 100% (30 of 30) for each module. Analysis of matched MCQs mean responses demonstrated a significant increase in learners' knowledge following the completion of each module (Table 1), characterized by predominantly large effect sizes ($M = 2.9$ vs. $M = 4.2$, $p < .001$; Cohen's $d = 1.2$). Similarly, matched responses from the pre/post retrospective survey (Table 2) indicated a significant enhancement in learners' perceived competence following each module, again with large effect sizes ($M = 1.9$ vs. $M = 3.4$, $p < .001$; Cohen's $d = 2.1$). The consistently large effect sizes across the MCQs and surveys, as evidence by Cohen's d being higher than 0.8, demonstrated that the change in knowledge and perceived confidence was both functionally and statistically significant. Notably, module 3 exhibited a slightly smaller, yet still statistically and functionally significant ($M = 4.6$ vs. $M = 5.1$, $p = .01$; Cohen's $d = .50$), difference in premodule versus postmodule MCQ scores compared to other modules. This discrepancy likely stems from the relative ease of the knowledge check questions in module 3, as evidenced by the higher premodule score compared to the other modules. It could also be because many ICCs in this study already had basic TMD diagnostic knowledge before taking this module. Regardless, this small difference did not impact the overall findings, as learners' perceived competence change remained substantial for module 3 ($M = 1.8$ vs. $M = 3.3$, $p < .001$; Cohen's $d = 2.2$), similar to the other three modules, signifying learners' perception of having little to no perceived competence of this material before completing the module.

Furthermore, awareness of patients at risk for developing a TMD or already having TMD notably increased postmodule completion. Following module 1 (Appendix C), learners were asked, "Based on the information in this module, do you believe you have seen patients with risk factors for developing TMD in your clinic that you previously did not realize you should assess for?" (yes/no). Ninety-three percent ($n = 28$) responded affirmatively. This likely indicates increased recognition of TMD risk factors after exposure to TMD risk factor information. Following modules 2 through 4 (Appendices D-F, respectively), learners were asked, "Based on the information in this module, do you believe you have seen patients with a TMD in your clinic that you were unaware of?" (yes/no). Ninety-three percent

US Government work in the public domain. This is an open-access publication distributed under the terms of the Creative Commons Public Domain Mark.

Table 1. Mean Scores of Matched Learner Responses to Pre- and Postmodule MCQ Knowledge Checks ($N = 30$)

Module	Premodule Score *M* (*SD*)	Postmodule Score *M* (*SD*)	*p*	Effect Size (Cohen's *d*)	95% CI
1[a]	2.2 (1.3)	4.0 (1.1)	<.001	1.6	(1.0-2.1)
2[b]	1.8 (1.1)	3.2 (1.1)	<.001	1.3	(0.7-1.8)
3[a]	4.6 (1.2)	5.1 (0.8)	.01	0.5	(0.0-1.0)
4[b]	3.0 (1.0)	4.3 (0.7)	<.001	1.5	(1.0-2.1)

Abbreviation: MCQ, multiple-choice question.
[a]Based on six MCQs.
[b]Based on five MCQs.

($n = 28$) responded affirmatively after module 2, and 90% responded affirmatively after modules 3 and 4. This likely indicates increased efficacy in recognizing patients with a TMD after exposure to TMD assessment, TMD diagnostic, and TMD management processes. Importantly, 100% ($n = 30$) of learners responded affirmatively to at least one of these questions, underscoring the tangible application of acquired knowledge to clinical practice.

Discussion

Our team successfully developed and implemented an engaging and interactive TMD curriculum, resulting in substantial enhancements in both the knowledge and perceived competence of learners across clinically relevant areas of TMD pathophysiology, assessment, diagnosis, and management, as evidenced by statistically significant findings with large effect sizes. This improvement was demonstrated across a spectrum of health professions (medicine, dentistry, physician assistant, physical therapy, and nursing) that may act as the ICCs for patients suffering from a TMD. Based on these positive findings, ICCs should have a much better understanding of TMDs, as well as their role in evaluating, managing, and appropriately referring patients with a TMD. For patients, this should arguably lead to increased access to care, improved outcomes, and decreased risk of iatrogenic harm. For clinicians, this should likely lead to improved confidence and increased satisfaction when treating patients with a TMD.

At a policy level, our curriculum directly addresses NASEM recommendation nine to improve education and training on TMDs for health professionals at undergraduate, graduate, postgraduate, and continuing education levels.[3] Based on the flexible nature of this brief, self-paced, online, no-cost curriculum, educational institutions across the health professions have the opportunity to download these materials and integrate them into their curricular plan. Additionally, professional societies can incorporate these materials into their learning management platform and incentivize participation by offering continuing education credits, thereby expanding the reach of these important educational tools.

Table 2. Mean Scores of Matched Learner Responses to Pre- and Postmodule Retrospective Surveys[a] ($N = 30$)

Survey Item	Premodule Score[a] *M* (*SD*)	Postmodule Score[a] *M* (*SD*)	*p*	Effect Size (Cohen's *d*)	95% CI
Module 1: Prevalence and impact of TMD	2.2 (0.9)	3.6 (0.5)	<.001	2.0	(1.4-2.6)
Module 1: Normal TMJ and masticatory muscle function	2.5 (0.8)	3.5 (0.5)	<.001	1.5	(0.9-2.0)
Module 1: Neuroanatomical and physiological factors related to TMDs	2.1 (0.7)	3.5 (0.6)	<.001	2.2	(1.5-2.8)
Module 1: Common TMD comorbidities and risk factors	1.8 (0.8)	3.4 (0.5)	<.001	2.5	(1.8-3.2)
Module 2: Performing a basic TMD screening in your clinical practice	1.8 (0.9)	3.3 (0.5)	<.001	2.0	(1.4-2.6)
Module 2: How to take a TMD history	2.0 (0.8)	3.5 (0.6)	<.001	2.1	(1.0-2.8)
Module 2: How to perform a TMD examination	1.9 (0.8)	3.3 (0.6)	<.001	2.0	(1.4-2.6)
Module 2: When to order/perform appropriate diagnostic tests and images	1.8 (0.8)	3.3 (0.7)	<.001	2.0	(1.4-2.6)
Module 2: Performing an auriculotemporal nerve block	1.4 (0.7)	3.1 (0.8)	<.001	2.1	(1.5-2.8)
Module 3: Signs and symptoms of common arthrogenous TMD diagnoses	1.8 (0.8)	3.3 (0.5)	<.001	2.2	(1.6-2.9)
Module 3: Signs and symptoms of common myogenous TMD diagnoses	1.9 (0.7)	3.4 (0.6)	<.001	2.2	(1.5-2.8)
Module 3: Differentiate a true TMD versus a TMD mimicker	1.8 (0.7)	3.3 (0.6)	<.001	2.3	(1.6-2.9)
Module 4: First-line TMD management strategies	2.0 (0.7)	3.5 (0.6)	<.001	2.3	(1.6-2.9)
Module 4: Self-care TMD management strategies	2.1 (0.9)	3.5 (0.6)	<.001	1.9	(1.3-2.5)
Module 4: Initial pharmacologic TMD management strategies	1.9 (0.7)	3.4 (0.7)	<.001	2.1	(1.4-2.7)
Module 4: Appropriate referral sources for complex or secondary TMDs	2.0 (1.0)	3.5 (0.6)	<.001	1.8	(1.2-2.4)

Abbreviations: TMD, temporomandibular disorders; TMJ, temporomandibular joints.
[a]Rated on a 4-point Likert-like scale (1 = *no knowledge*, 2 = *little knowledge*, 3 = *basic knowledge*, 4 = *very knowledgeable*).

US Government work in the public domain. This is an open-access publication distributed under the terms of the Creative Commons Public Domain Mark.

Despite the success of this curriculum, we encountered several challenges while developing and implementing this curriculum that resulted in lessons learned that may help with future iterations of implementation. First, using Google Classroom LMS required that each learner had a Google account, and not all potential learners wanted to create an account. Additionally, Google Classroom did not provide an option to require learners to complete the premodule MCQs before the module, and therefore learners could have completed it after the module to achieve a better score. Future implementation may be improved by utilizing an LMS that learners already have access to and that allows instructors more administrative controls. A second challenge was encouraging learners to find time to complete the course. Although the entire curriculum only took approximately 5 hours to complete, learners typically had to engage with materials after working hours. Therefore, some learners delayed completion of the course due to competing priorities. Future implementation may be enhanced by providing learners a focused block of time to complete the curriculum during working hours. A final challenge was collecting feedback for future curriculum improvements. Although some learners proactively contacted the course director via email or the chat feature in Google Classroom to provide qualitative feedback, there was no direct mechanism to collect curriculum feedback. It may be beneficial to add a predesignated feedback collection mechanism within the LMS during future implementation.

Several limitations to the evaluation of this curriculum exist. Our sample size was small, and incorporating various health professions further limited the amount of data collected from each profession. Despite this small sample size, statistically and functionally significant improvements were demonstrated for all measures in each module. Another potential limitation is the generalizability of the results, as all participants practice within governmental health care (Department of Defense or Department of Veterans Affairs). This limitation likely has little impact on the generalizability though, as all learners completed their primary health professions training (MD, DO, DDS, DMD, DPT, DNP) at various civilian institutions before working for the government, indicating that a lack of TMD education is not only found within government health care. A further limitation is limited validity and reliability evidence for the surveys utilized. An additional limitation is that the evaluation was not able to assess for change in clinical skill when evaluating, diagnosing, or managing a real patient.

While online delivery was effective for increasing learner knowledge and perceived competence, there may also be a benefit to incorporating in-person activities for future curriculum iterations. Small-group activities (described in Appendix A) may support increased learning and interprofessional collaboration, especially when working through the interactive cases in Appendices E and F. Small-group interactions could also provide learners the opportunity to practice delivering diagnosis and management education to a colleague in a controlled environment using the patient education tools (Appendix G) before delivering the same information in a real clinical scenario. There may also be benefit from incorporating hands-on instruction. For example, the auriculotemporal nerve block tutorial in module 2 (Appendix D) demonstrates how to perform this important diagnostic injection, yet most clinicians have never performed this injection and are apprehensive about trying it for the first time. Additionally, performing a clinical examination on a colleague may help reinforce muscle palpation and mandibular range of motion measurement skills learned in module 2 (Appendix D). A hybrid or fully in-person course would allow learners to practice this skill under direct supervision and ultimately increase their clinical armamentarium for improved clinical care.

In conclusion, our curriculum represents a notable step forward in addressing the educational gaps identified by the NASEM regarding TMDs. Through a theory-informed, structured, and interactive approach, our findings suggest a diverse group of health professionals were successfully equipped with the knowledge to better understand, assess, diagnose, and manage patients with TMDs. This pioneering curriculum will help prepare current and future ICCs to manage patients suffering from a TMD and fill a much-needed gap in areas where demand outweighs the availability of orofacial pain specialists.

Appendices

A. Facilitator Guide.docx

B. Learner Guide and Clinical Tools.pdf

C. Module 1 - TMD Pathophysiology.pptx

D. Module 2 - TMD Assessment.pptx

E. Module 3 - TMD Diagnosis.pptx

F. Module 4 - TMD Management.pptx

G. Sample Patient Education Tools.pptx

All appendices are peer reviewed as integral parts of the Original Publication.

US Government work in the public domain. This is an open-access publication distributed under the terms of the Creative Commons Public Domain Mark.

James Hawkins, DDS, MS, MEd-HPE: Commander, Dental Corps, United States Navy; Associate Professor, Uniformed Services University of the Health Sciences Postgraduate Dental College; Chair, Department of Orofacial Pain, Naval Postgraduate Dental School, Naval Medical Leader and Professional Development Command; ORCID: https://orcid.org/0000-0001-7422-8313

Ronald Cervero, PhD: Deputy Chair, Department of Health Professions Education, Uniformed Services University of the Health Sciences; ORCID: https://orcid.org/0000-0002-8244-8671

Steven J. Durning, MD, PhD: Chair, Department of Health Professions Education, Uniformed Services University of the Health Sciences; Vice Chair, Department of Medicine, Uniformed Services University of the Health Sciences; ORCID: https://orcid.org/0000-0001-5223-1597

Acknowledgments
The authors thank Dr. Preston Criddle, Mr. Michael Gallagher, Dr. Paul Goforth, Dr. Steve Hargitai, Dr. Dina Kurzweil, Dr. John Johnson, Dr. Anita Samuel, Dr. Abigail Schmidt, and Dr. Tom Weber for the support in creating this project.

Disclosures
None to report.

Funding/Support
None to report.

Prior Presentations
Hawkins J. Enhancing temporomandibular disorders education for initial care clinicians. Presented at: Uniformed Services University Postgraduate Dental College Federal Services Dental Educators Workshop; April 17, 2024; Bethesda, MD.

Ethical Approval
The Uniformed Services University Human Research Protections Program deemed further review of this project not necessary.

Disclaimer
The views in this article reflect the results of research conducted by the authors and do not necessarily reflect the official policy or position of the Department of the Navy, Department of Defense, Uniformed Services University of the Health Sciences, or the US Government.

We are military service members or federal/contracted employees of the US government. This work was prepared as part of our official duties. Title 17 U.S.C. 105 provides that "copyright protection under this title is not available for any work of the United States Government." Title 17 U.S.C. 101 defines a US Government work as work prepared by a military service member or employee of the US Government as part of that person's official duties.

Neither we nor any member of our families have a financial arrangement or affiliation with any corporate organization offering financial support or grant monies for this research, nor do we have a financial interest in any commercial product(s) or service(s) we will discuss in the presentation or publication.

References

1. Slade GD, Ohrbach R, Greenspan JD, et al. Painful temporomandibular disorder: decade of discovery from OPPERA studies. *J Dent Res.* 2016;95(10):1084-1092. https://doi.org/10.1177/0022034516653743
2. Facial pain. National Institute of Dental and Craniofacial Research. Updated July 2018. Accessed October 28, 2024. http://www.nidcr.nih.gov/DataStatistics/FindDataByTopic/FacialPain
3. National Academies of Sciences, Engineering, and Medicine. *Temporomandibular Disorders: Priorities for Research and Care*. National Academies Press; 2020.
4. Durham J, Breckons M, Vale L, Shen J. DEEP study: modeling outcomes and costs of persistent orofacial pain. *JDR Clin Trans Res.* 2021;8(1):16-26. https://doi.org/10.1177/23800844211063870
5. Fricton J, Chen H, Shaefer JR, et al. New curriculum standards for teaching temporomandibular disorders in dental schools: a commentary. *J Am Dent Assoc.* 2022;153(5):395-398. https://doi.org/10.1016/j.adaj.2021.11.013
6. Mezei L, Murinson BB; Johns Hopkins Pain Curriculum Development Team. Pain education in North American medical schools. *J Pain.* 2011;12(12):1199-1208. https://doi.org/10.1016/j.jpain.2011.06.006
7. De Medeiros Tormes AK, Lemos GA, Da Silva PLP, et al. Temporomandibular disorders: knowledge, competency, and attitudes of predoctoral dental students. *Cranio.* 2023;41(1): 32-40. https://doi.org/10.1080/08869634.2020.1812816
8. Yanni LM, McKinney-Ketchum JL, Harrington SB, et al. Preparation, confidence, and attitudes about chronic noncancer pain in graduate medical education. *J Grad Med Educ.* 2010;2(2):260-268. https://doi.org/10.4300/JGME-D-10-00006.1
9. Gonzalez Y, Chwirut J, List T, Ohrbach R. DC/TMD examination protocol. *MedEdPORTAL*. 2014;10:9946. https://doi.org/10.15766/mep_2374-8265.9946
10. Mayer RE. Applying the science of learning: evidence-based principles for the design of multimedia instruction. *Am Psychol.* 2008;63(8):760-769. https://doi.org/10.1037/0003-066X.63.8.760
11. Health Professions Accreditors Collaborative. *Guidance on Developing Quality Interprofessional Education for the Health Professions*. Health Professions Accreditors Collaborative; 2019. Accessed October 28, 2024. https://healthprofessionsaccreditors.org/wp-content/uploads/2019/02/HPACGuidance02-01-19.pdf
12. American Academy of Orofacial Pain. *Orofacial Pain: Guidelines for Assessment, Diagnosis, and Management*. 7th ed. Quintessence Publishing Co; 2023.
13. Thomas PA, Kern DE, Hughes MT, Chen BY, eds. *Curriculum Development for Medical Education: A Six-Step Approach.* 3rd ed. Johns Hopkins University Press; 2016.

US Government work in the public domain. This is an open-access publication distributed under the terms of the Creative Commons Public Domain Mark.

14. NBME Item-Writing Guide. National Board of Medical Examiners. Accessed October 28, 2024. https://www.nbme.org/item-writing-guide

15. Robinson S. Starting at the end: measuring learning using retrospective pre-post evaluations by Debi Lang and Judy Savageau. *AEA365* blog. July 31, 2017. Accessed October 28, 2024. https://aea365.org/blog/starting-at-the-end-measuring-learning-using-retrospective-pre-post-evaluations-by-debi-lang-and-judy-savageau/

16. Drennan J, Hyde A. Controlling response shift bias: the use of the retrospective pre-test design in the evaluation of a master's programme. *Assess Eval High Educ.* 2008;33(6):699-709. https://doi.org/10.1080/02602930701773026

Received: April 3, 2024
Accepted: August 8, 2024
Published: November 19, 2024

US Government work in the public domain. This is an open-access publication distributed under the terms of the Creative Commons Public Domain Mark.
