# Supplementary material for: Enhancing Temporomandibular Disorders Education for Initial Care Clinicians Through Interprofessional Education
Source: MedEdPORTAL. 2024 Nov 19;20:11467. doi: 10.15766/mep_2374-8265.11467 (PMC11575917; doi:10.15766/mep_2374-8265.11467)
Supplement: Supplementary file 1 — Facilitator Guide.docxLearner Guide and Clinical Tools.pdfModule 1 - TMD Pathophysiology.pptxModule 2 - TMD Assessment.pptxModule 3 - TMD Diagnosis.pptxModule 4 - TMD Management.pptxSample Patient Education Tools.pptx [file mep_2374-8265.11467-s001.zip › A. Facilitator Guide.docx]

**Directions:** Welcome to the facilitator preparation guide for “Enhancing Temporomandibular Disorders Education for Initial Care Clinicians through Interprofessional Education.” Please review this guide for recommendations on how to implement this curriculum in either an asynchronous or synchronous format, as well as an estimated timeline for completion. Additionally, this document contains multiple choice question (MCQ) assessments and pre-post retrospective surveys that can be used to assess changes in learner knowledge and perceived competence.

**Overview:** This curriculum was designed to be implemented using either an asynchronous approach via a learning management system (LMS), or synchronous approach in a classroom setting. This guide will provide implementation strategies for both approaches.

Of note, the authors have only evaluated the asynchronous implementation approach. Results from this are presented in the ESR. The synchronous implementation approach has not been evaluated.

**Asynchronous Implementation**

**Set-up:** Create an online course using Google Classroom or another LMS. Within the LMS, create four modules that sequentially present the videos from corresponding Appendices C, D, E, F. In modules three and four, include a copy of the Interactive Learning Activity PowerPoint presentations (slides 10-153 in Appendix E; slides 11-50 in Appendix F) that learners can download to practice their TMD Diagnostic and TMD Management skills.

For each module, create online pre and post-module multiple-choice question (MCQ) assessments and post-module retrospective surveys, provided on pages 2-7 of this guide (Appendix A), using Google Forms or another test-making software. Place the link for the respective MCQ assessment at the beginning and end of each module, and the respective retrospective survey link at the end of each module.

**Timing:** The curriculum takes approximately five hours for the learner to complete, with modules one and two taking approximately one hour each, and modules three and four taking approximately one- and one-half hours each. We recommend that learners complete no more than one module per sitting, with a suggested schedule of one module per week.

**Appendices:** Appendices B and G include sample clinical resources created for this curriculum that complement the information presented in modules two and four, respectively. Asynchronous learners may download these resources to assist them in immediately translating the information presented in these modules into direct patient care.

**Synchronous Implementation**

**Set-up:** Secure a classroom with a projector or large television to play the videos from Appendices C, D, E, F. Arrange classroom seating into small groups of approximately 5 learners in which they can see the screen. Each group should have:

- One laptop or large tablet with a preloaded copy of:
  - The two Interactive Learning Activity PowerPoints (slides 10-153 in Appendix E; slides 11-50 in Appendix F) on TMD Diagnosis and TMD Management.
  - Sample Patient Education Tools PowerPoint (Appendix G).
- Hard copy of the pre and post module multiple-choice question (MCQ) assessments (without highlighted answers) and post-module retrospective survey (1 copy of each per learner). These documents are provided on pages 2-7 of this document (Appendix A).
- Hard copy of the Sample TMD Clinical Tools (Appendix E). Each learner should have 6 hard copies of the TMD Essentials form (pages 9-12 of Appendix B) that they will use for Sessions 3 and 4.

**Timing:** We recommend organizing the course into 5 sessions, each lasting between one to one- and one-half hours. Sessions should take place weekly.

- **Session 1:** (~60 minutes) Course introduction. Watch the videos from Module 1 (Appendix C) as a class.
- **Session 2:** (~90 minutes) Watch the videos from Module 2 (Appendix D) as a class.
  - After the videos, break into designated small groups and review the Sample TMD Clinical Tools (Appendix B).
- **Session 3:** (~75 minutes) Watch the videos from Module 3 (Appendix E) as a class.
  - After the videos, break into designated small groups and work through the Interactive TMD Diagnostic Learning Activity PowerPoint (Appendix E). Each learner should record the information from each interactive patient (6 total) on the TMD Essentials form (pages 9-12 of Appendix B). These forms should be brought back to Session 5.
  - Learners should use the Sample Patient Education Tools (Appendix G) to practice explaining the diagnoses from each case to the members of their small group as if they were reviewing this with a TMD patient.
- **Session 4:** (~75 minutes) Watch the videos from Module 4 (Appendix F) as a class.
- **Session 5:** (~60 minutes) Break into designated small groups and work through the Interactive TMD Management Learning Activity PowerPoint (Appendix F). Each learner should continue recording the information from each interactive patient (6 total) on the TMD Essentials form they began in Session 3 (pages 9-12 of Appendix B).
  - Learners should use the Sample Patient Education Tools (Appendix G) to practice explaining the management plan for each case to the members of their small group as if they were reviewing this with a TMD patient.

**Facilitator Role:** The facilitator should review all the material before the class and be prepared to answer any questions the learners have. In addition to setting up each session, the facilitator should distribute the pre and post-MCQ assessments before and after sessions 1-4, respectively, as well as the post-module retrospective surveys after sessions 1-4. Of note: correct answers are highlighted for each MCQ in this document in order to facilitate easy grading. Remove the highlights prior to distributing.

**Pre and post-module multiple-choice question (MCQ) assessments**

*Instructions to learners:* These multiple-choice questions are designed to assess your ability to critically think about the subject. Please read each question carefully before answering. Be aware that some questions may appear to have more than one correct answer, but you are to select the answer that makes the most sense and is most correct.

***Module 1***

1. A 32-year-old man comes to your office with a 2-year history of his mouth getting stuck open twice per month when yawning, after which is able to maneuver his mandible to close his mouth within seconds. These episodes are associated with a focal right preauricular pain that lasts for 30 seconds. On examination, his is mouth opening is 60mm. Which muscle assists in opening the mandible?
2. Lateral Pterygoid
3. Masseter
4. Medial Pterygoid
5. Orbicularis Oris
6. Temporalis
7. The intermittent pain the patient experiences is associated with the temporomandibular joint. Which nerve provides >75% sensory innervation to the TMJ?
8. Auriculotemporal nerve
9. Mandibular nerve
10. Maxillary nerve
11. Temporomandibular nerve
12. Temporal nerve
13. A 40-year-old female comes to the office with a 1-year history of right maxillary sinus and maxillary tooth pain. The pain is worse upon opening, chewing, and clenching the teeth together. On examination, palpation of the right anterior temporalis replicated the patient’s maxillary sinus and tooth pains. What is the most likely mechanism of this effect?
    1. Muscle inflammation
    2. Nerve entrapment
    3. Periapical abscess
    4. Peripheral sensitization
    5. Trigeminal convergence
14. A 65-year-old man presents to the office with symptoms indicating a myocardial infarction, including chest pain, left arm pain, heartburn, and shortness of breath. The patient also reports left jaw and tooth pain. Which nerve is responsible for these facial pain symptoms?
15. Cervical nerve three
16. Facial Nerve
17. Trigeminal nerve
18. Vagus nerve
19. Vestibulocochlear nerve
20. A 52-year-old female comes to the office with a 25-year history of bilateral facial pain. The pain is a constant dull ache with an average pain intensity of 5/10. Upon examination, the patient is tender to palpation of the bilateral masseter and temporalis muscles. Which of the following comorbid conditions is this patient most likely to have?
21. Diabetes
22. Hypertension
23. Irritable bowel syndrome
24. Multiple sclerosis
25. Rheumatoid arthritis
26. A 25-year-old female comes to your office for evaluation of her temporomandibular disorder. The patient states she has a history of asthma, menstrual migraine, seasonal allergies, insomnia, and urinary tract infections preceding the onset of the TMD. Which of the following factors in the patient’s history most increased her risk for developing this condition?
27. Asthma
28. Menstrual migraine
29. Seasonal allergies
30. Insomnia
31. Urinary tract infections

***Module 2***

1. A 22-year-old woman comes to the office with a 3-year history of bilateral face pain. Which of the following information regarding this patient’s pain history is most appropriate to obtain initially?
   1. Aggravating factors
   2. Current medications
   3. Family medical history
   4. Sleep history
   5. Stressor at pain onset
2. A 55-year-old man comes to the office with a 2-year history of bilateral masseter and temporalis myalgia that occurs daily on awakening. The pain lasts approximately 1 hour. The patient also reports daytime tiredness and snoring. Which of the following additional information regarding this patient’s history is most appropriate to obtain at this time?
   1. Amount of time to sleep onset
   2. Number of awakenings per night
   3. Observation of apneas
   4. Stimulant use
   5. Total sleep time
3. A 29-year-old man comes to the office with a 3-month history of limited mouth opening. On examination, the patient can open to a maximum of 25mm. Which of the following is the most appropriate focus of the physical examination at this time?
   1. Cervical range of motion
   2. Intraoral examination
   3. Mandibular excursive movements
   4. Masticatory muscle palpation
   5. TMJ palpation
4. A 25-year-old woman comes into the office with a 4-month history of focal unilateral TMJ area pain. The pain is not provoked with mandibular movement, and there are no positive palpation findings on examination. Which of the following is the most appropriate diagnostic test to perform at this time?
   1. Diagnostic blood work
   2. Diagnostic injection of the TMJ
   3. Diagnostic image of the TMJ
   4. Diagnostic medication trial
   5. No diagnostic testing
5. A 30-year-old man comes into the office with a 4-month history of focal TMJ area pain and a grating noise on mouth opening. The patient states that both the pain and noise are progressively worsening. Which is the most appropriate diagnostic image to obtain at this time?
   1. Computerized Tomography (CT)
   2. Dental panoramic x-ray
   3. Magnetic Resonance Imaging (MRI)
   4. Single-photon emission computerized tomography (SPECT)
   5. TMJ x-ray axiolateral view

***Module 3***

1. A 29-year-old man comes to the office with a 3-month history of right sided pre-auricular clicking. The click is painless and not impacting the patient’s quality of life. On examination, the click occurs at 25mm of mouth opening, 5mm of mouth closing, and with left lateral jaw excursion. During opening the mandible deviates to the right with correction to center. Which of the following is the most likely diagnosis?
   1. Right TMJ deviation in form
   2. Right TMJ osteoarthritis
   3. Right TMJ subluxation
   4. Right TMJ disc displacement with reduction
   5. Right TMJ disc displacement without reduction
2. A 45-year-old woman comes to the office with a 6-month history of right sided jaw pain, maxillary tooth pain, and retro orbital headache. On examination, the patient opens to a maximum of 40mm with mild aggravation of the chief complaints. Excursive movements are normal and non-painful. Intraoral palpation of the right mandibular ascending ramus to the coronoid process replicates the patient’s pain complaints. Which of the following is the most likely diagnosis?
   1. Masseter myalgia
   2. Temporal tendonitis
   3. Trigeminal neuralgia
   4. TMJ arthralgia
   5. TMJ disc displacement with reduction
3. A 35-year-old man awoke 3 days ago with limited mouth opening. The patient reported a history of bilateral TMJ clicking that also stopped 3 days ago. On examination, the patient opens to a maximum of 25mm with a hard end-feel. During opening the mandible deviates to the right without correction to center. Mandibular excursion is 8mm to the right and 3mm to the left. Which of the following is the most likely diagnosis?
   1. Right masseter myospasm
   2. Right TMJ ankylosis
   3. Right TMJ luxation
   4. Right TMJ disc displacement with reduction
   5. Right TMJ disc displacement without reduction
4. A 40-year-old woman comes to the office with a 2-year history of right sided TMJ, maxillary sinus, and ear pain. On examination, these pains are replicated with wide mouth opening, as well as mandibular protrusion and left lateral excursive movements against resistance. Which of the following is the most likely diagnosis?
   1. Right lateral pterygoid myofascial pain
   2. Right masseter myalgia
   3. Right temporalis myalgia
   4. Right TMJ arthralgia
   5. Right TMJ osteoarthritis
5. A 25-year-old woman comes to the office with 12-year history of right sided jaw pain that occurs 10 days per month. The patient has no pain today. When present, the pain is accompanied by nausea and sensitivity to bright lights. On examination, the patient is able to open to 50mm pain free. All palpation findings were non-painful. What is the most appropriate differential diagnosis?
   1. Cluster headache
   2. Masseter myalgia
   3. Migraine headache
   4. Temporalis myalgia
   5. TMJ arthralgia
6. A 60-year-old man comes to the office with a 2-day history of left-sided jaw and neck pain. The pain is pressing and averages a 4/10 intensity. The pain worsens when walking upstairs. The patient’s health history includes hypertension and hyperlipidemia. On examination, the patient is able to open to 50mm pain-free. All palpation findings were non-painful. What is the most appropriate differential diagnosis?
   1. Left masseter myalgia
   2. Left TMJ arthralgia
   3. Lung neoplasm
   4. Maxillary sinusitis
   5. Myocardial infarction

***Module 4***

1. A 45-year-old woman comes to the office with a 4-year history of bilateral jaw pain. The pain is a constant ache with an average intensity of 4/10. The patient’s health history includes diagnoses of fibromyalgia and depression. On examination, the patient opens to a maximum of 40mm with mild aggravation of the chief complaint. All masticatory and cervical palpation sites were tender. Which of the following is the most appropriate initial step in this patient’s care?
   1. Dental oral orthotic (mouth guard)
   2. Education on diagnosis
   3. Oral habit awareness training
   4. Pharmacotherapy
   5. Referral to behavioral health
2. A 32-year-old man comes to the office with a 5-year history of bilateral masseter pain. The pain is a constant ache with an average intensity of 4/10. The patient also notes a 5-year history of stable mild anxiety. The patient also notes clenching the teeth together, keeping the tongue pressed to the palate, and chewing gum regularly. On examination, palpation of the bilateral masseters replicated the primary pain complaint. You have educated the patient on the diagnosis of masticatory myalgia. Which of the following is the most appropriate next step in patient care?
   1. Dental occlusal rehabilitation
   2. Dental oral orthotic (mouth guard)
   3. Injection of local anesthetic to the masseters
   4. Injection of botulinum toxin to the masseters
   5. Oral habit awareness training
   6. Referral to behavioral health
3. A 38-year-old woman comes to the office with a 20+-year history of bilateral jaw, temple, and neck area pain. The pain is a constant ache with an average intensity of 4/10. The patient’s health history includes anxiety, widespread body pain, chronic headache, and poor sleep quality. On examination, the patient opens to a maximum of 30mm with mild aggravation of the chief complaint. All masticatory and cervical palpation sites were tender. Which of the following is the most appropriate step in this patient’s care?
   1. Dental oral orthotic (mouth guard)
   2. Dental referral
   3. Multidisciplinary evaluation
   4. Pharmacotherapy
   5. Referral to behavioral health
4. The previous patient would like to utilize a medication as part of her pain management plan. The patient is not currently using any medications and has no known drug allergies. She has no known health conditions that would preclude use of any pharmacologic agent. She has had tubal ligation surgery to prevent pregnancy. Which of the following is the most appropriate pharmacotherapy?
   1. Acetaminophen (analgesic)
   2. Carbamazepine (anticonvulsant)
   3. Fluoxetine (selective serotonin reuptake inhibitor)
   4. Amitriptyline (tricyclic antidepressant)
   5. Ibuprofen (anti-inflammatory)
5. A 52-year-old man comes to the office with a 2-month history of progressively worsening right jaw pain. The pain is an intermittent sharp pain lasting a few seconds with an average intensity of 8/10. The patient also notes right jaw numbness and hearing loss. The patient’s health history includes diagnoses of anxiety, depression, and chronic low back pain. On cranial nerve screening, there was right V3 hypoesthesia and decreased hearing. The patient was able to open to 50mm comfortably and had no pertinent palpation findings. Which of the following is the most appropriate initial step in this patient’s care?
   1. Behavioral health referral
   2. Dental referral
   3. Neurology referral
   4. Otolaryngology referral
   5. Pain management referral

**Post-module retrospective surveys**

The below introductory statement and scale applies to each of the following surveys and will be sent as an instructional guide with each survey.

“The purpose of this evaluation is to assess your self-reported change in knowledge for the topics listed below from before taking Module ____ compared to after completing Module ___. Your responses will be kept confidential and be used solely for the purpose of assessing the effectiveness of the course.”

For each of the topics listed below, please check the box under the number that indicates your level of knowledge both **before** and **after** completing the course:

1 = None – I have no knowledge of the content

2 = Low – I know very little about the content

3 = Moderate – I have basic knowledge, but there is more to learn

4 = High – I consider myself very knowledgeable

**Module 1**

| **How do you rate your knowledge about the following topics:** | **Knowledge BEFORE The Class** | | | | **Knowledge AFTER The Class** | | | |
| --- | --- | --- | --- | --- | --- | --- | --- | --- |
|  | 1 | 2 | 3 | 4 | 1 | 2 | 3 | 4 |
| Q1. The prevalence and impact of TMD. |  |  |  |  |  |  |  |  |
| Q2. Normal TMJ and masticatory muscle function. |  |  |  |  |  |  |  |  |
| Q3. Anatomical, neuroanatomical, and physiological factors related to TMDs. |  |  |  |  |  |  |  |  |
| Q4. Common TMD comorbidities and risk factors. |  |  |  |  |  |  |  |  |

**Module 2**

| **How do you rate your knowledge about the following topics:** | **Knowledge BEFORE The Class** | | | | **Knowledge AFTER The Class** | | | |
| --- | --- | --- | --- | --- | --- | --- | --- | --- |
|  | 1 | 2 | 3 | 4 | 1 | 2 | 3 | 4 |
| Q1. How to take a TMD history. |  |  |  |  |  |  |  |  |
| Q2. How to perform a TMD examination. |  |  |  |  |  |  |  |  |
| Q3. When to order/perform appropriate diagnostic tests and images. |  |  |  |  |  |  |  |  |
| Q4. Performing a basic TMD screening in your clinical practice. |  |  |  |  |  |  |  |  |
| Q5. Performing an auriculotemporal nerve block. |  |  |  |  |  |  |  |  |

**Module 3**

| **How do you rate your knowledge about the following topics:** | **Knowledge BEFORE The Class** | | | | **Knowledge AFTER The Class** | | | |
| --- | --- | --- | --- | --- | --- | --- | --- | --- |
|  | 1 | 2 | 3 | 4 | 1 | 2 | 3 | 4 |
| Q1. The signs and symptoms of common arthrogenous TMD diagnoses. |  |  |  |  |  |  |  |  |
| Q2. The signs and symptoms of common myogenous TMD diagnoses. |  |  |  |  |  |  |  |  |
| Q3. Differentiate a true TMD versus a TMD mimicker. |  |  |  |  |  |  |  |  |

**Module 4**

| **How do you rate your knowledge about the following topics:** | **Knowledge BEFORE The Class** | | | | **Knowledge AFTER The Class** | | | |
| --- | --- | --- | --- | --- | --- | --- | --- | --- |
|  | 1 | 2 | 3 | 4 | 1 | 2 | 3 | 4 |
| Q1. First-line TMD management strategies. |  |  |  |  |  |  |  |  |
| Q2. Self-care TMD management strategies. |  |  |  |  |  |  |  |  |
| Q3. Initial pharmacologic TMD management strategies. |  |  |  |  |  |  |  |  |
| Q4 .Appropriate referral sources for complex or secondary TMD diagnoses. |  |  |  |  |  |  |  |  |

**Image/Animation Sources & Credits**

All images, animations, and videos in the Appendices are author owned unless otherwise cited at the end of each module PPT.
