# Supplementary material for: Enhancing Temporomandibular Disorders Education for Initial Care Clinicians Through Interprofessional Education
Source: MedEdPORTAL. 2024 Nov 19;20:11467. doi: 10.15766/mep_2374-8265.11467 (PMC11575917; doi:10.15766/mep_2374-8265.11467)
Supplement: Supplementary file 1 — Facilitator Guide.docxLearner Guide and Clinical Tools.pdfModule 1 - TMD Pathophysiology.pptxModule 2 - TMD Assessment.pptxModule 3 - TMD Diagnosis.pptxModule 4 - TMD Management.pptxSample Patient Education Tools.pptx [file mep_2374-8265.11467-s001.zip › B. Learner Guide and Clinical Tools.pdf]

# Enhancing Temporomandibular Disorders Education for Initial Care Clinicians
## Learner Guide

**Directions:** Welcome to the learner guide for "Enhancing Temporomandibular Disorders Education for Initial Care Clinicians through Interprofessional Education." Please review this guide for a course overview, curriculum timing, available module resources, a glossary of common course abbreviations, and multiple clinical tools that can be immediately implemented in your clinical practice to enhance assessment, diagnosis, and management of a TMD patient. These tools are elaborated on in the videos within the curriculum.

**Purpose:** Temporomandibular Disorders (TMD) are common musculoskeletal pain conditions that can significantly impact a person's quality of life. TMD are often complex and multifactorial, and they rarely occur in isolation. Many patients with a TMD also experience overlapping pain conditions, sleep difficulties, and mental health challenges that complicate diagnosis and management and ultimately compromise the prognosis.

Due to the unique anatomical location of TMD, patients may present to a variety of health professionals for initial care. This includes, but is not limited to, physicians, physician assistants, dentists, physical therapists, and nurse practitioners. As an initial care clinician, this curriculum was designed to enhance your knowledge, skills, and abilities to understand, evaluate, diagnose, and manage TMD.

**Course Overview:** This course may be offered either asynchronously via a Learning Management System (LMS) or synchronously in a classroom setting. Both methods provide you with the opportunity to:

1) Experience a concise overview of TMD Pathophysiology, Assessment, Diagnosis, and Management topics delivered by experts in the field through a focused video series.
2) Interact with six digitally simulated patients who present with common TMD diagnoses and risk factors that are likely to present in your clinical practice.
3) Access and utilize clinical evaluation tools the patient education tools that facilitate immediate translation of the course materials to your clinical practice.

The primary differences between the two delivery methods include:

- Asynchronous: Self-paced, globally accessible, work through interactive cases on your own, may access the material multiple times to review content
- Synchronous: Scheduled class sessions, work through interactive cases as a small group, practice utilizing sample patient education tools on small group classmates

**Timing:** The course is composed of four modules that include a total of 19 brief videos overviewing various facets of TMD. If you are taking this course asynchronously, we recommend that you complete one module per week to minimize cognitive overload and optimize your understanding of the concepts.

- Module 1: (~60 minutes) TMD Pathophysiology
- Module 2: (~90 minutes) TMD Assessment
- Module 3: (~75 minutes) TMD Diagnosis
- Module 4: (~90 minutes) TMD Management

**Resources:** Modules 3 and 4 contain Interactive Learning Activities, available as a PowerPoint file, that allow you to practice evaluating, diagnosing, and managing six digitally simulated TMD patients. Module 4 builds upon the patient diagnoses discovered in module 3, and therefore should be completed second. Download these PowerPoints and use presenter mode to work through each case. It is recommended the cases be worked through sequentially, and that they be completed in one sitting. This should take approximately 60 minutes.

Sample Clinical Evaluation Tools (Appendix B; see below) and Patient Education Tools (Appendix G) have been provided for you to practice with during the simulated cases, as well as utilize afterward in your clinical practice. Download these materials and practice utilizing them when evaluating and managing the digitally simulated TMD patients noted above.

## Glossary of Terms

**<u>AAOP:</u>** American Academy of Orofacial Pain

**<u>ABOP:</u>** American Board of Orofacial Pain

**<u>CC:</u>** Chief Complaint

**<u>COPC:</u>** Chronic Overlapping Pain Condition

**<u>CPAP:</u>** Continuous Positive Airway Pressure

**<u>AAOP:</u>** American Academy of Orofacial Pain

**<u>CTML:</u>** Cognitive Theory of Multimedia Learning

**<u>DC/TMD:</u>** Diagnostic Criteria for Temporomandibular Disorders

**<u>GAD-7:</u>** Generalized Anxiety Disorder – 7 Item Questionnaire

**<u>IASP:</u>** International Association for the Study of Pain

**<u>IBS:</u>** Irritable Bowel Syndrome

**<u>ICC:</u>** Initial Care Clinician

**<u>ICOP:</u>** International Classification for Orofacial Pain

**<u>IPC:</u>** Interprofessional Collaboration

**<u>IPE:</u>** Interprofessional Education

**<u>Multi-D ICC:</u>** Interdisciplinary Initial Care Clinician

**<u>NASEM:</u>** National Academies of the Sciences, Engineering, and Medicine

**<u>NSAID:</u>** Nonsteroidal Anti-inflammatory

**<u>OFP:</u>** Orofacial Pain

**<u>PHQ-4:</u>** Patient Health Questionnaire – 4 Item Questionnaire

**<u>PII:</u>** Personally Identifiable Information

**<u>PTSD:</u>** Post-Traumatic Stress Disorder

**<u>SNRI:</u>** Serotonin Norepinephrine Reuptake Inhibitor

**<u>SSRI:</u>** Selective Serotonin Reuptake Inhibitor

**<u>TAC:</u>** Trigeminal Autonomic Cephalalgia

**<u>TBI:</u>** Traumatic Brain Injury

**<u>TCA:</u>** Tricyclic Antidepressant

**<u>TMD:</u>** Temporomandibular Disorders

**<u>TMJ:</u>** Temporomandibular Joint

# Comprehensive TMD Questionnaire

***Please complete all questions, as this helps us understand how we can best serve you.***

**Name** ______________________________________________ **Exam Date**________________

**DOB** ________________ **Gender:** - **Ethnicity**__________________

**Phone (H)** (_____)_____________ **(W)** (_____)_____________ **(Cell)** (____)_______________

**Email** ______________________________________________________________________

**The provider who referred you for this evaluation?** ________________________________________

**Is this evaluation for one of the following:** -

**Why are you here? Describe your pain or problem(s):**

_____________________________________________________________________________

_____________________________________________________________________________

**When and how did your pain /problem(s) start?**

_____________________________________________________________________________

_____________________________________________________________________________

**Who have you seen for your pain problem(s)?**

(Hold "Ctrl" key to select multiple)

-
Dentist
Primary Care
Neruology
ENT
Pain Clinic
Physical Therapy
Chiropractic
Other

**What treatments and/or medications have you received for this pain problem(s)?**

_____________________________________________________________________________

_____________________________________________________________________________

**What do you think is wrong or causing your pain/problem(s)? What do you think needs to be done about it?**

_____________________________________________________________________________

_____________________________________________________________________________

**Why did you decide to seek care at this time?**

_____________________________________________________________________________

_____________________________________________________________________________

Image is Public Domain

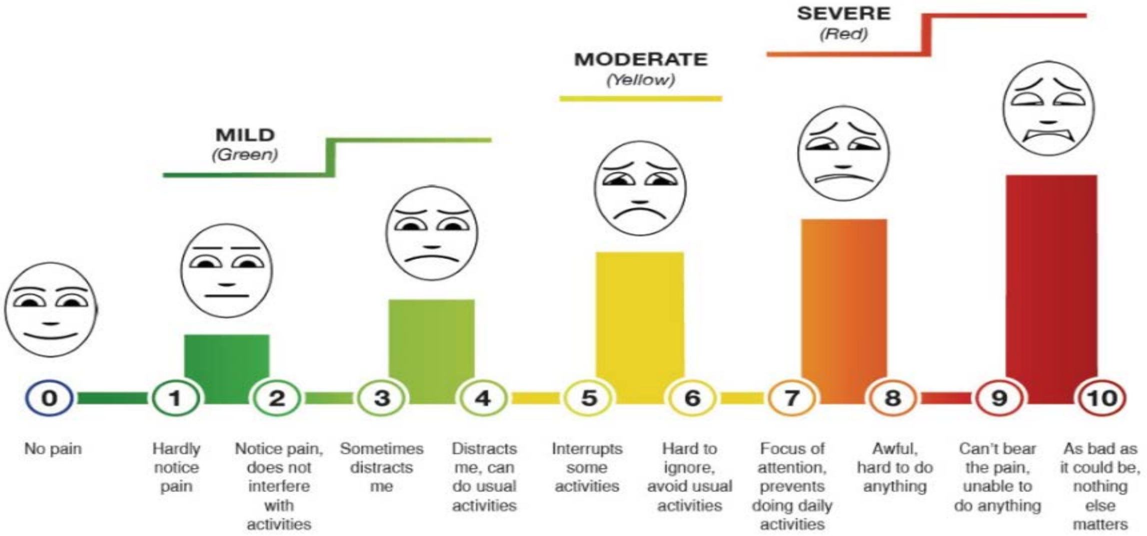


**What is your level of pain from <u>the painful area that is the main reason for your visit</u>?**

| | No discomfort 0 | 1 | 2 | 3 | 4 | 5 | 6 | 7 | 8 | 9 | Worst pain imaginable 10 |
|---|---|---|---|---|---|---|---|---|---|---|---|
| 1. **Today** | ○ | ○ | ○ | ○ | ○ | ○ | ○ | ○ | ○ | ○ | ○ |
| 2. **At its Worst** | ○ | ○ | ○ | ○ | ○ | ○ | ○ | ○ | ○ | ○ | ○ |
| 3. **Average** | ○ | ○ | ○ | ○ | ○ | ○ | ○ | ○ | ○ | ○ | ○ |

**Any pain free days?** _ **When were you last completely pain free**? ________________

**Check the word(s) that describe your pain or problem(s)?**

☐ Sharp ☐ Burning ☐ Electric-like ☐ Aching ☐ Throbbing ☐ Dull ☐ Pulsing ☐ Pressing ☐ Stabbing ☐ Tingling

## Please Rate Your Pain Interference:

4. **In the past 7 days, how much has your pain interfered with your general activities?**

| No Interference 0 | 1 | 2 | 3 | 4 | 5 | 6 | 7 | 8 | 9 | Unable to perform any activities 10 |
|---|---|---|---|---|---|---|---|---|---|---|
| ○ | ○ | ○ | ○ | ○ | ○ | ○ | ○ | ○ | ○ | ○ |

5. **In the past 7 days, how much has your pain interfered with your enjoyment of life?**

| No Interference 0 | 1 | 2 | 3 | 4 | 5 | 6 | 7 | 8 | 9 | Unable to enjoy life 10 |
|---|---|---|---|---|---|---|---|---|---|---|
| ○ | ○ | ○ | ○ | ○ | ○ | ○ | ○ | ○ | ○ | ○ |

6. **In the past 6 months how much has your pain interfered with your ability to work including housework?**

| No Interference 0 | 1 | 2 | 3 | 4 | 5 | 6 | 7 | 8 | 9 | Unable to perform any activities 10 |
|---|---|---|---|---|---|---|---|---|---|---|
| ○ | ○ | ○ | ○ | ○ | ○ | ○ | ○ | ○ | ○ | ○ |

7. **About how many days, in the last six months, have you been kept from your usual activities (work, school and/or housework) because of your pain?** ______________________________________________

8. **What does your pain limit you from doing?**

______________________________________________________________________

## Pain Modifiers:

**What starts your pain?** ____________________________________________________________________

**What makes your pain worse?** ______________________________________________________________

**What makes your pain better?** ______________________________________________________________

**Does anything else happen when your pain is present (swelling, change in vision, nausea, etc.)?** ___________

______________________________________________________________________________________

**Outline/draw the location(s) of <u>ANY AND ALL BODY PAIN</u> that you are experiencing.**

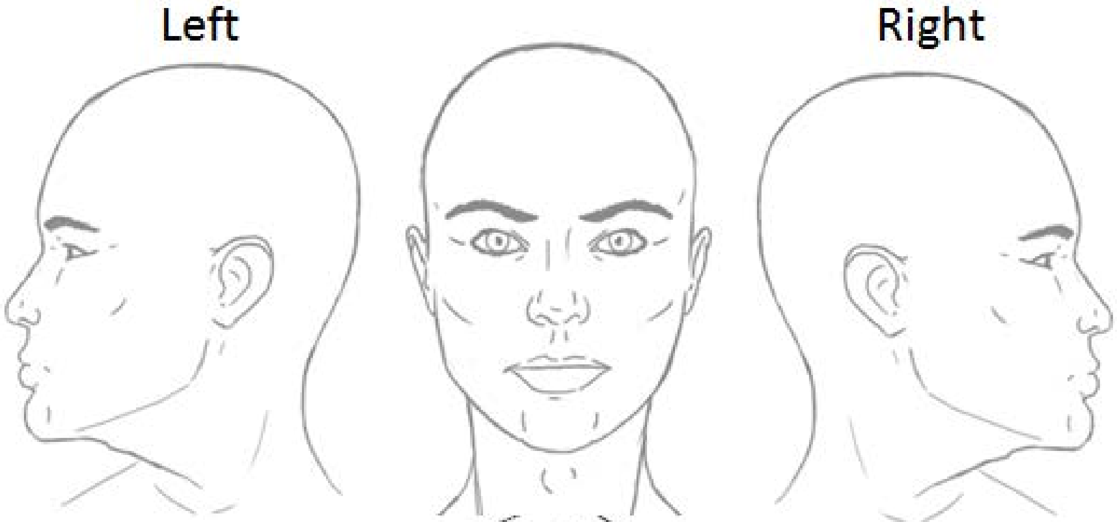


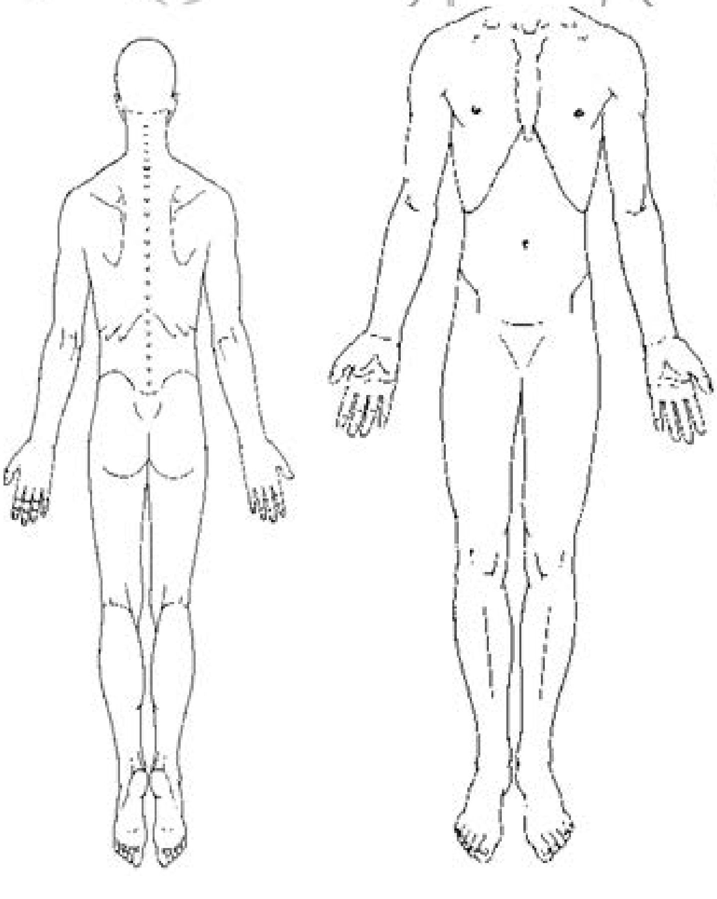

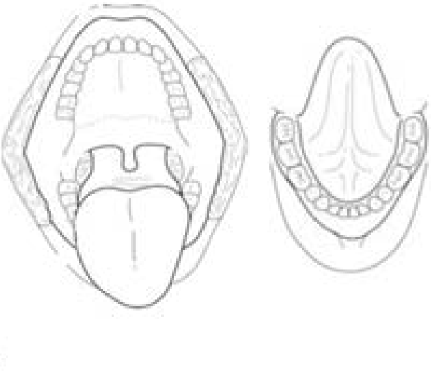

**List <u>ALL</u> of your pain problems (worst pain first then add others in decreasing order of severity):**

1. ______________________________
2. ______________________________
3. ______________________________
4. ______________________________
5. ______________________________

**Which pain occurred first?**

______________________________

**What is your overall level of total body pain?**

| | No discomfort | | | | | | | | Worst pain imaginable | |
|---|---|---|---|---|---|---|---|---|---|---|
| | **0** | **1** | **2** | **3** | **4** | **5** | **6** | **7** | **8** | **9** | **10** |
| 1. **Today** | ◯ | ◯ | ◯ | ◯ | ◯ | ◯ | ◯ | ◯ | ◯ | ◯ | ◯ |
| 2. **Worst** | ◯ | ◯ | ◯ | ◯ | ◯ | ◯ | ◯ | ◯ | ◯ | ◯ | ◯ |
| 3. **Average** | ◯ | ◯ | ◯ | ◯ | ◯ | ◯ | ◯ | ◯ | ◯ | ◯ | ◯ |

**Any pain free days?** - **When were you last completely pain free?** ________________

## Medical History

**Medical Conditions:** ____________________________________________________________

____________________________________________________________

**Allergies:** ____________________________________________________________

**History of hospitalizations?**____________________________________________________________

____________________________________________________________

**History of injury or trauma?** - ____________________________________________________________

**Have you ever had a traumatic brain injury (TBI) or a concussion?** -

**If yes, when?** ______________ **How did it occur?**____________________________________________

**If yes, did it happen on a military deployment?** -

**Current prescription medications**: ____________________________________________________________

____________________________________________________________

**Current non-prescription medications**: ____________________________________________________________

**Herbal/Dietary supplements and Vitamins**:____________________________________________________________

**History of family medical conditions** (headache, fibromyalgia, etc)? ______________________________________

## Personal Information

**Nicotine** - How long? _____ cigarettes _____/day cigars ☐ pipe ☐ snuff ☐ vap ☐

**Alcohol** - beer ____ /day wine _____ glasses/day liquor ____ drinks/day

**Caffeine** - cups(cans)/day _____ coffee ☐ tea ☐ soda ☐ chocolate ☐ pre-workout ☐ energy drinks ☐

**Water** - _______ glasses or bottles/day

**Do you skip any meals?** - **Which?** ☐ Breakfast ☐ Lunch ☐ Dinner

**Weight**: ______lbs **Height**: ____ft___inches **Neck size**:____inches **Recent weight gain/loss**? -

**Exercise level:** - **Any activity limitations?**

**Type of exercise** _______________ **Frequency**________________ **Duration** ______________

**Please estimate how many hours a day (0 to 24 hours) that your teeth touch in any contact** ________________

**What is your typical tongue position?** -

**Do you clench or grind your teeth?** -

**If yes, how do you know?** ☐ self-aware ☐ told by dentist ☐ told by others

**Oral Habits?** ☐ bite your nails ☐ chew gum ☐ protrude jaw ☐ other habits

____________________________________________________________

**Please rate your levels of:**

| | 0 None | 1 | 2 | 3 | 4 | 5 | 6 | 7 | 8 Worst possible | 9 | 10 |
|---|---|---|---|---|---|---|---|---|---|---|---|
| **Stress** | ◯ | ◯ | ◯ | ◯ | ◯ | ◯ | ◯ | ◯ | ◯ | ◯ | ◯ |
| **Anxiety** | ◯ | ◯ | ◯ | ◯ | ◯ | ◯ | ◯ | ◯ | ◯ | ◯ | ◯ |
| **Depression** | ◯ | ◯ | ◯ | ◯ | ◯ | ◯ | ◯ | ◯ | ◯ | ◯ | ◯ |
| **Anger** | ◯ | ◯ | ◯ | ◯ | ◯ | ◯ | ◯ | ◯ | ◯ | ◯ | ◯ |

**Have you ever thought of harming yourself?** - **Is this current?** -

## Personal/Family History

**Occupation**: ______________________________________________________________________

**Marital status:**

**Children**: If yes, list ages ____________________________________________________

**Are there any special needs or circumstances involving you, your family members or your job?**

______________________________________________________________________________

______________________________________________________________________________

**Do you have a history of the following or similarly threatening, stressful or frightening life events?** -

**Abuse** - at any age (physical, emotional or sexual), childhood neglect, physical or sexual assault, motor vehicle accident, deployment to a conflict zone, panic attacks, near drowning, other __________________

______________________________________________________________________________

______________________________________________________________________________

______________________________________________________________________________

**Have you been told that you have post-traumatic stress symptoms (PTSS) or disorder (PTSD)?** -

If yes, when?_____________________________________________________________________

**Do you have social/home support?** - If yes, whom? ______________________________________

**Did you grow up religious?** - If yes, are you still practicing? ________________________________

**Do you have unforgiveness/bitterness towards anyone?** _____________________________________

## Headaches

**Do you have problems with headaches?** - **For how long?** ___________________________________

**Family history of headaches?**

**Do you have more than one kind of headache?** - **If yes, how many kinds?**___________________

**Please describe each type of headache you experience.**

| | #1 | #2 | #3 |
|---|---|---|---|
| **Where on your head does the headache occur?** | | | |
| **Average pain level 0 (no pain) to 10 (worst ever)** | | | |
| **How often do they occur? (daily, weekly, monthly)** | | | |
| **When do they occur? (morning, evening, etc.)** | | | |
| **How long do they last? (secs, mins, hours, days)** | | | |
| **What starts (triggers) your headache?** | | | |

**With a headache, do you experience?** (select all that apply)

- (Hold "Ctrl" key to select multiple)

Nausea
Vomitting
Light Sensitivity
Sound Sensitivity
Aura
Change in vision
Other

## Do you experience any of the following?

**Neck pain?** - **Neck sounds?** -

**If yes, when did it start?** ____________________ **When is it the worst?** __________________________

**Pain from areas below your shoulders?** - If yes, where? _____________________________________

**Dizziness or lightheadedness?** - ________________________________________________________

**Ear problems?** ☐ fullness ☐ stuffiness ☐ ringing ☐ sounds ☐ pain

**Numbness or tingling?** ☐ around mouth ☐ head/face ☐ arms/fingers ☐ legs/toes ☐ other

**Jaw pain?** - ________________________________________________________________

**Tooth pain?** - ______________________________________________________________

**Changes in your bite?** - _______________________________________________________

**Altered jaw movement(s)?** - ___________________________________________________

**Jaw joint (TMJ) sounds?** - **If yes, is it?** ☐ popping ☐ clicking ☐ grating/grinding ☐ other

**Did jaw joint (TMJ) sounds begin before your pain started?** -

**Have there been any changes in the jaw sounds?** ___________________________________________

**If you have jaw pain or stiffness, when is it the worst?** -

**Does your jaw problem affect your ability to eat?** -

## Sleep History

**How many hours do you sleep?** Average night _______Good night _________ Bad night ________

**How long does it take to fall asleep?** Average night _______Good night _________Bad night _________

**Do you awaken at night?** - **If so, how many times?**___________

**Do you have a regular/consistent sleep schedule?** - **Hours** ____________ to_________________

**Do you snore or have a history of sleep apnea?** - **Diagnosis Date:** _________________________

**Do you sleep using a CPAP &/or an oral device for sleep apnea?** - **Type:** ___________________

**Is your obstructive sleep apnea** -

**What position do you fall asleep in?** -

**Do you have problems with nightmares?** - **If yes, are they recurring?** -

**What are the words that best describe your sleep?** -

**Do you consider your sleep to be restful or restorative?** -

**Please check the most appropriate box concerning your sleep during the last 4 weeks.**

| | No, not in last 4 weeks | Yes, less than once a week | Yes, 1 or 2 times a week | Yes, 3 or 4times a week | Yes, 5 or more times a week |
|---|---|---|---|---|---|
| **Did you have trouble falling asleep?** | ☐ | ☐ | ☐ | ☐ | ☐ |
| **Did you wake up several times a night?** | ☐ | ☐ | ☐ | ☐ | ☐ |
| **Did you wake up earlier than you planned?** | ☐ | ☐ | ☐ | ☐ | ☐ |
| **Did you have trouble getting back to sleep after you woke up too early?** | ☐ | ☐ | ☐ | ☐ | ☐ |

**Please list any additional information that you feel is important for us to know about you, your pain complaint or other aspects of your visit.**

______________________________________________________________________

______________________________________________________________________

______________________________________________________________________

______________________________________________________________________

RESET FORM

This work was prepared as part of official military duties. This document reflects the views of the authors and does not reflect the policies of the U.S. Government, Department of Defense, U.S. Navy, or Uniformed ServicesUniversity of the Health Sciences.

# TMD Essentials

## **History** - Relevant Chief Complaint Information

**Onset**: ______________________________ Trauma? Y / N Stressor @ Onset? Y / N

______________________________

______________________________

______________________________

**Previous Treatment**: ______________________________

**Impact**: **P**ain (Intensity): __/10 **E**njoyment (Interference): __/10 **G**eneral Activity (Interference): __/10

**Chief Complaint Description**

| | |
|---|---|
| Location | |
| Character | |
| Frequency / Duration | |
| Temporal Pattern | |
| Intensity | Now: /10 Avg: /10 Worst: /10 |
| Aggravating | |
| Alleviating | |
| Associated Sx | |

**Med History / Meds:** ______________________________

**Perpetuating Factors:** ______________________________

**Sleep Difficulties? Y/N** ______________ (Y - Sleep Quality: __/10, ESS: __/24, STOP-BANG: __/8)

**Sleep Hygiene:** ______________________________

**Body Pain? Y/N** Fibromyalgia, Head, Neck, Stomach, Pelvic, Back, Other ________(Y - CSI: __/100)

**Psych/Social Vulnerability? Y/N** ______________ (Y - PHQ-4: __/12, GAD-7: __/21, ACE: __/10)

**Stress Level:** (__/10) __________, **Job:** __________, **Family Status:** __________

**Activity Level:** ______________________________

**Oral Parafunction? Y/N** Teeth Together Y/N, Tongue to Palate Y/N, Nail Biting Y/N, Other________

**Hydrated? Y/N, Nutrition:** ______________Caffeine Y/N, Nicotine Y/N, Alcohol Y/N

# Examination

General Appearance: ______________________________, **Red flag(s)?** N/Y(refer)

## **Palpation Pain** (Includes discomfort or tenderness)

| Muscle | Left | Right | Familiar | Referral (location) |
|---|---|---|---|---|
| Masseter | ▢ | ▢ | ▢ | ▢ |
| Temporalis | ▢ | ▢ | ▢ | ▢ |
| Temporal Tendon | ▢ | ▢ | ▢ | ▢ |
| TMJ (static/dynamic) | ▢ | ▢ | ▢ | ▢ |
| Trapezius | ▢ | ▢ | ▢ | ▢ |
| SCM | ▢ | ▢ | ▢ | ▢ |
| Other ___________ | ▢ | ▢ | ▢ | ▢ |

## Jaw Range of Motion

**Opening**: Comfortable _________ mm

Unassisted __________ mm Painful? Y / N Familiar? Y / N Location ________

Assisted ____________ mm Painful? Y / N Familiar? Y / N Location ________

**Excursive**:

Protrusive ________ mm Painful? Y / N Familiar? Y / N Location ________

Left _____________ mm Painful? Y / N Familiar? Y / N Location ________

Right ____________ mm Painful? Y / N Familiar? Y / N Location ________

## Jaw Opening Pattern

- ▢ Straight
- ▢ Corrected Deviation ( R / L )
- ▢ Uncorrected Deviation ( R / L )

## TMJ Noises

▢ Click/Pop ( R / L ) Painful ( Y / N ) Familiar ( Y / N )
▢ Crepitus ( R / L ) Painful ( Y / N ) Familiar ( Y / N )

**Cervical Screening** Limited Movement (Y/N), Pain (Y/N), Familiar (Y/N)____________

**Intraoral Screening** (Teeth / Gingiva / Other), Recent Dental Work? Y/N ___________

________________________________________________

# Diagnostic Testing

Panoramic ______ CT/CBCT ______ TMJ MRI ______ AT Nerve Block ______ Other ________

Findings: ______________________________________________

# Diagnoses

| Pain Disorder(s) | Notes |
|---|---|
| ☐ None | |
| ☐ Myalgia (R/L)<br>☐ Temporal Tendonitis (R/L)<br>☐ Myofascial Pain w/ Referral<br>☐ Centrally-Mediated Myalgia | |
| ☐ TMJ Arthralgia (R/L) | |

| TMJ Disorder(s) | Notes |
|---|---|
| ☐ None | |
| **Disc Displacement**<br>☐ w/ Reduction (R/L)<br>☐ w/ Red. w/ Intermittent Locking (R/L)<br>☐ w/o Red., w/ limited opening (R/L)<br>☐ w/o Red., w/o limited opening (R/L) | |
| ☐ TMJ Subluxation (R/L) | |
| ☐ Degenerative Joint Disease (R/L) | |

**Notes**: ______________________________________________

# Assessment

______________________________________________

______________________________________________

______________________________________________

**Prognosis**: Good, Guarded, Poor

# Plan

| Management Option(s) | Notes and Resources |
|---|---|
| ☐ **Education** | ☐ Diagnoses<br>☐ Physiology & Risk Factors (provide poster handout) |
| ☐ **Self-Care** | ☐ Habit Awareness Training (provide infographic & video)<br>☐ Heat, Massage, Gentle Movement, Diet Modification<br>☐ Sleep Hygiene Instruction<br>☐ Physical Activity<br>☐ Nutrition Modification<br>☐ Diaphragmatic Breathing Training |
| ☐ **Medication** | ☐ Topical Diclofenac (1-2 week trial for TMJ Arthralgia)<br>☐ NSAID (1-2 week clock-regulated trial for TMJ Arthralgia)<br>☐ Muscle Relaxant(s) (2-4 week trial - medication taken daily)<br>    ☐ Methocarbamol (daytime)<br>    ☐ Cyclobenzaprine (nighttime)<br>☐ Tricyclic Antidepressant (3-6 month trial - medication taken daily)<br>    ☐ Amitriptyline (nighttime)<br>    ☐ Notriptyline (nighttime)<br>☐ Gabapentinoid<br>    ☐ Gabapentin (nighttime or t.i.d.) |
| ☐ **Intervention** | |
| ☐ **Referral** | ☐ Physician (Primary)<br>☐ Dentist<br>☐ Physical Therapy<br>☐ Occupational Therapy<br>☐ Mental Health<br>☐ Sleep Medicine<br>☐ Nutrition<br>☐ Orofacial Pain<br>☐ Specialty Care - Other ________________________________ |

**Follow-up Timeline:** 1 week, 1 month, Other ________________________________

**Follow-up Considerations:** ________________________________

________________________________

________________________________

This work was prepared as part of official military duties. This document reflects the views of the authors and does not reflect the policies of the U.S. Government, Department of Defense, U.S. Navy, or Uniformed Services University of the Health Sciences.

This work was prepared as part of official military duties.

(Hawkins, 2023)

# DIAGNOSIS

## Muscle Pain

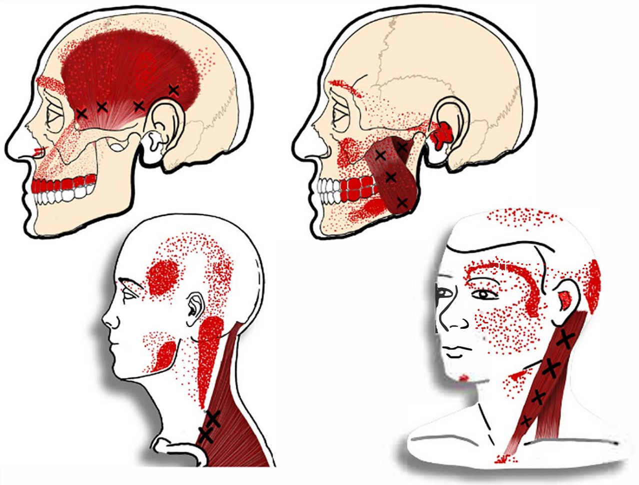

## TMJ Disorder

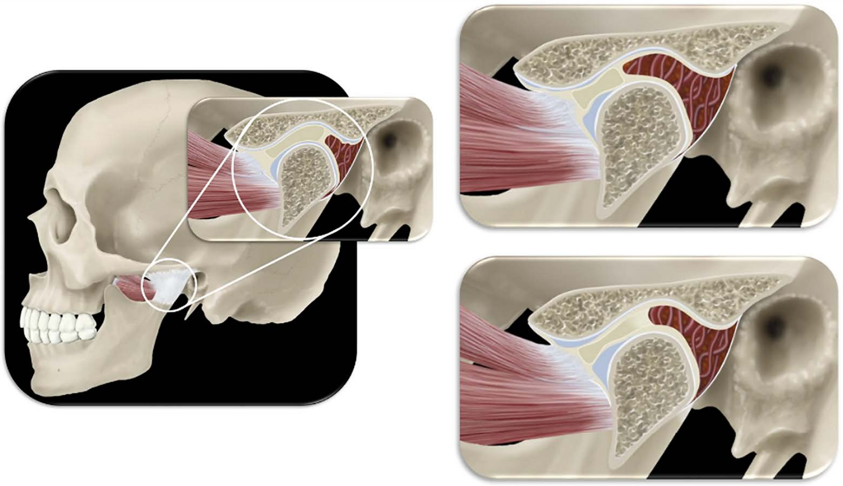

# RISK FACTORS

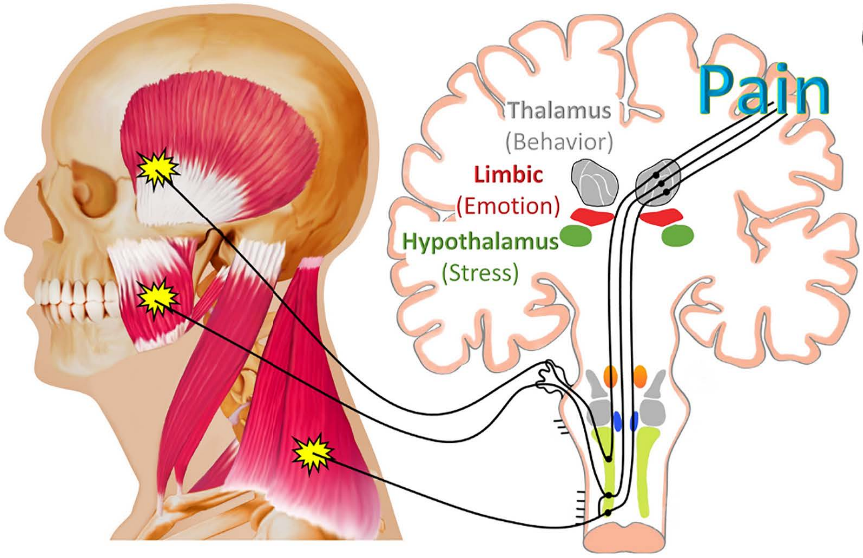


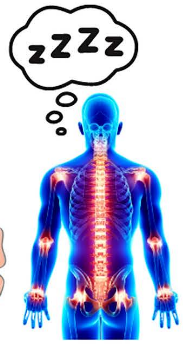


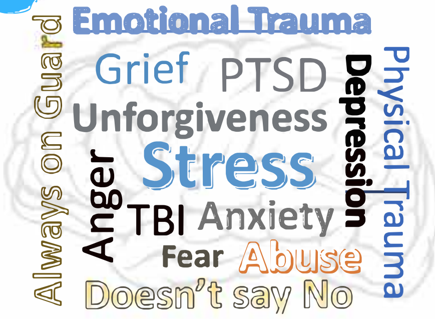


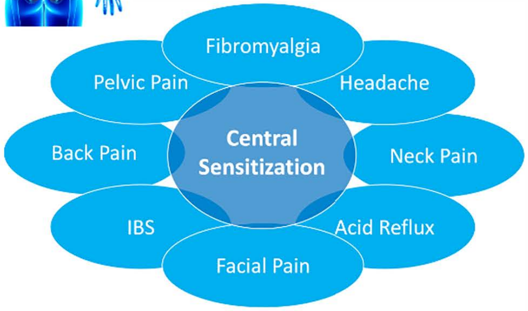


# MANAGEMENT

-Rehabilitation involves a team approach between you and your doctors

-Referrals to appropriate specialists may be beneficial to your overall care

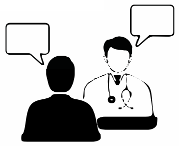

## Self-Care

-Healthy lifestyle behaviors & modifying risk factors are very important

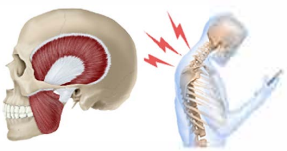

Maintain a Position of Rest

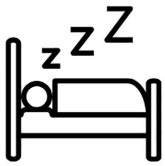

Practice Healthy Sleep Hygiene

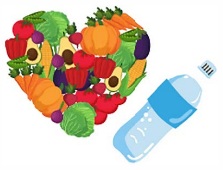

Healthy Nutrition & Hydration

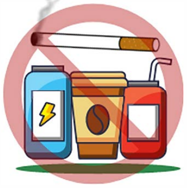

Minimize Stimulants

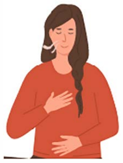

Breathe

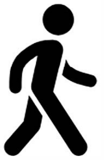

Exercise

This document reflects the views of the authors and does not reflect the policies of the U.S. Government, Department of Defense, U.S. Navy, or Uniformed Services University of the Health Sciences.

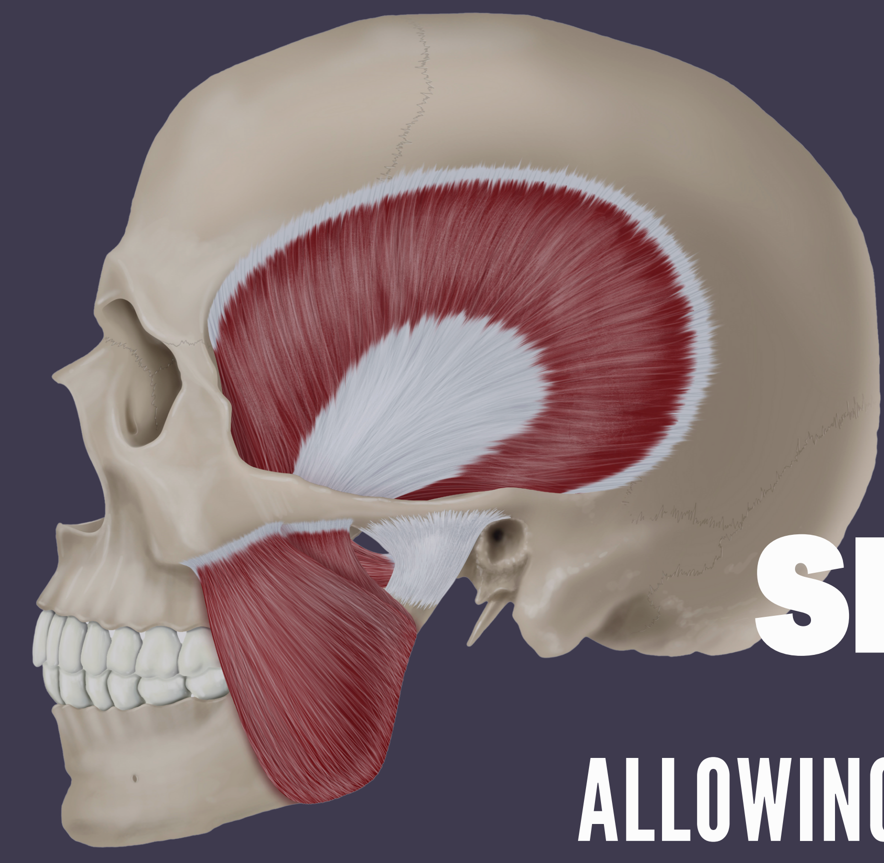

This work was prepared as part of official military duties. This document reflects the views of the authors and does not reflect the policies of the U.S. Government, Department of Defense, U.S. Navy, or Uniformed Services University of the Health Sciences.

# TMD SELF-CARE

## ALLOWING HEALING TO OCCUR

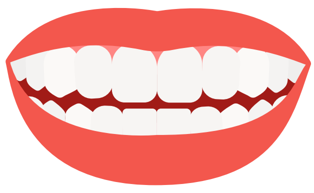

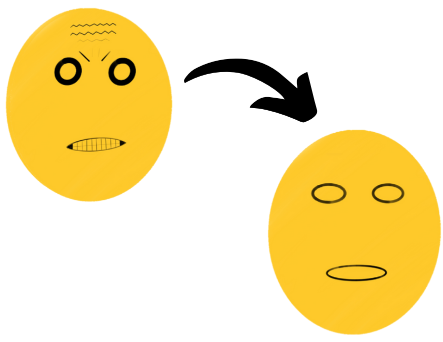

### REST POSITION

Teeth Apart
Tongue Relaxed
Face Relaxed
Neck & shoulders relaxed

Use a Reminder 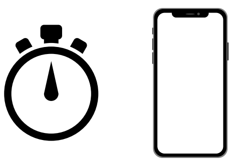

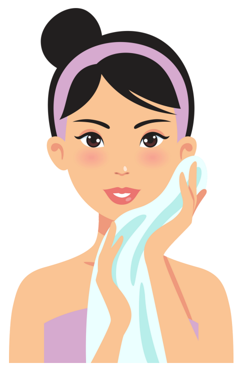

### APPLY HEAT & MASSAGE

Place heat on the painful muscles 2-4 x per day for 3-5 minutes

Gently massage the muscles

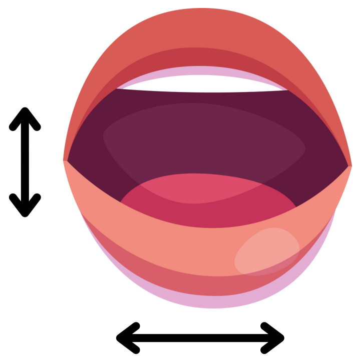

### GENTLE MOVEMENT

Gently move your mouth up & down, side to side

Stretch using your fingers, but do not increase your pain

Learn to listen to your body

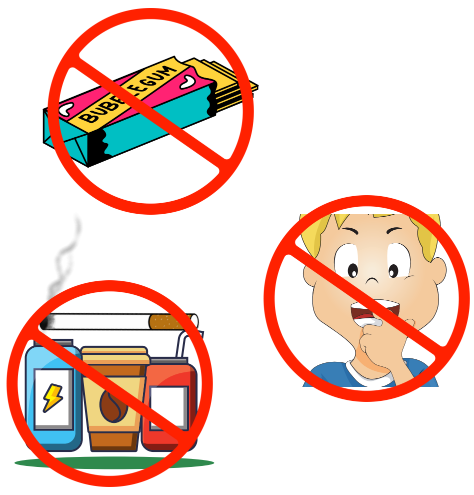

### CAUTION

Be mindful when using your jaw:

- Avoid gum, nail biting, etc
- Minimize aggravating foods
- Avoid stimulants

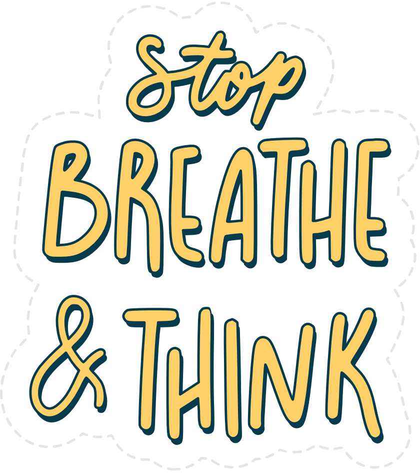


### TAKE BREAKS

Take a break from your daily tasks:

- Belly breathe
- Go for a walk
- Stretch

(Hawkins, 2023)

# TMD Exam Form Part II

## Patient Summary

**History of Present Illness (HPI):** (onset, change over time, precipitated by trauma/life changes?)

**Description of Pain Complaint(s):**

| Pain Complaint | Primary | Secondary | Tertiary |
|---|---|---|---|
| Location | | | |
| Character<br>(quality) | | | |
| Intensity (0-10)<br>(today, avg, worst) | | | |
| Frequency<br>(daily, weekly, etc.)<br>Temporal pattern | | | |
| Duration<br>(secs, mins, hrs, days) | | | |
| Precipitating<br>Factors | | | |
| Aggravating<br>Factors | | | |
| Alleviating<br>Factors | | | |
| Associated<br>Symptoms | | | |
| Previous Treatments | | | |

# EXAMINATION

## GENERAL APPEARANCE

| | | | |
|---|---|---|---|
| **Head and Neck (Development, Symmetry)** | Not assessed | WNL | Remarkable findings: |
| **Overall Body** | Not assessed | WNL | Remarkable findings: |

## CRANIAL NERVE SCREENING

| | | | |
|---|---|---|---|
| **(II) Gross vision** | Not assessed | WNL | Remarkable findings: |
| **(III, IV, VI) Extra-ocular muscles / pupils** | Not assessed | WNL | Remarkable findings: |
| **(V) Sensory** | Not assessed | WNL | Remarkable findings: |
| **(V) Motor function / symmetry** | Not assessed | WNL | Remarkable findings: |
| **(VII) Motor function / symmetry** | Not assessed | WNL | Remarkable findings: |
| **(VIII) Gross hearing** | Not assessed | WNL | Remarkable findings: |
| **EAC / Tympanic Membrane** | Not assessed | WNL | Remarkable findings: |
| **(IX, X) Palatal elevation / gag reflex** | Not assessed | WNL | Remarkable findings: |
| **(XI) Shoulder shrug / head turns** | Not assessed | WNL | Remarkable findings: |
| **(XII) Tongue Protrusion** | Not assessed | WNL | Remarkable findings: |

## BALANCE COORDINATION

| | | | |
|---|---|---|---|
| **Gait & Gross Motor Movement** | Not assessed | WNL | Remarkable findings: |
| **Finger to Nose Movement** | Not assessed | WNL | Remarkable findings: |

## CERVICAL EXAMINATION

| | | | | | |
|---|---|---|---|---|---|
| **Head/ Neck Position** | Not assessed | WNL | Forward head/body | Lateral tilt//turn ( R L ) | |
| **Rotation Right** | Not assessed | WNL | Restricted | Neck Pain | CC pain ( R / L / B ) |
| **Rotation Left** | Not assessed | WNL | Restricted | Neck Pain | CC pain ( R / L / B ) |
| **Tilt Right** | Not assessed | WNL | Restricted | Neck Pain | CC pain ( R / L / B ) |
| **Tilt Left** | Not assessed | WNL | Restricted | Neck Pain | CC pain ( R / L / B ) |
| **Flexion (forward)** | Not assessed | WNL | Restricted | Neck Pain | CC pain ( R / L / B ) |
| **Extension (backward)** | Not assessed | WNL | Restricted | Neck Pain | CC pain ( R / L / B ) |

# PALPATION EXAM

**(X) = painful/tender (T) = palpable knot/trigger point (*) = reproduced familiar pain (→) = pain referral**

| | **Right** | **Left** |
|---|---|---|
| **Trapezius** | | |
| **SCM** | | |
| **Splenius capitis** | | |
| **Occipitalis** | | |
| **Paracervical** | | |
| **C Spine** | | |
| | | |
| **Masseter** | | |
| **Temporalis** | | |
| **Frontalis** | | |
| **TMJ (static)** | | |
| **TMJ (dynamic)** | | |
| **Lateral pterygoid (provocation)** | | |
| **Temporal tendon** | | |
| **Anterior digastric** | | |
| **Posterior digastric** | | |

**Additional palpation exam findings**: ______________________________

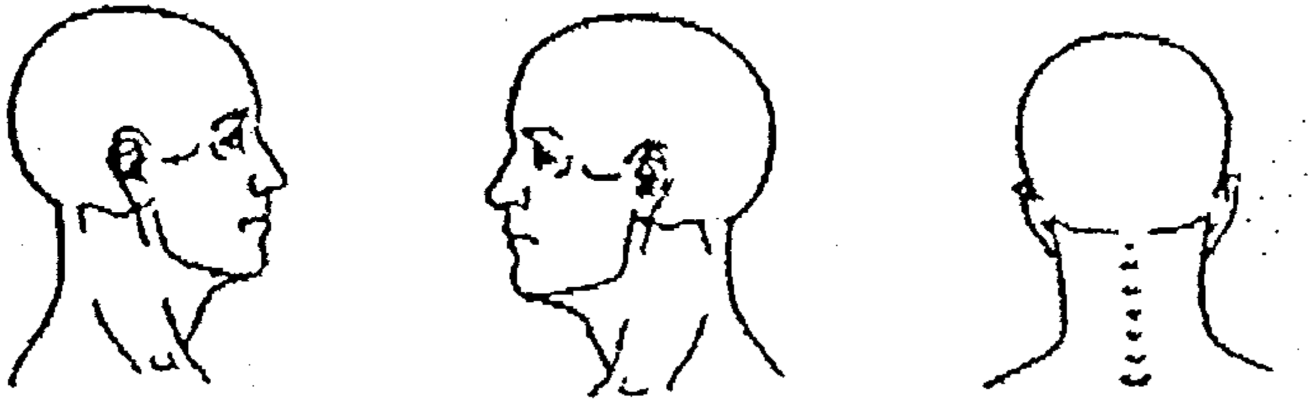

# MANDIBULAR RANGE OF MOTION

**Pain on maximum opening**: Y N R L

**Maximum comfortable opening**: ______mm

**Maximum unassisted opening:** N/A_________mm **Pain on** R L

**Maximum assisted opening:** N/A_________mm **Pain on** R L **Overbite**:

________________% **Overjet**:________mm

**Protrusive movement**: _______mm **Painful**: Y N R L

**R lateral movement**: _______mm **Painful**: Y N R L

**L lateral movement**: _______mm **Painful**: Y N R L

**TMJ locking:** Not observed Open lock ( R / L / B ) Closed lock ( R / L / B )

**Deviation**: Corrected Uncorrected R L

**Deviation**

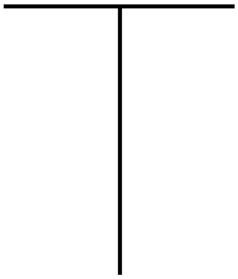

# TMJ SOUNDS

**Right**: None Opening Reciprocal Intermittent Painful Crepitus Eminence Click Painless **Left**:

None Opening Reciprocal Intermittent Painful Crepitus Eminence Click Painless **Is sound**

**Eliminated with protrusion?** Y N

# INTRA-ORAL EXAMINATION

**Soft Tissue / Oral Cancer Screening**: WNL Remarkable findings: ______________________________

**Modified Mallampati / Tonsil Grade**: WNL Remarkable findings: ______________________________

**Dentition / Periodontium**: Grossly WNL Remarkable findings: ______________________________

**Tooth Wear**: Physiologic Moderate Severe Notes: ________________________

**Occlusion**: Class I Class II Div 1 2 Class III

Open Bite? Yes No ____________________________________________________

Cross Bites? Yes No ____________________________________________________

**Caries/Restorations:** ____________________________________________________________

**Percussion:**______________________________________________________________________

**Palpation:** ______________________________________________________________________

**Cold:**___________________________________________________________________________

**Tooth Slooth:** ___________________________________________________________________

**Other notable intra-oral findings**: N/A Notes:____________________________________________

This work was prepared as part of official military duties. This document reflects the views of the authors and does not reflect the policies of the U.S. Government, Department of Defense, U.S. Navy, or Uniformed Services University of the Health Sciences.
