# Supplementary material for: Enhancing Temporomandibular Disorders Education for Initial Care Clinicians Through Interprofessional Education
Source: MedEdPORTAL. 2024 Nov 19;20:11467. doi: 10.15766/mep_2374-8265.11467 (PMC11575917; doi:10.15766/mep_2374-8265.11467)
Supplement: Supplementary file 1 — Facilitator Guide.docxLearner Guide and Clinical Tools.pdfModule 1 - TMD Pathophysiology.pptxModule 2 - TMD Assessment.pptxModule 3 - TMD Diagnosis.pptxModule 4 - TMD Management.pptxSample Patient Education Tools.pptx [file mep_2374-8265.11467-s001.zip › E. Module 3 - TMD Diagnosis.pptx]

## Slide 1
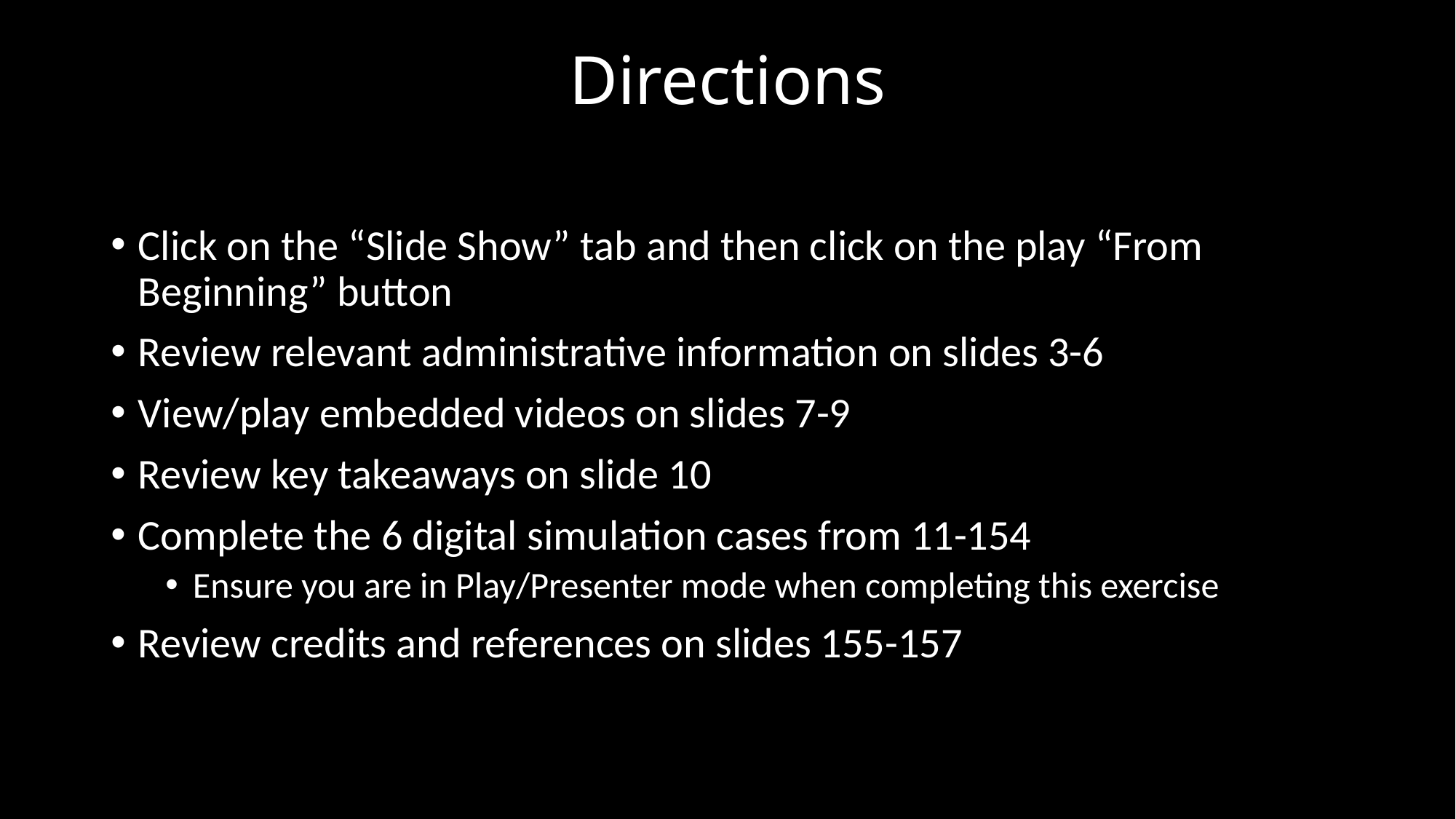

# Directions
Click on the “Slide Show” tab and then click on the play “From Beginning” button
Review relevant administrative information on slides 3-6
View/play embedded videos on slides 7-9
Review key takeaways on slide 10
Complete the 6 digital simulation cases from 11-154
Ensure you are in Play/Presenter mode when completing this exercise
Review credits and references on slides 155-157

## Slide 2
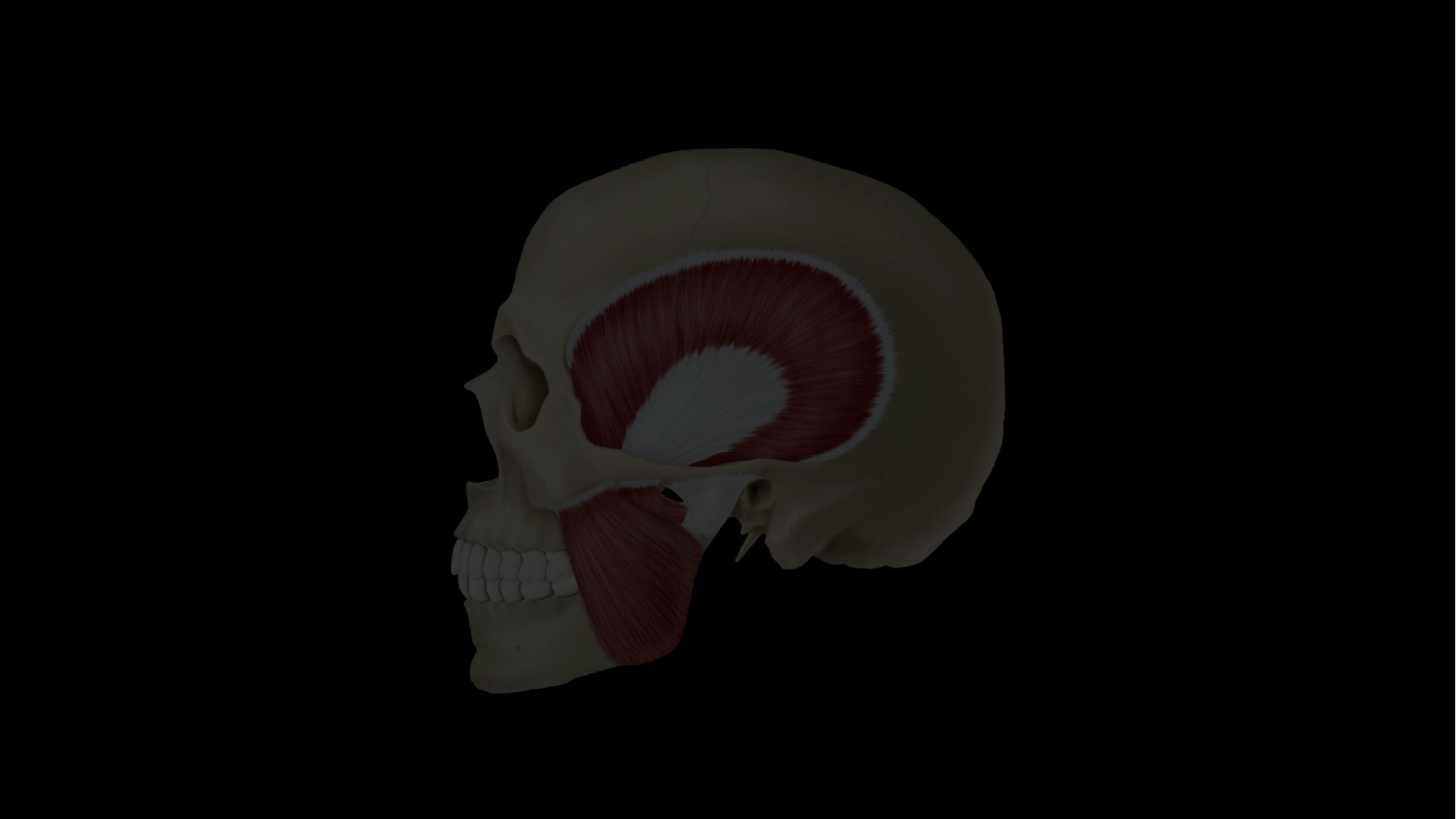

## Slide 3
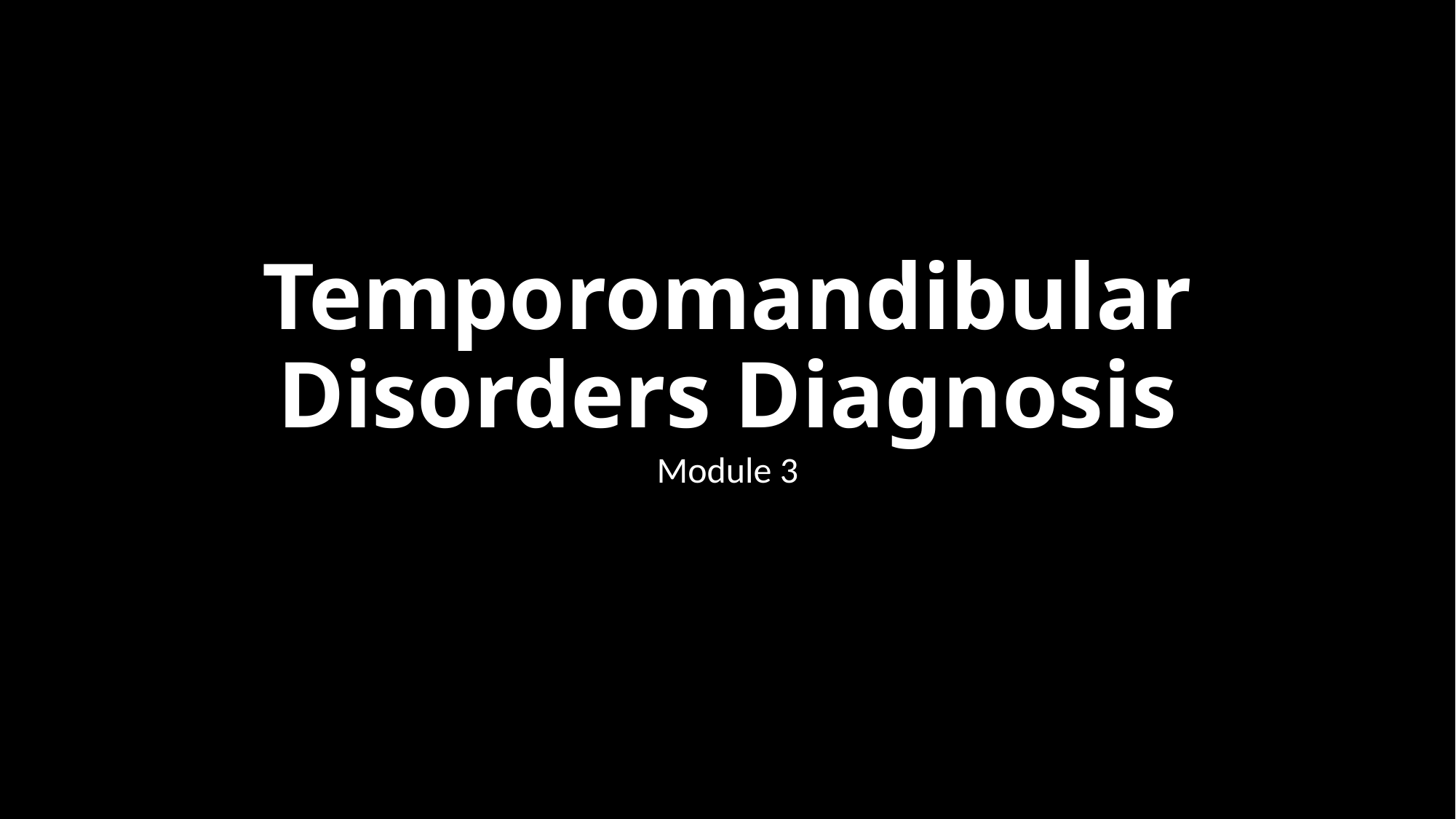

# Temporomandibular Disorders Diagnosis
Module 3

## Slide 4
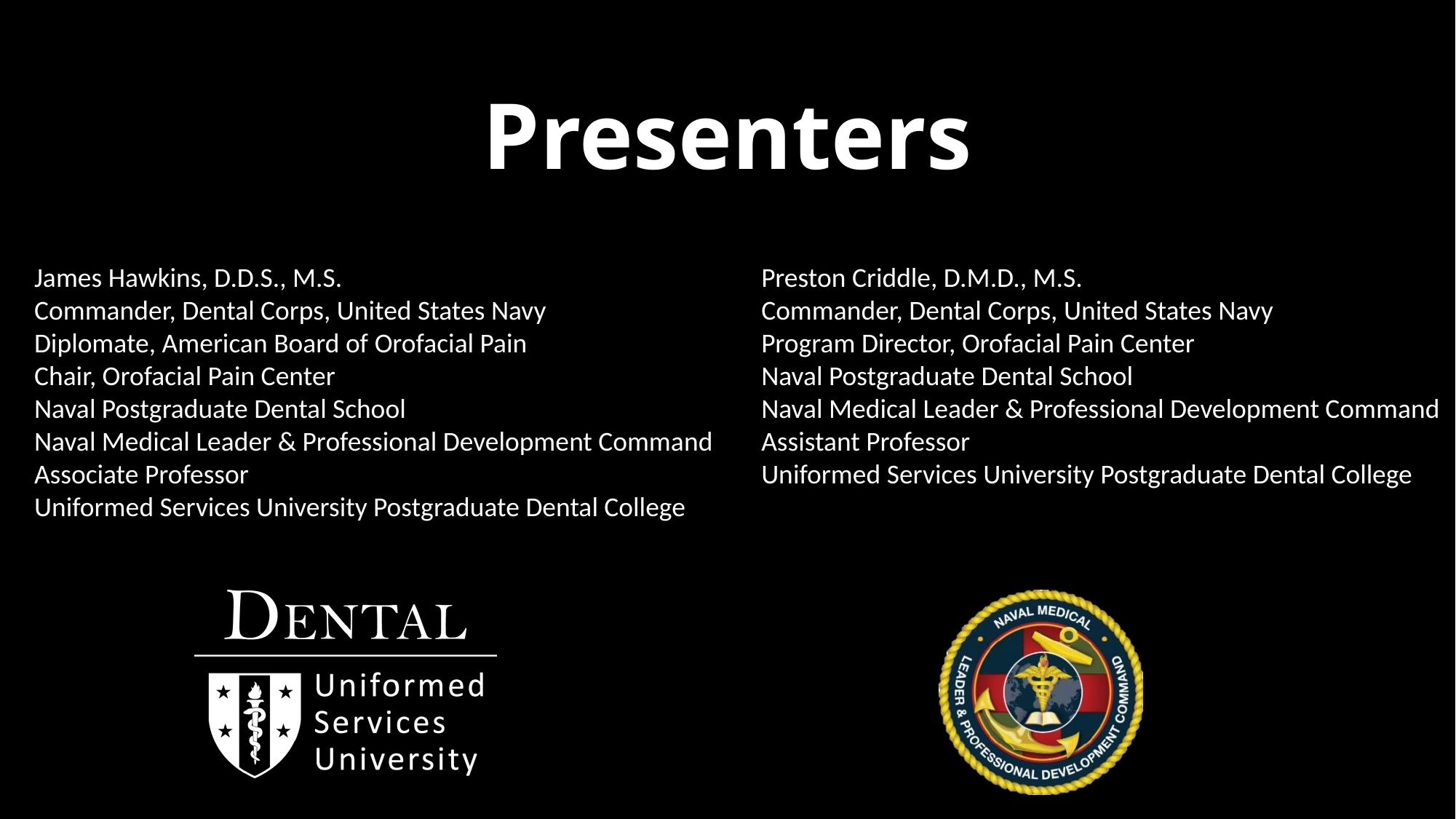

# Presenters
James Hawkins, D.D.S., M.S.
Commander, Dental Corps, United States Navy
Diplomate, American Board of Orofacial Pain
Chair, Orofacial Pain Center
Naval Postgraduate Dental School
Naval Medical Leader & Professional Development Command
Associate Professor
Uniformed Services University Postgraduate Dental College
Preston Criddle, D.M.D., M.S.
Commander, Dental Corps, United States Navy
Program Director, Orofacial Pain Center
Naval Postgraduate Dental School
Naval Medical Leader & Professional Development Command
Assistant Professor
Uniformed Services University Postgraduate Dental College

## Slide 5
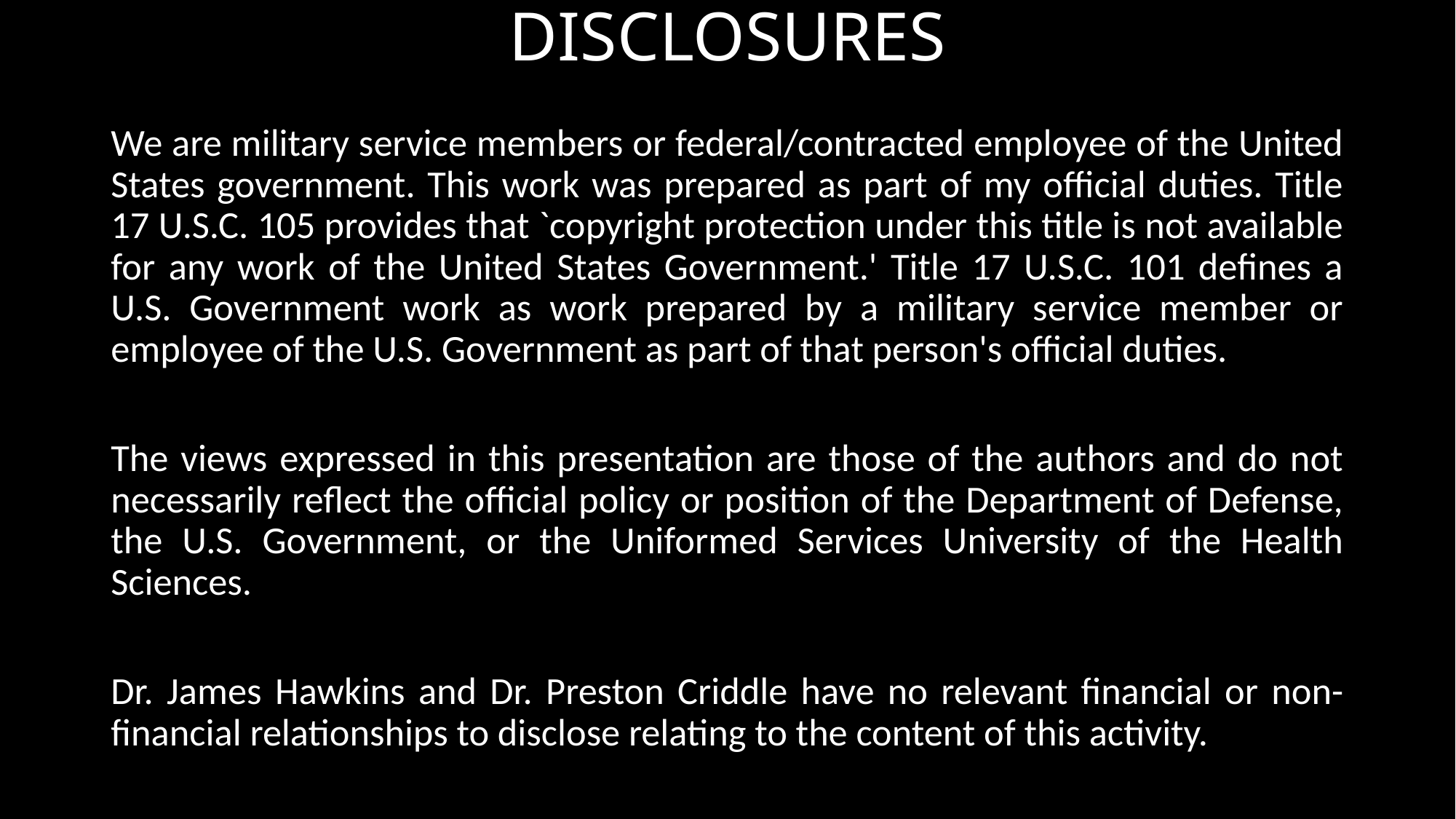

# DISCLOSURES
We are military service members or federal/contracted employee of the United States government. This work was prepared as part of my official duties. Title 17 U.S.C. 105 provides that `copyright protection under this title is not available for any work of the United States Government.' Title 17 U.S.C. 101 defines a U.S. Government work as work prepared by a military service member or employee of the U.S. Government as part of that person's official duties.
The views expressed in this presentation are those of the authors and do not necessarily reflect the official policy or position of the Department of Defense, the U.S. Government, or the Uniformed Services University of the Health Sciences.
Dr. James Hawkins and Dr. Preston Criddle have no relevant financial or non-financial relationships to disclose relating to the content of this activity.

## Slide 6
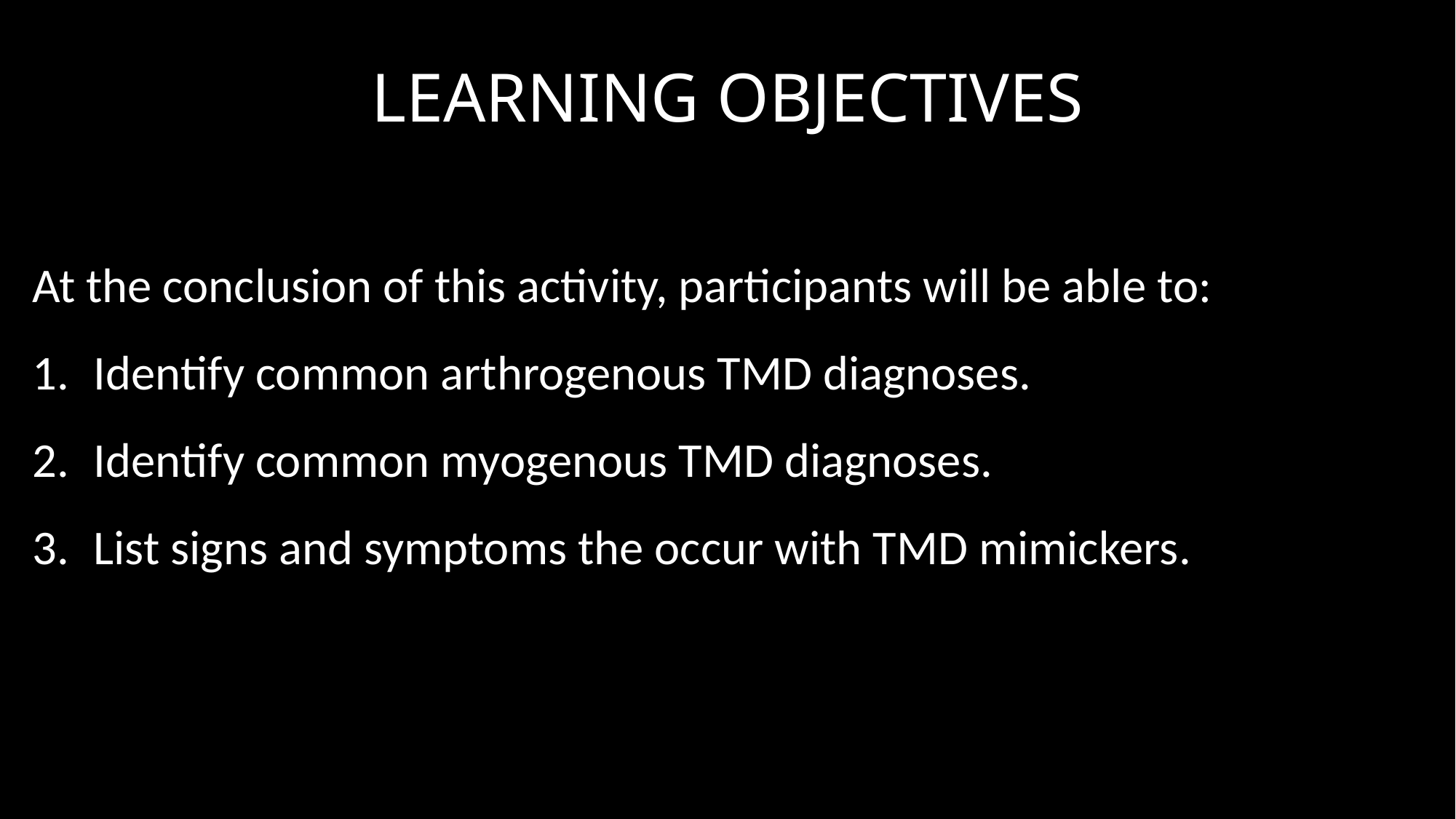

# LEARNING OBJECTIVES
At the conclusion of this activity, participants will be able to:
Identify common arthrogenous TMD diagnoses.
Identify common myogenous TMD diagnoses.
List signs and symptoms the occur with TMD mimickers.

## Slide 7
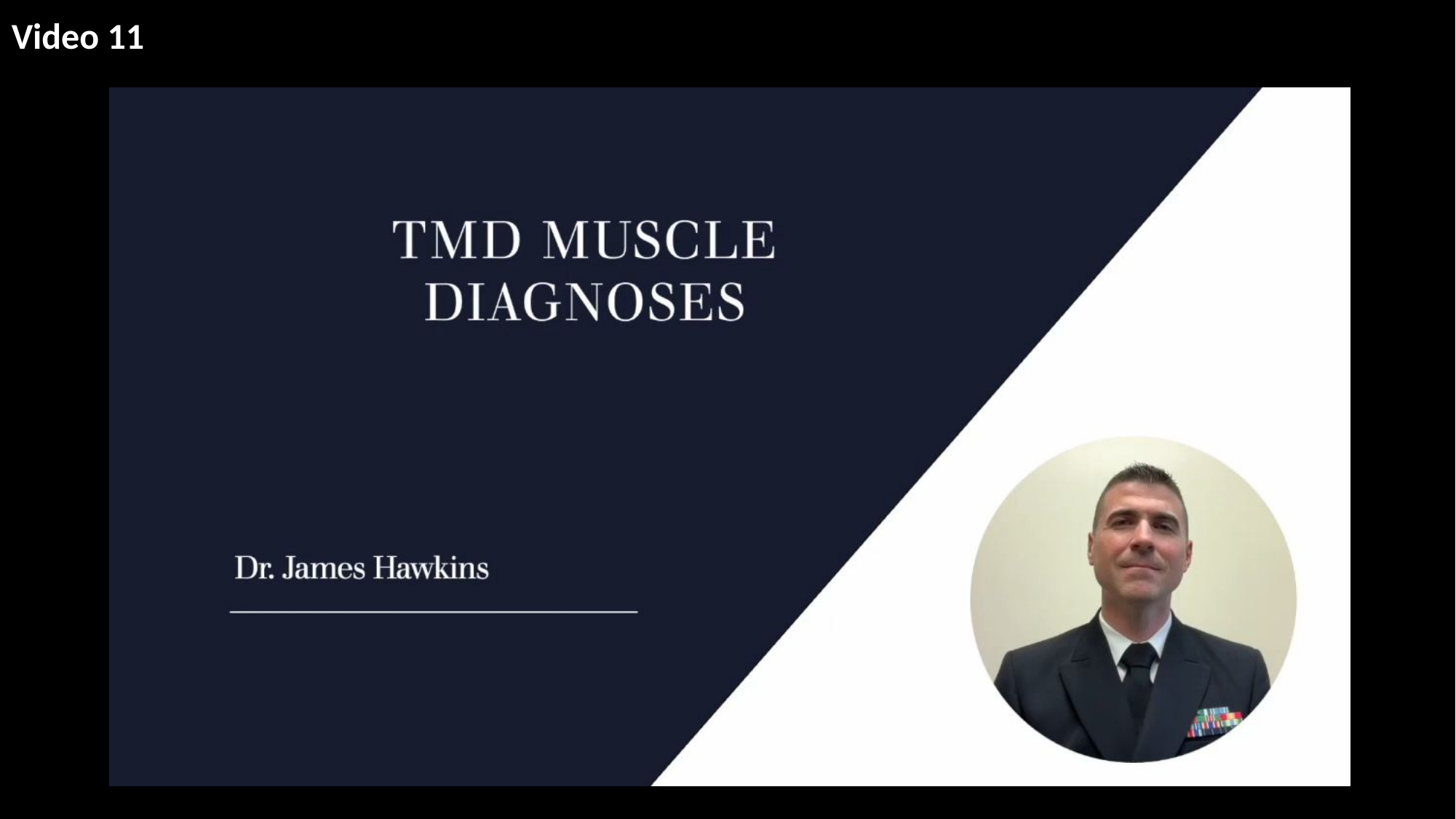

Video 11

## Slide 8
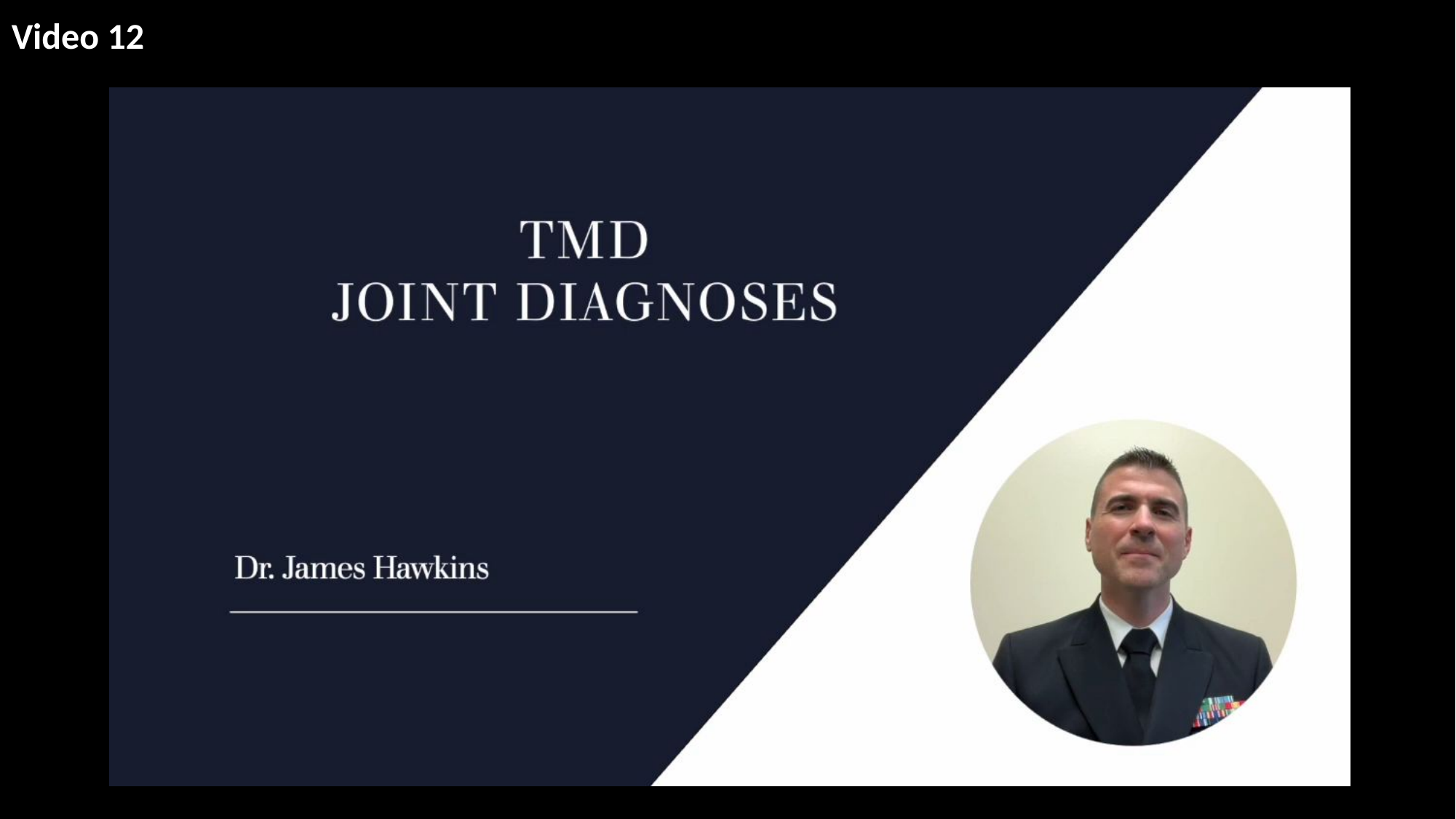

Video 12

## Slide 9
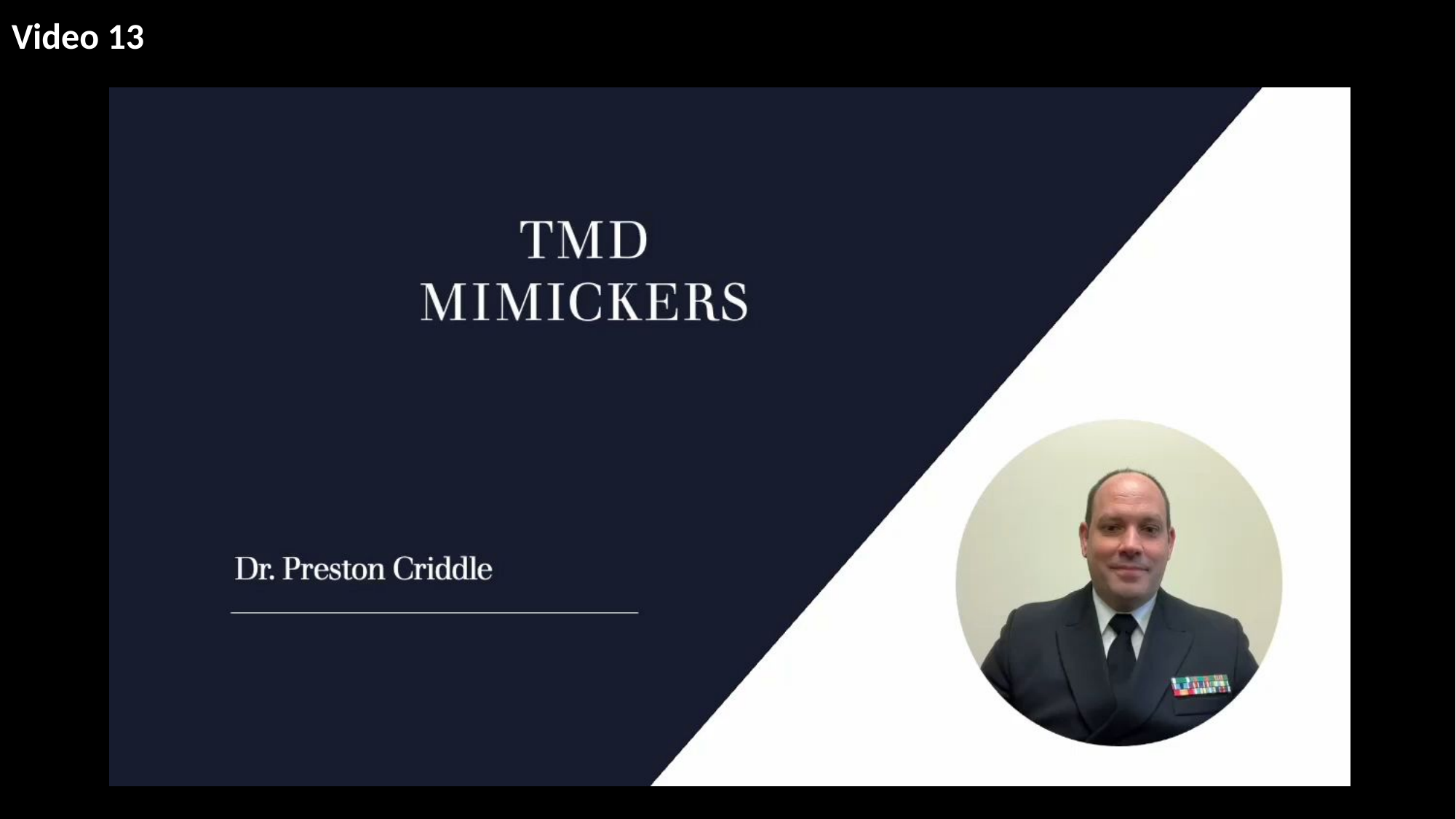

Video 13

## Slide 10
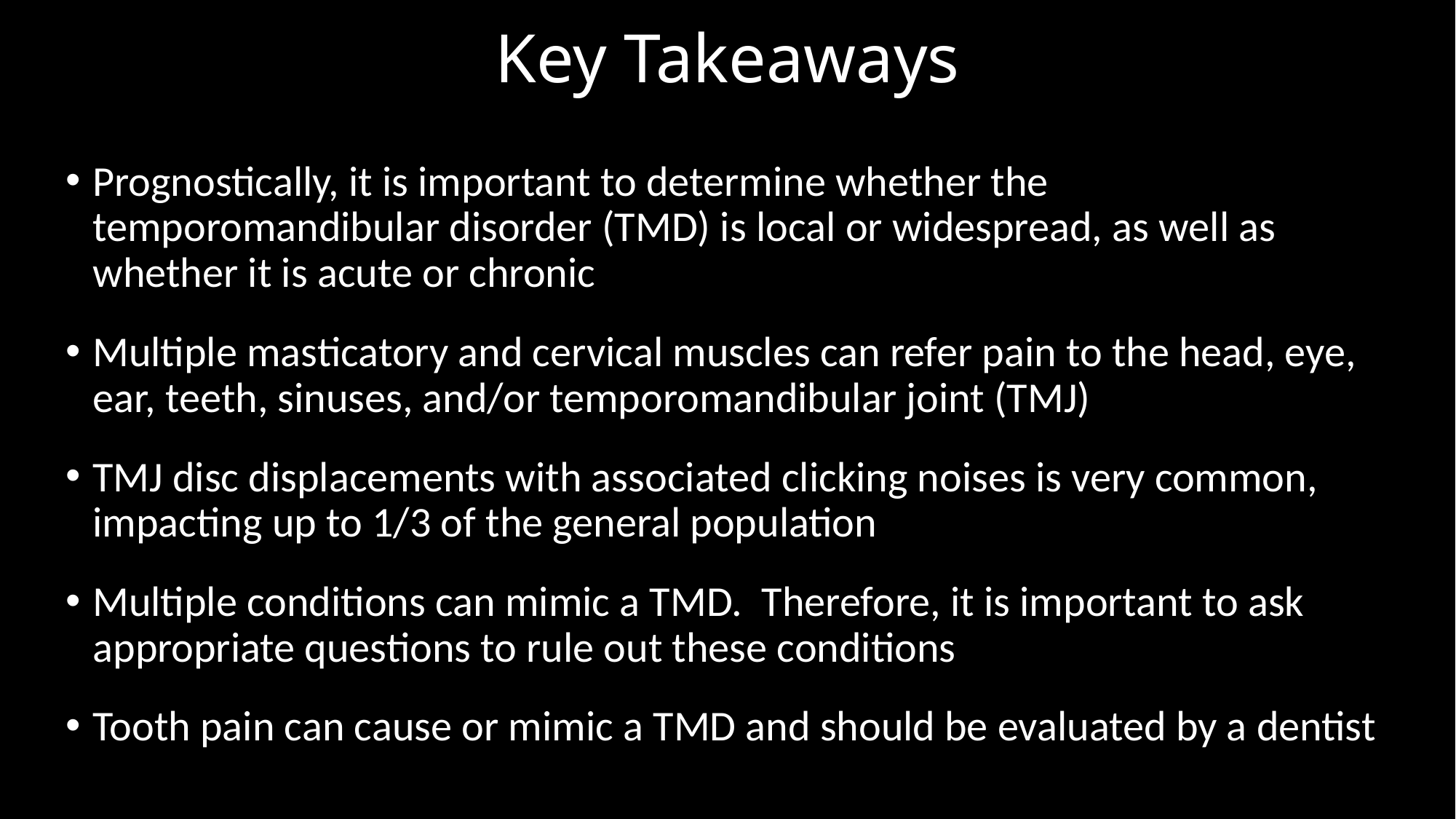

# Key Takeaways
Prognostically, it is important to determine whether the temporomandibular disorder (TMD) is local or widespread, as well as whether it is acute or chronic
Multiple masticatory and cervical muscles can refer pain to the head, eye, ear, teeth, sinuses, and/or temporomandibular joint (TMJ)
TMJ disc displacements with associated clicking noises is very common, impacting up to 1/3 of the general population
Multiple conditions can mimic a TMD. Therefore, it is important to ask appropriate questions to rule out these conditions
Tooth pain can cause or mimic a TMD and should be evaluated by a dentist

## Slide 11
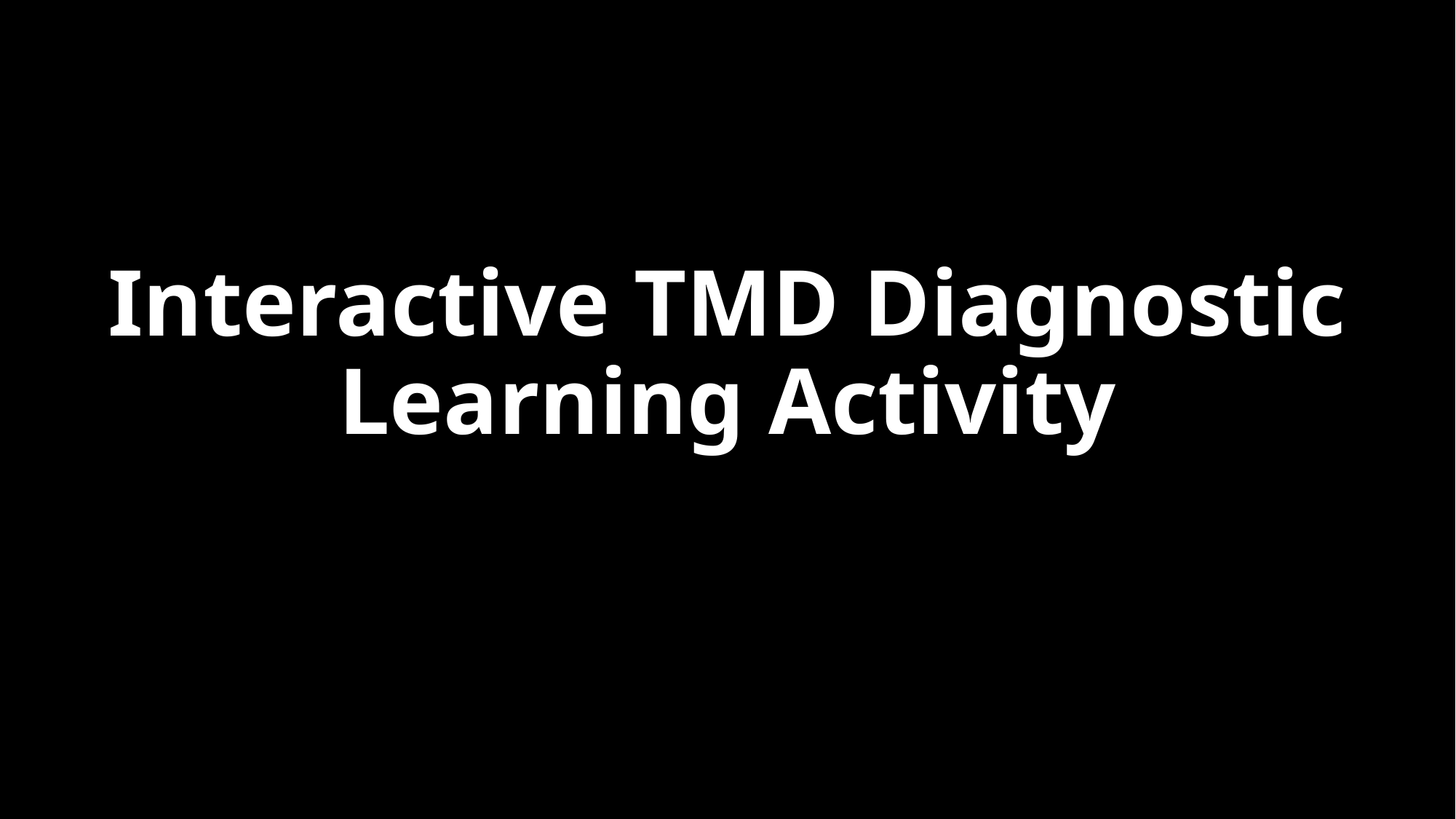

# Interactive TMD Diagnostic Learning Activity

## Slide 12
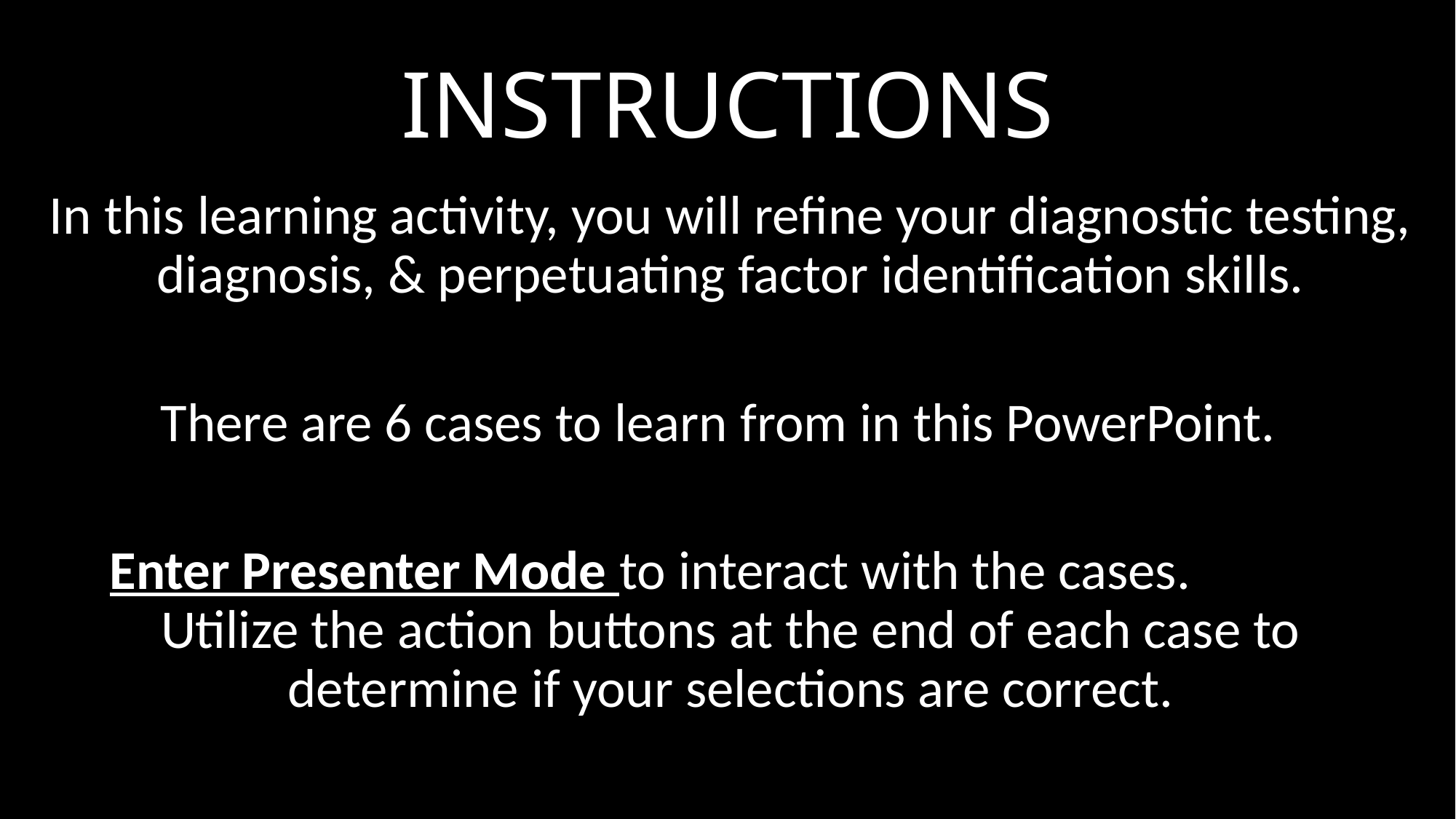

# INSTRUCTIONS
In this learning activity, you will refine your diagnostic testing, diagnosis, & perpetuating factor identification skills.
There are 6 cases to learn from in this PowerPoint.
Enter Presenter Mode to interact with the cases. Utilize the action buttons at the end of each case to determine if your selections are correct.

## Slide 13
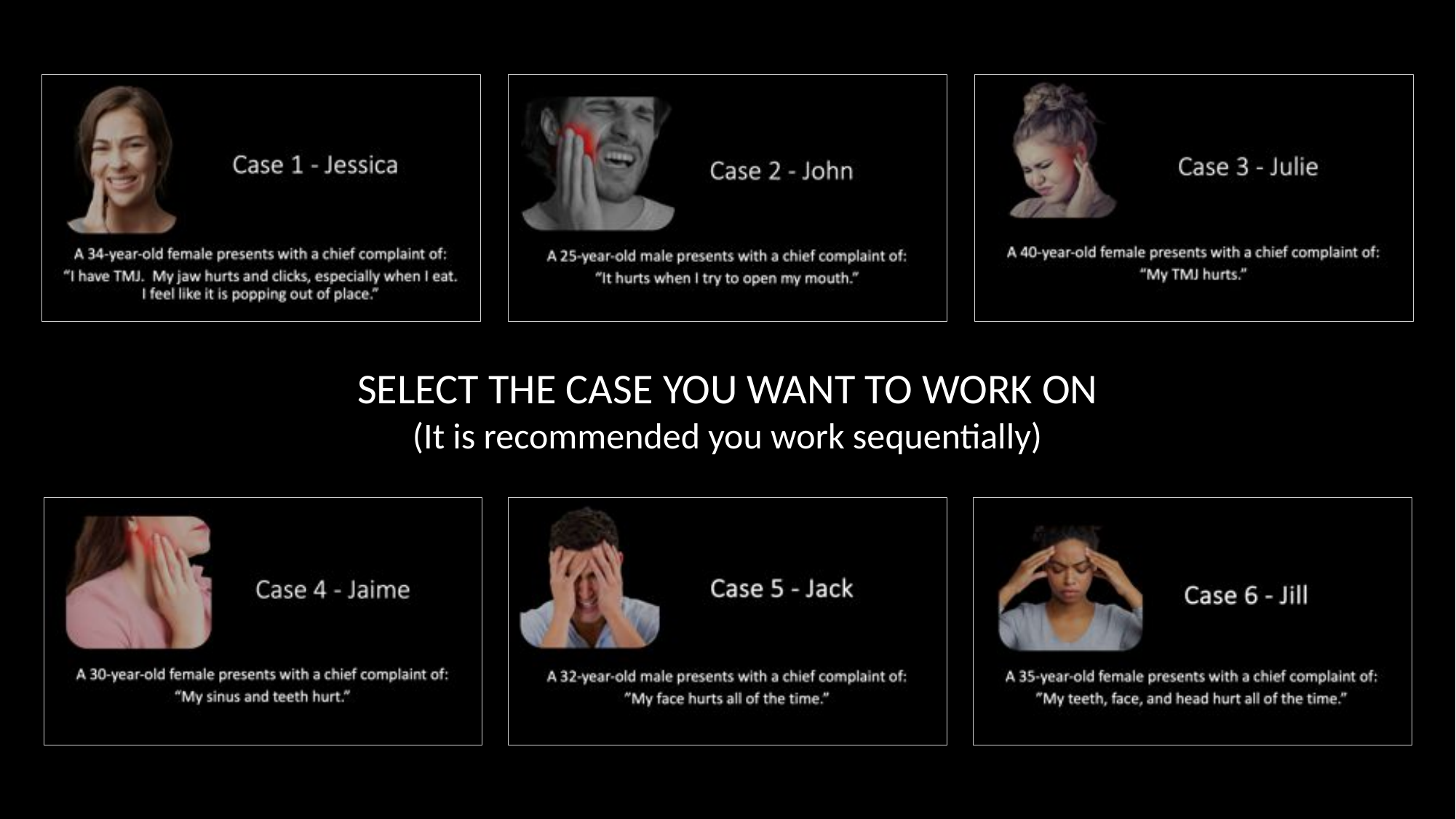

SELECT THE CASE YOU WANT TO WORK ON
(It is recommended you work sequentially)

## Slide 14
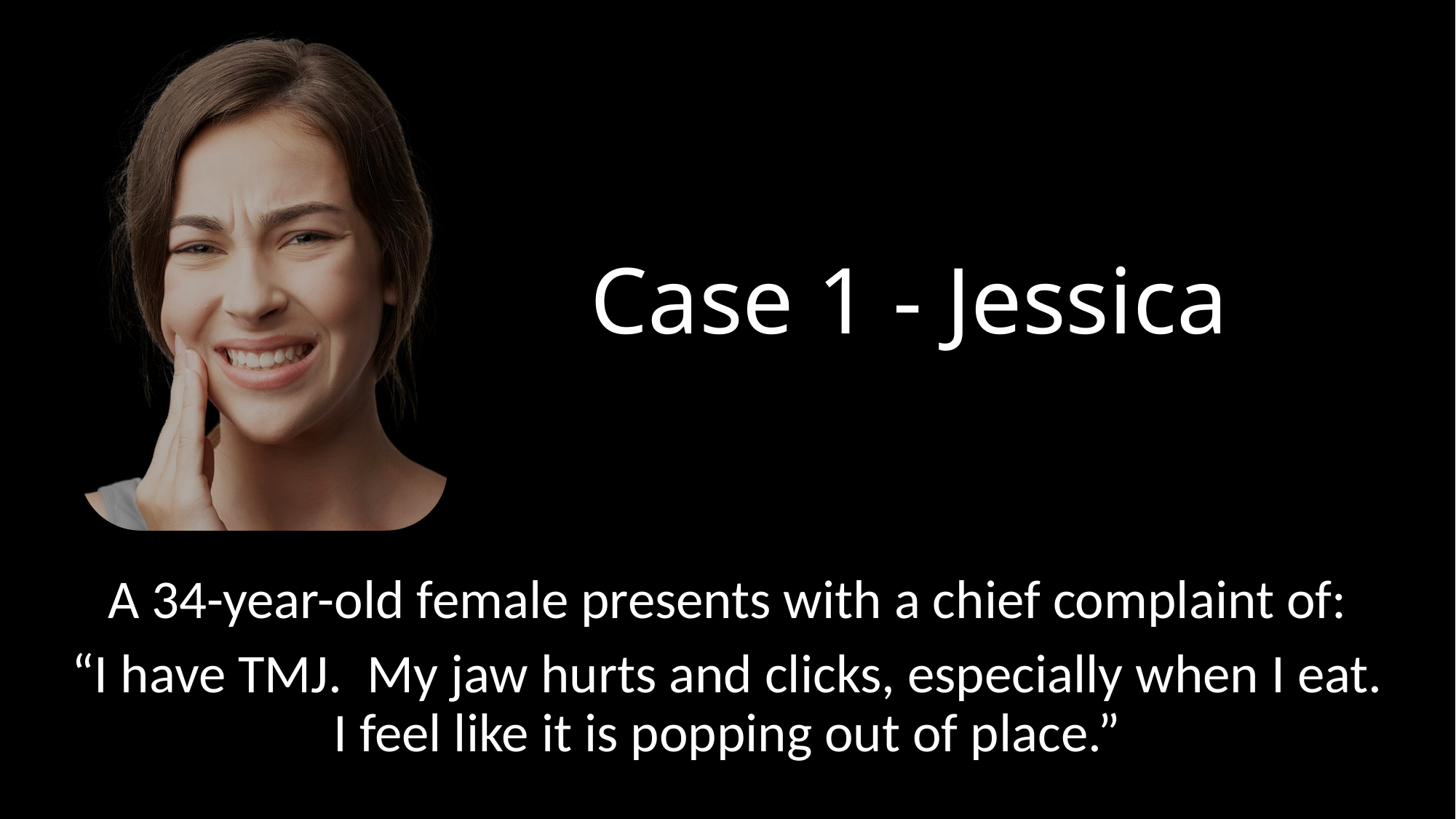

# Case 1 - Jessica
A 34-year-old female presents with a chief complaint of:
“I have TMJ. My jaw hurts and clicks, especially when I eat. I feel like it is popping out of place.”

## Slide 15
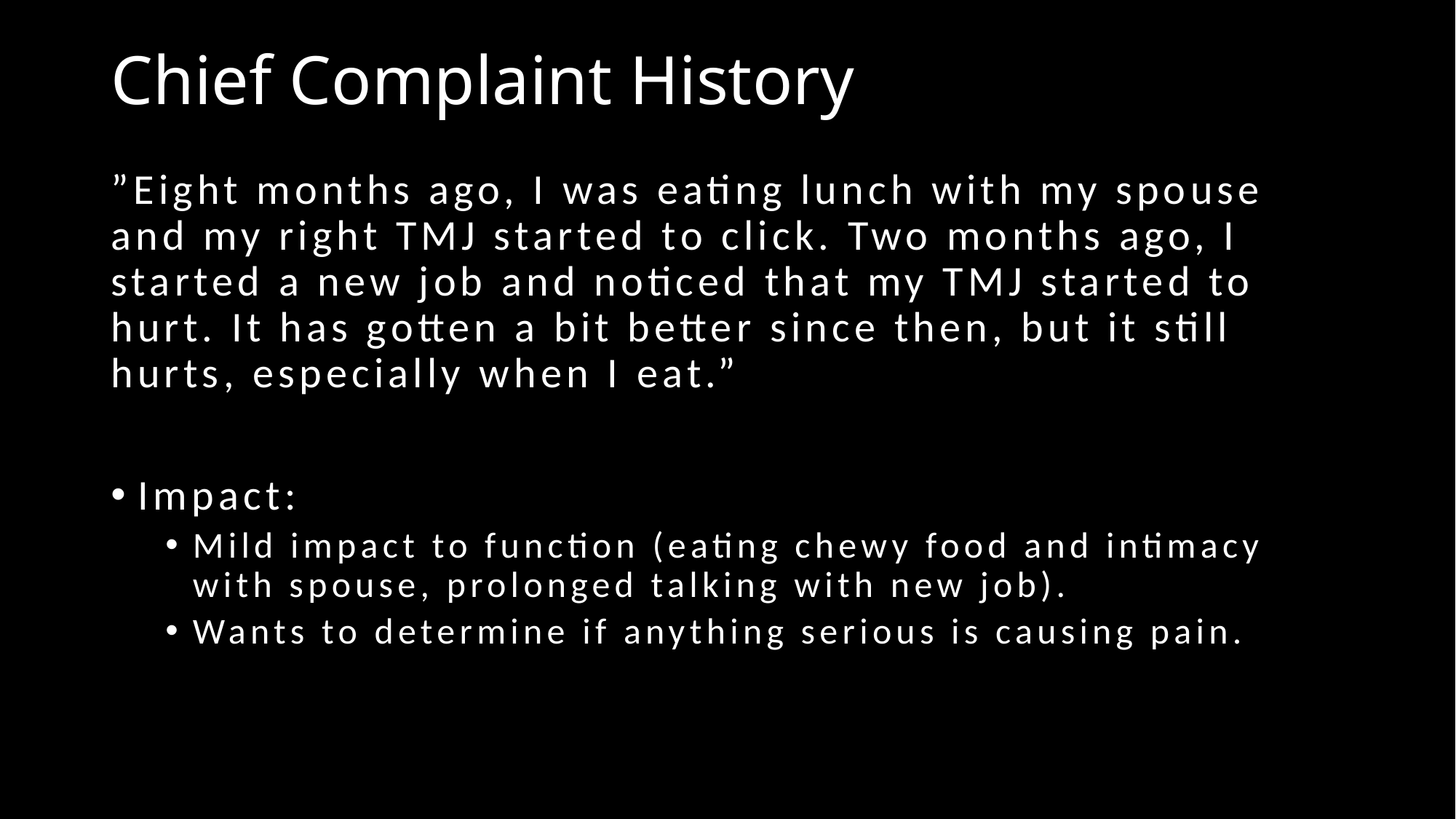

# Chief Complaint History
”Eight months ago, I was eating lunch with my spouse and my right TMJ started to click. Two months ago, I started a new job and noticed that my TMJ started to hurt. It has gotten a bit better since then, but it still hurts, especially when I eat.”
Impact:
Mild impact to function (eating chewy food and intimacy with spouse, prolonged talking with new job).
Wants to determine if anything serious is causing pain.

## Slide 16
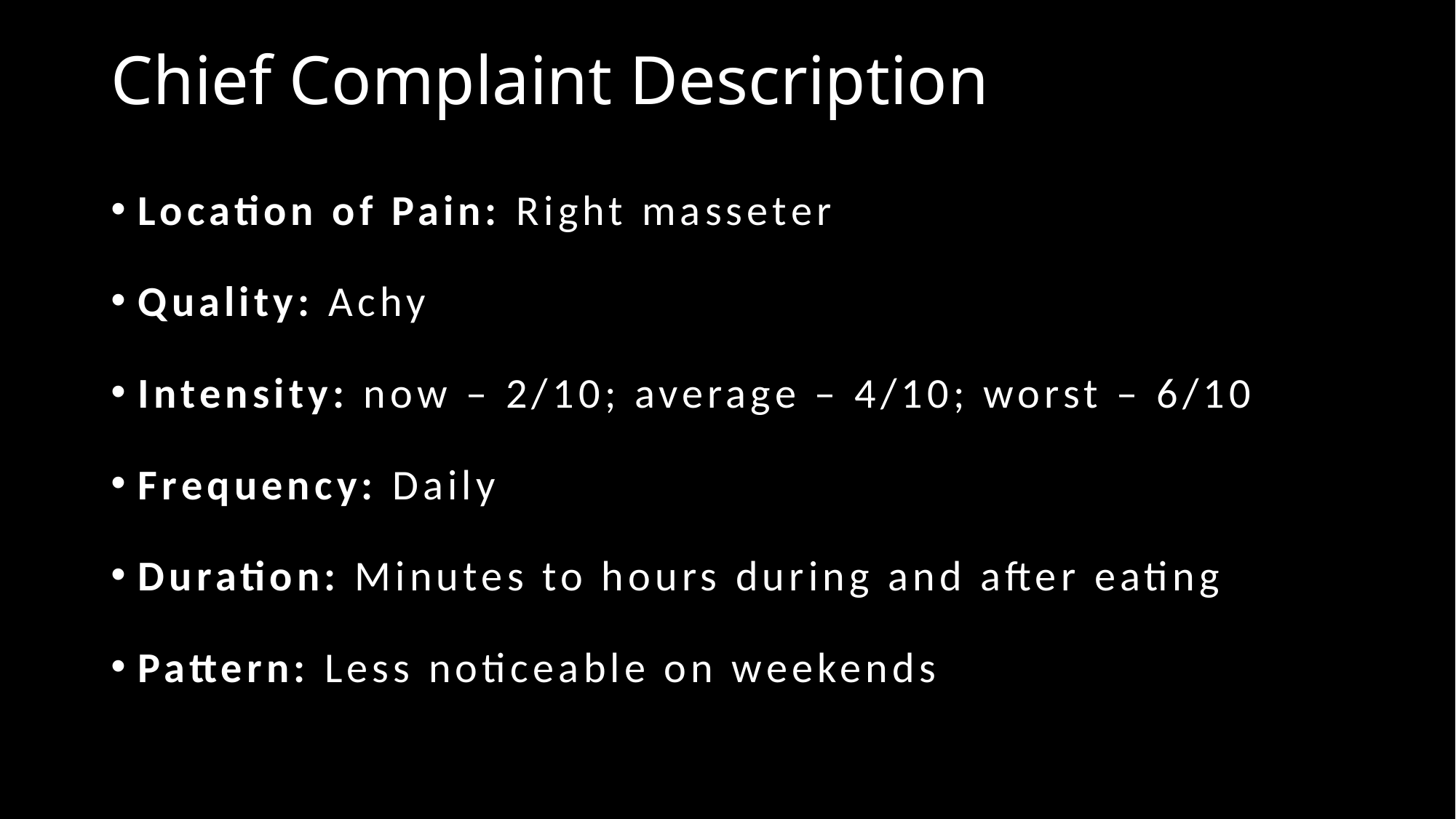

# Chief Complaint Description
Location of Pain: Right masseter
Quality: Achy
Intensity: now – 2/10; average – 4/10; worst – 6/10
Frequency: Daily
Duration: Minutes to hours during and after eating
Pattern: Less noticeable on weekends

## Slide 17
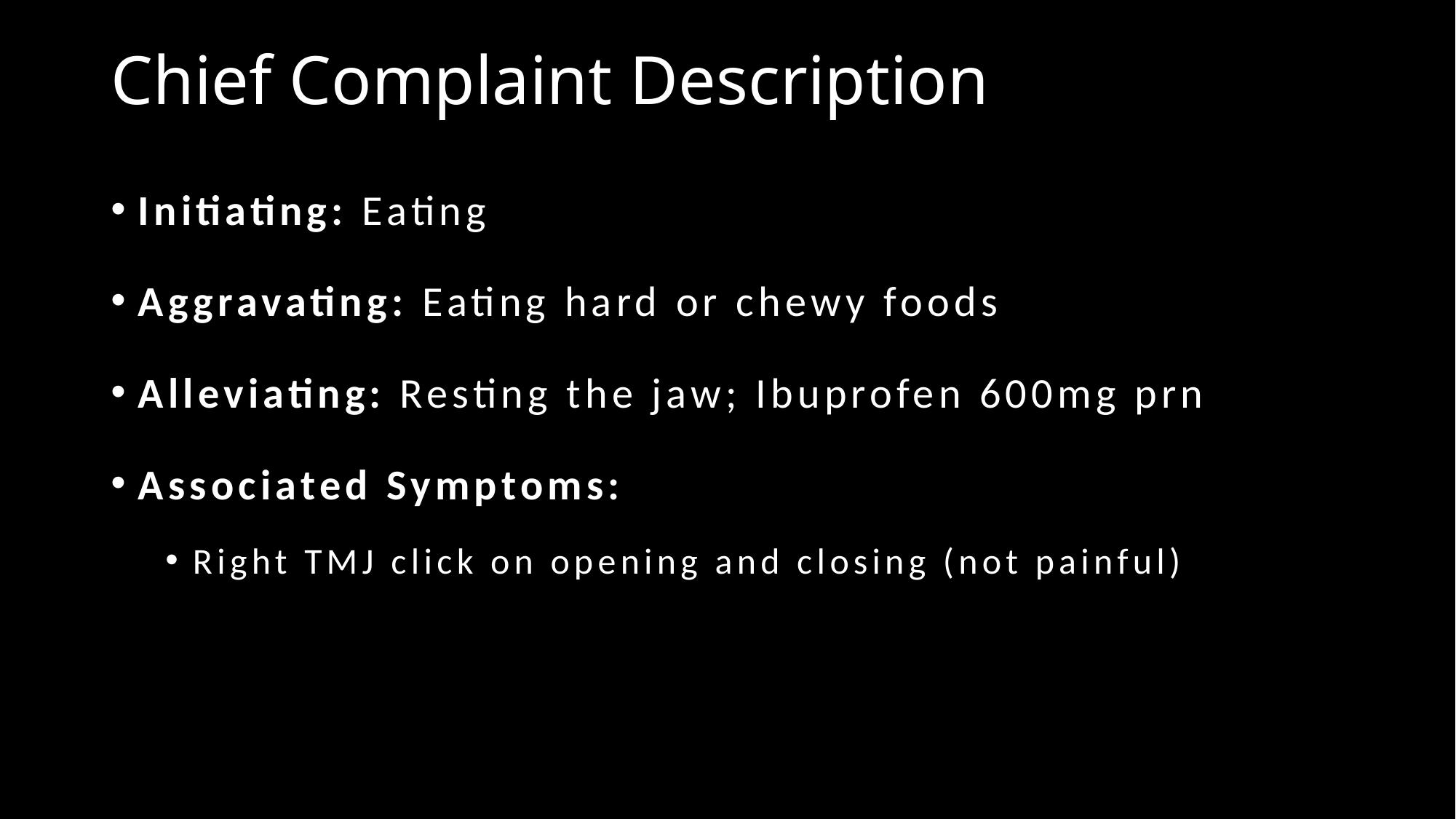

# Chief Complaint Description
Initiating: Eating
Aggravating: Eating hard or chewy foods
Alleviating: Resting the jaw; Ibuprofen 600mg prn
Associated Symptoms:
Right TMJ click on opening and closing (not painful)

## Slide 18
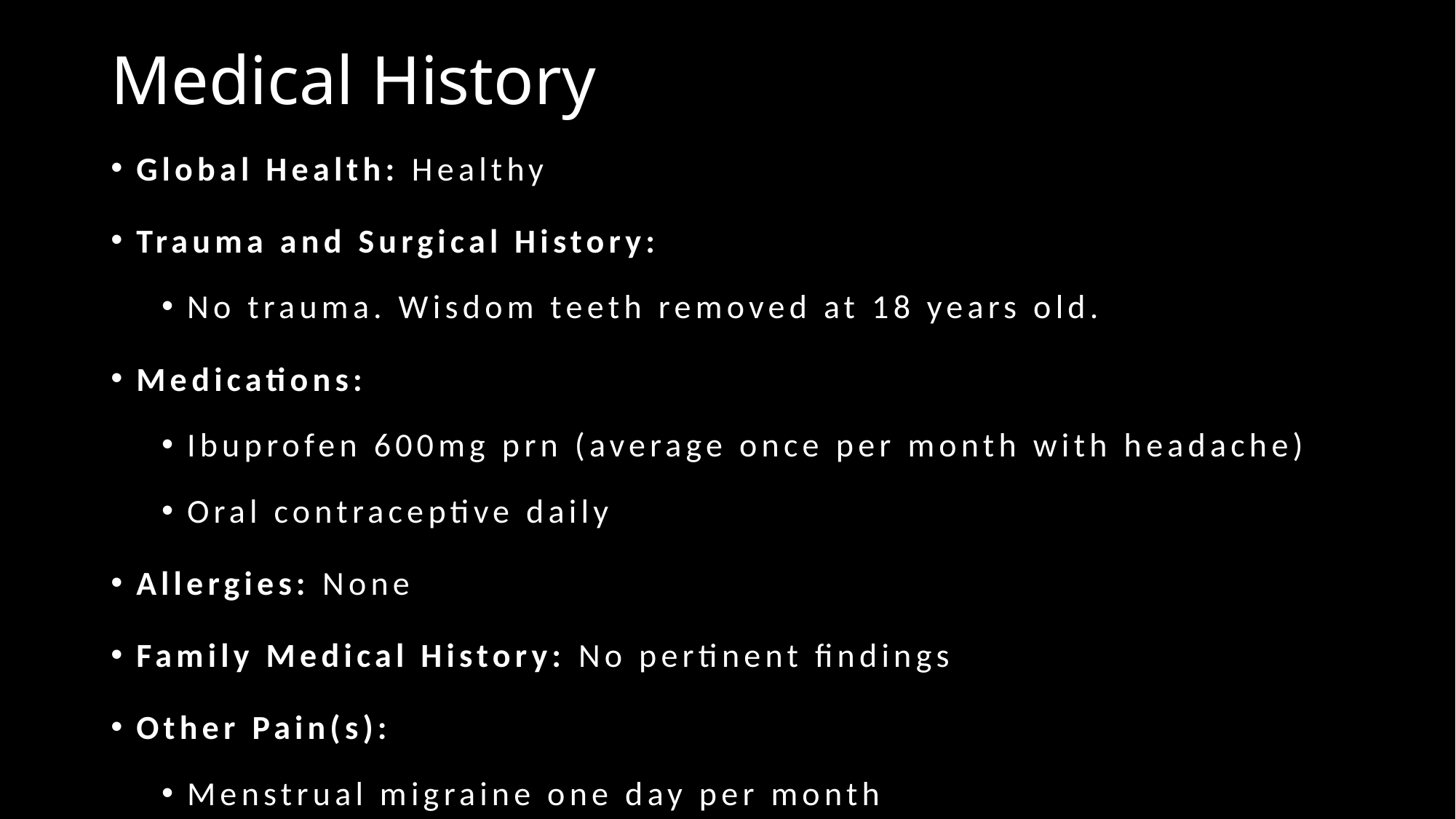

# Medical History
Global Health: Healthy
Trauma and Surgical History:
No trauma. Wisdom teeth removed at 18 years old.
Medications:
Ibuprofen 600mg prn (average once per month with headache)
Oral contraceptive daily
Allergies: None
Family Medical History: No pertinent findings
Other Pain(s):
Menstrual migraine one day per month

## Slide 19
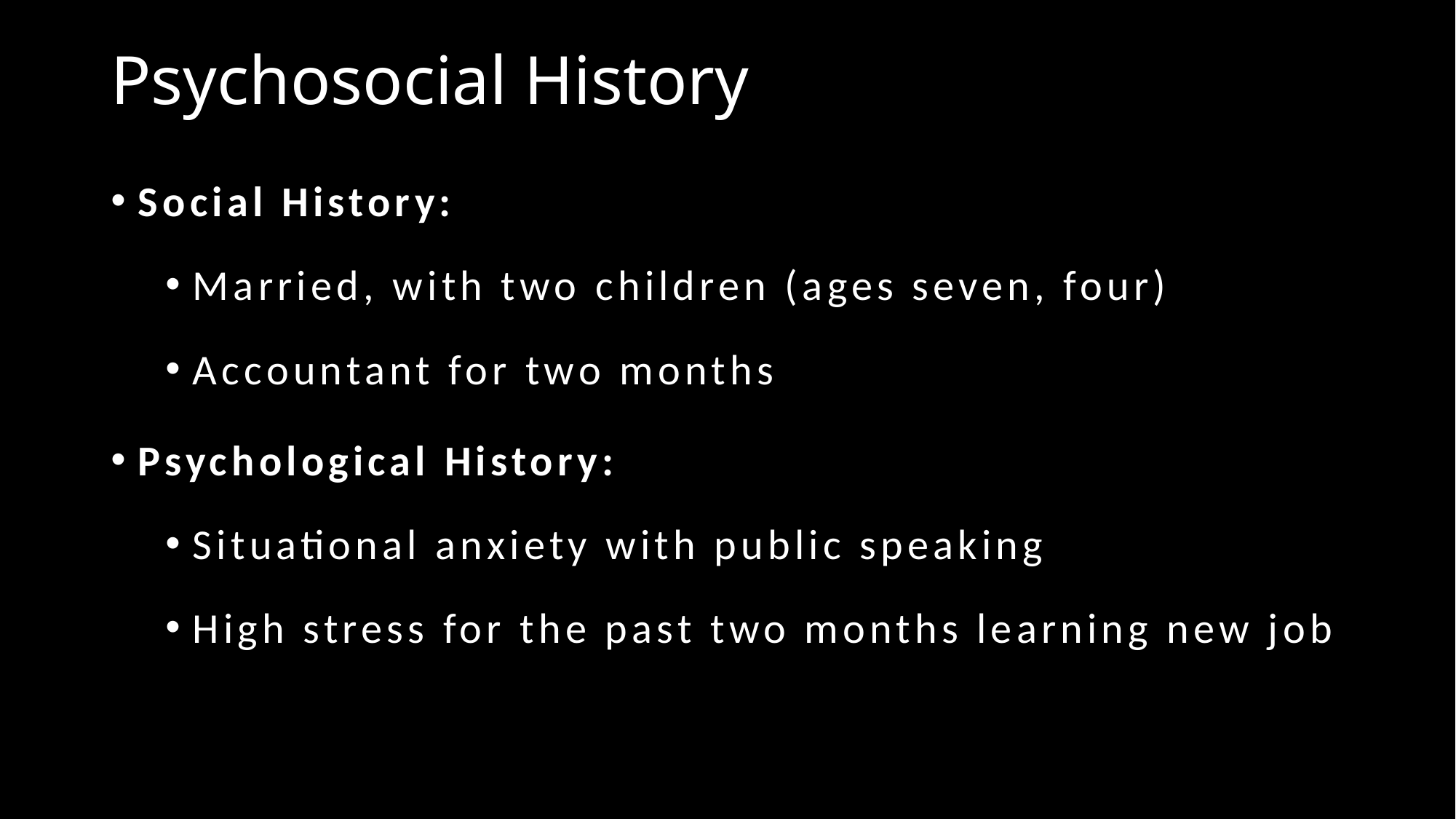

# Psychosocial History
Social History:
Married, with two children (ages seven, four)
Accountant for two months
Psychological History:
Situational anxiety with public speaking
High stress for the past two months learning new job

## Slide 20
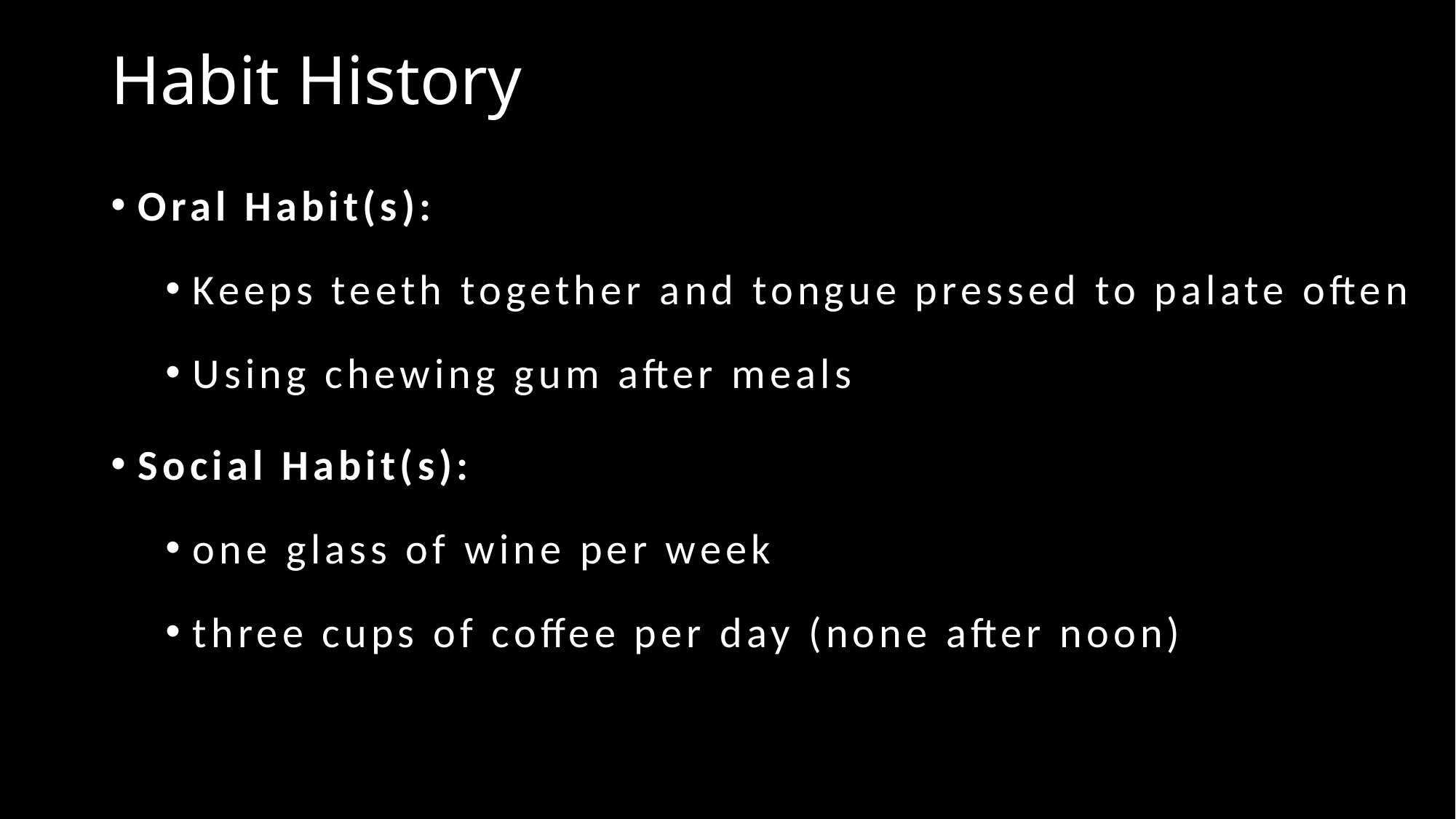

# Habit History
Oral Habit(s):
Keeps teeth together and tongue pressed to palate often
Using chewing gum after meals
Social Habit(s):
one glass of wine per week
three cups of coffee per day (none after noon)

## Slide 21
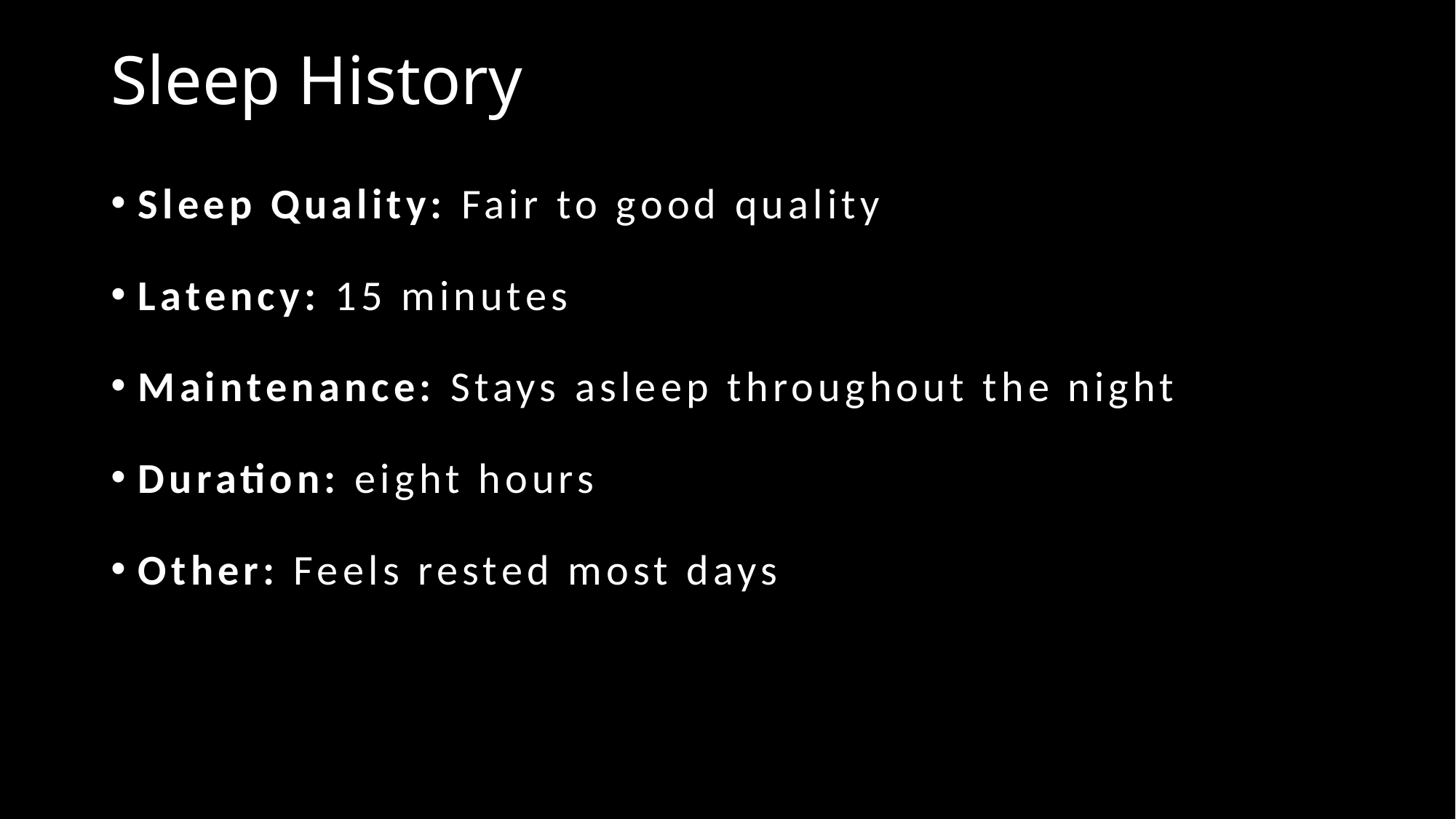

# Sleep History
Sleep Quality: Fair to good quality
Latency: 15 minutes
Maintenance: Stays asleep throughout the night
Duration: eight hours
Other: Feels rested most days

## Slide 22
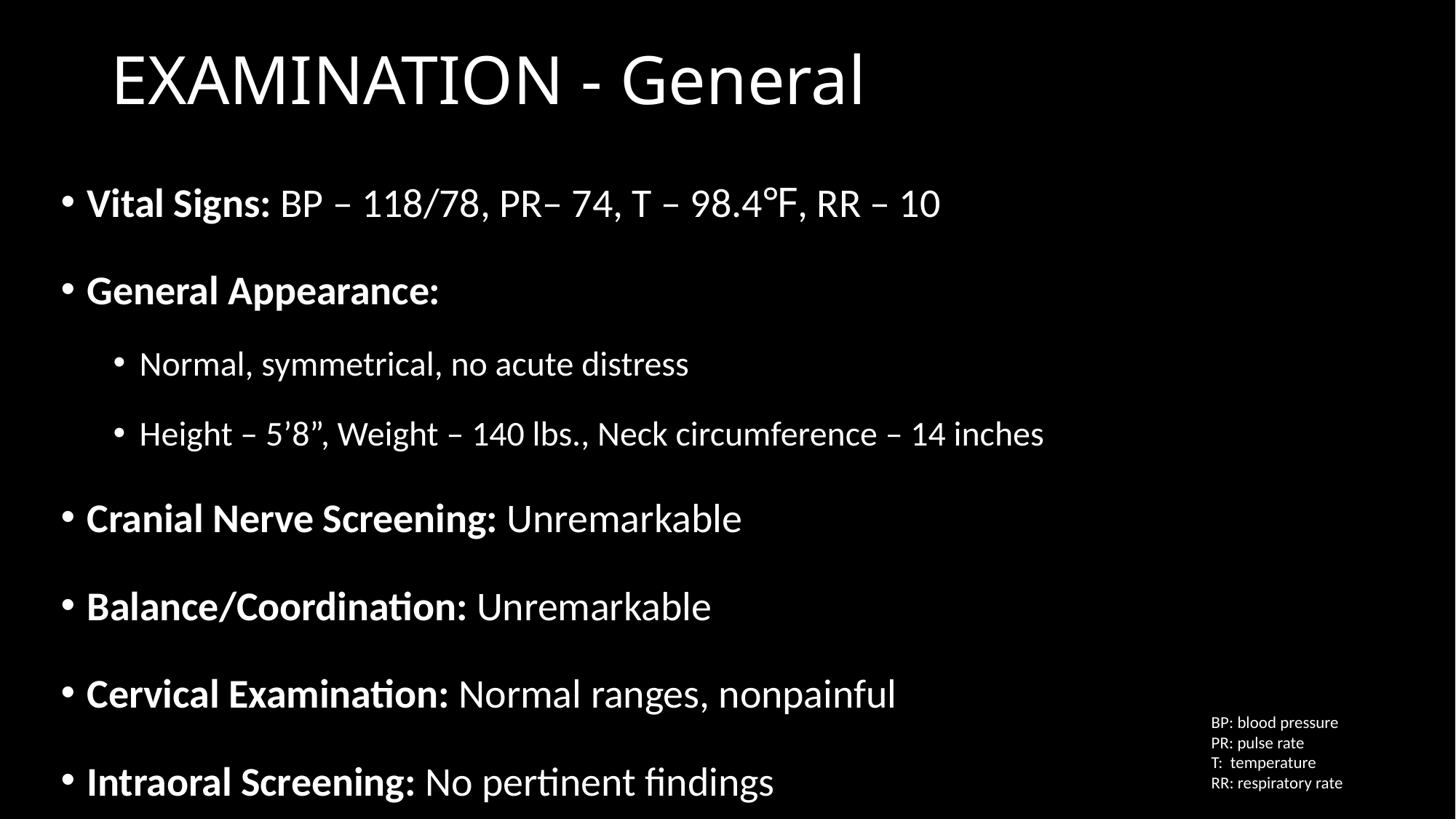

# EXAMINATION - General
Vital Signs: BP – 118/78, PR– 74, T – 98.4℉, RR – 10
General Appearance:
Normal, symmetrical, no acute distress
Height – 5’8”, Weight – 140 lbs., Neck circumference – 14 inches
Cranial Nerve Screening: Unremarkable
Balance/Coordination: Unremarkable
Cervical Examination: Normal ranges, nonpainful
Intraoral Screening: No pertinent findings
BP: blood pressure
PR: pulse rate
T: temperature
RR: respiratory rate

## Slide 23
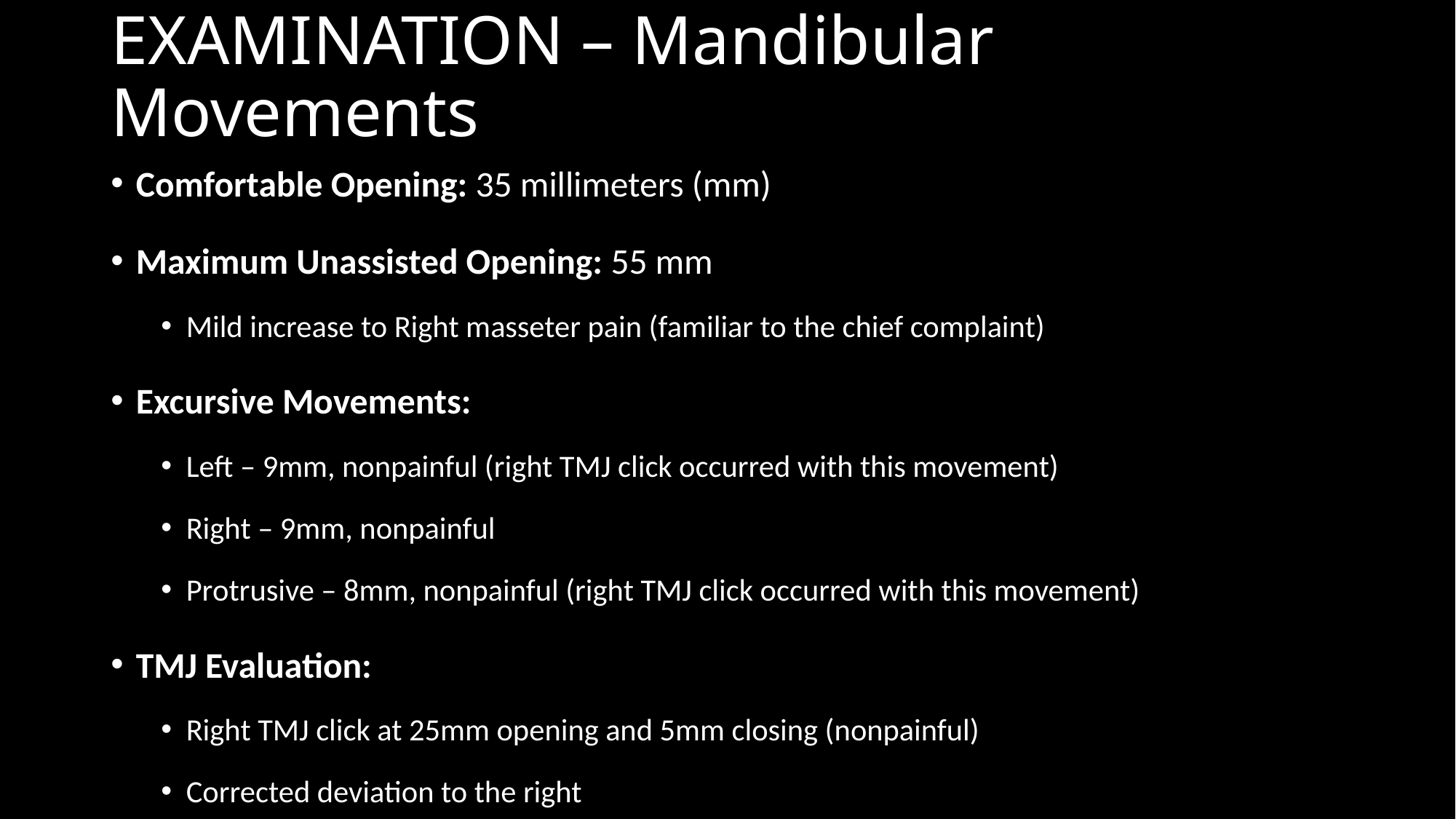

# EXAMINATION – Mandibular Movements
Comfortable Opening: 35 millimeters (mm)
Maximum Unassisted Opening: 55 mm
Mild increase to Right masseter pain (familiar to the chief complaint)
Excursive Movements:
Left – 9mm, nonpainful (right TMJ click occurred with this movement)
Right – 9mm, nonpainful
Protrusive – 8mm, nonpainful (right TMJ click occurred with this movement)
TMJ Evaluation:
Right TMJ click at 25mm opening and 5mm closing (nonpainful)
Corrected deviation to the right

## Slide 24
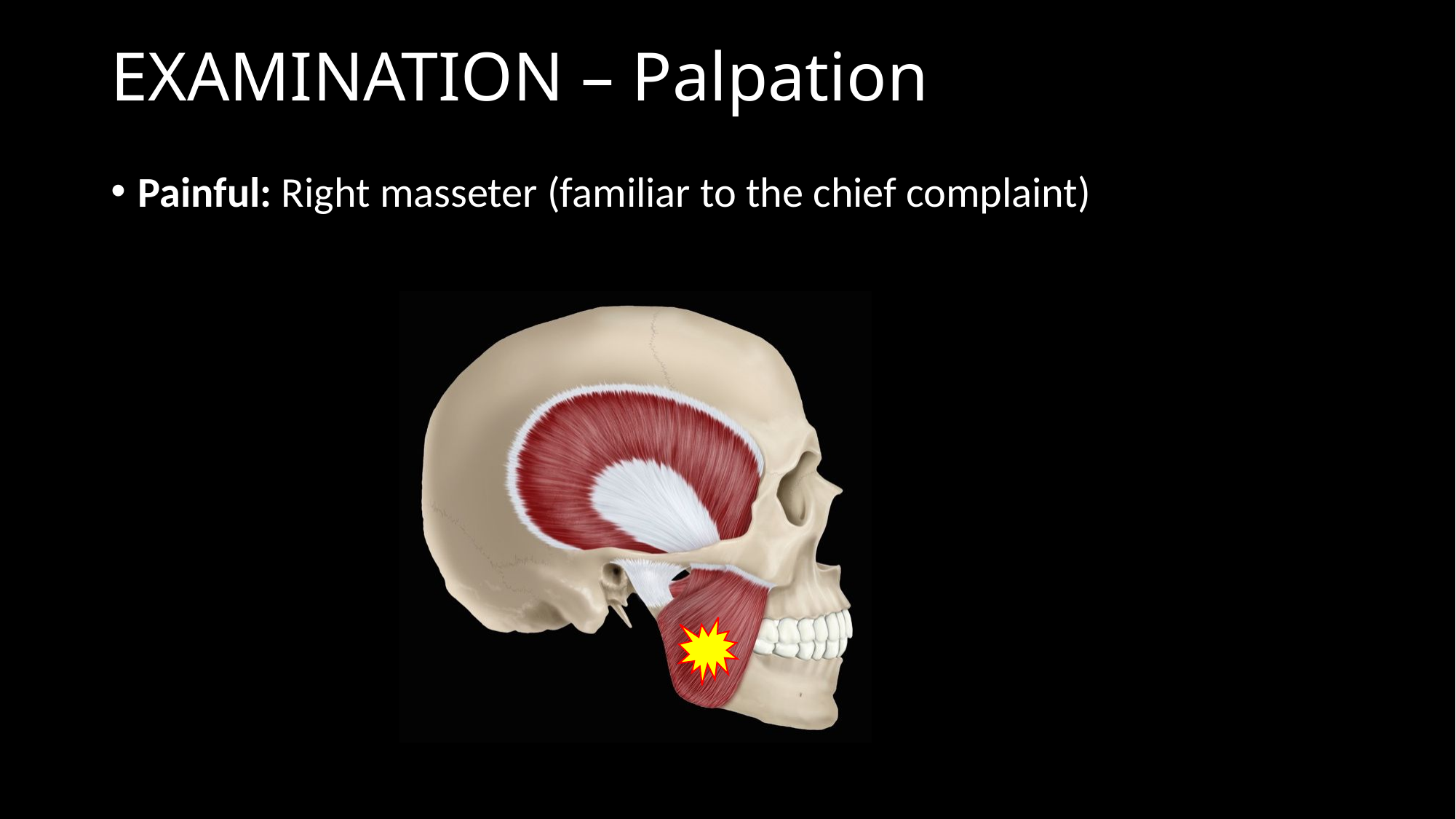

# EXAMINATION – Palpation
Painful: Right masseter (familiar to the chief complaint)

## Slide 25
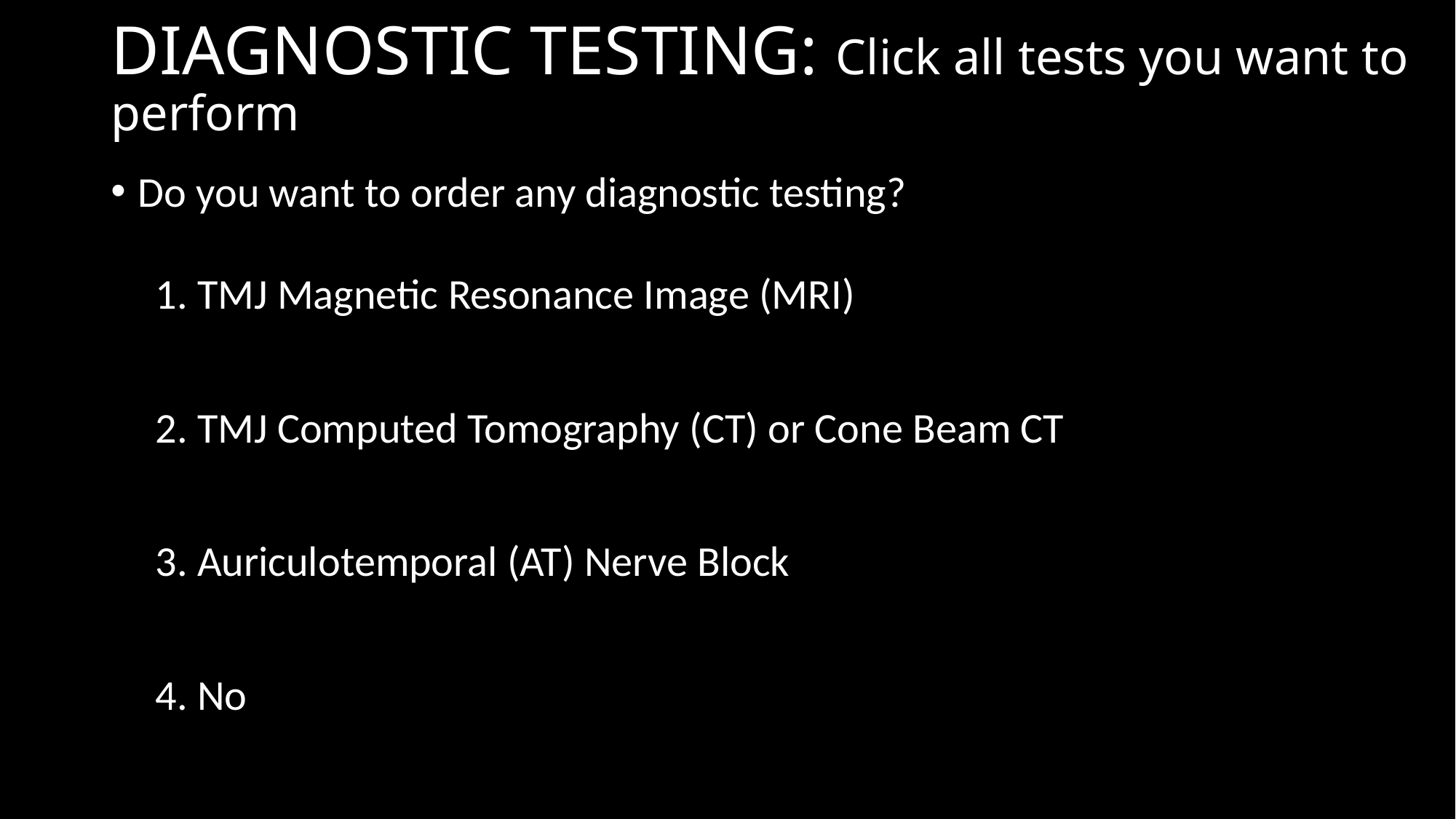

DIAGNOSTIC TESTING: Click all tests you want to perform
Do you want to order any diagnostic testing?
1. TMJ Magnetic Resonance Image (MRI)
2. TMJ Computed Tomography (CT) or Cone Beam CT
3. Auriculotemporal (AT) Nerve Block
4. No

## Slide 26
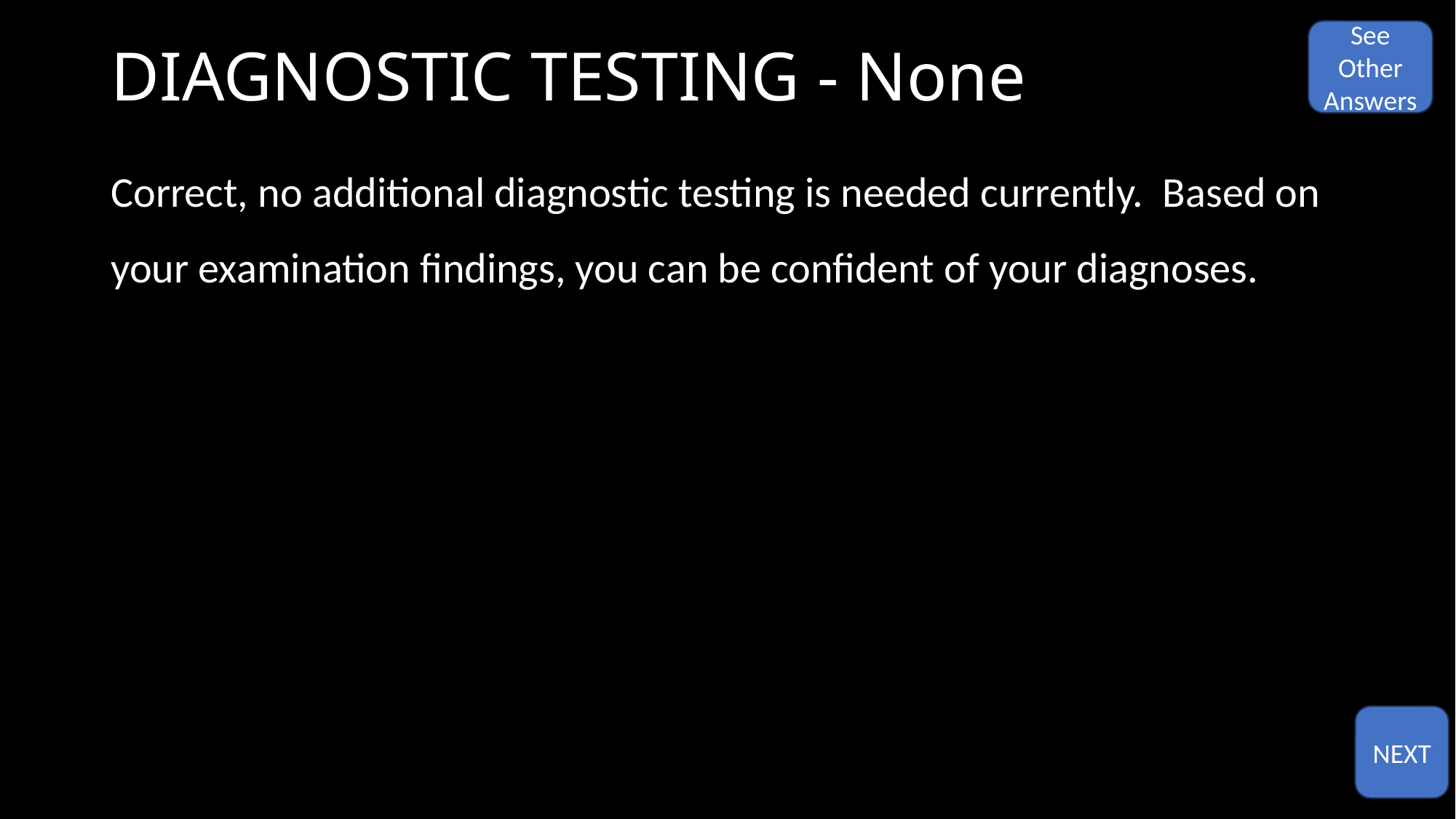

# DIAGNOSTIC TESTING - None
See Other Answers
Correct, no additional diagnostic testing is needed currently. Based on your examination findings, you can be confident of your diagnoses.
NEXT

## Slide 27
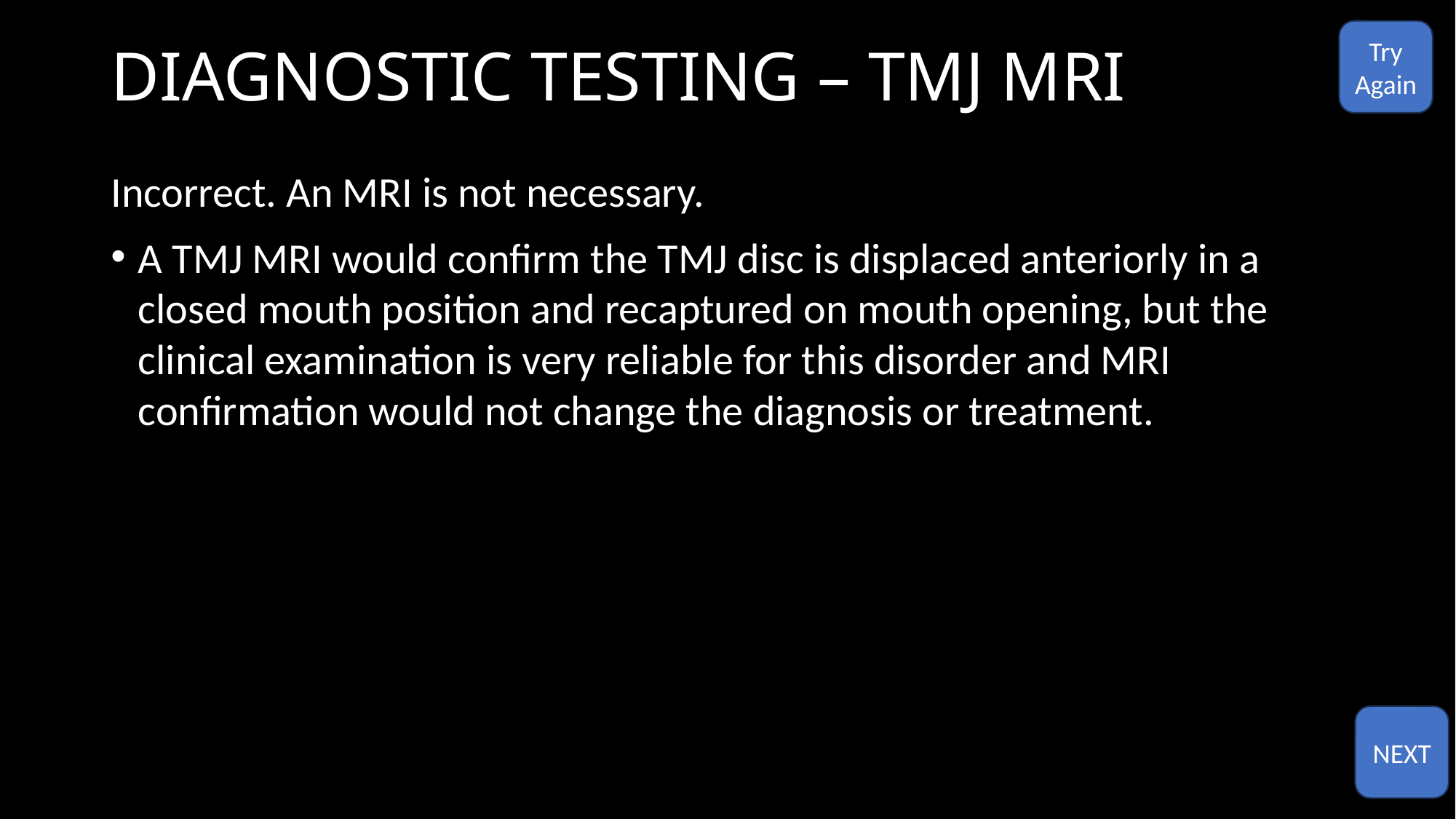

# DIAGNOSTIC TESTING – TMJ MRI
Try
Again
Incorrect. An MRI is not necessary.
A TMJ MRI would confirm the TMJ disc is displaced anteriorly in a closed mouth position and recaptured on mouth opening, but the clinical examination is very reliable for this disorder and MRI confirmation would not change the diagnosis or treatment.
NEXT

## Slide 28
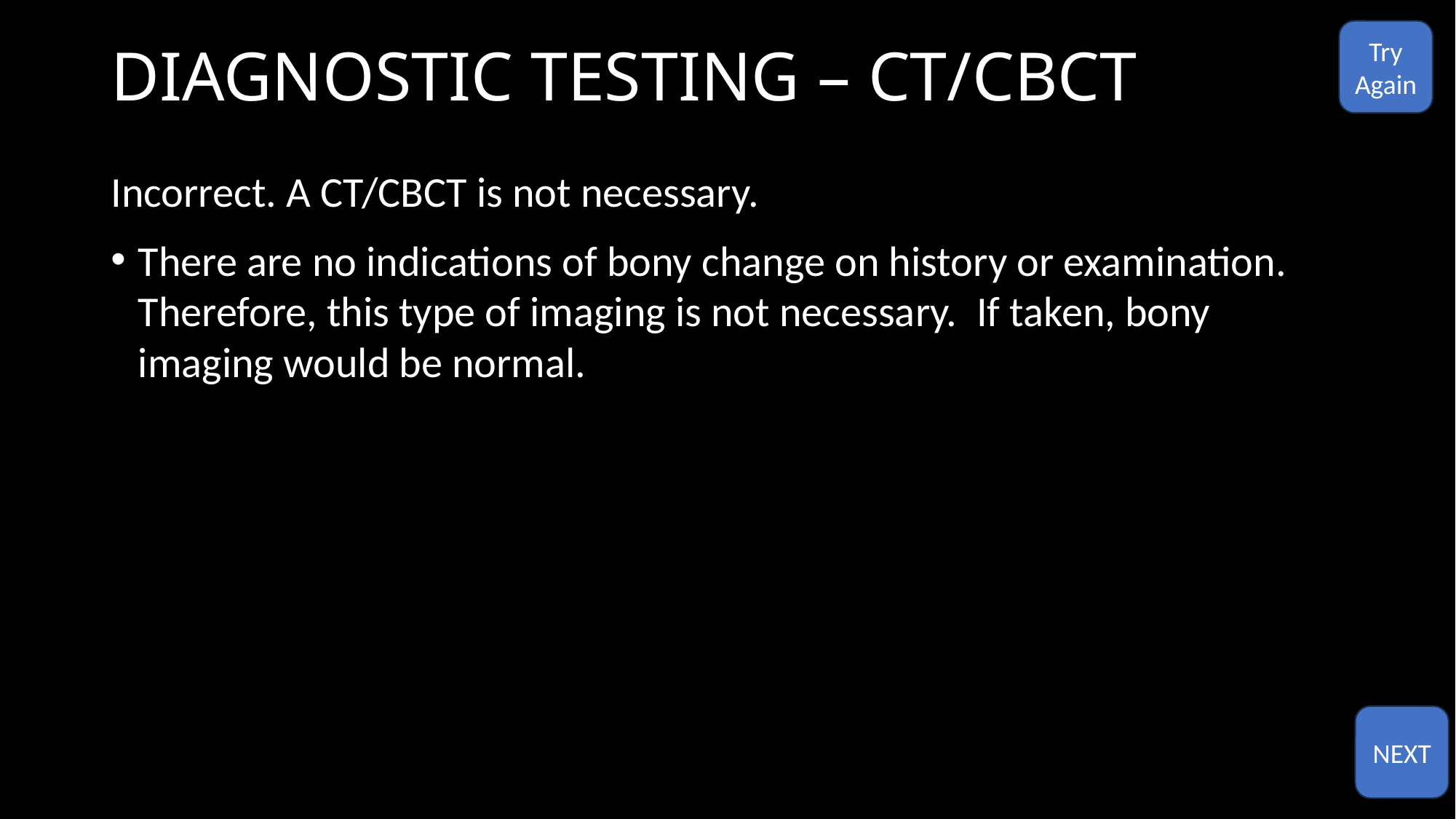

# DIAGNOSTIC TESTING – CT/CBCT
Try
Again
Incorrect. A CT/CBCT is not necessary.
There are no indications of bony change on history or examination. Therefore, this type of imaging is not necessary. If taken, bony imaging would be normal.
NEXT

## Slide 29
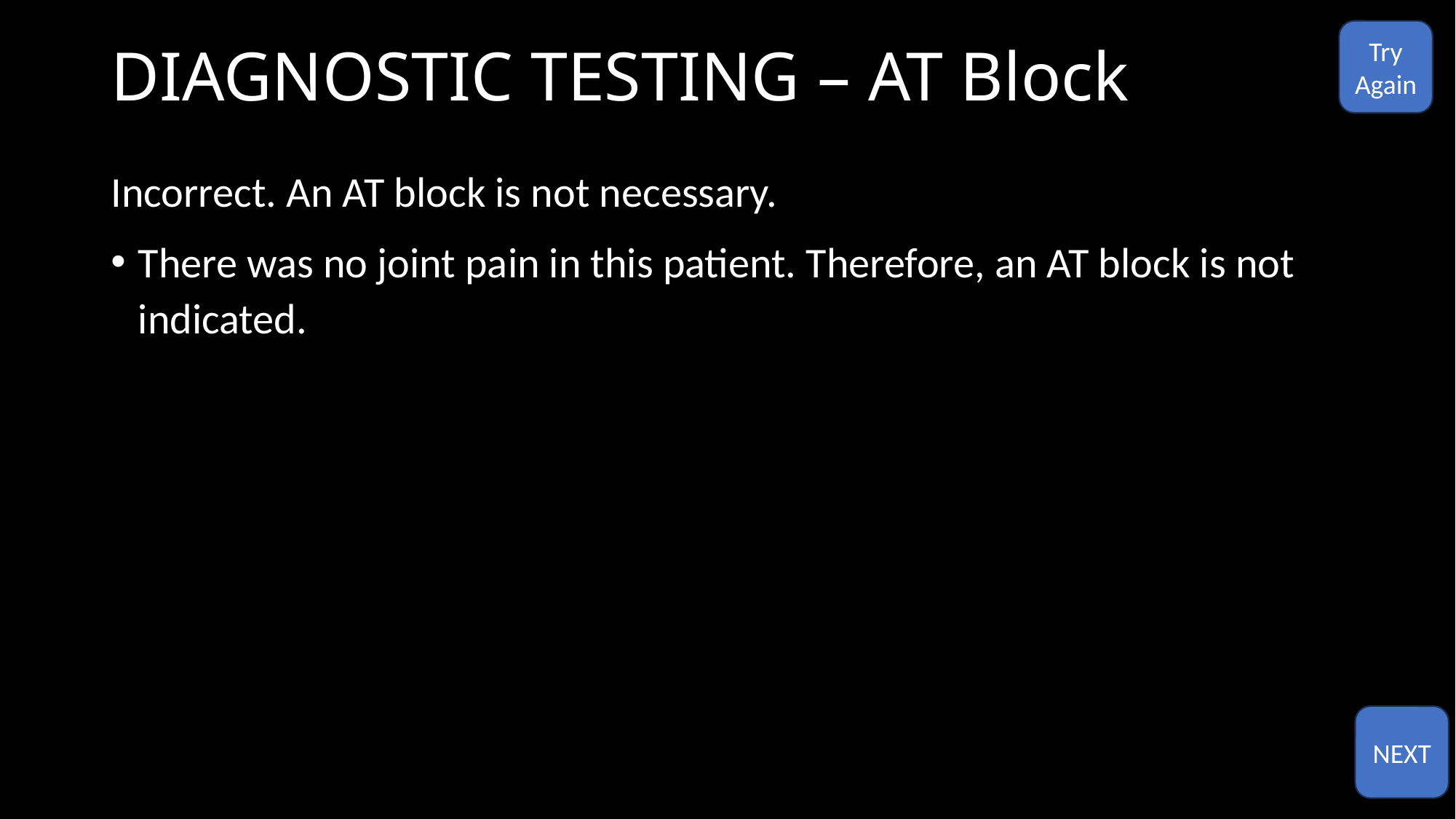

# DIAGNOSTIC TESTING – AT Block
Try
Again
Incorrect. An AT block is not necessary.
There was no joint pain in this patient. Therefore, an AT block is not indicated.
NEXT

## Slide 30
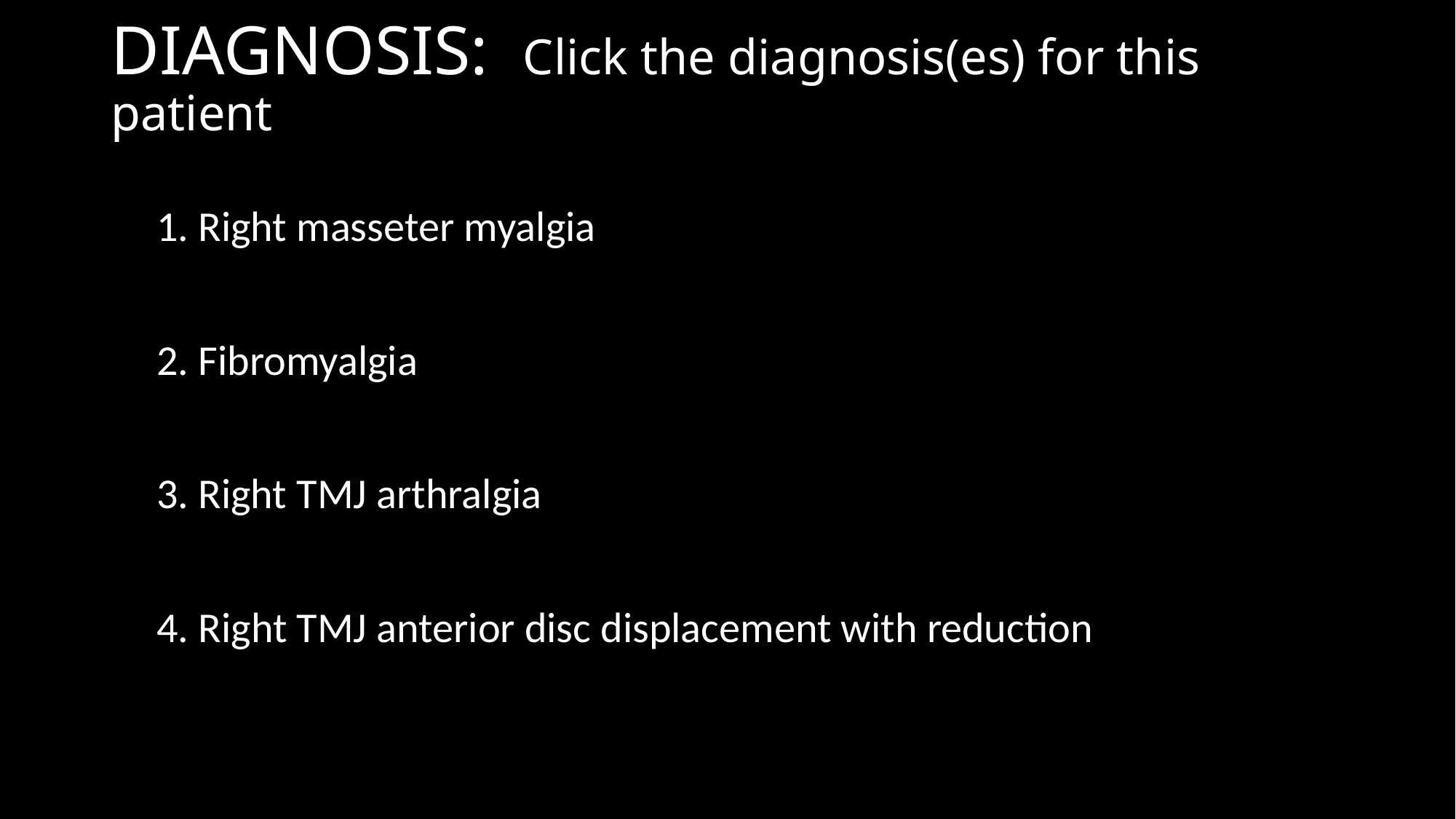

# DIAGNOSIS: Click the diagnosis(es) for this patient
1. Right masseter myalgia
2. Fibromyalgia
3. Right TMJ arthralgia
4. Right TMJ anterior disc displacement with reduction

## Slide 31
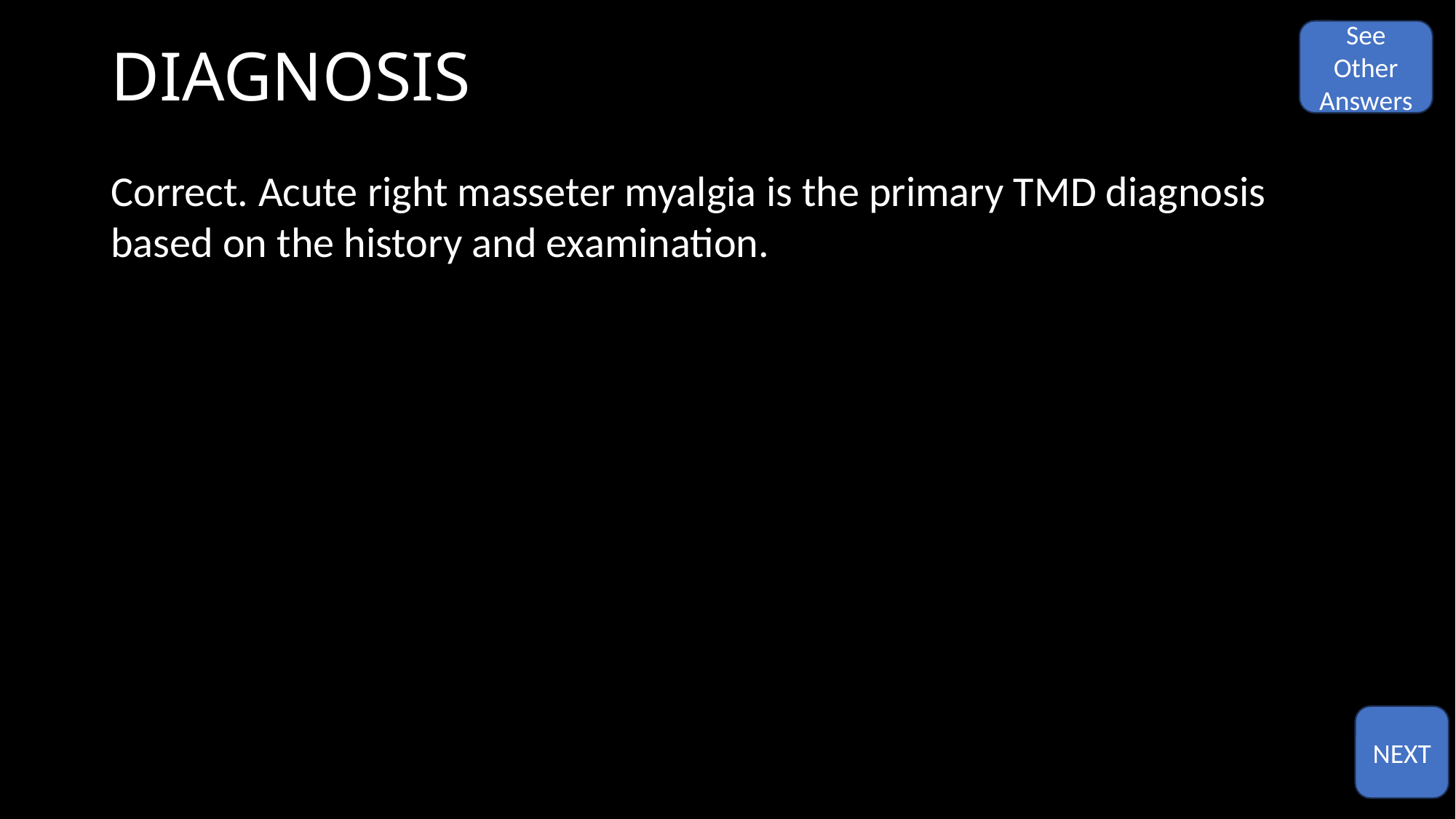

# DIAGNOSIS
See Other Answers
Correct. Acute right masseter myalgia is the primary TMD diagnosis based on the history and examination.
NEXT

## Slide 32
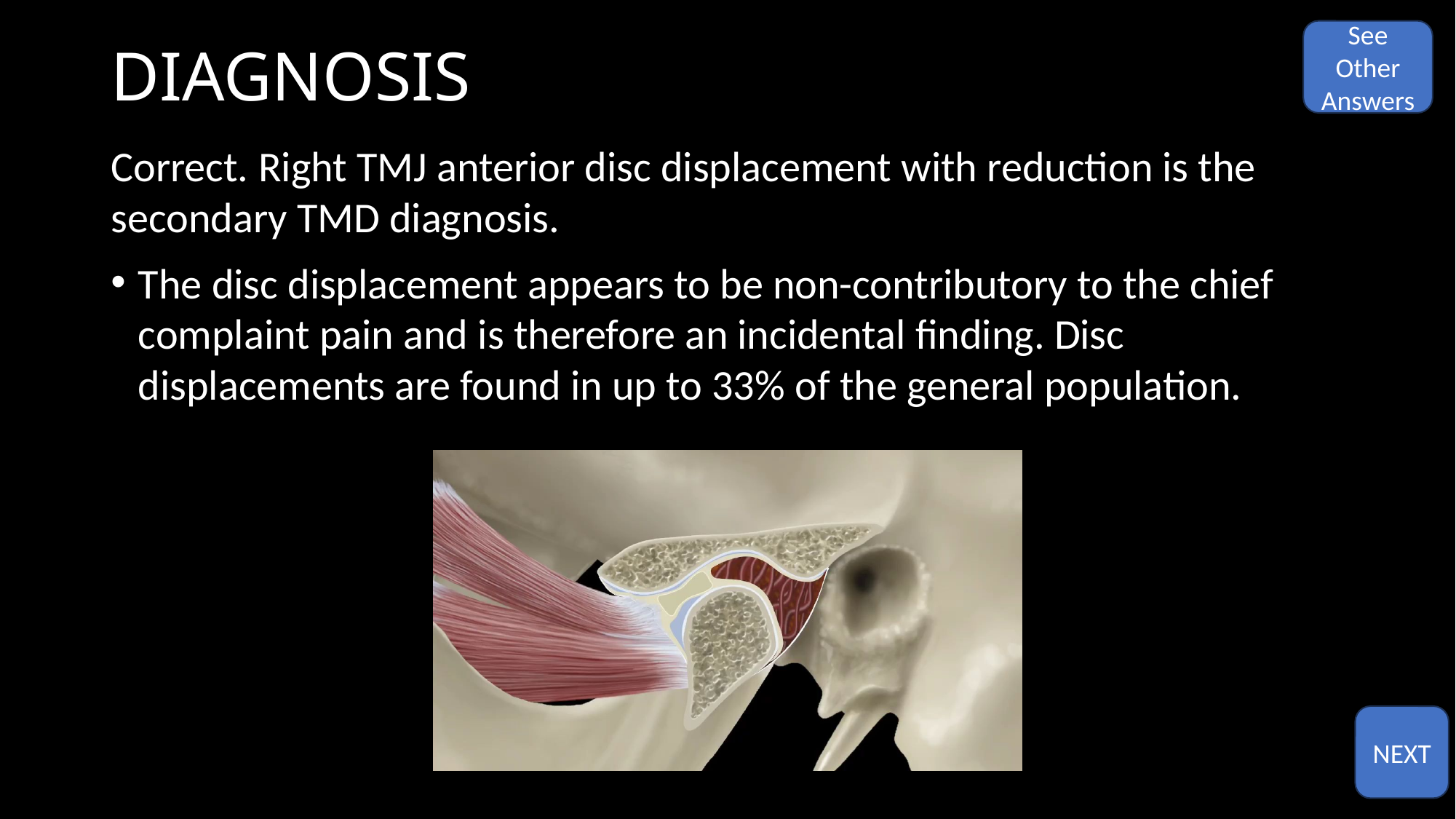

# DIAGNOSIS
See Other Answers
Correct. Right TMJ anterior disc displacement with reduction is the secondary TMD diagnosis.
The disc displacement appears to be non-contributory to the chief complaint pain and is therefore an incidental finding. Disc displacements are found in up to 33% of the general population.
NEXT

## Slide 33
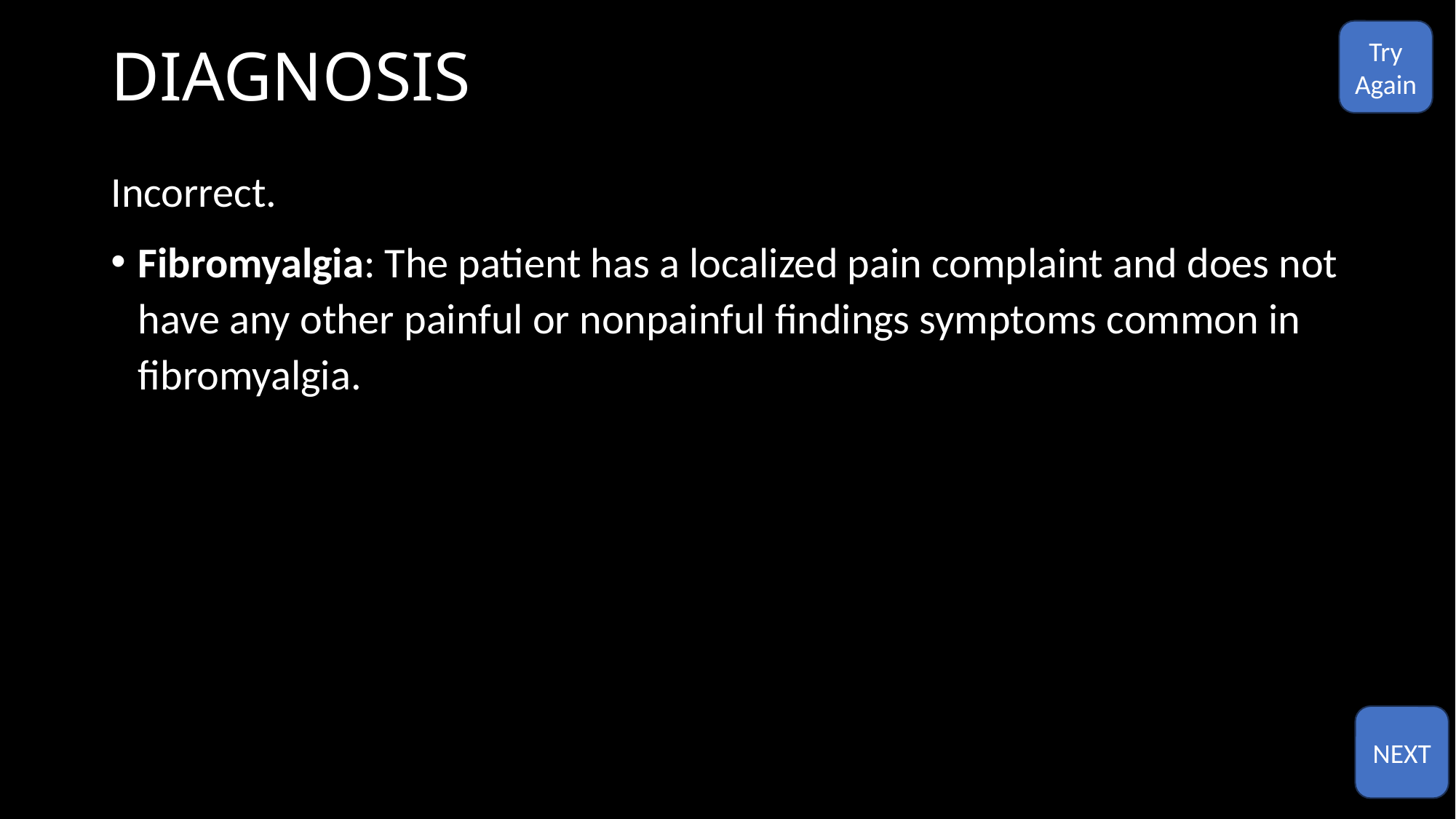

# DIAGNOSIS
Try
Again
Incorrect.
Fibromyalgia: The patient has a localized pain complaint and does not have any other painful or nonpainful findings symptoms common in fibromyalgia.
NEXT

## Slide 34
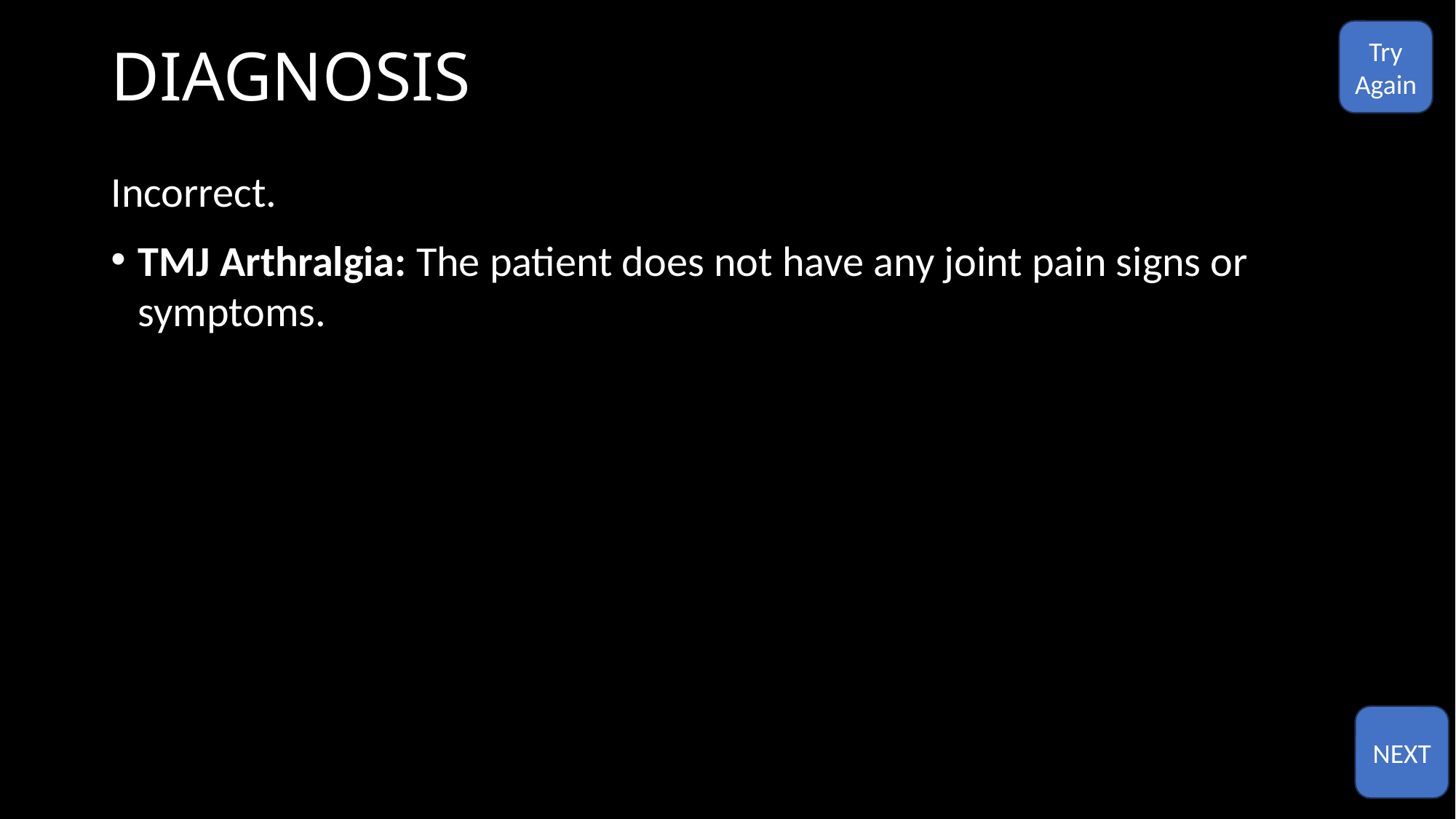

# DIAGNOSIS
Try
Again
Incorrect.
TMJ Arthralgia: The patient does not have any joint pain signs or symptoms.
NEXT

## Slide 35
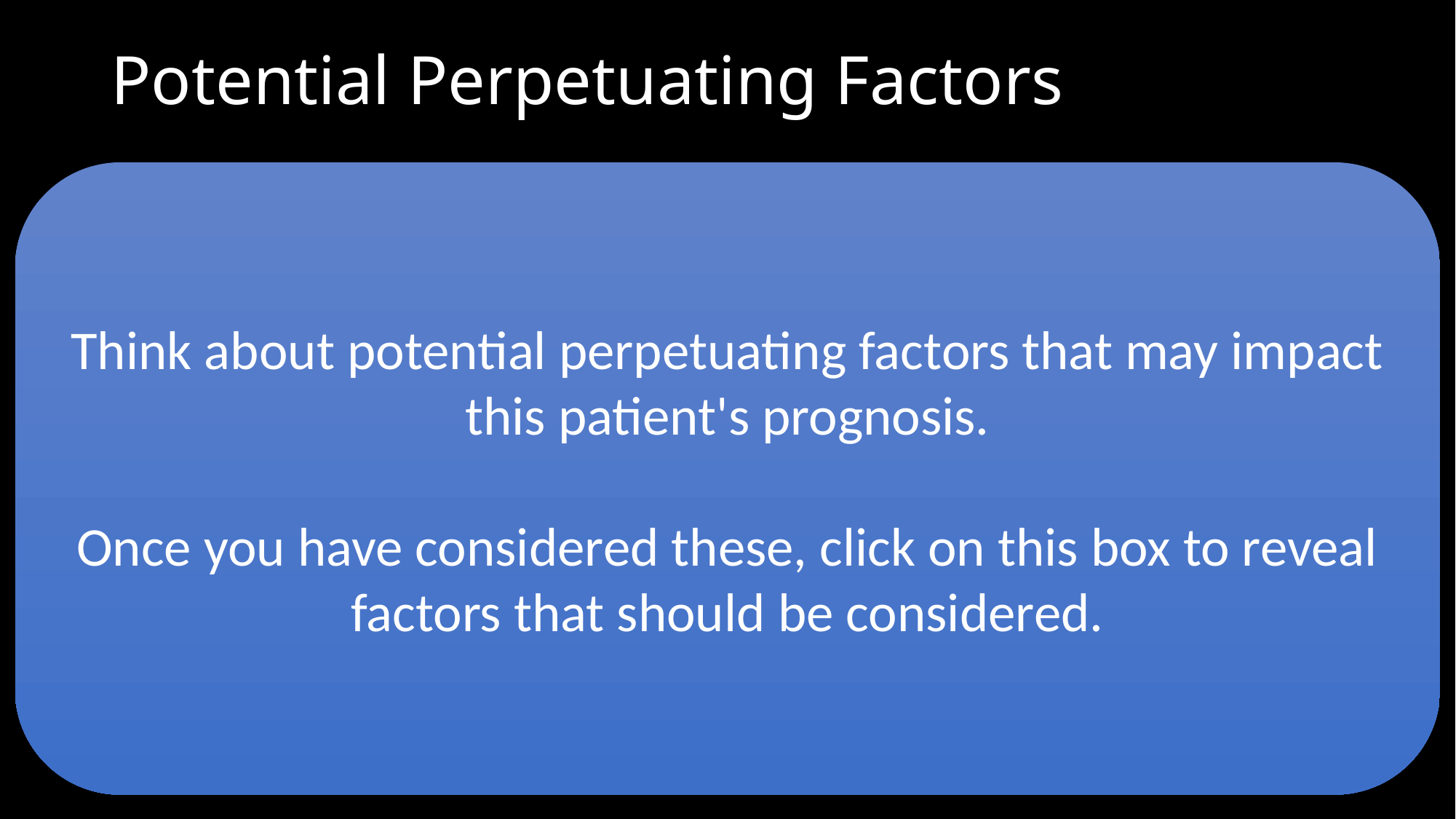

# Potential Perpetuating Factors
Stress Reactivity
Onset of chief complaint temporally related to stressful new job
Oral Parafunction
Teeth together, tongue to palate, chewing gum
Caffeine
3 cups of coffee (300mg caffeine)
Think about potential perpetuating factors that may impact this patient's prognosis.
Once you have considered these, click on this box to reveal factors that should be considered.

## Slide 36
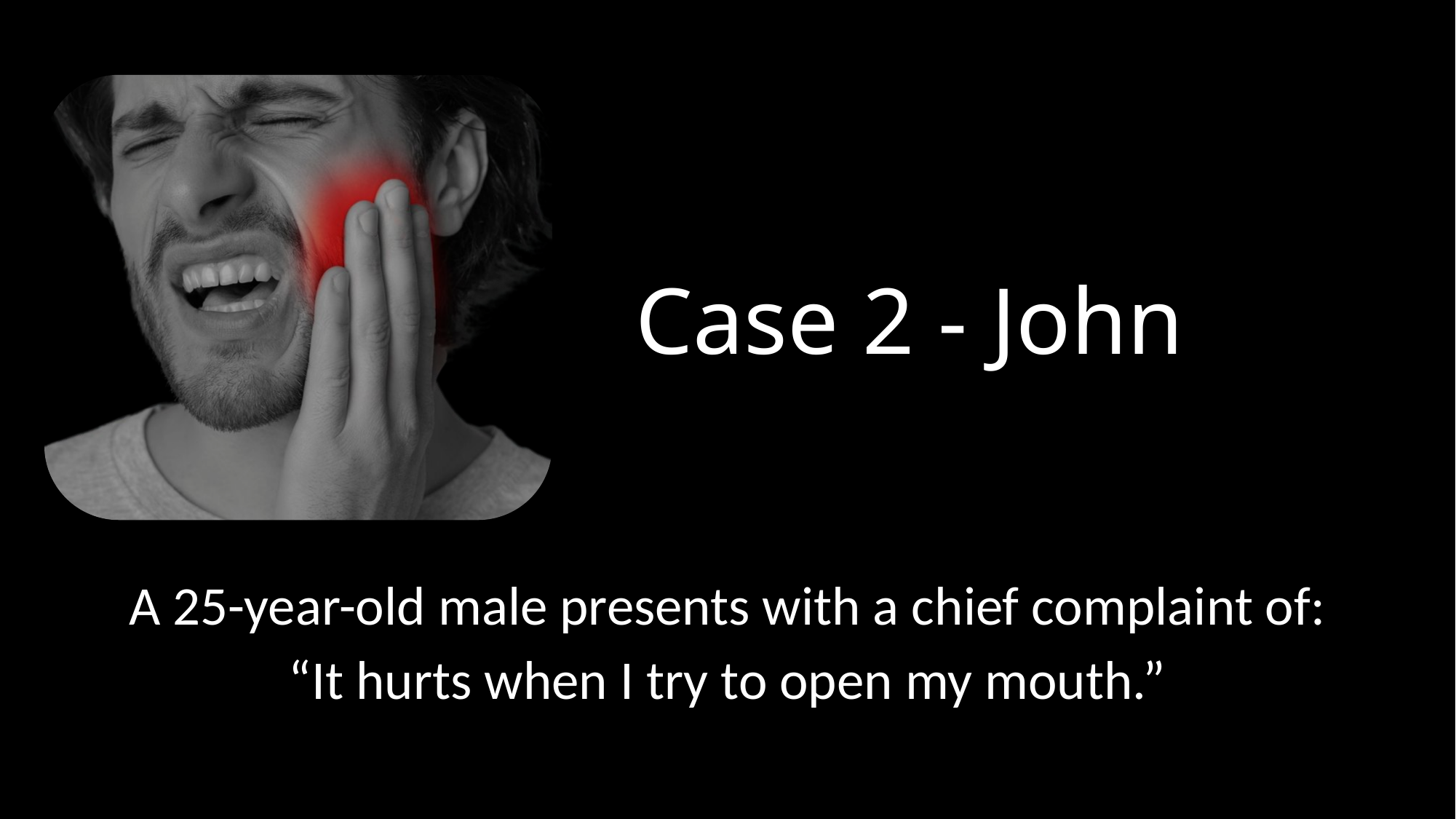

# Case 2 - John
A 25-year-old male presents with a chief complaint of:
“It hurts when I try to open my mouth.”

## Slide 37
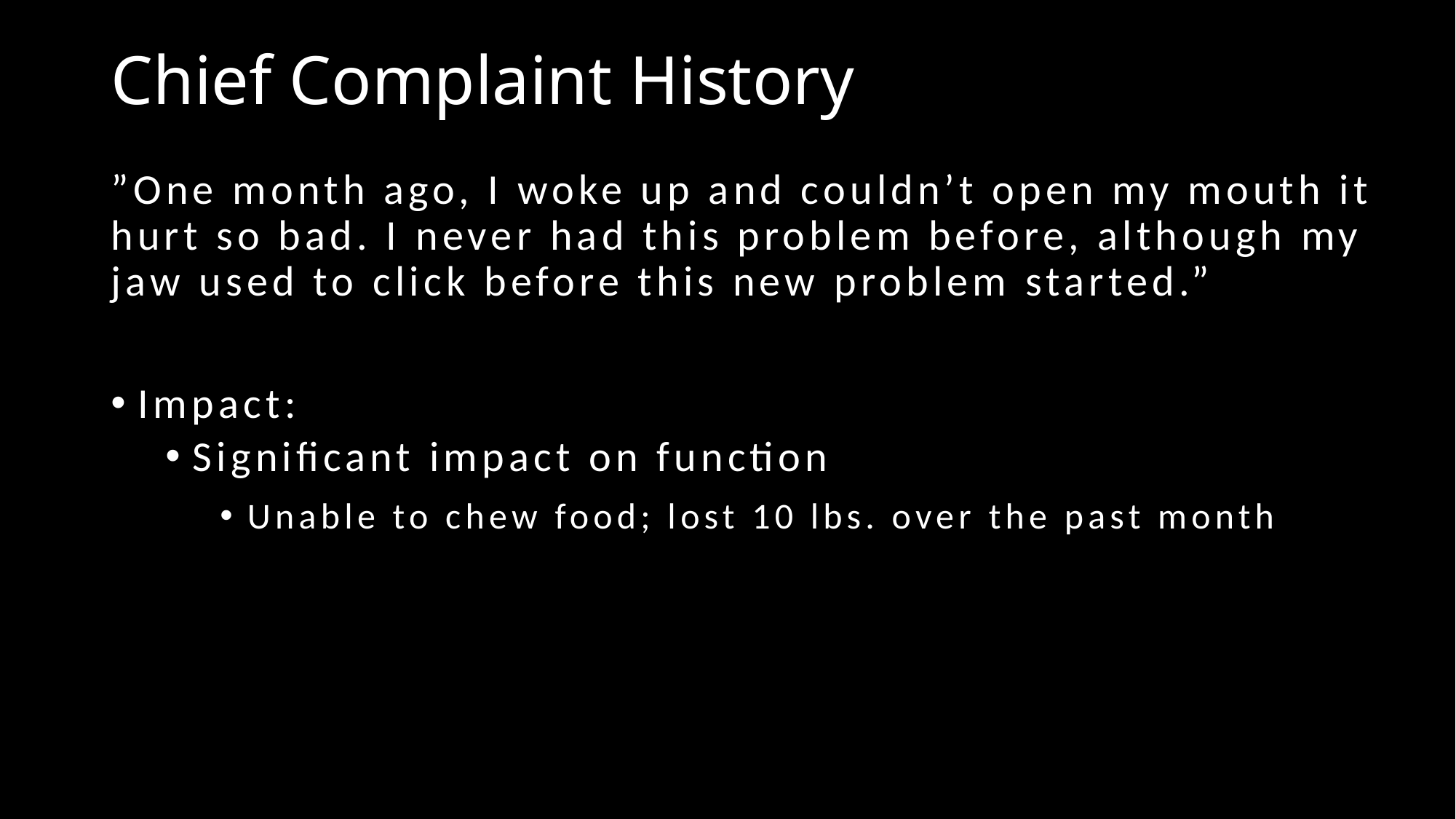

# Chief Complaint History
”One month ago, I woke up and couldn’t open my mouth it hurt so bad. I never had this problem before, although my jaw used to click before this new problem started.”
Impact:
Significant impact on function
Unable to chew food; lost 10 lbs. over the past month

## Slide 38
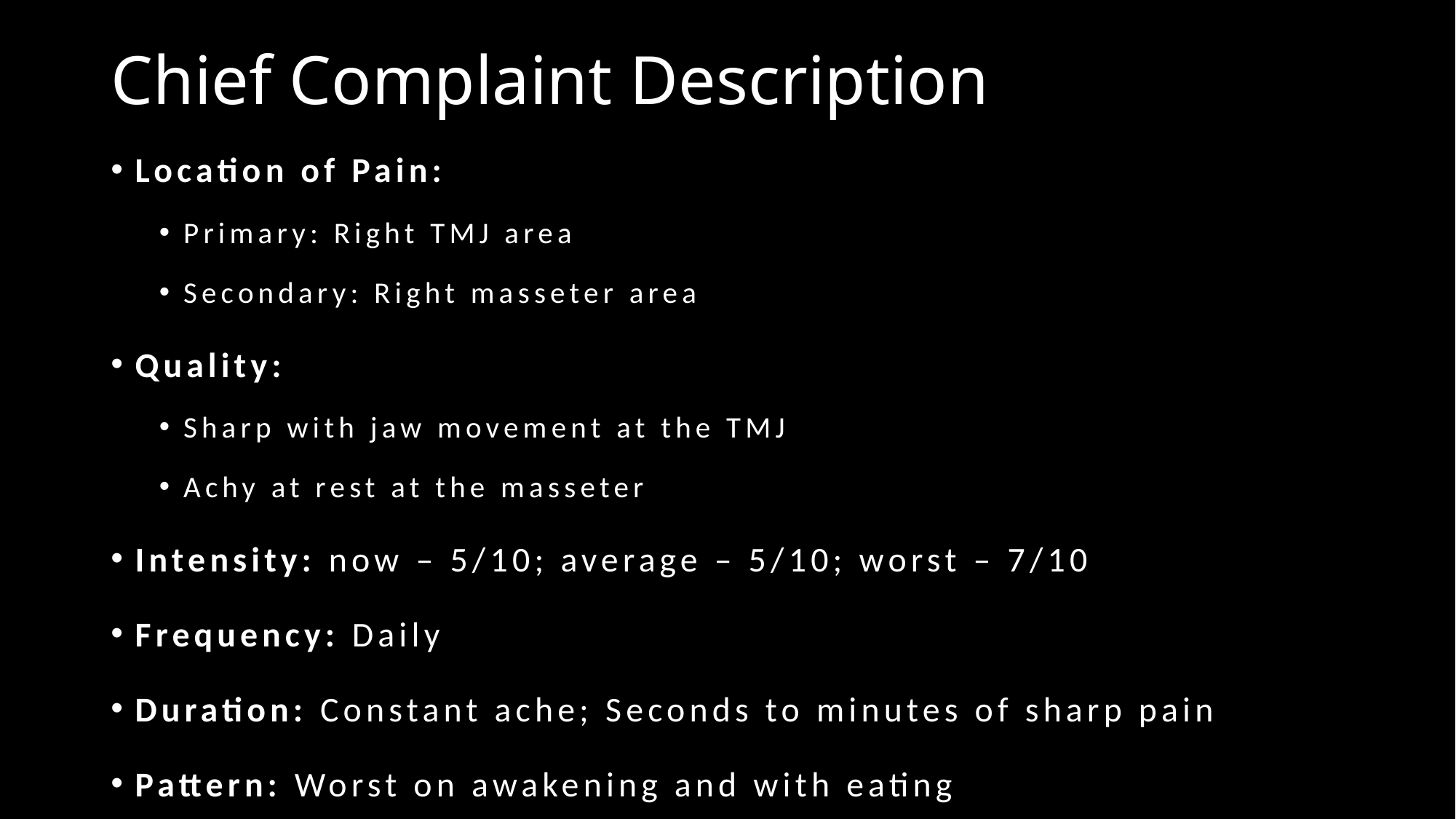

# Chief Complaint Description
Location of Pain:
Primary: Right TMJ area
Secondary: Right masseter area
Quality:
Sharp with jaw movement at the TMJ
Achy at rest at the masseter
Intensity: now – 5/10; average – 5/10; worst – 7/10
Frequency: Daily
Duration: Constant ache; Seconds to minutes of sharp pain
Pattern: Worst on awakening and with eating

## Slide 39
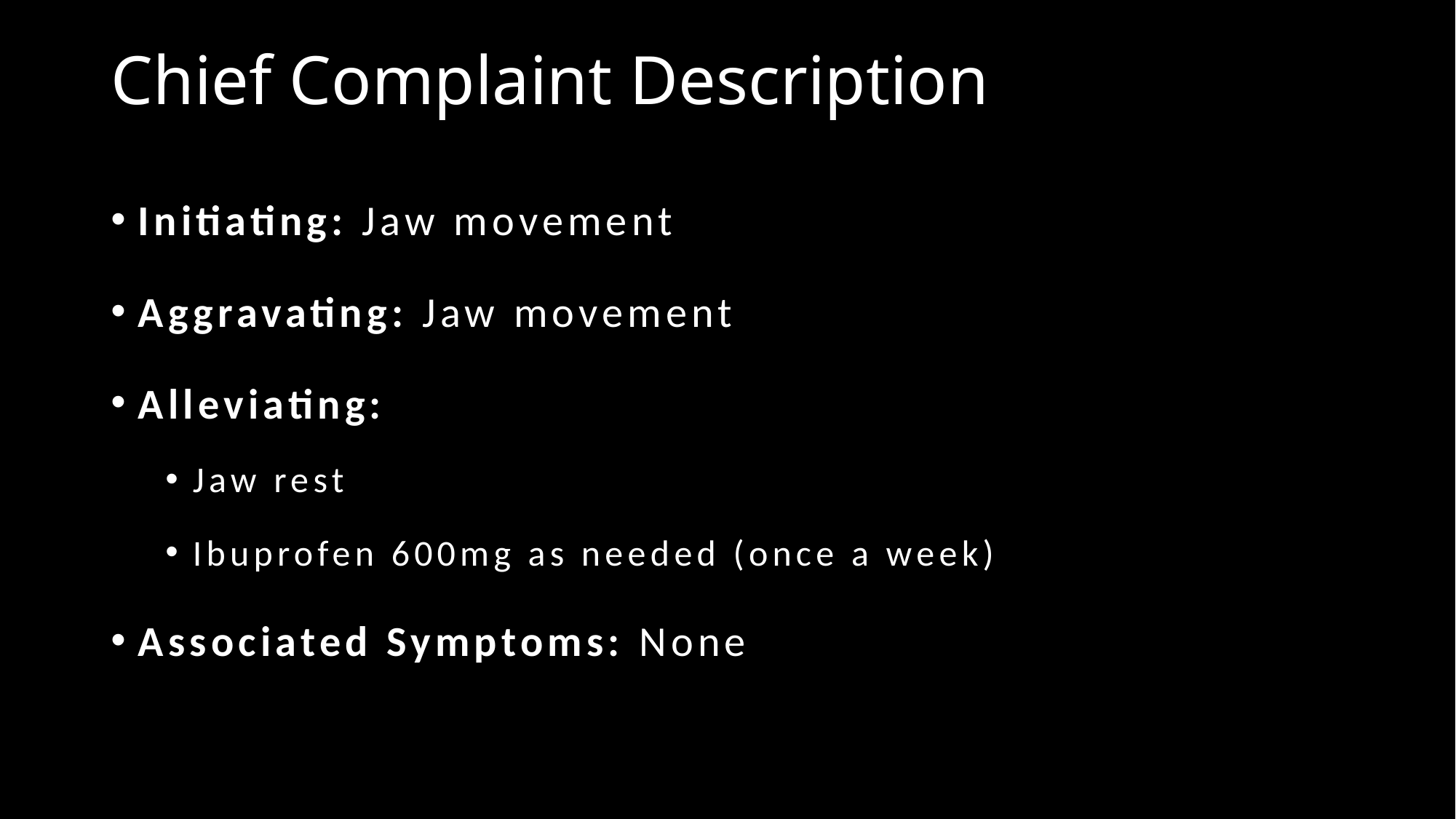

# Chief Complaint Description
Initiating: Jaw movement
Aggravating: Jaw movement
Alleviating:
Jaw rest
Ibuprofen 600mg as needed (once a week)
Associated Symptoms: None

## Slide 40
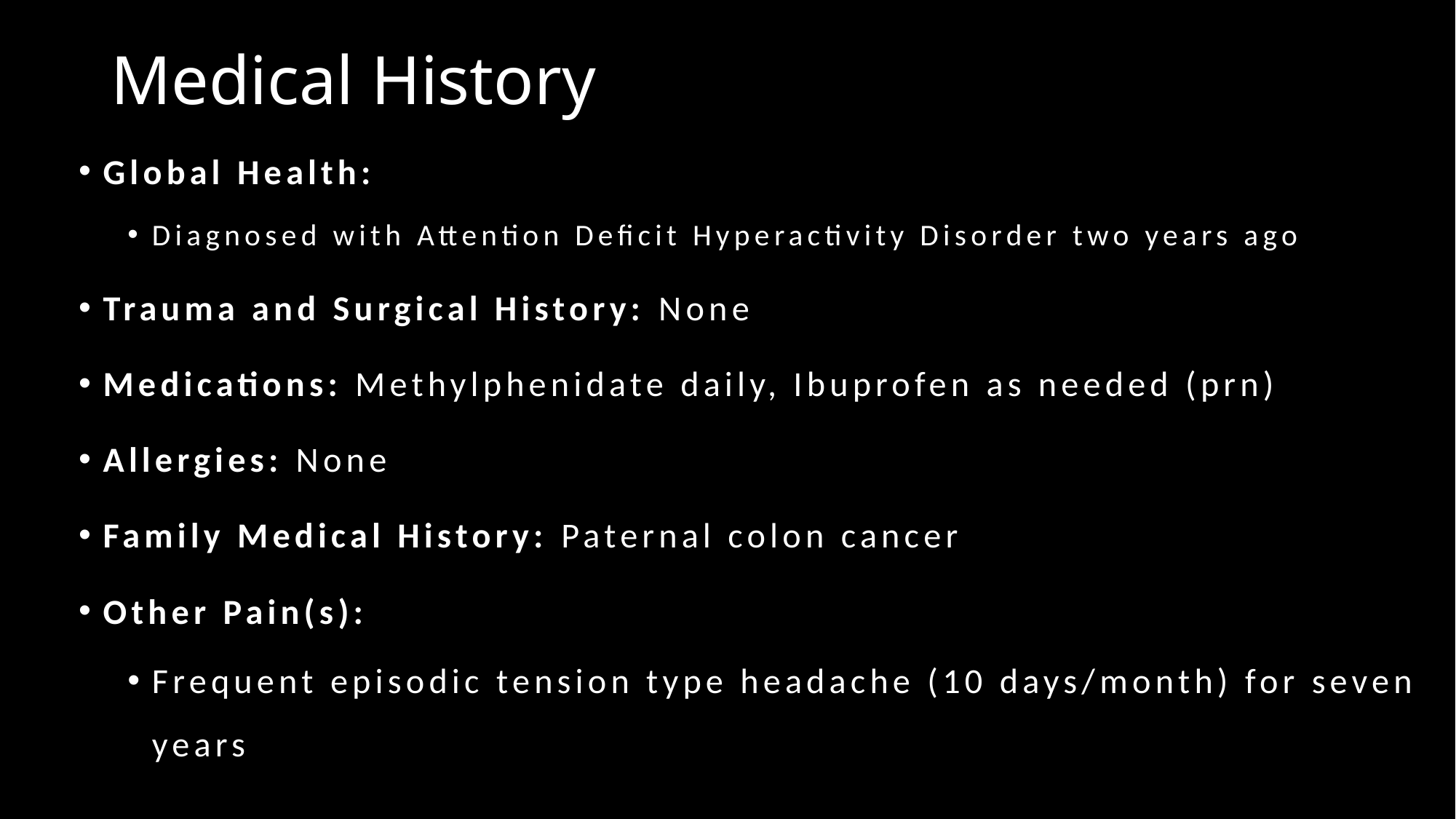

# Medical History
Global Health:
Diagnosed with Attention Deficit Hyperactivity Disorder two years ago
Trauma and Surgical History: None
Medications: Methylphenidate daily, Ibuprofen as needed (prn)
Allergies: None
Family Medical History: Paternal colon cancer
Other Pain(s):
Frequent episodic tension type headache (10 days/month) for seven years

## Slide 41
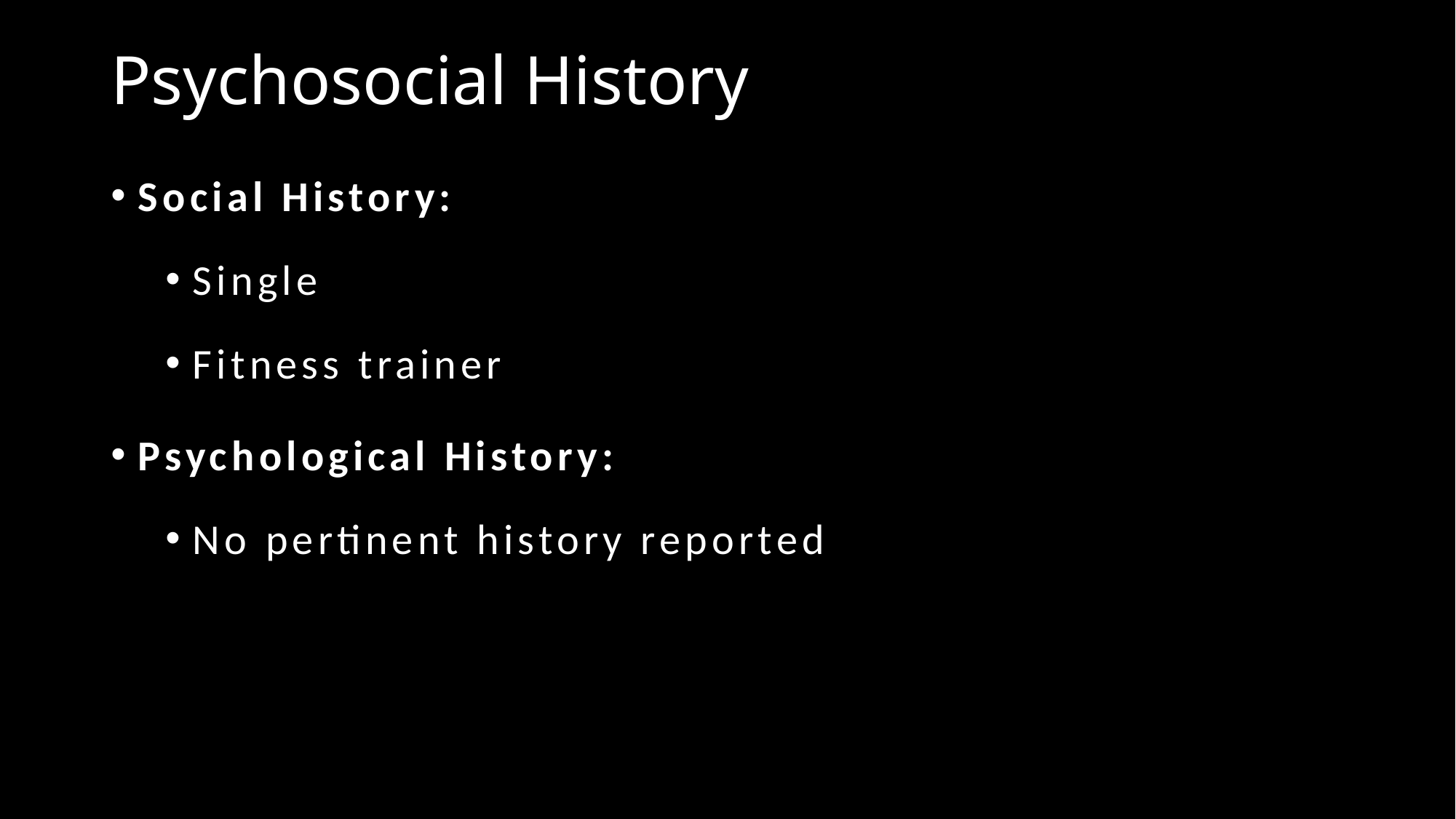

# Psychosocial History
Social History:
Single
Fitness trainer
Psychological History:
No pertinent history reported

## Slide 42
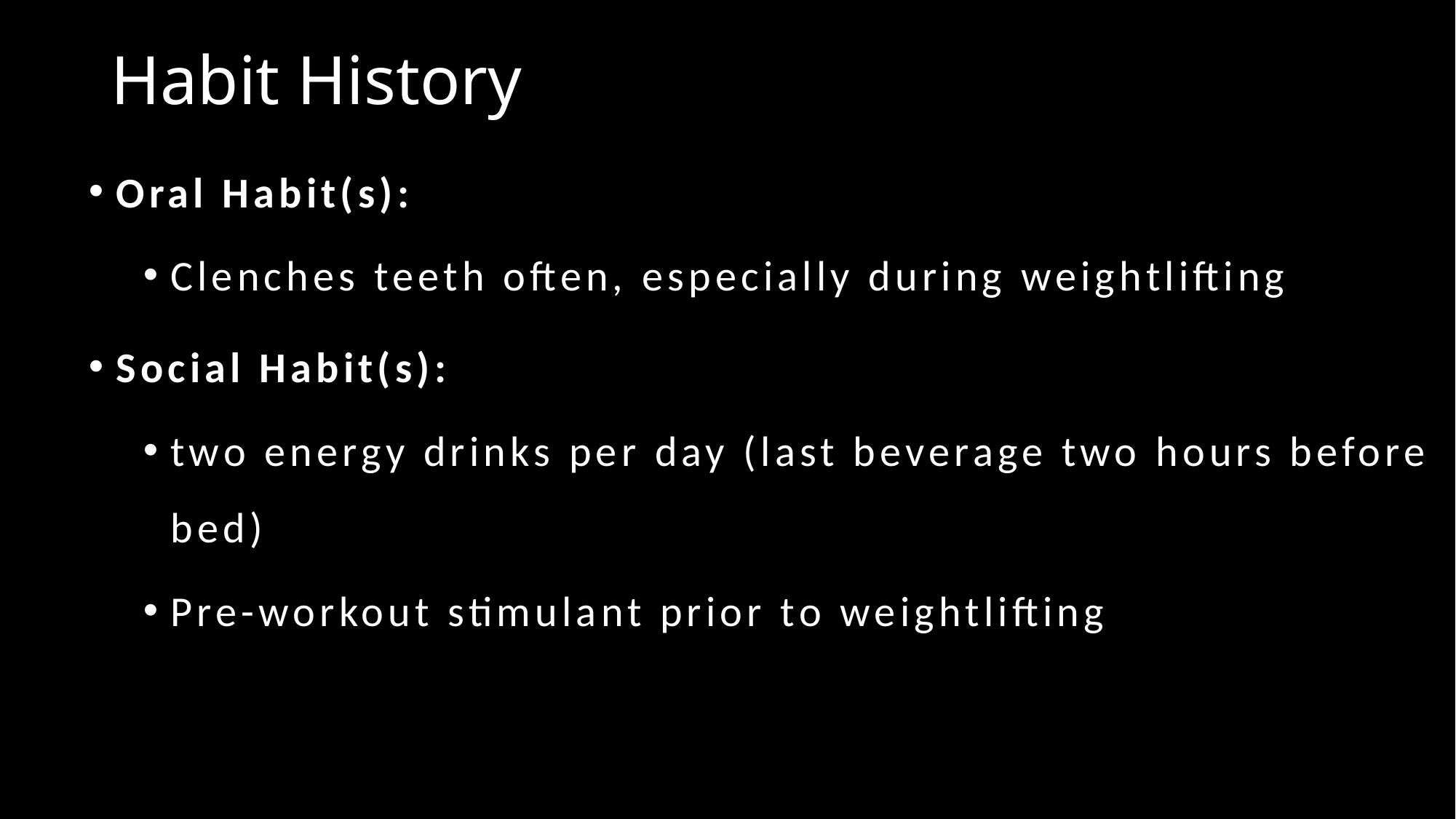

# Habit History
Oral Habit(s):
Clenches teeth often, especially during weightlifting
Social Habit(s):
two energy drinks per day (last beverage two hours before bed)
Pre-workout stimulant prior to weightlifting

## Slide 43
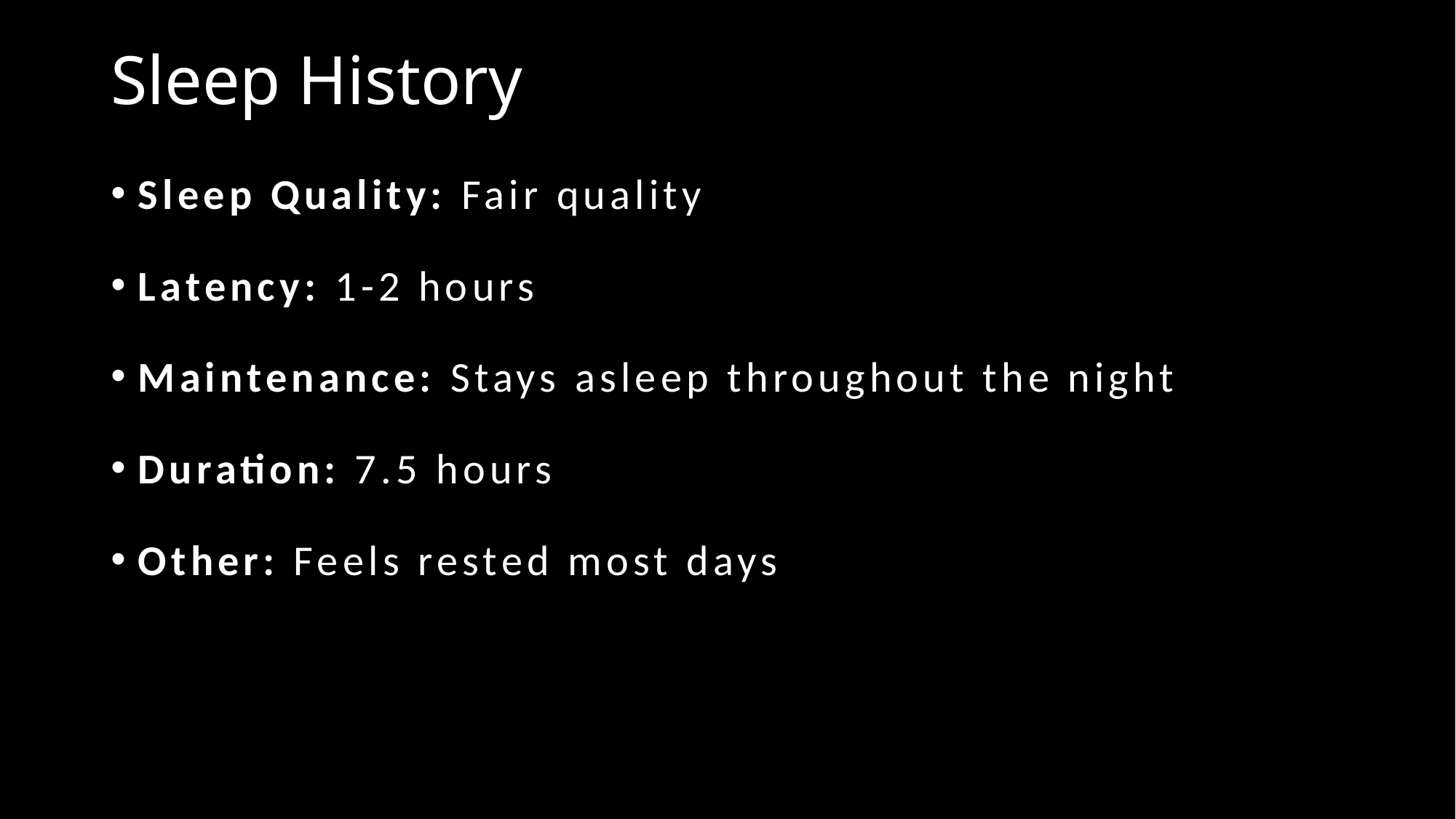

# Sleep History
Sleep Quality: Fair quality
Latency: 1-2 hours
Maintenance: Stays asleep throughout the night
Duration: 7.5 hours
Other: Feels rested most days

## Slide 44
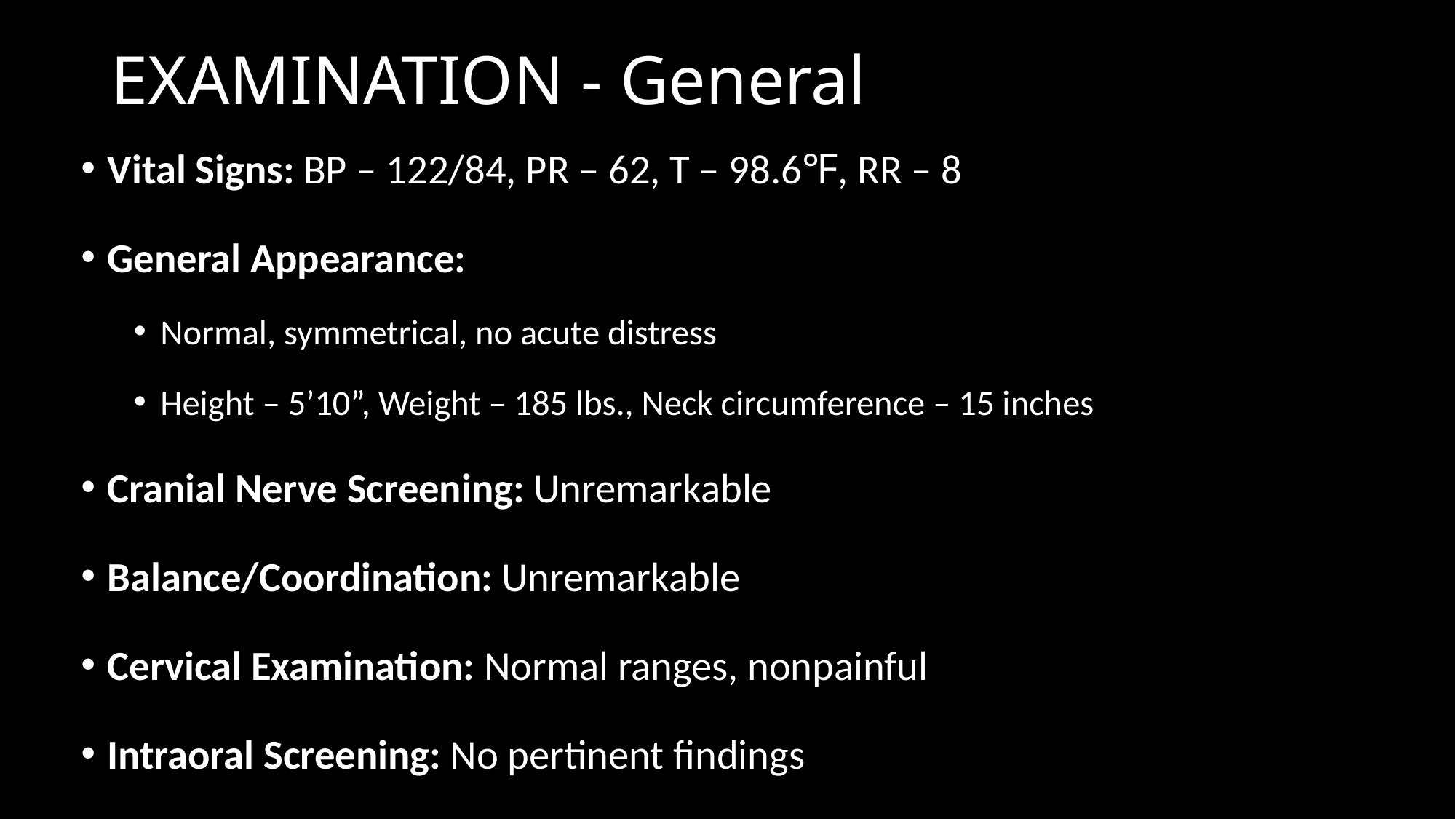

# EXAMINATION - General
Vital Signs: BP – 122/84, PR – 62, T – 98.6℉, RR – 8
General Appearance:
Normal, symmetrical, no acute distress
Height – 5’10”, Weight – 185 lbs., Neck circumference – 15 inches
Cranial Nerve Screening: Unremarkable
Balance/Coordination: Unremarkable
Cervical Examination: Normal ranges, nonpainful
Intraoral Screening: No pertinent findings

## Slide 45
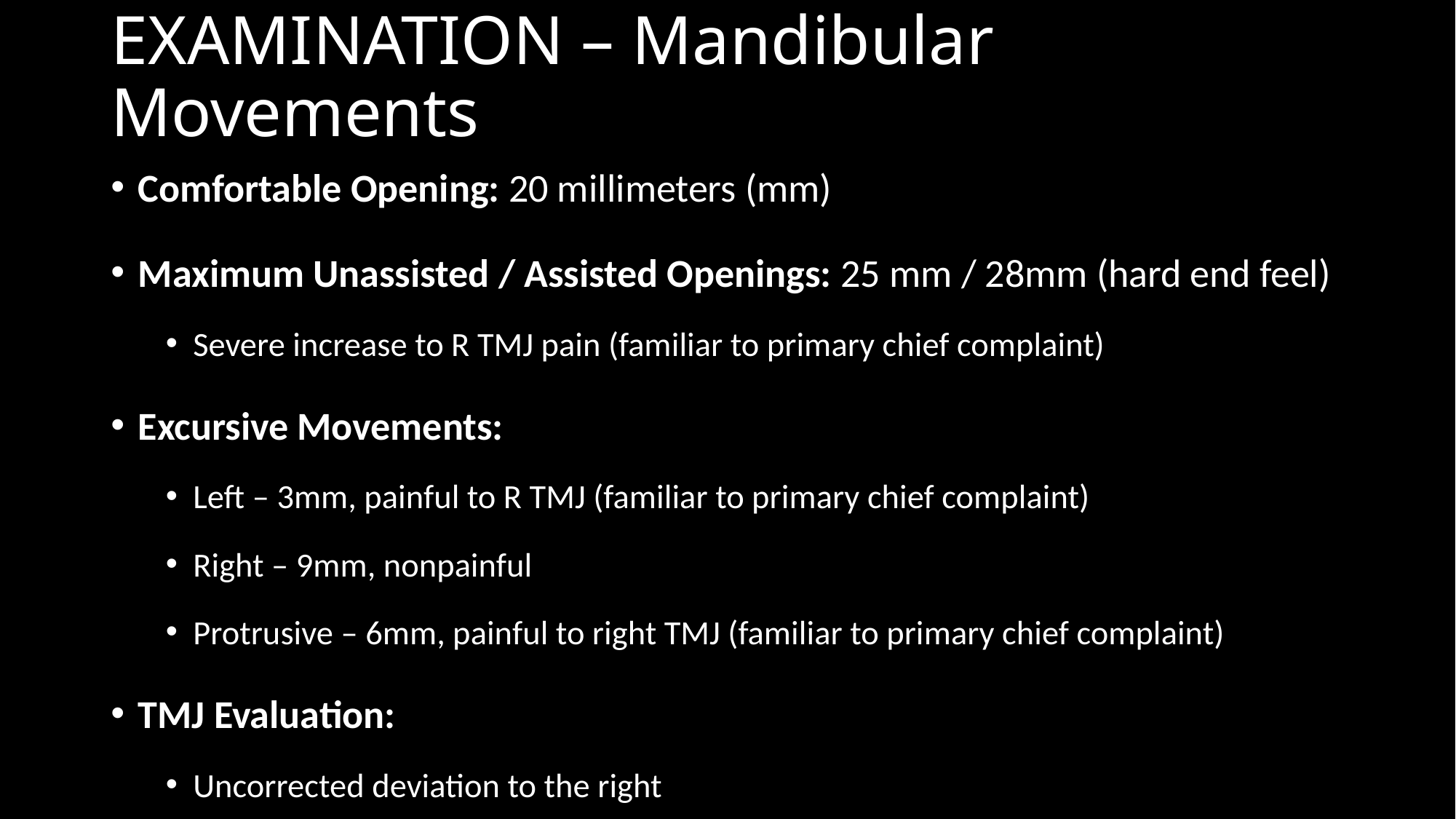

# EXAMINATION – Mandibular Movements
Comfortable Opening: 20 millimeters (mm)
Maximum Unassisted / Assisted Openings: 25 mm / 28mm (hard end feel)
Severe increase to R TMJ pain (familiar to primary chief complaint)
Excursive Movements:
Left – 3mm, painful to R TMJ (familiar to primary chief complaint)
Right – 9mm, nonpainful
Protrusive – 6mm, painful to right TMJ (familiar to primary chief complaint)
TMJ Evaluation:
Uncorrected deviation to the right

## Slide 46
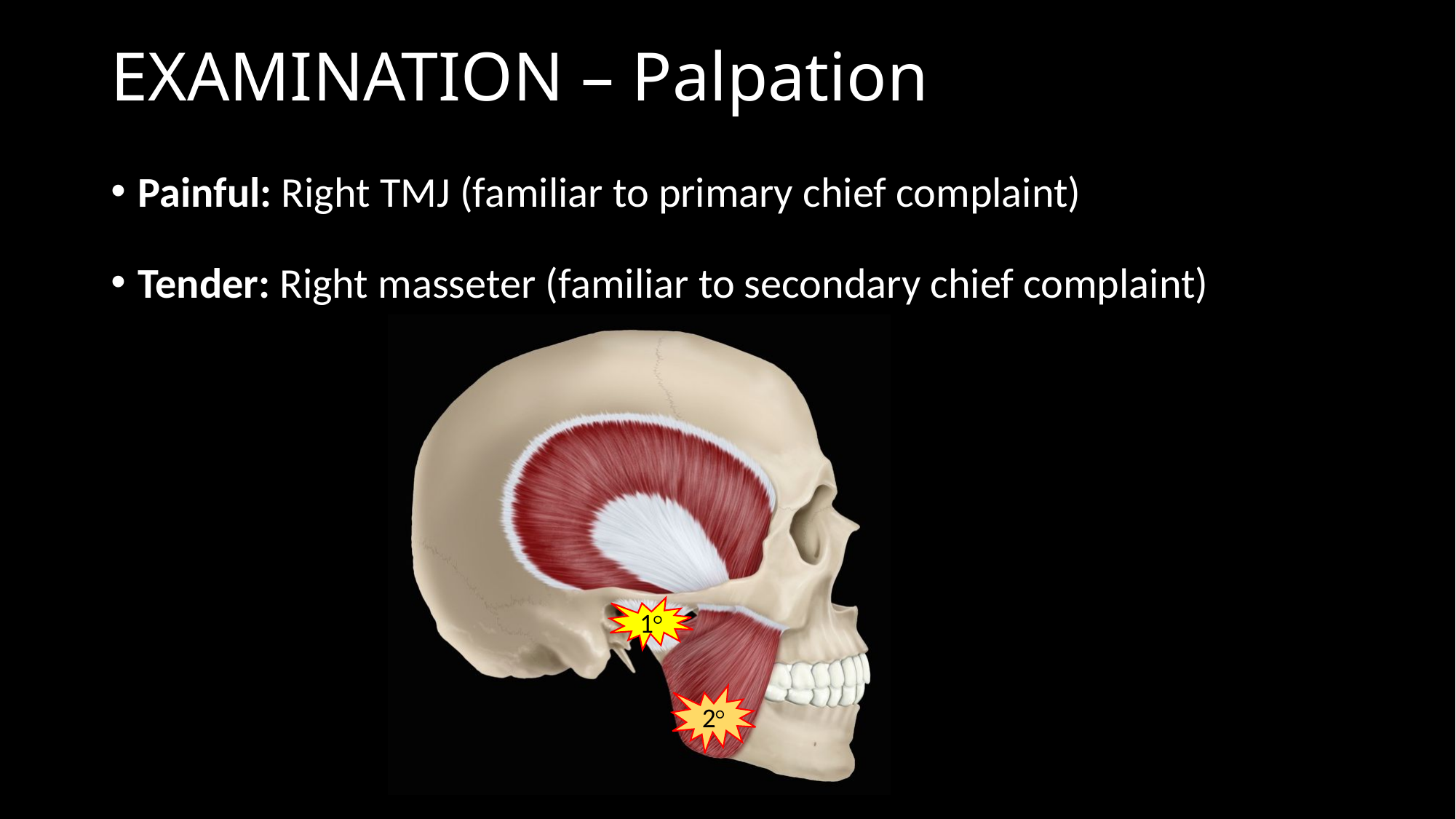

# EXAMINATION – Palpation
Painful: Right TMJ (familiar to primary chief complaint)
Tender: Right masseter (familiar to secondary chief complaint)
1○
2○

## Slide 47
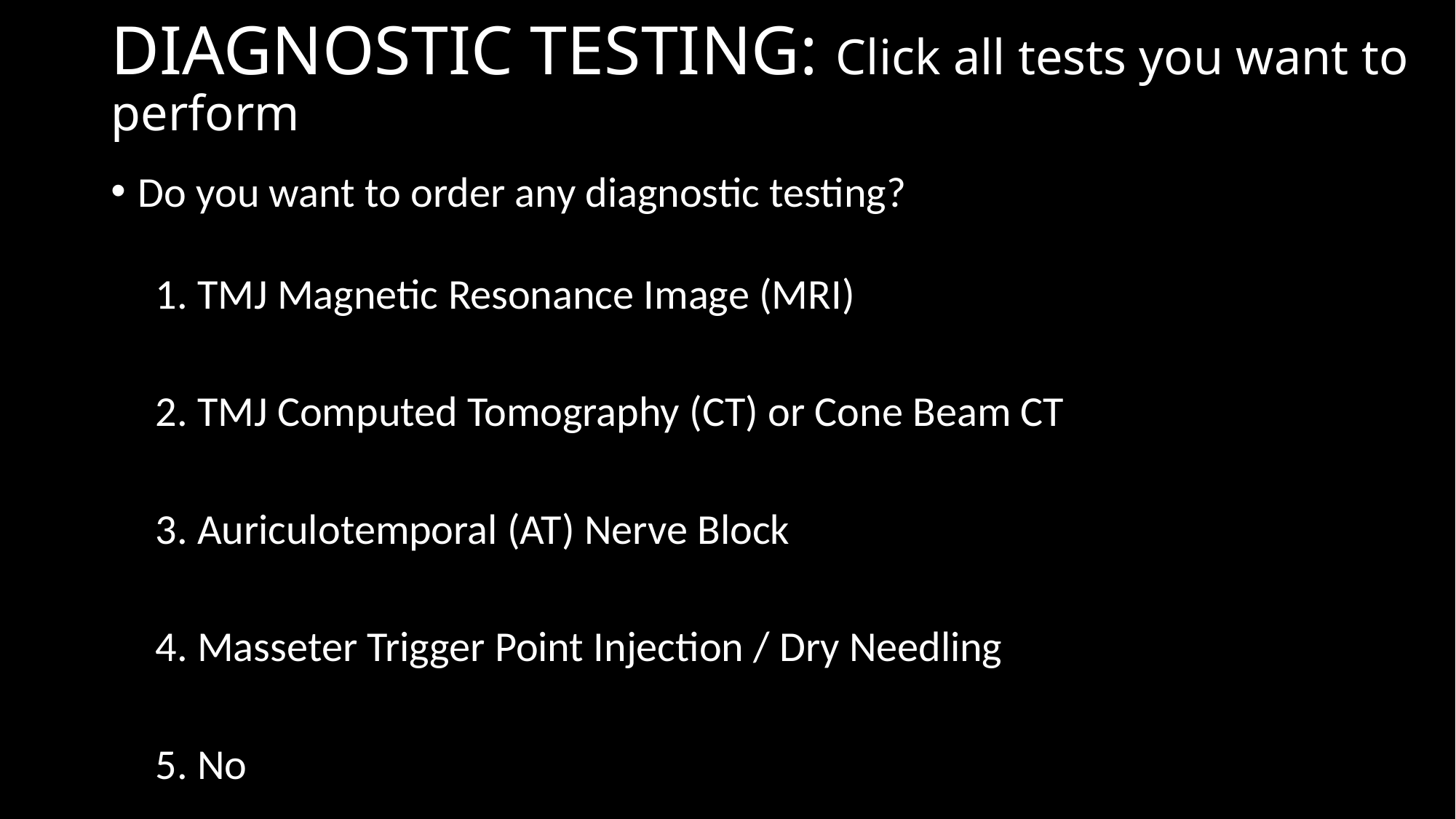

DIAGNOSTIC TESTING: Click all tests you want to perform
Do you want to order any diagnostic testing?
1. TMJ Magnetic Resonance Image (MRI)
2. TMJ Computed Tomography (CT) or Cone Beam CT
3. Auriculotemporal (AT) Nerve Block
4. Masseter Trigger Point Injection / Dry Needling
5. No

## Slide 48
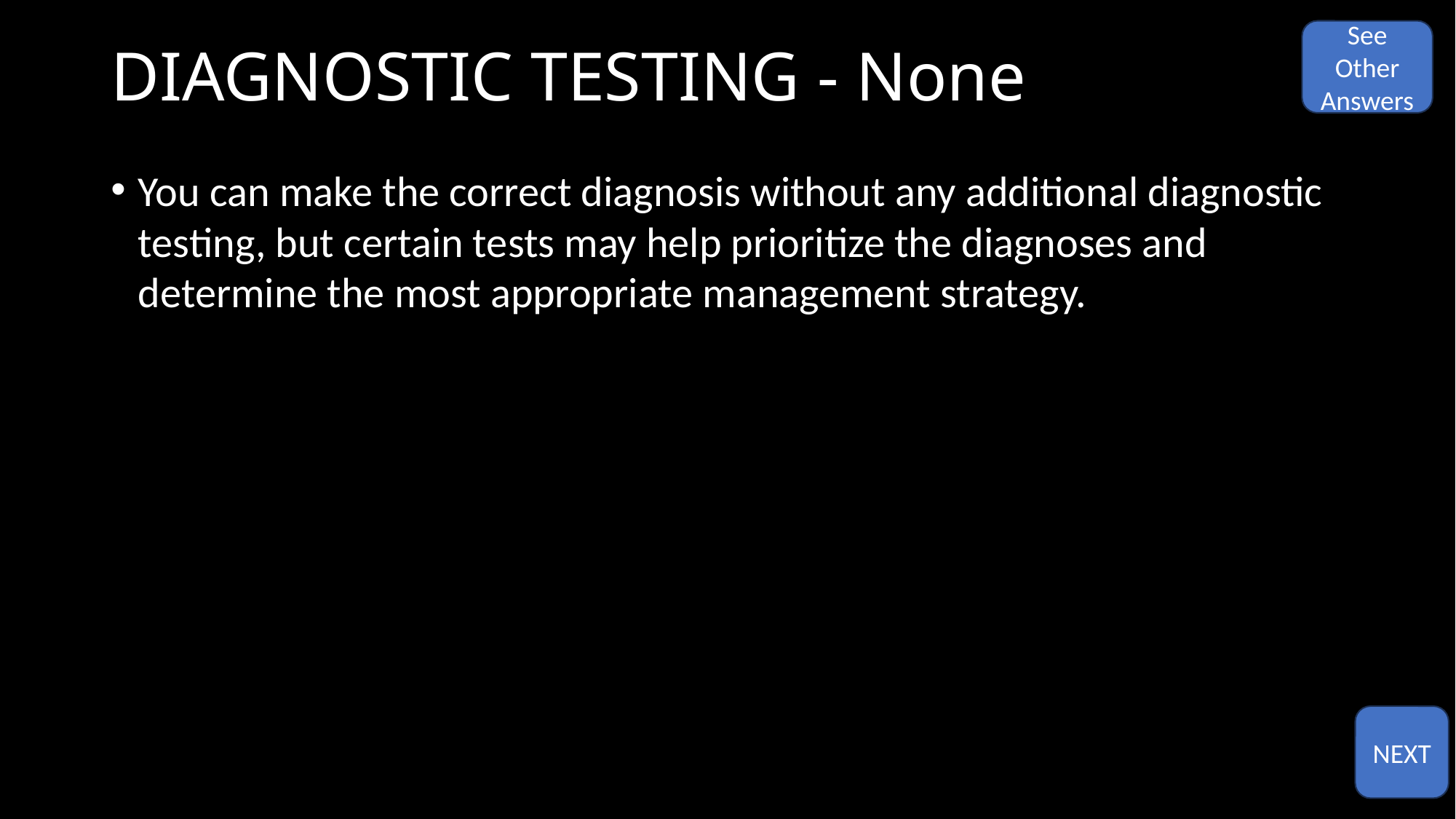

# DIAGNOSTIC TESTING - None
See Other Answers
You can make the correct diagnosis without any additional diagnostic testing, but certain tests may help prioritize the diagnoses and determine the most appropriate management strategy.
NEXT

## Slide 49
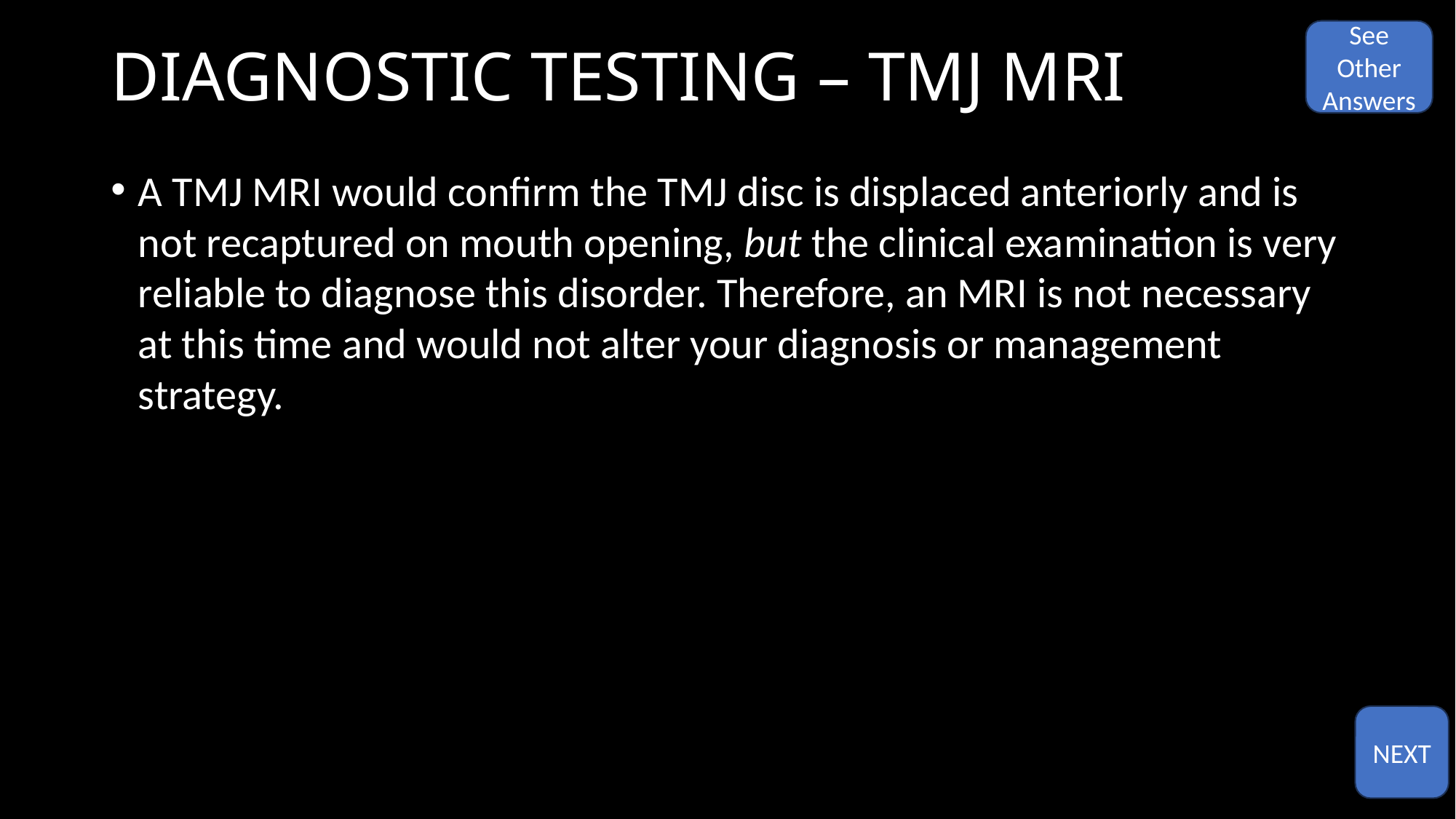

# DIAGNOSTIC TESTING – TMJ MRI
See Other Answers
A TMJ MRI would confirm the TMJ disc is displaced anteriorly and is not recaptured on mouth opening, but the clinical examination is very reliable to diagnose this disorder. Therefore, an MRI is not necessary at this time and would not alter your diagnosis or management strategy.
NEXT

## Slide 50
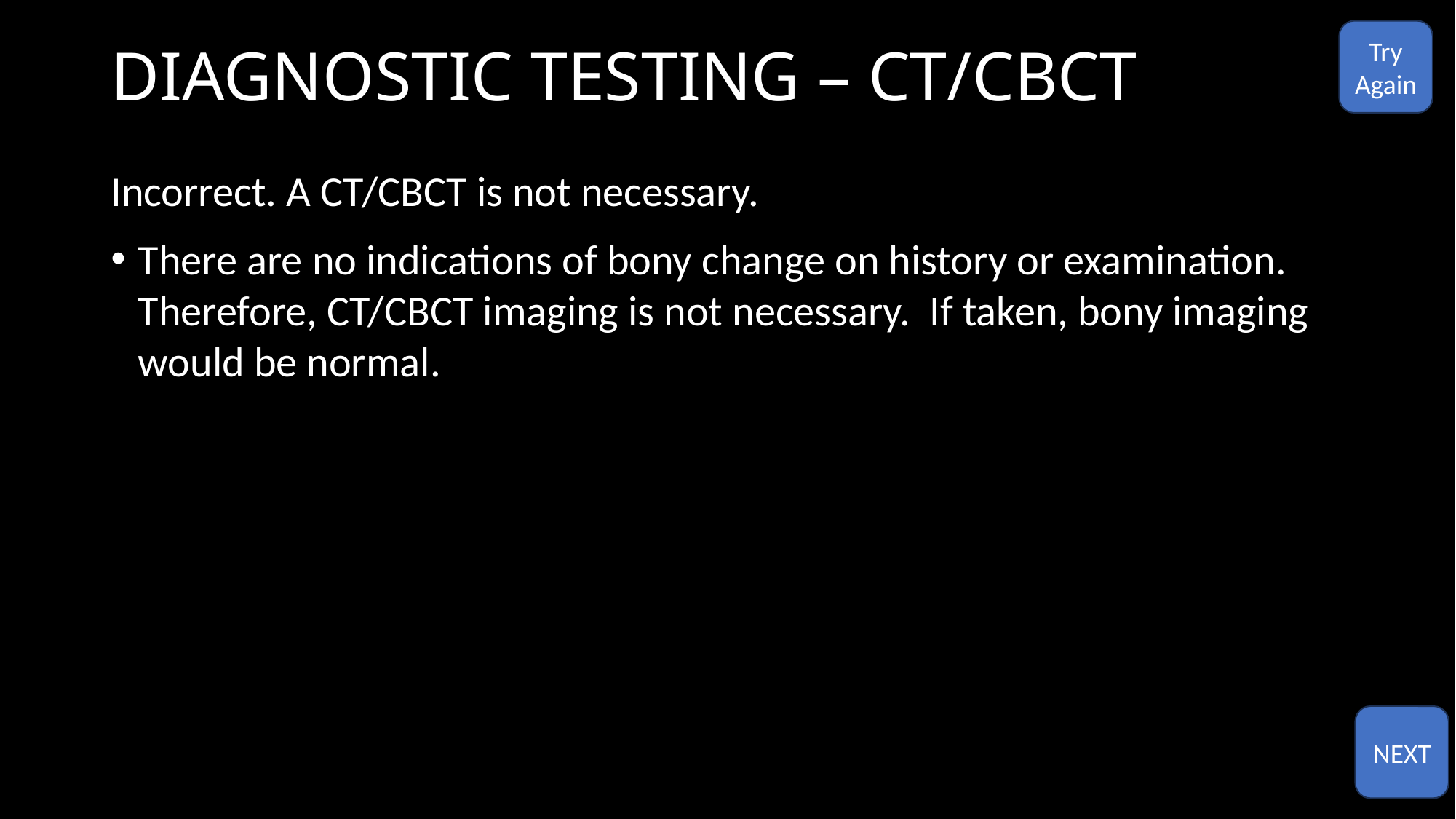

# DIAGNOSTIC TESTING – CT/CBCT
Try
Again
Incorrect. A CT/CBCT is not necessary.
There are no indications of bony change on history or examination. Therefore, CT/CBCT imaging is not necessary. If taken, bony imaging would be normal.
NEXT

## Slide 51
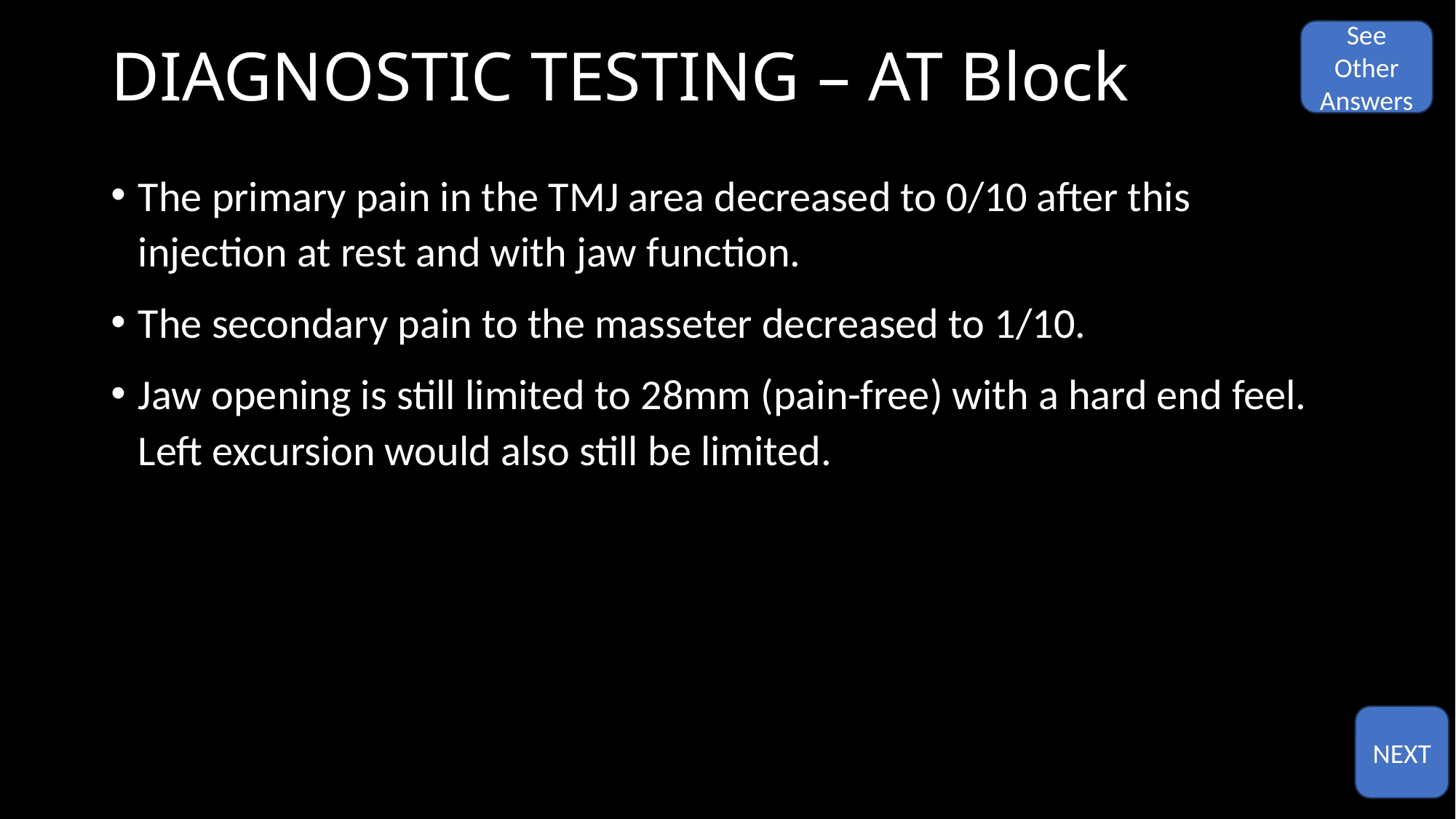

# DIAGNOSTIC TESTING – AT Block
See Other Answers
The primary pain in the TMJ area decreased to 0/10 after this injection at rest and with jaw function.
The secondary pain to the masseter decreased to 1/10.
Jaw opening is still limited to 28mm (pain-free) with a hard end feel. Left excursion would also still be limited.
NEXT

## Slide 52
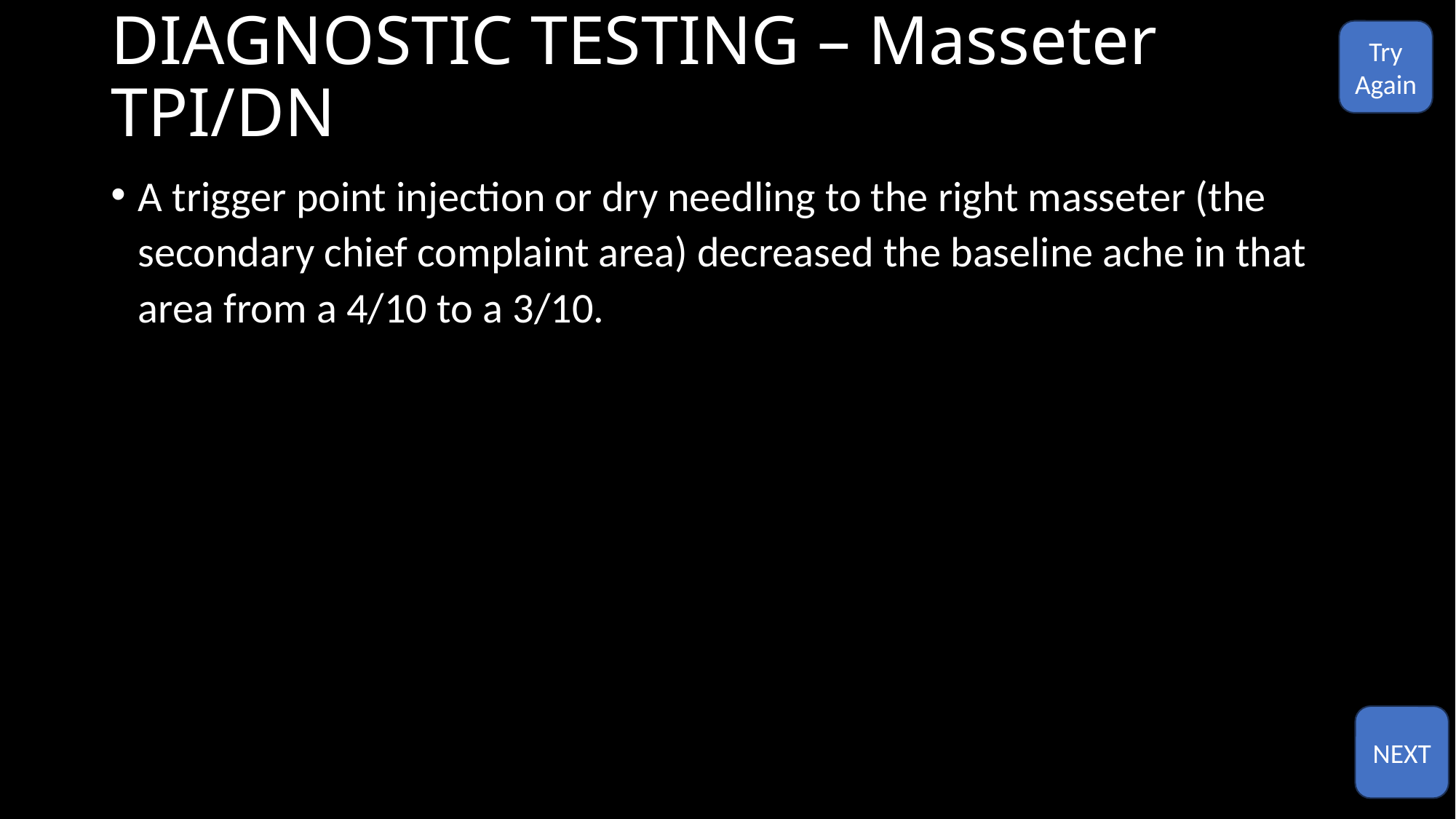

# DIAGNOSTIC TESTING – Masseter TPI/DN
Try
Again
A trigger point injection or dry needling to the right masseter (the secondary chief complaint area) decreased the baseline ache in that area from a 4/10 to a 3/10.
NEXT

## Slide 53
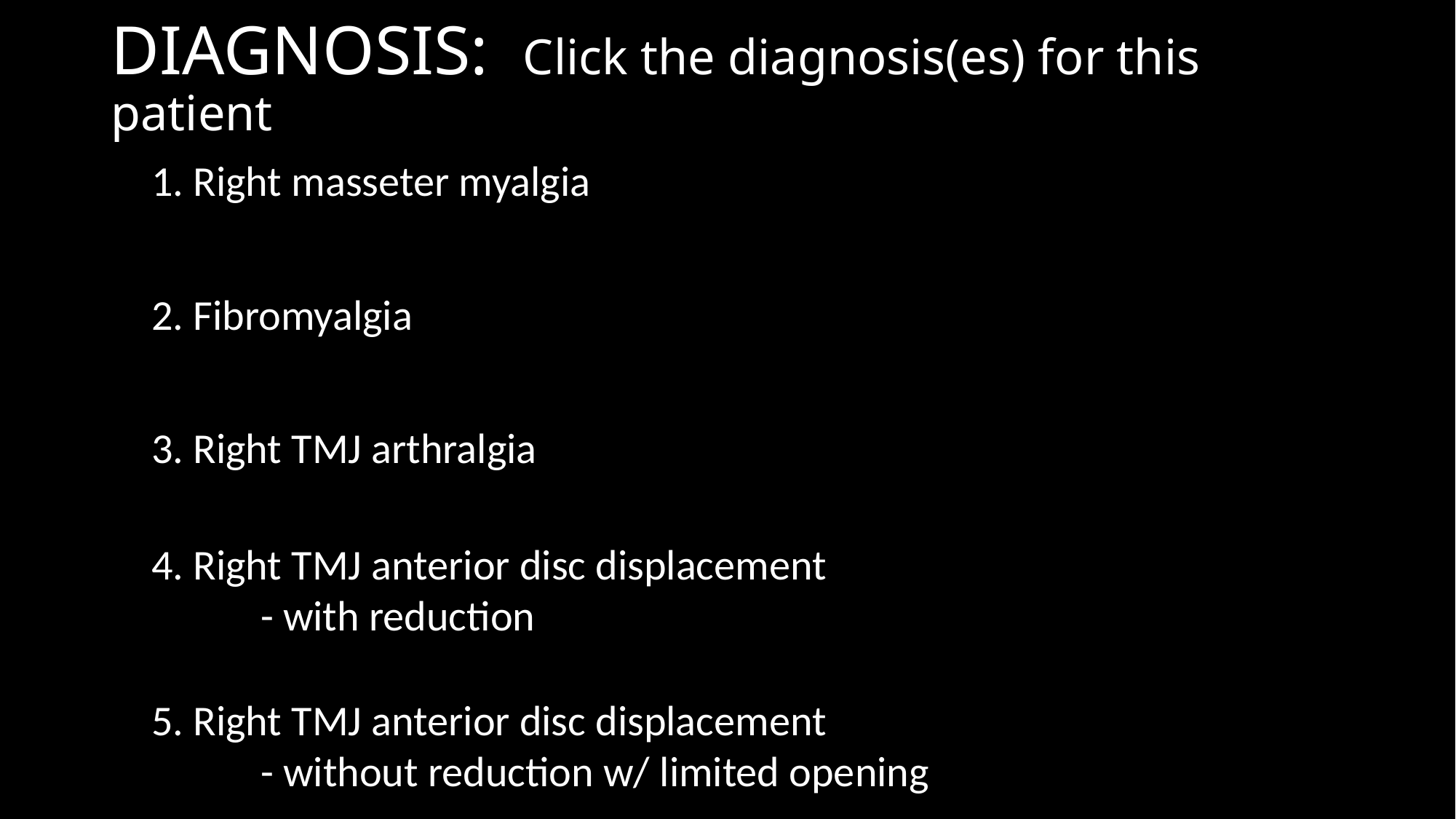

# DIAGNOSIS: Click the diagnosis(es) for this patient
1. Right masseter myalgia
2. Fibromyalgia
3. Right TMJ arthralgia
4. Right TMJ anterior disc displacement
	- with reduction
5. Right TMJ anterior disc displacement
	- without reduction w/ limited opening

## Slide 54
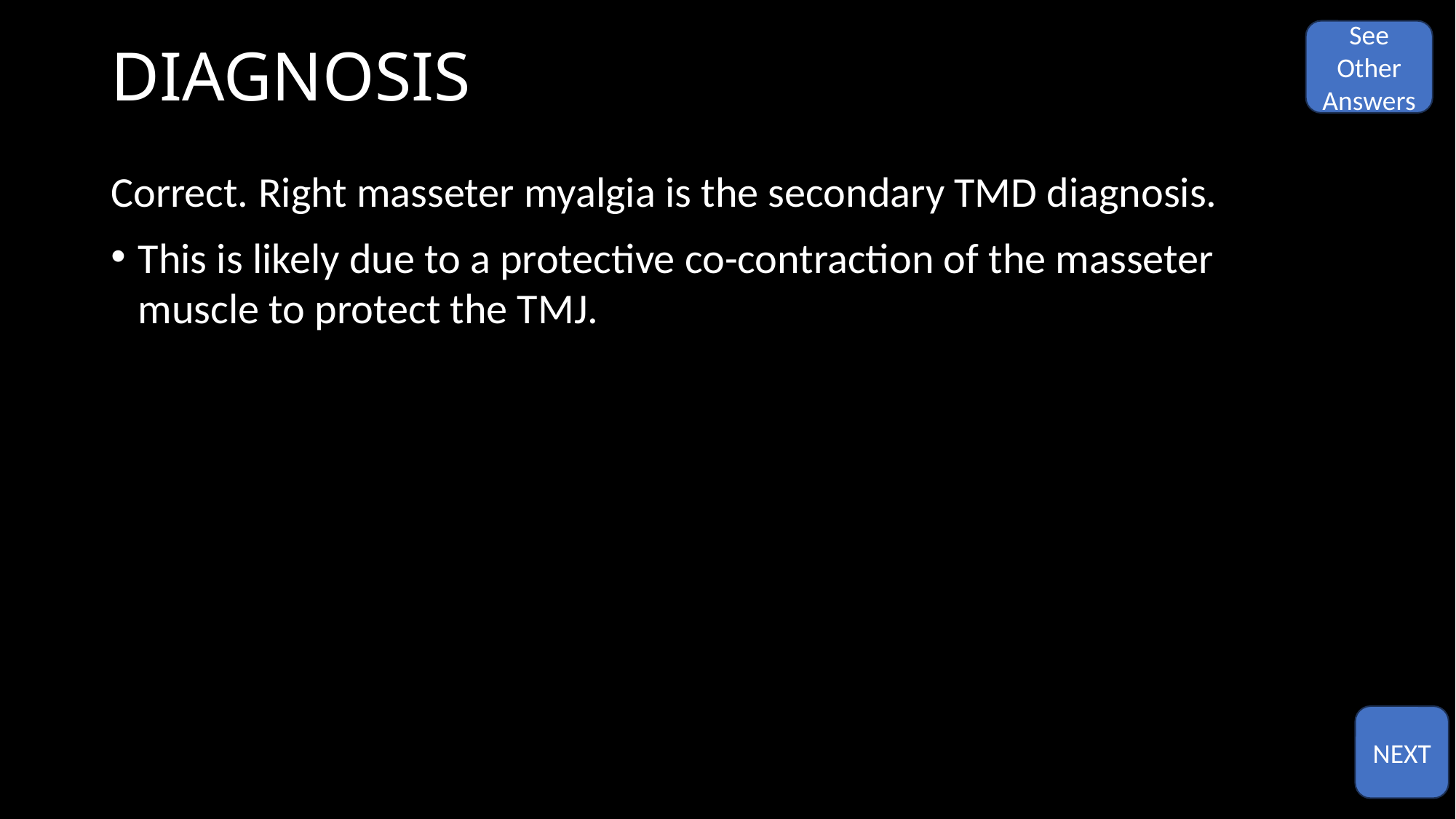

# DIAGNOSIS
See Other Answers
Correct. Right masseter myalgia is the secondary TMD diagnosis.
This is likely due to a protective co-contraction of the masseter muscle to protect the TMJ.
NEXT

## Slide 55
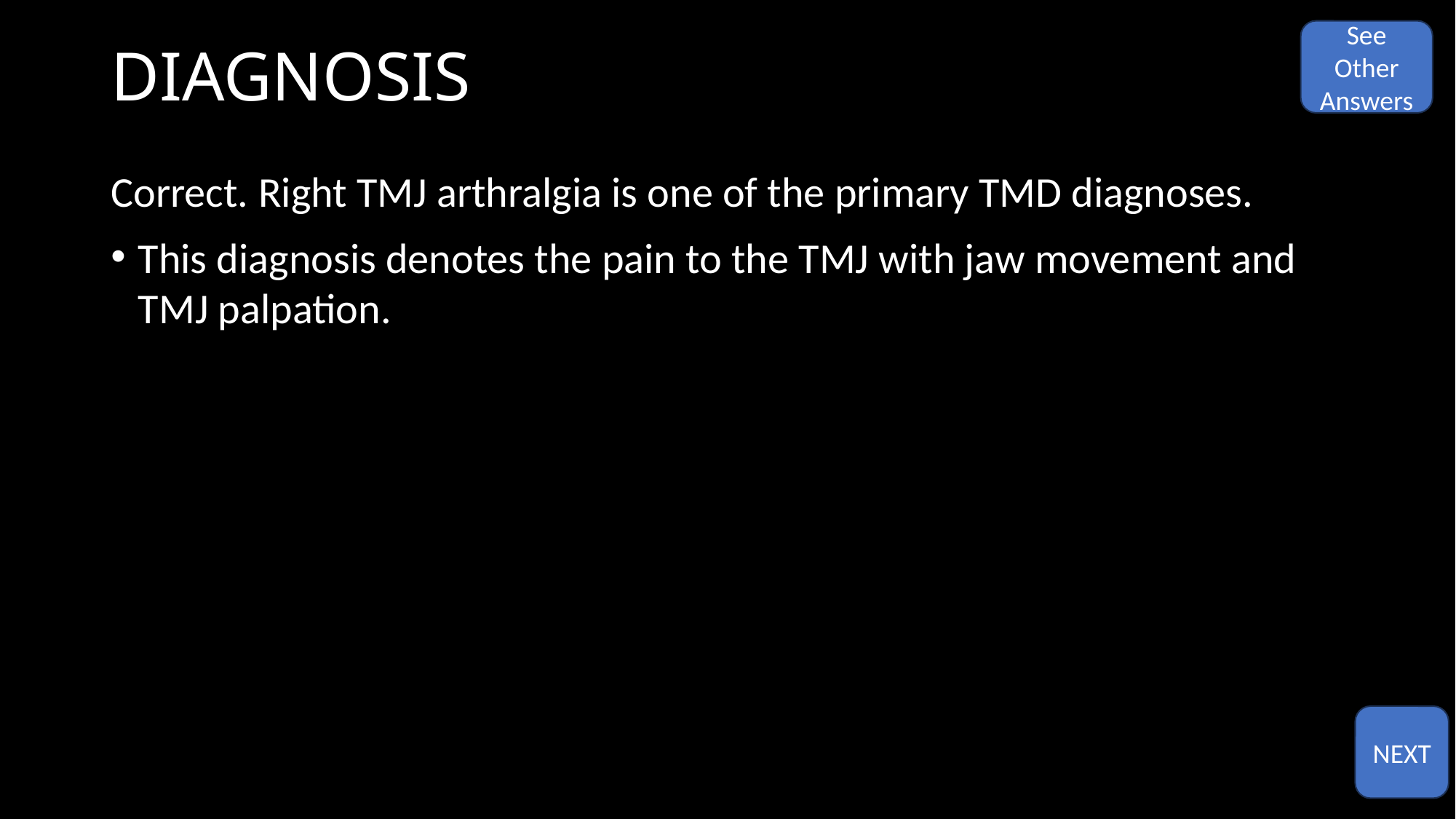

# DIAGNOSIS
See Other Answers
Correct. Right TMJ arthralgia is one of the primary TMD diagnoses.
This diagnosis denotes the pain to the TMJ with jaw movement and TMJ palpation.
NEXT

## Slide 56
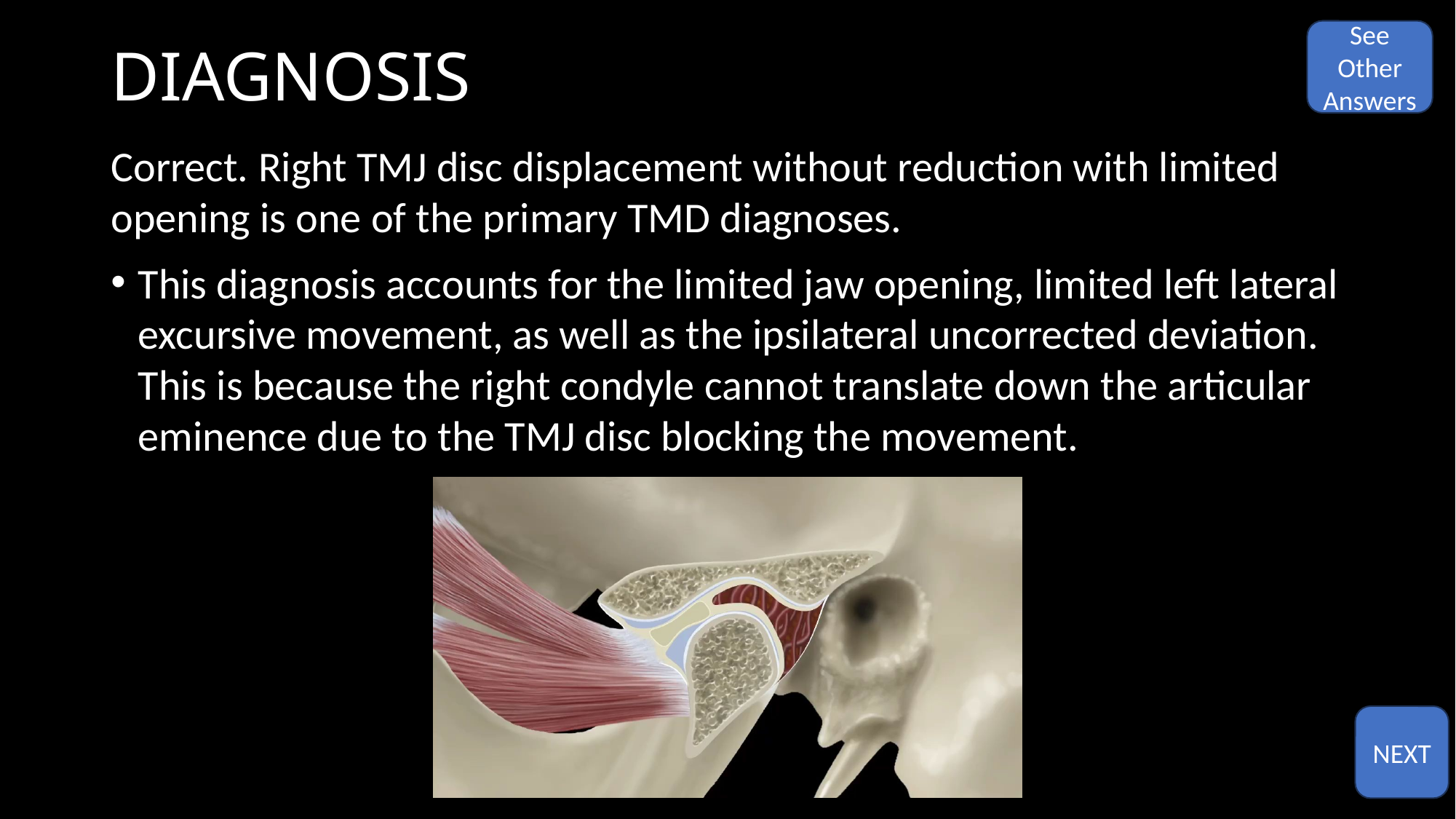

# DIAGNOSIS
See Other Answers
Correct. Right TMJ disc displacement without reduction with limited opening is one of the primary TMD diagnoses.
This diagnosis accounts for the limited jaw opening, limited left lateral excursive movement, as well as the ipsilateral uncorrected deviation. This is because the right condyle cannot translate down the articular eminence due to the TMJ disc blocking the movement.
NEXT

## Slide 57
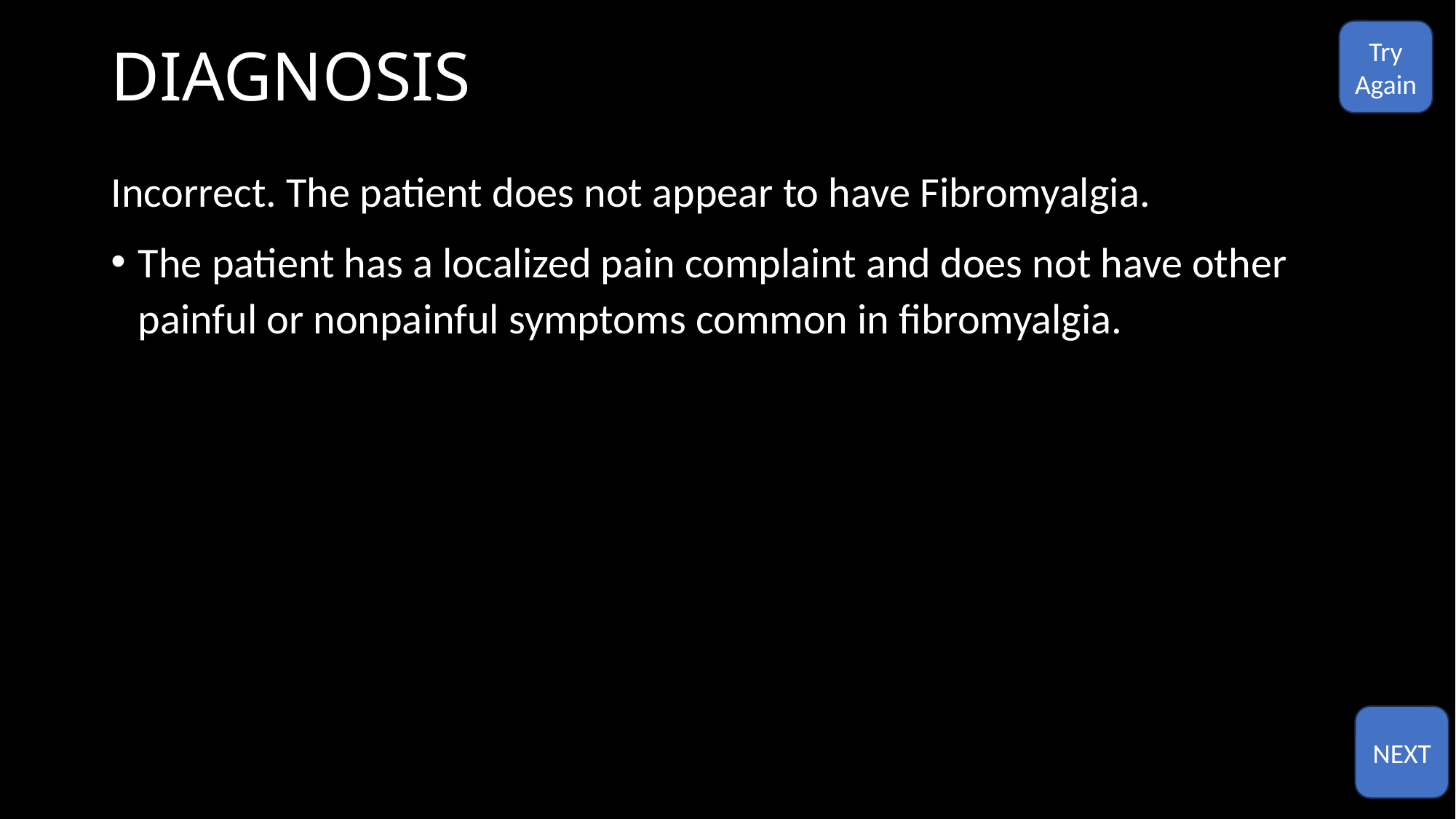

# DIAGNOSIS
Try
Again
Incorrect. The patient does not appear to have Fibromyalgia.
The patient has a localized pain complaint and does not have other painful or nonpainful symptoms common in fibromyalgia.
NEXT

## Slide 58
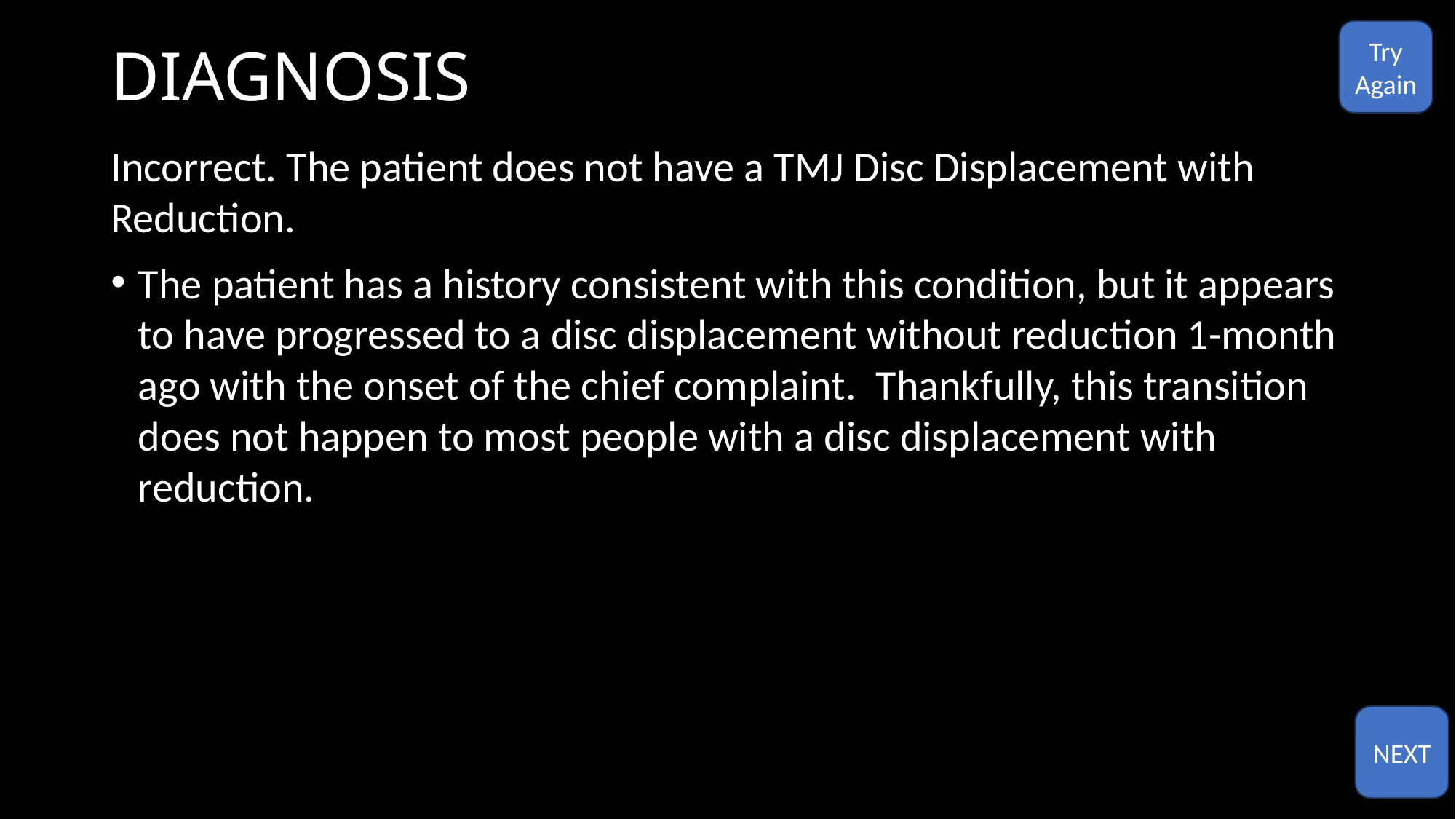

# DIAGNOSIS
Try
Again
Incorrect. The patient does not have a TMJ Disc Displacement with Reduction.
The patient has a history consistent with this condition, but it appears to have progressed to a disc displacement without reduction 1-month ago with the onset of the chief complaint. Thankfully, this transition does not happen to most people with a disc displacement with reduction.
NEXT

## Slide 59
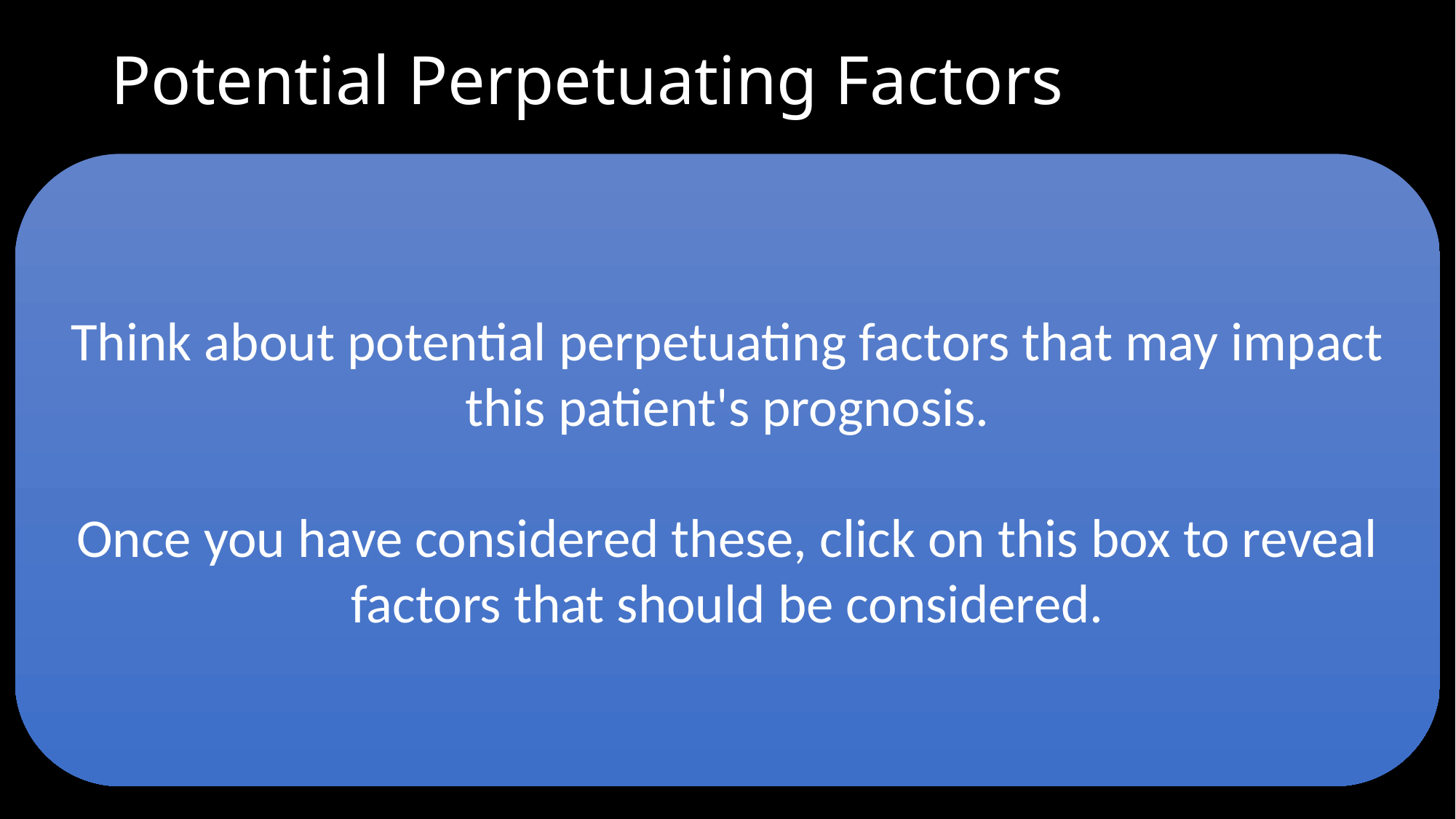

# Potential Perpetuating Factors
Oral Parafunction
Teeth together (especially when weightlifting)
Stimulants
Energy and Pre-workout drinks
Methylphenidate
Headache History
Prolonged history of frequent episodic tension type headache
Think about potential perpetuating factors that may impact this patient's prognosis.
Once you have considered these, click on this box to reveal factors that should be considered.

## Slide 60
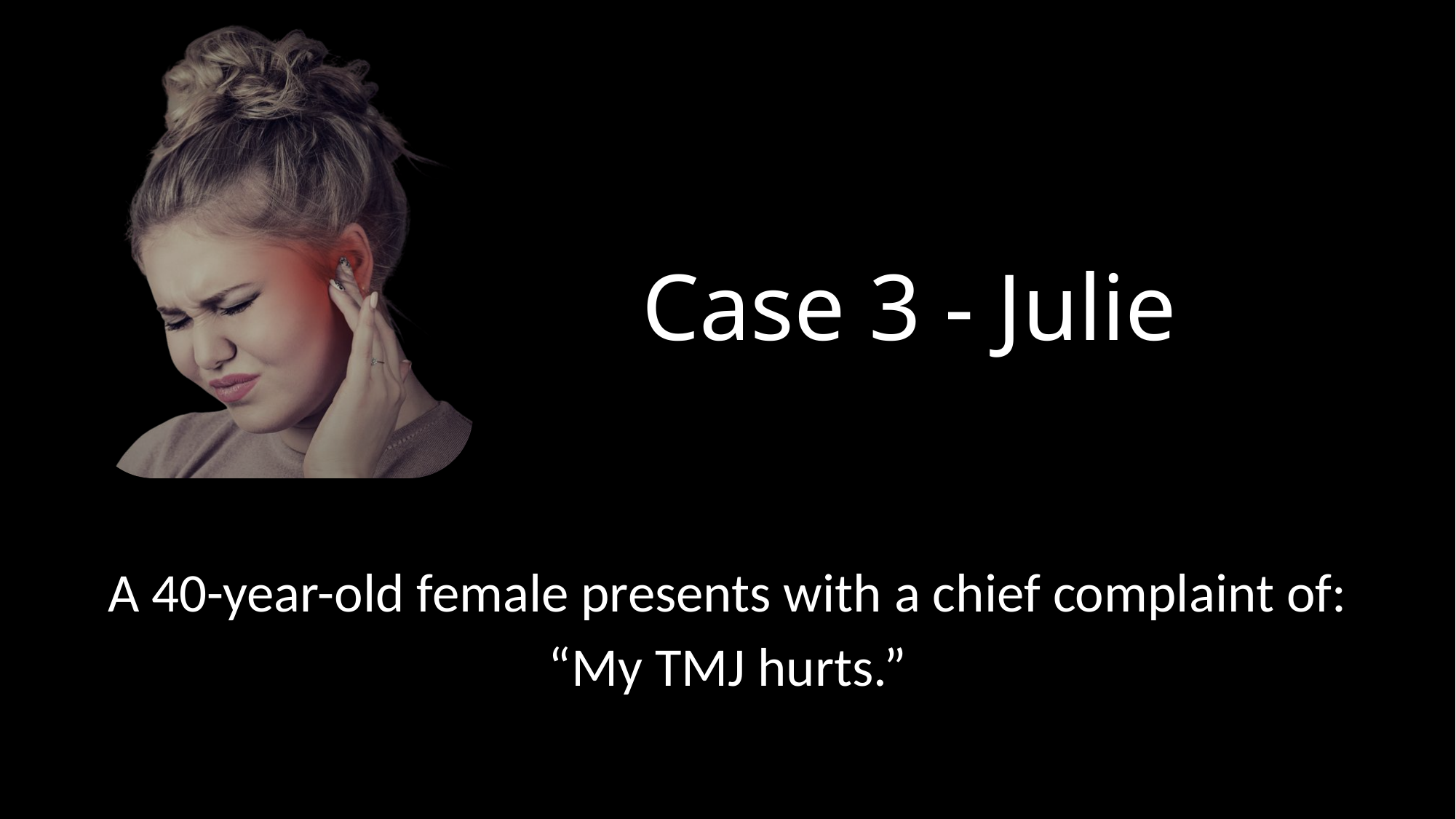

# Case 3 - Julie
A 40-year-old female presents with a chief complaint of:
“My TMJ hurts.”

## Slide 61
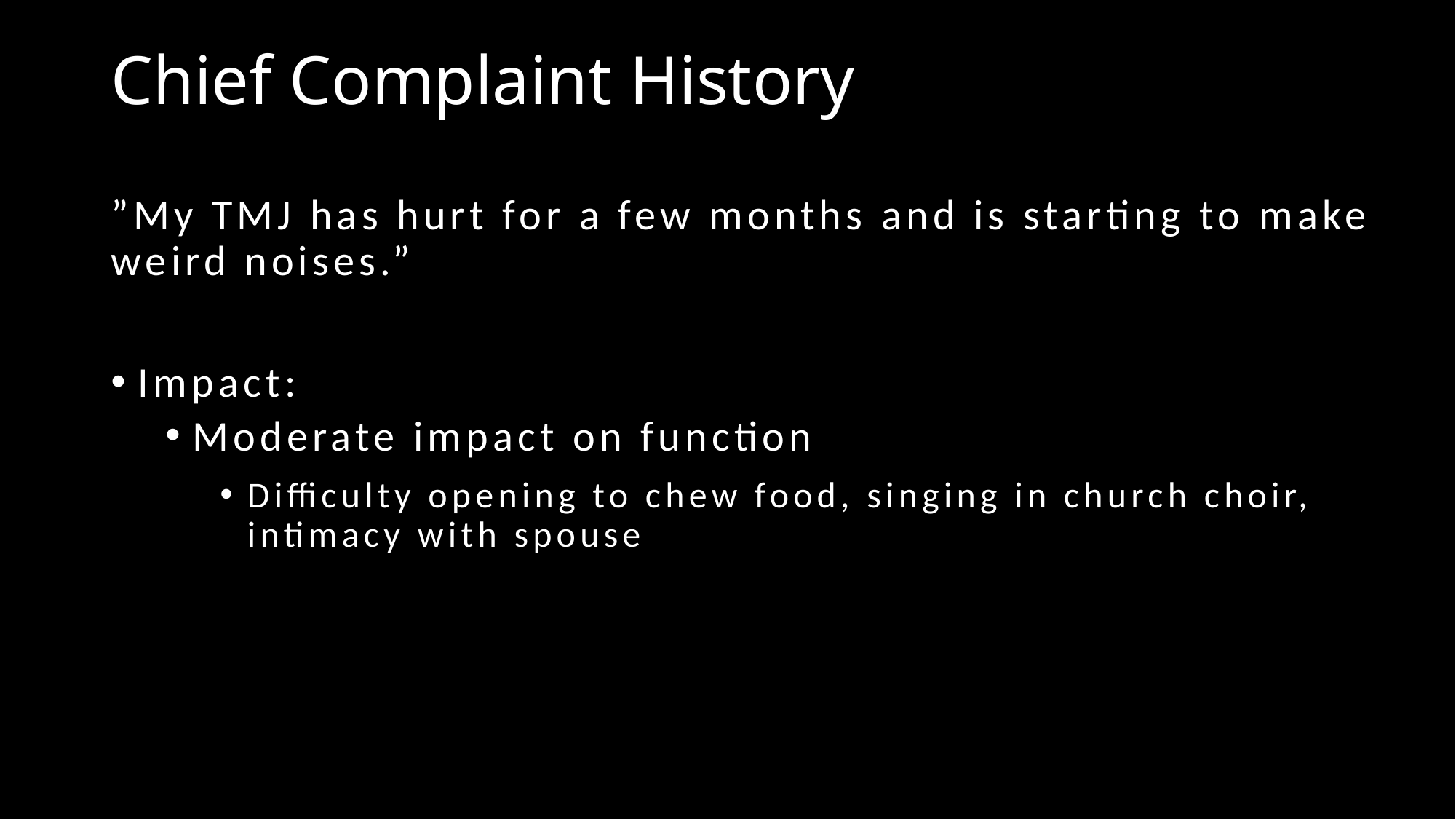

# Chief Complaint History
”My TMJ has hurt for a few months and is starting to make weird noises.”
Impact:
Moderate impact on function
Difficulty opening to chew food, singing in church choir, intimacy with spouse

## Slide 62
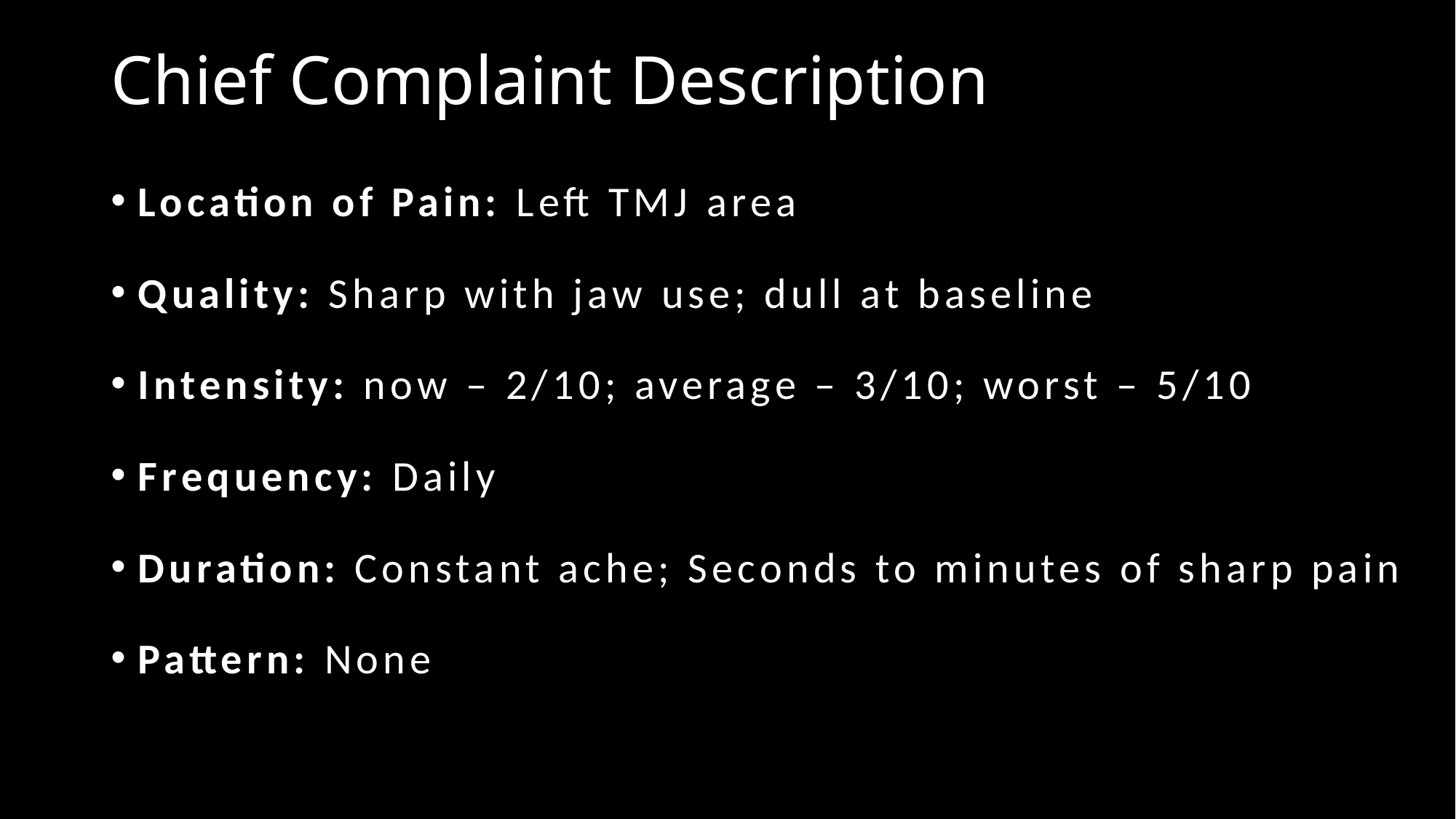

# Chief Complaint Description
Location of Pain: Left TMJ area
Quality: Sharp with jaw use; dull at baseline
Intensity: now – 2/10; average – 3/10; worst – 5/10
Frequency: Daily
Duration: Constant ache; Seconds to minutes of sharp pain
Pattern: None

## Slide 63
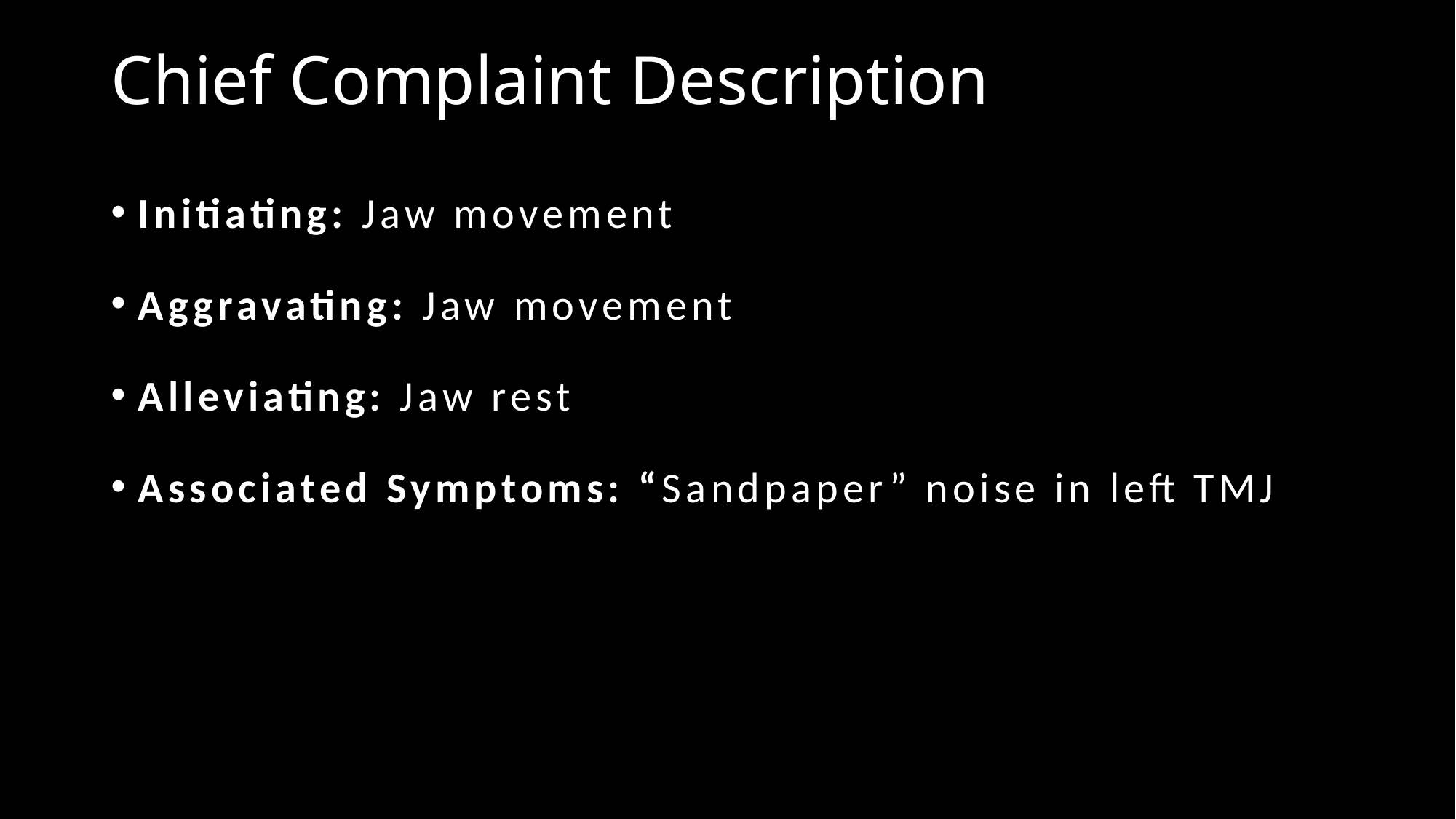

# Chief Complaint Description
Initiating: Jaw movement
Aggravating: Jaw movement
Alleviating: Jaw rest
Associated Symptoms: “Sandpaper” noise in left TMJ

## Slide 64
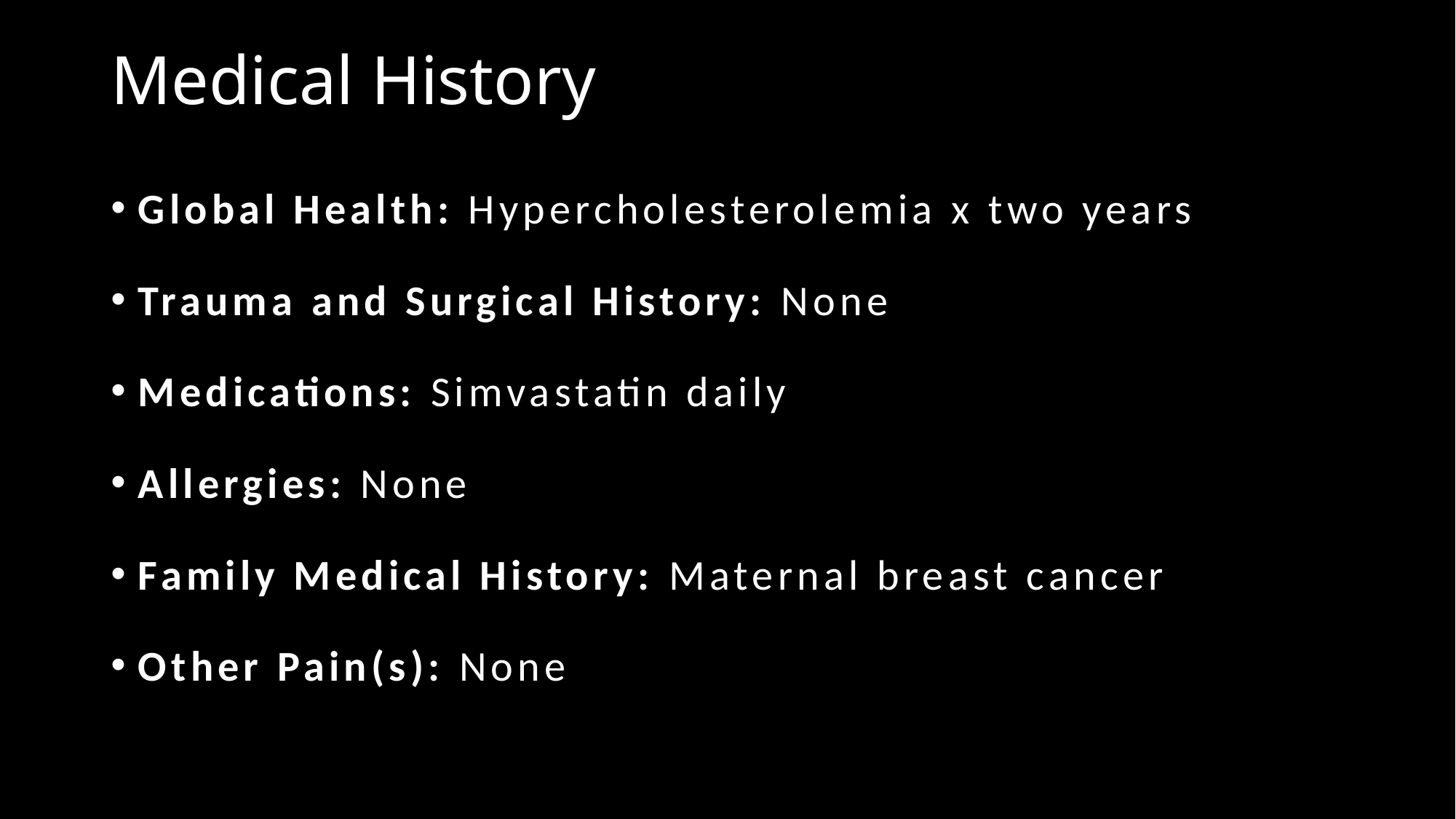

# Medical History
Global Health: Hypercholesterolemia x two years
Trauma and Surgical History: None
Medications: Simvastatin daily
Allergies: None
Family Medical History: Maternal breast cancer
Other Pain(s): None

## Slide 65
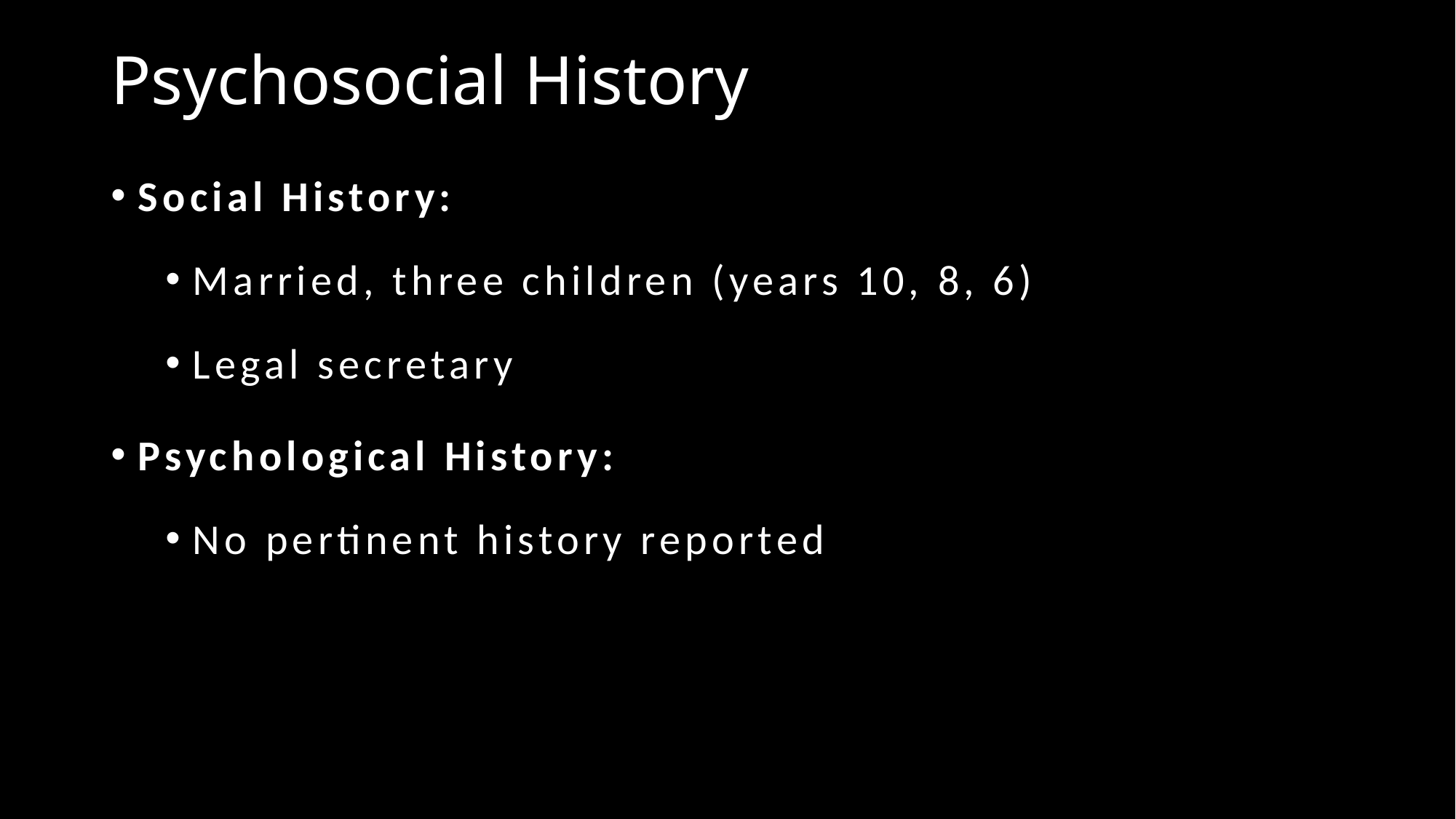

# Psychosocial History
Social History:
Married, three children (years 10, 8, 6)
Legal secretary
Psychological History:
No pertinent history reported

## Slide 66
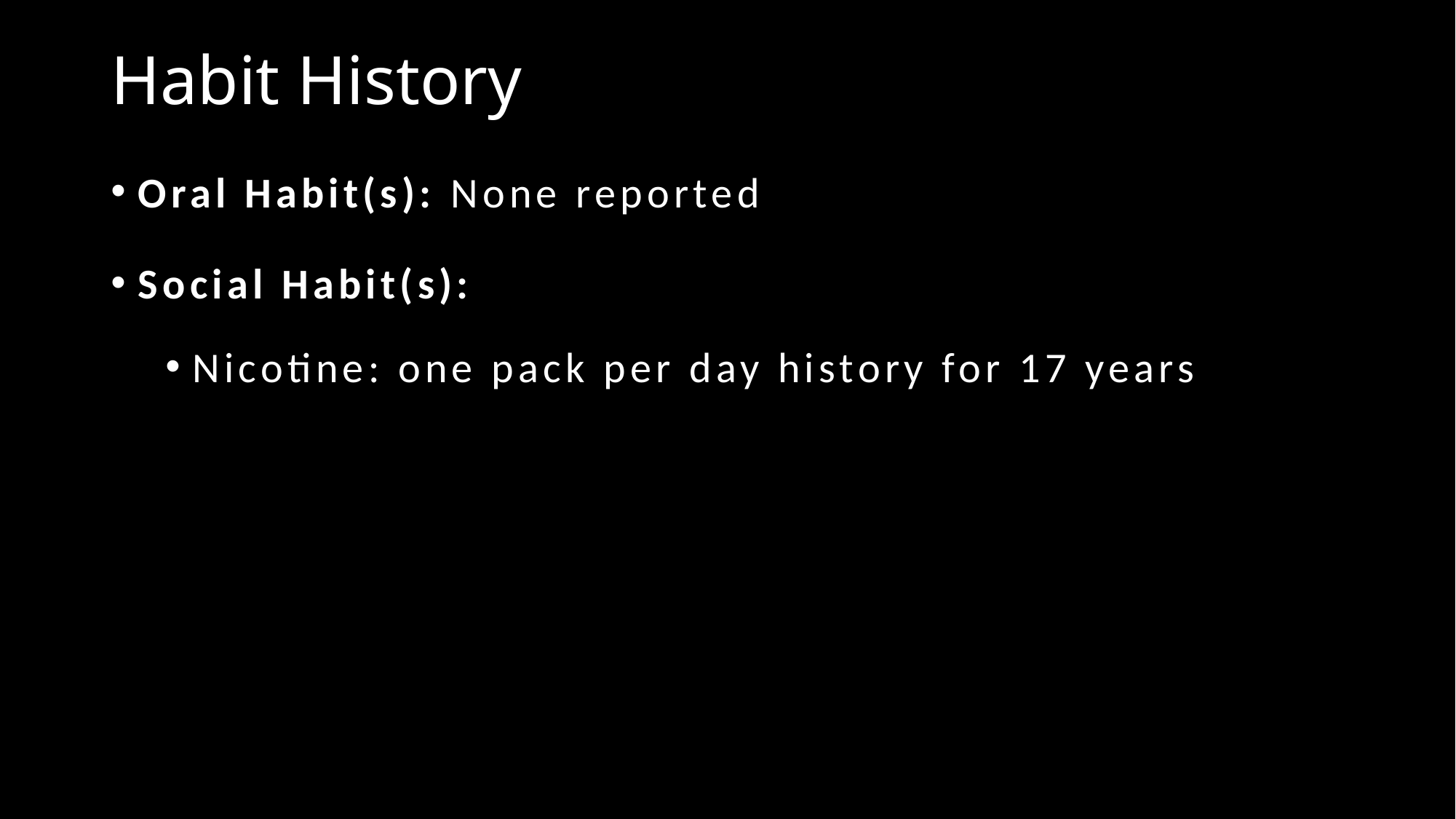

# Habit History
Oral Habit(s): None reported
Social Habit(s):
Nicotine: one pack per day history for 17 years

## Slide 67
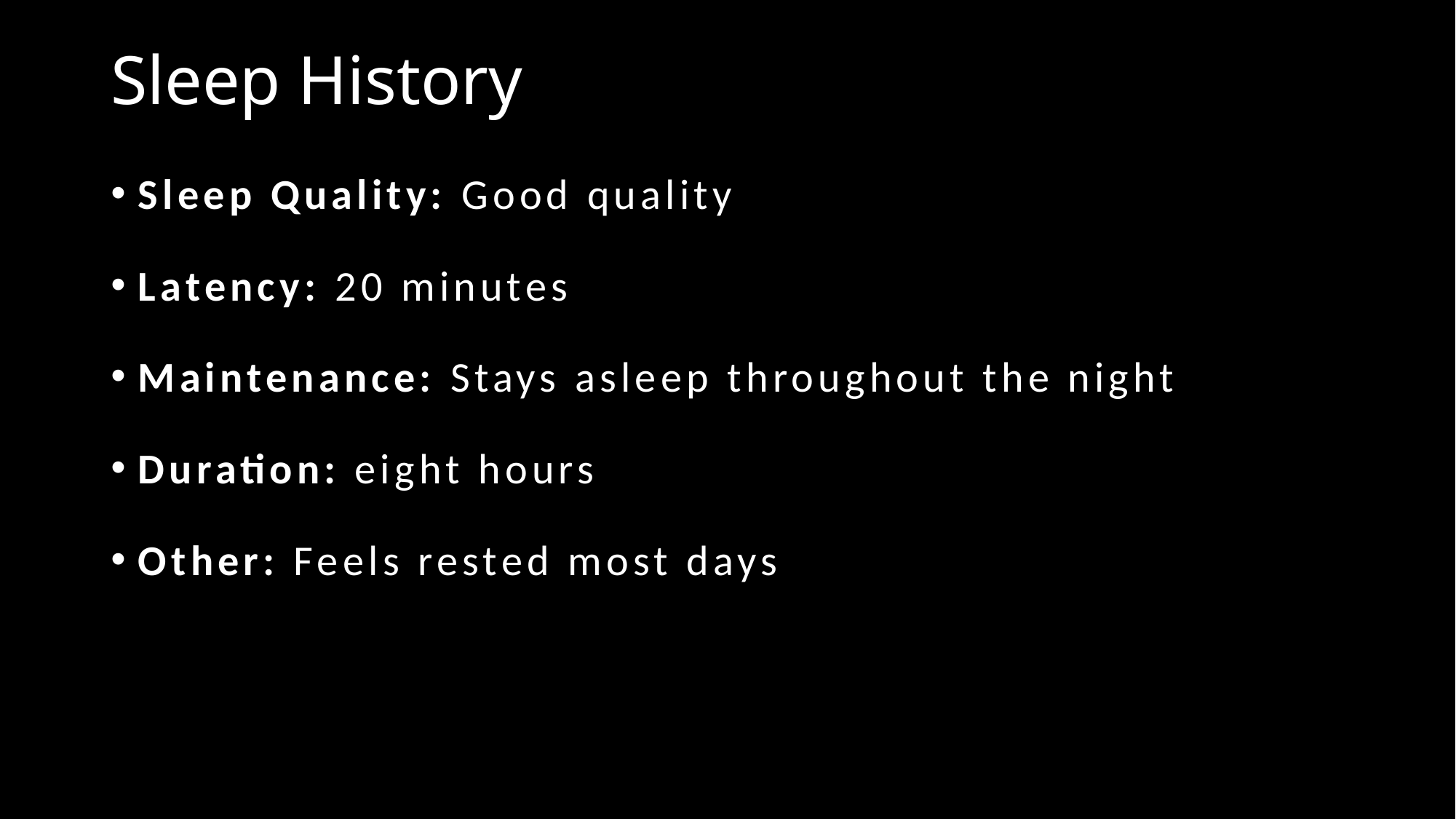

# Sleep History
Sleep Quality: Good quality
Latency: 20 minutes
Maintenance: Stays asleep throughout the night
Duration: eight hours
Other: Feels rested most days

## Slide 68
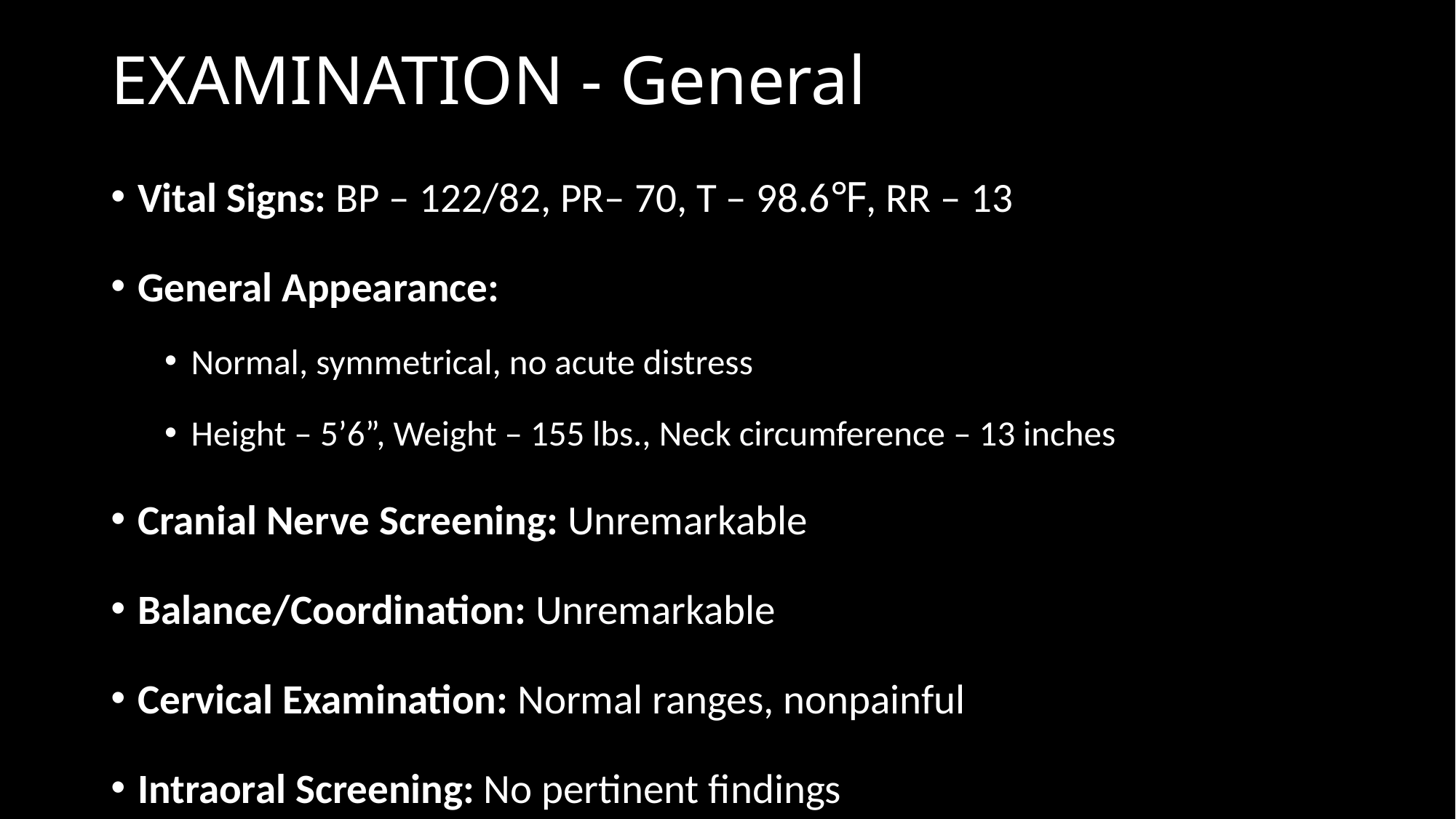

# EXAMINATION - General
Vital Signs: BP – 122/82, PR– 70, T – 98.6℉, RR – 13
General Appearance:
Normal, symmetrical, no acute distress
Height – 5’6”, Weight – 155 lbs., Neck circumference – 13 inches
Cranial Nerve Screening: Unremarkable
Balance/Coordination: Unremarkable
Cervical Examination: Normal ranges, nonpainful
Intraoral Screening: No pertinent findings

## Slide 69
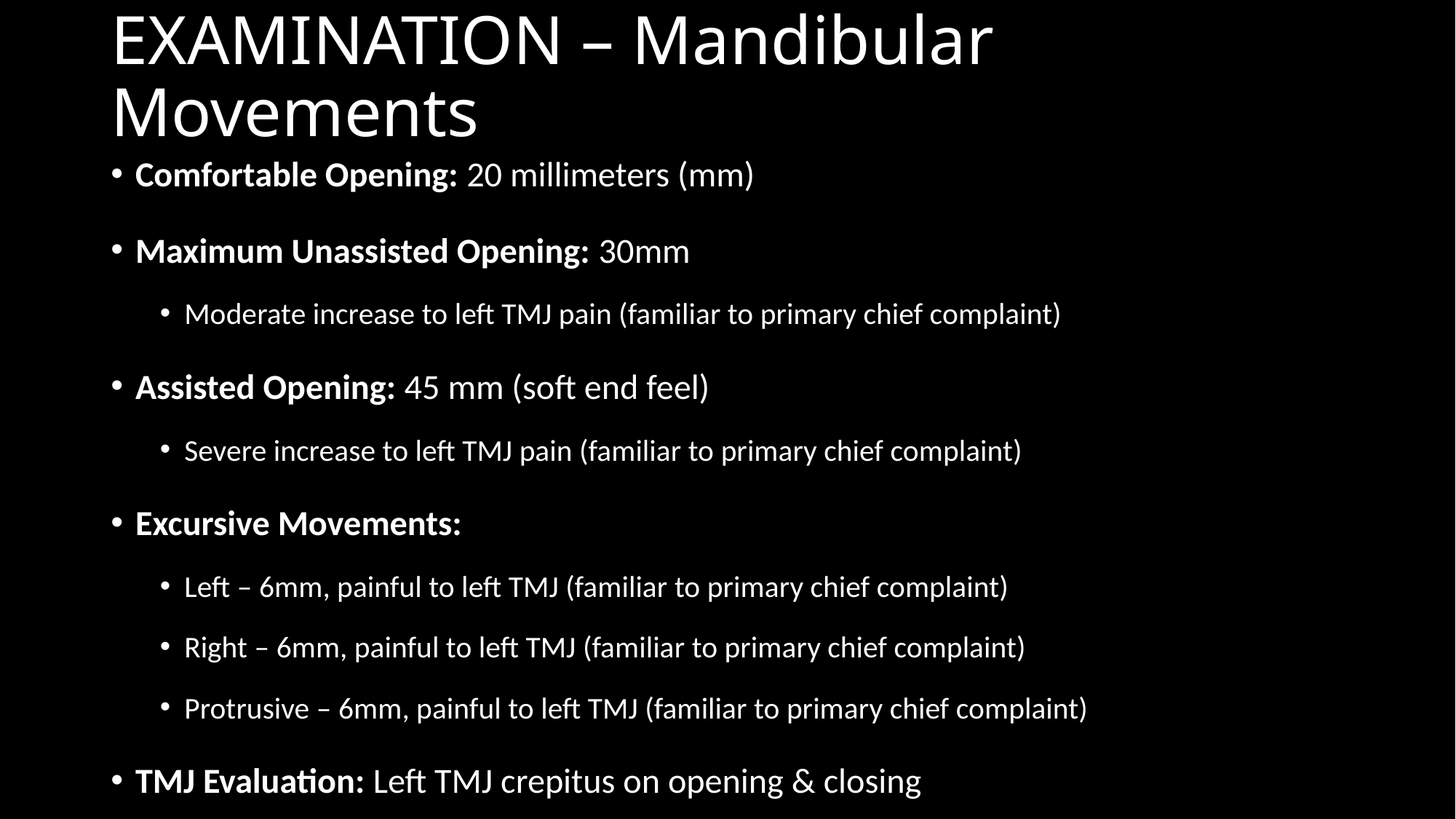

# EXAMINATION – Mandibular Movements
Comfortable Opening: 20 millimeters (mm)
Maximum Unassisted Opening: 30mm
Moderate increase to left TMJ pain (familiar to primary chief complaint)
Assisted Opening: 45 mm (soft end feel)
Severe increase to left TMJ pain (familiar to primary chief complaint)
Excursive Movements:
Left – 6mm, painful to left TMJ (familiar to primary chief complaint)
Right – 6mm, painful to left TMJ (familiar to primary chief complaint)
Protrusive – 6mm, painful to left TMJ (familiar to primary chief complaint)
TMJ Evaluation: Left TMJ crepitus on opening & closing

## Slide 70
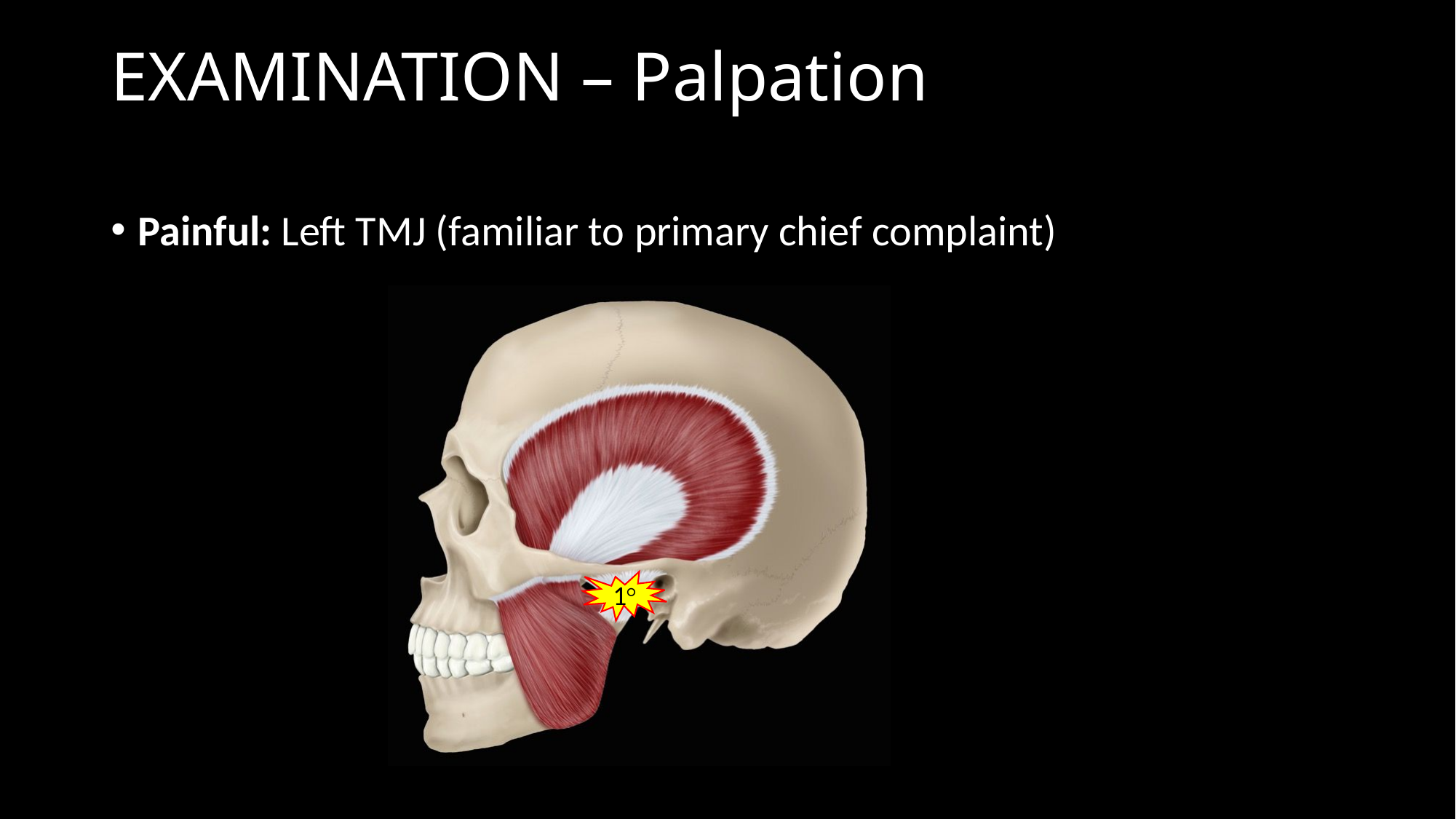

# EXAMINATION – Palpation
Painful: Left TMJ (familiar to primary chief complaint)
1○

## Slide 71
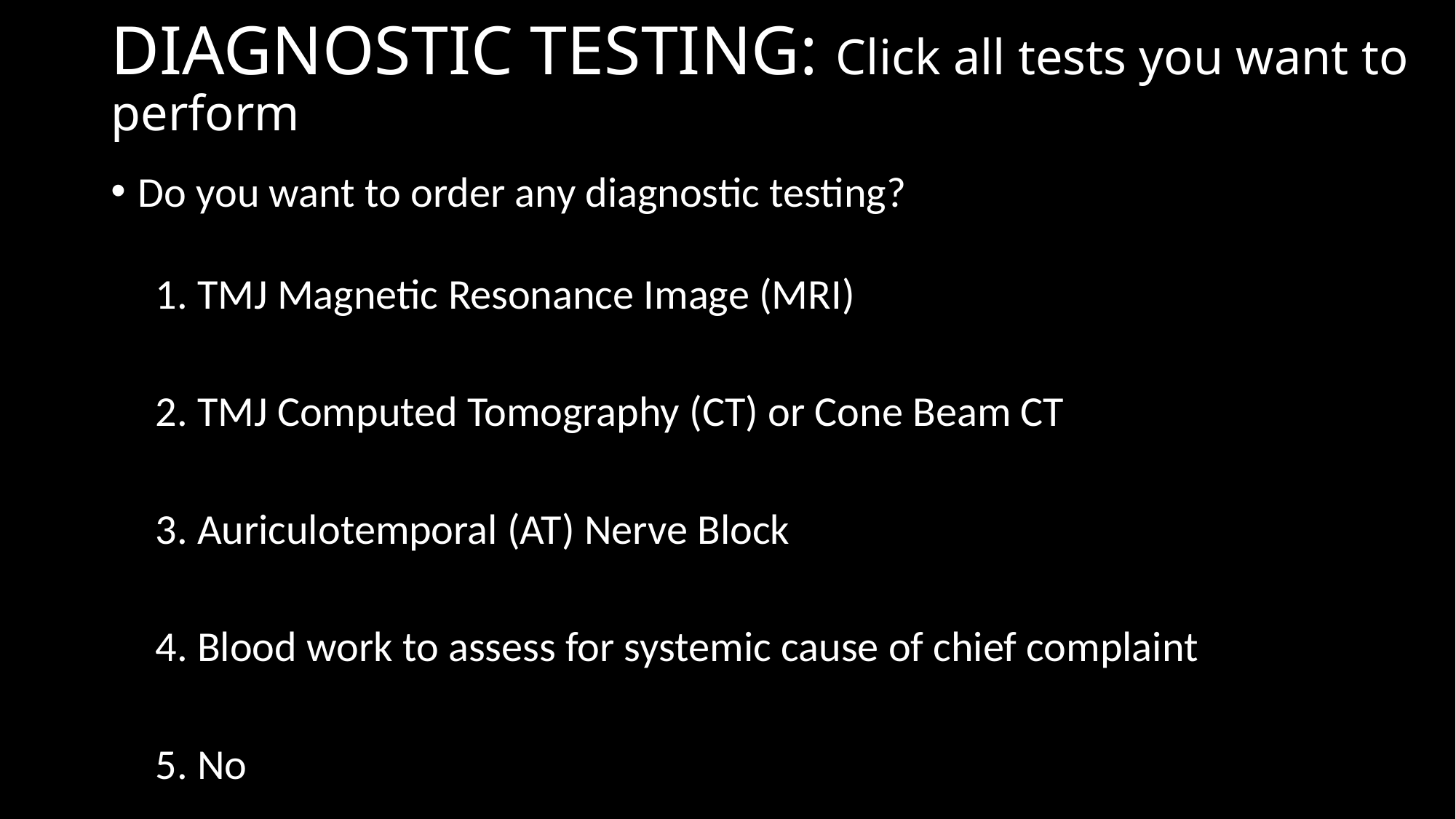

DIAGNOSTIC TESTING: Click all tests you want to perform
Do you want to order any diagnostic testing?
1. TMJ Magnetic Resonance Image (MRI)
2. TMJ Computed Tomography (CT) or Cone Beam CT
3. Auriculotemporal (AT) Nerve Block
4. Blood work to assess for systemic cause of chief complaint
5. No

## Slide 72
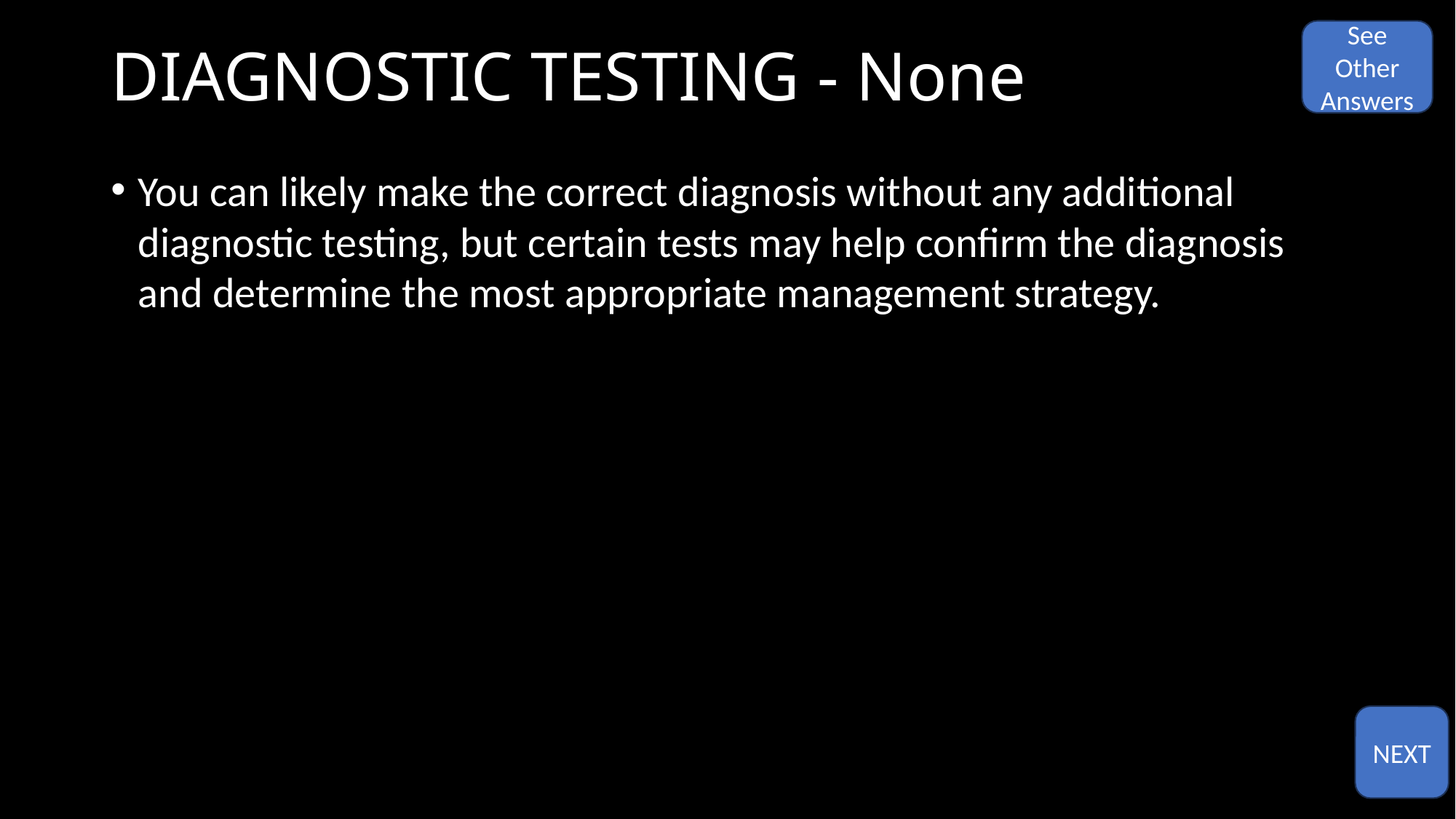

# DIAGNOSTIC TESTING - None
See Other Answers
You can likely make the correct diagnosis without any additional diagnostic testing, but certain tests may help confirm the diagnosis and determine the most appropriate management strategy.
NEXT

## Slide 73
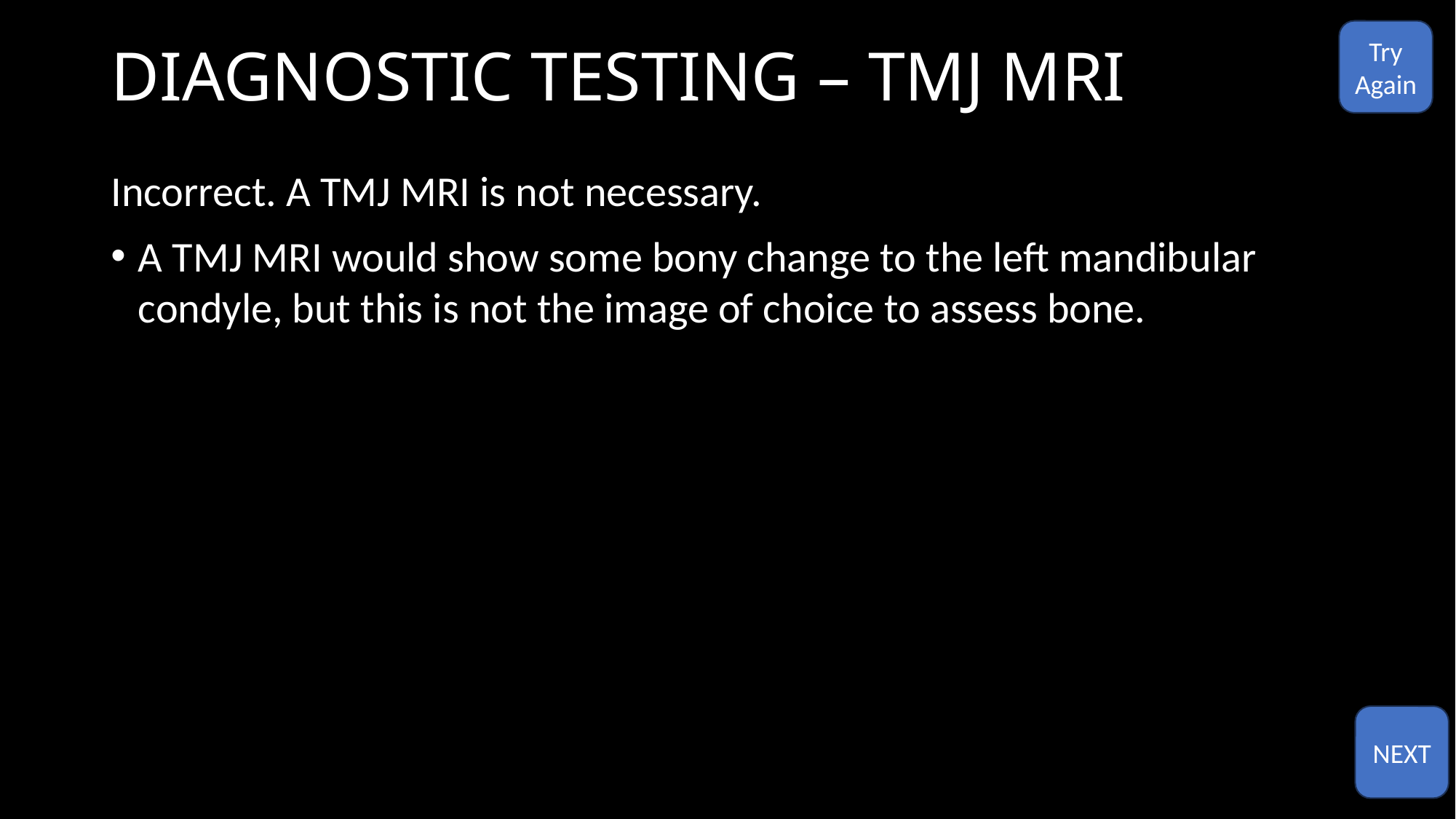

# DIAGNOSTIC TESTING – TMJ MRI
Try
Again
Incorrect. A TMJ MRI is not necessary.
A TMJ MRI would show some bony change to the left mandibular condyle, but this is not the image of choice to assess bone.
NEXT

## Slide 74
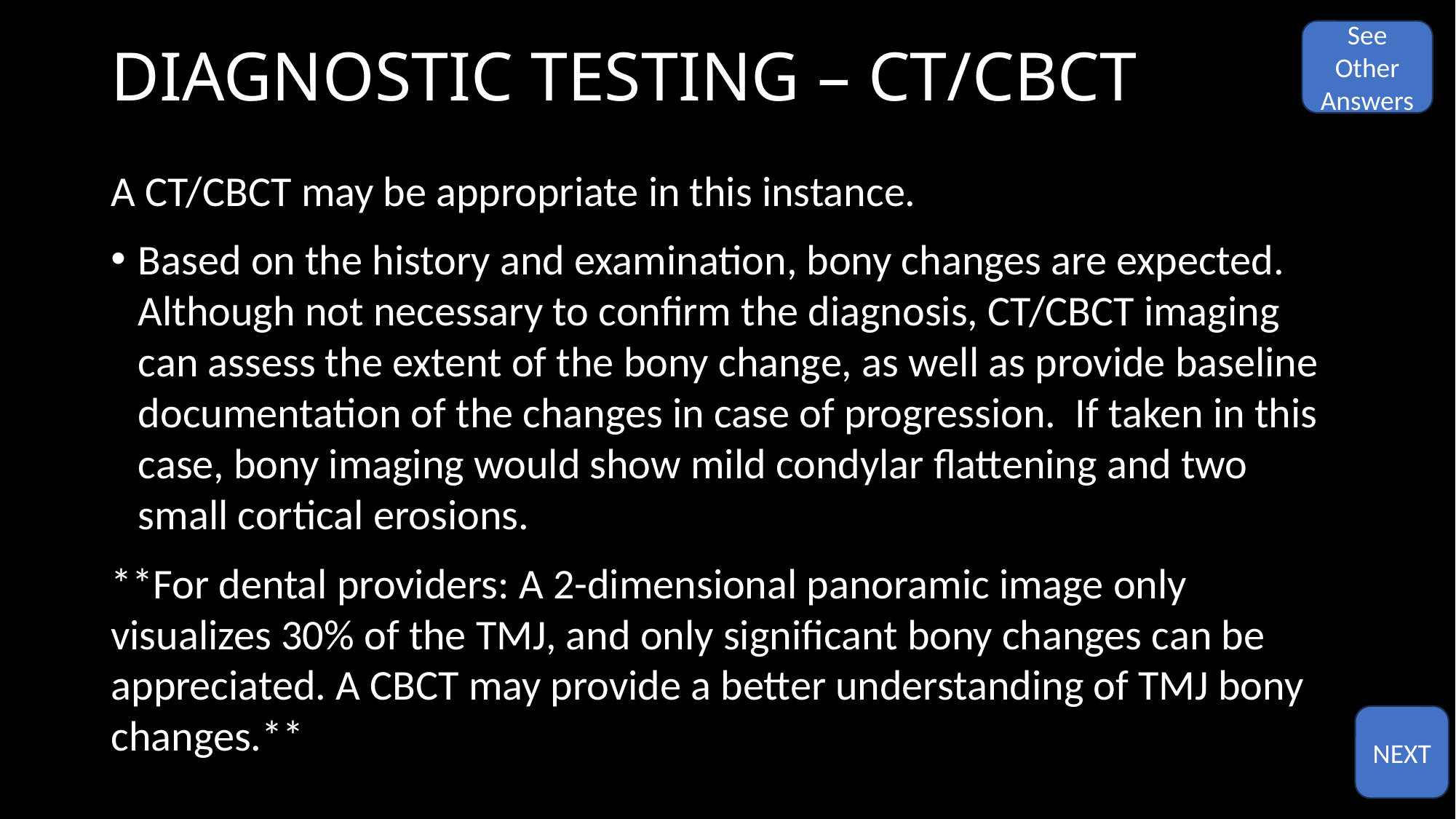

# DIAGNOSTIC TESTING – CT/CBCT
See Other Answers
A CT/CBCT may be appropriate in this instance.
Based on the history and examination, bony changes are expected. Although not necessary to confirm the diagnosis, CT/CBCT imaging can assess the extent of the bony change, as well as provide baseline documentation of the changes in case of progression. If taken in this case, bony imaging would show mild condylar flattening and two small cortical erosions.
**For dental providers: A 2-dimensional panoramic image only visualizes 30% of the TMJ, and only significant bony changes can be appreciated. A CBCT may provide a better understanding of TMJ bony changes.**
NEXT

## Slide 75
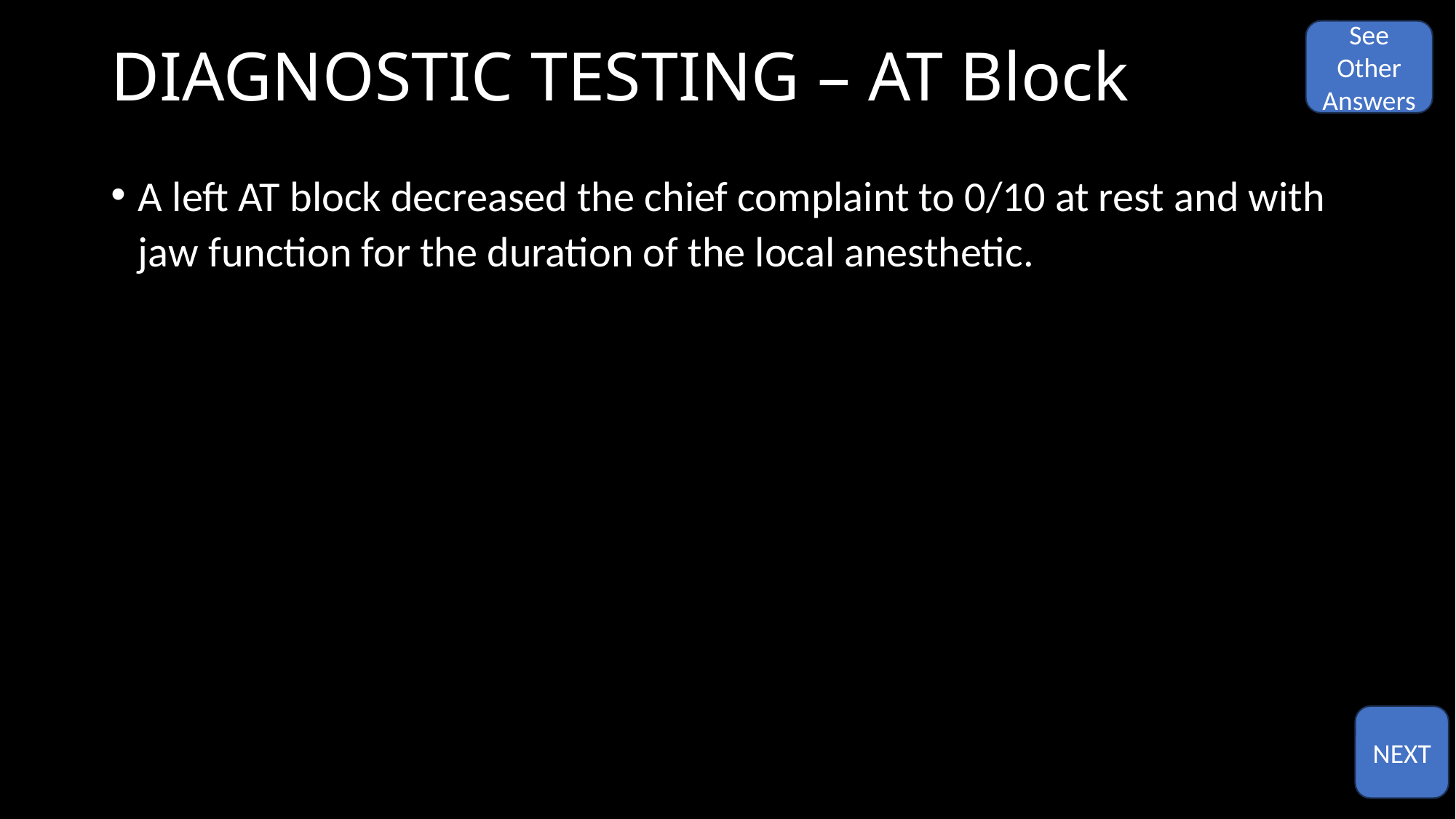

# DIAGNOSTIC TESTING – AT Block
See Other Answers
A left AT block decreased the chief complaint to 0/10 at rest and with jaw function for the duration of the local anesthetic.
NEXT

## Slide 76
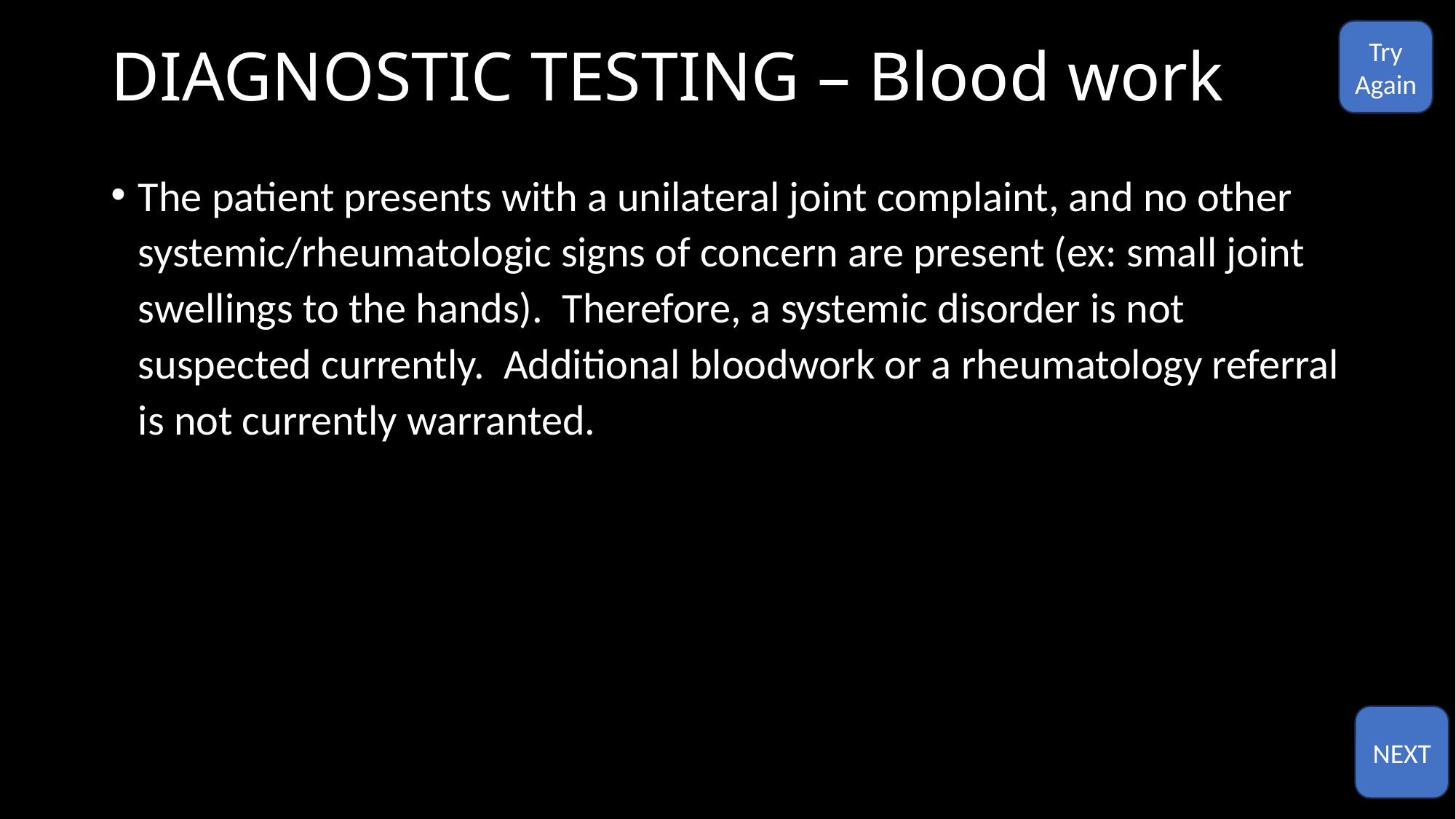

# DIAGNOSTIC TESTING – Blood work
Try
Again
The patient presents with a unilateral joint complaint, and no other systemic/rheumatologic signs of concern are present (ex: small joint swellings to the hands). Therefore, a systemic disorder is not suspected currently. Additional bloodwork or a rheumatology referral is not currently warranted.
NEXT

## Slide 77
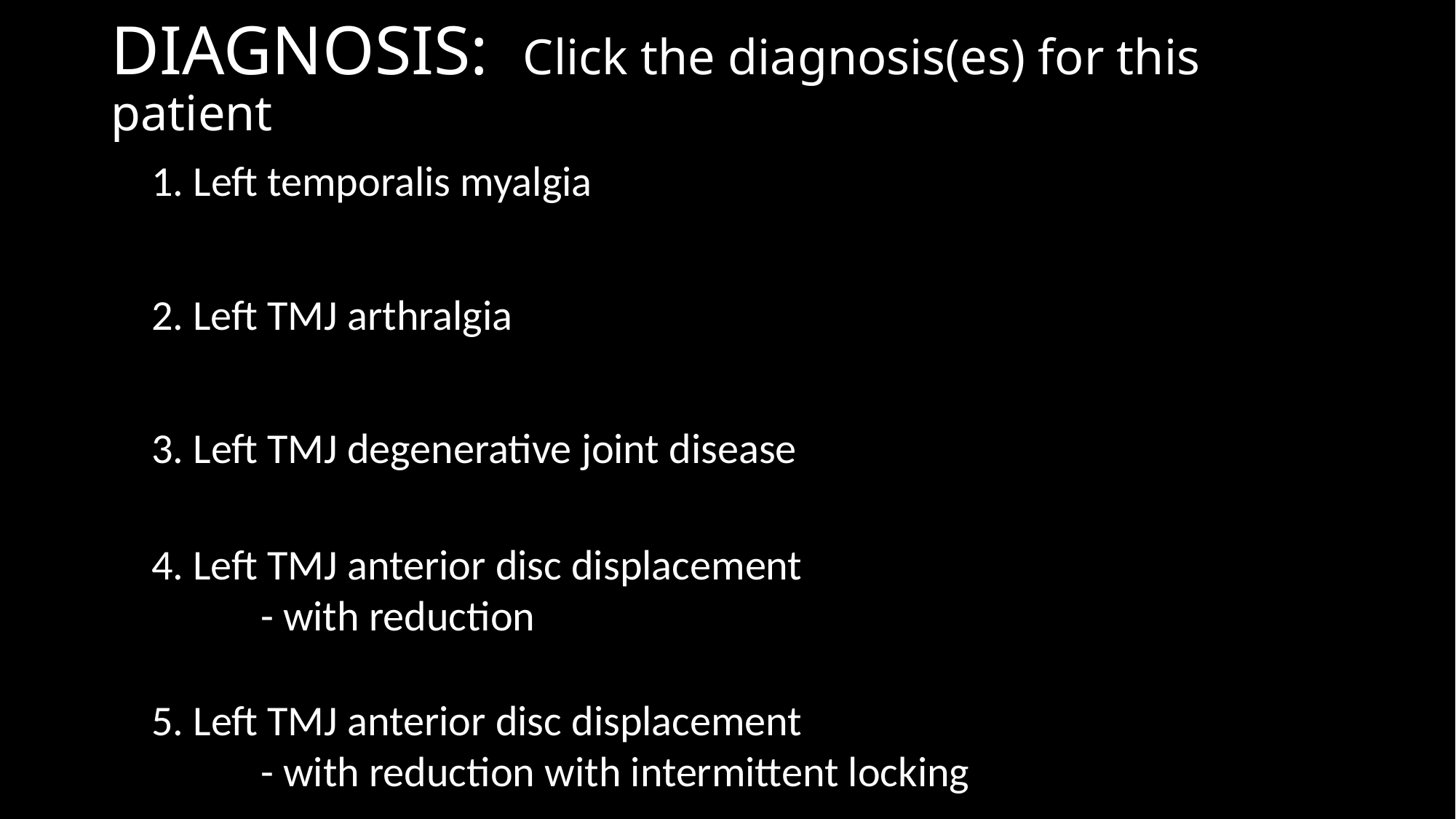

# DIAGNOSIS: Click the diagnosis(es) for this patient
1. Left temporalis myalgia
2. Left TMJ arthralgia
3. Left TMJ degenerative joint disease
4. Left TMJ anterior disc displacement
	- with reduction
5. Left TMJ anterior disc displacement
	- with reduction with intermittent locking

## Slide 78
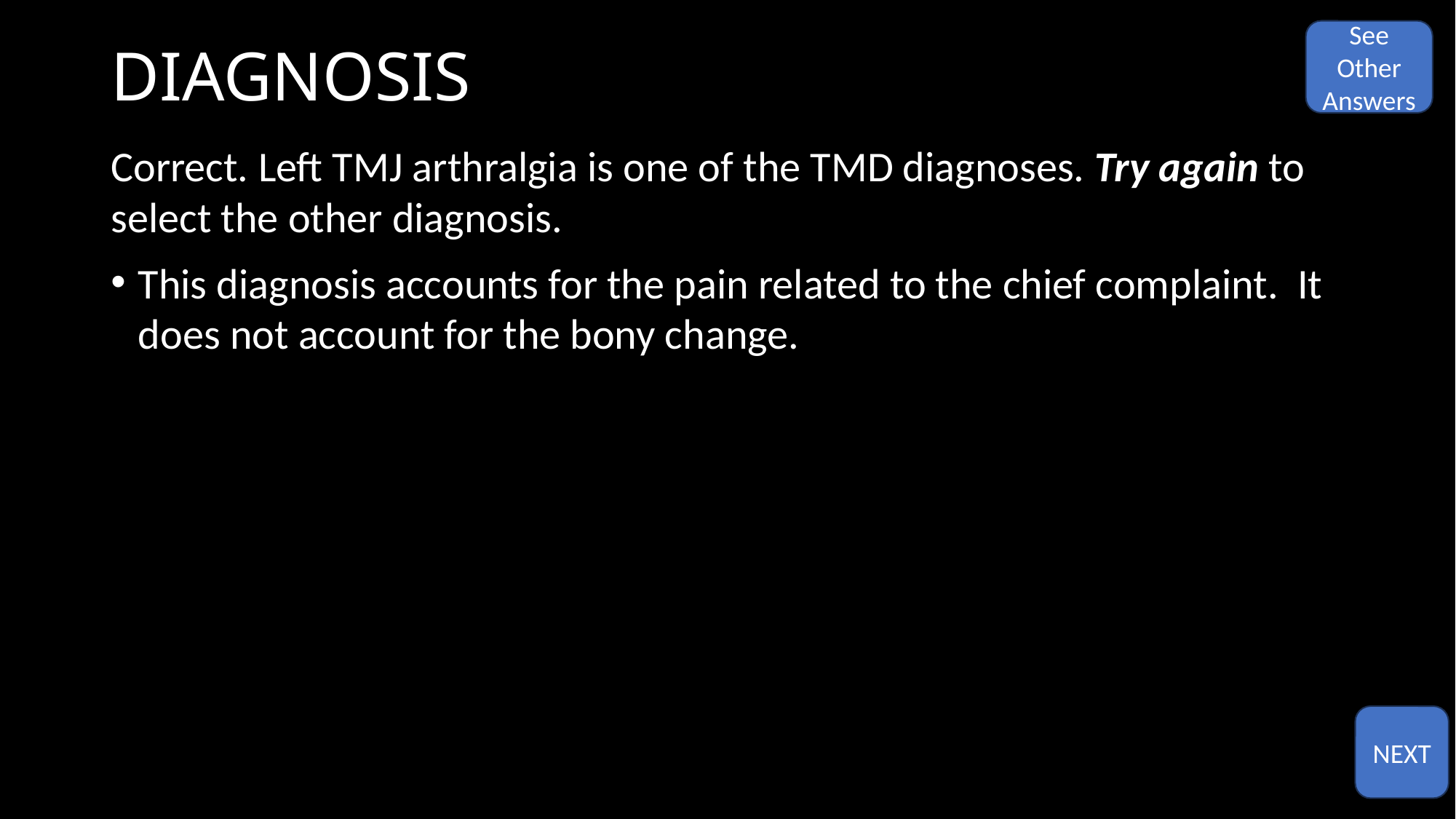

# DIAGNOSIS
See Other Answers
Correct. Left TMJ arthralgia is one of the TMD diagnoses. Try again to select the other diagnosis.
This diagnosis accounts for the pain related to the chief complaint. It does not account for the bony change.
NEXT

## Slide 79
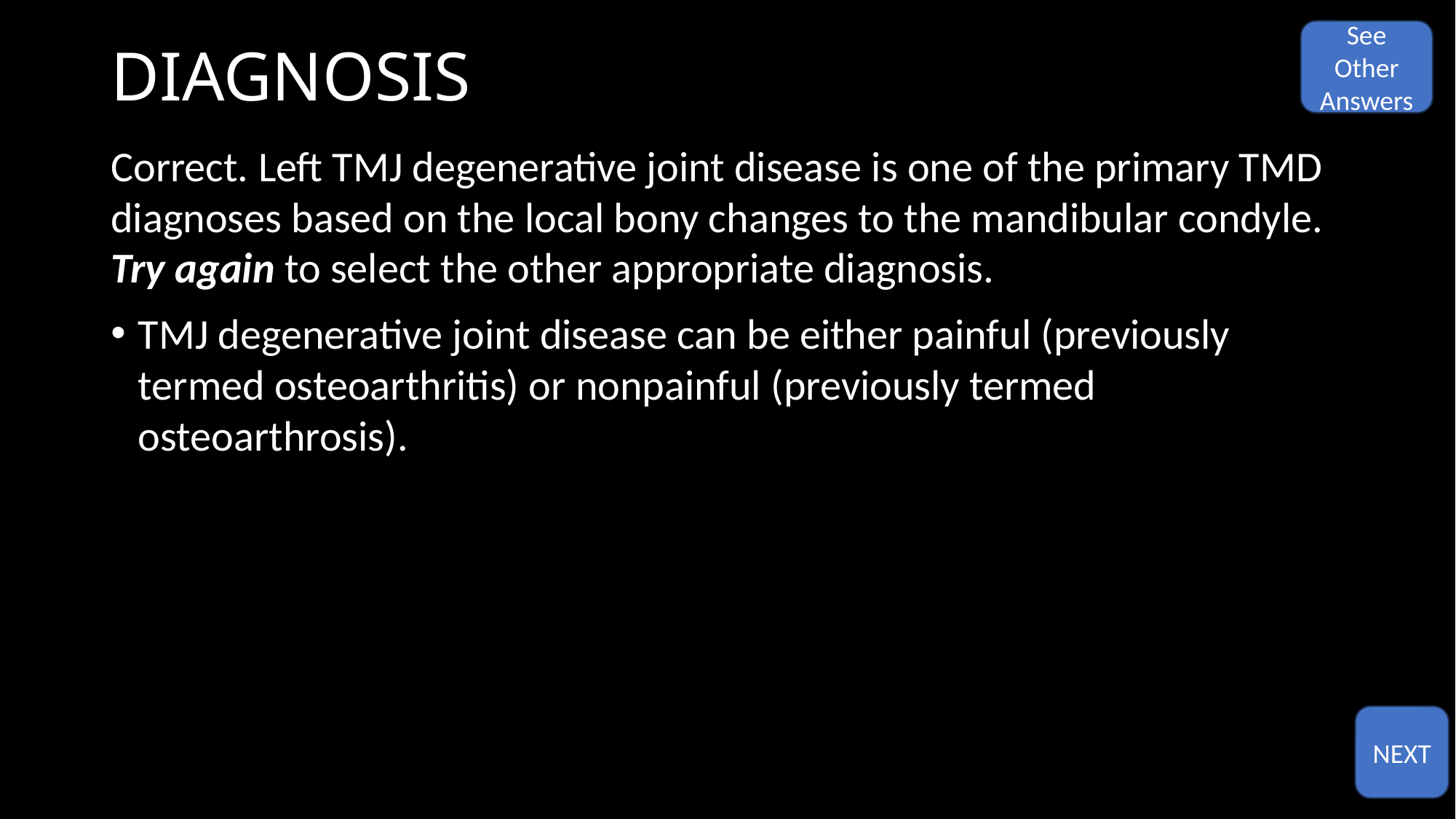

# DIAGNOSIS
See Other Answers
Correct. Left TMJ degenerative joint disease is one of the primary TMD diagnoses based on the local bony changes to the mandibular condyle. Try again to select the other appropriate diagnosis.
TMJ degenerative joint disease can be either painful (previously termed osteoarthritis) or nonpainful (previously termed osteoarthrosis).
NEXT

## Slide 80
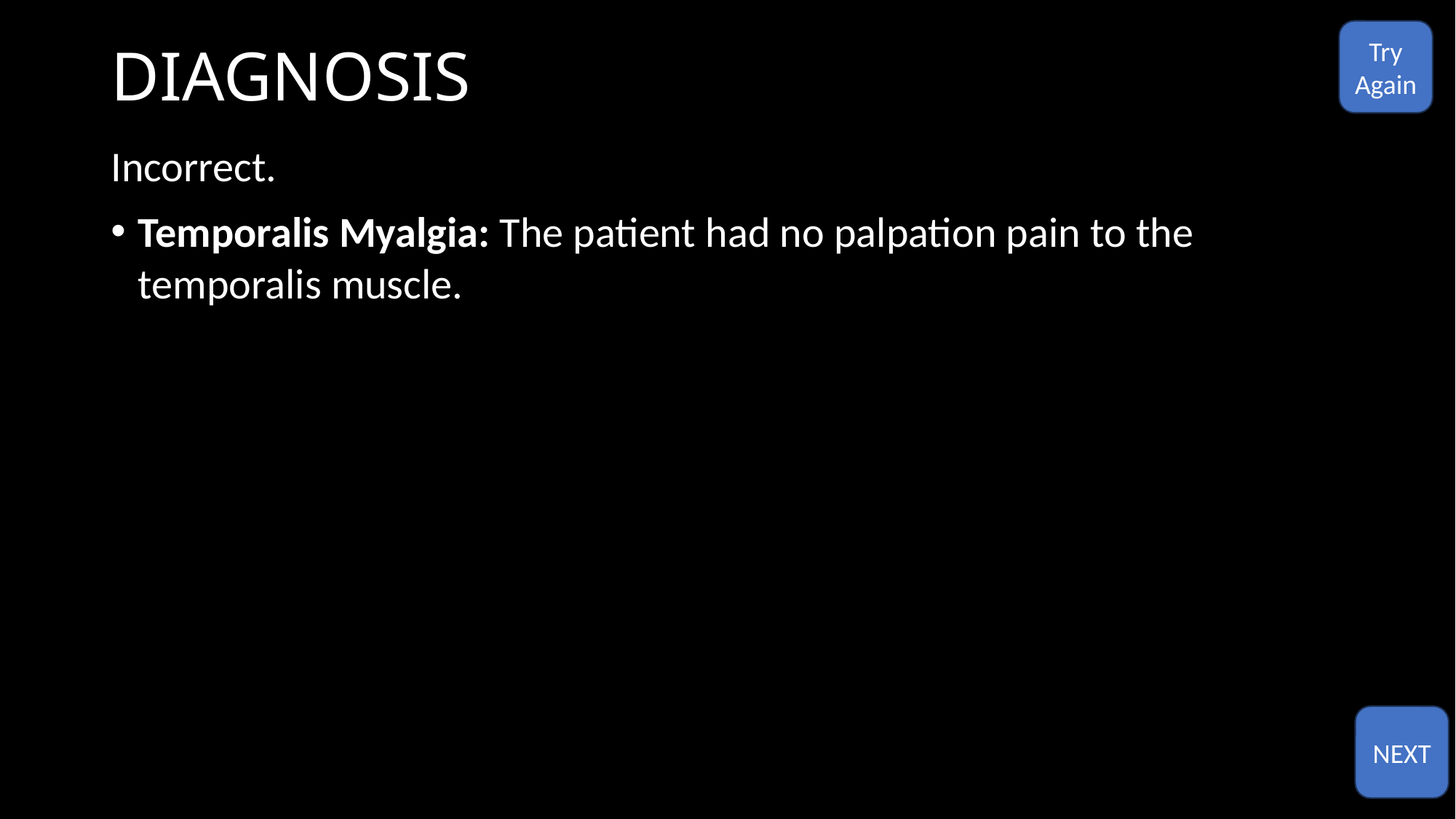

# DIAGNOSIS
Try
Again
Incorrect.
Temporalis Myalgia: The patient had no palpation pain to the temporalis muscle.
NEXT

## Slide 81
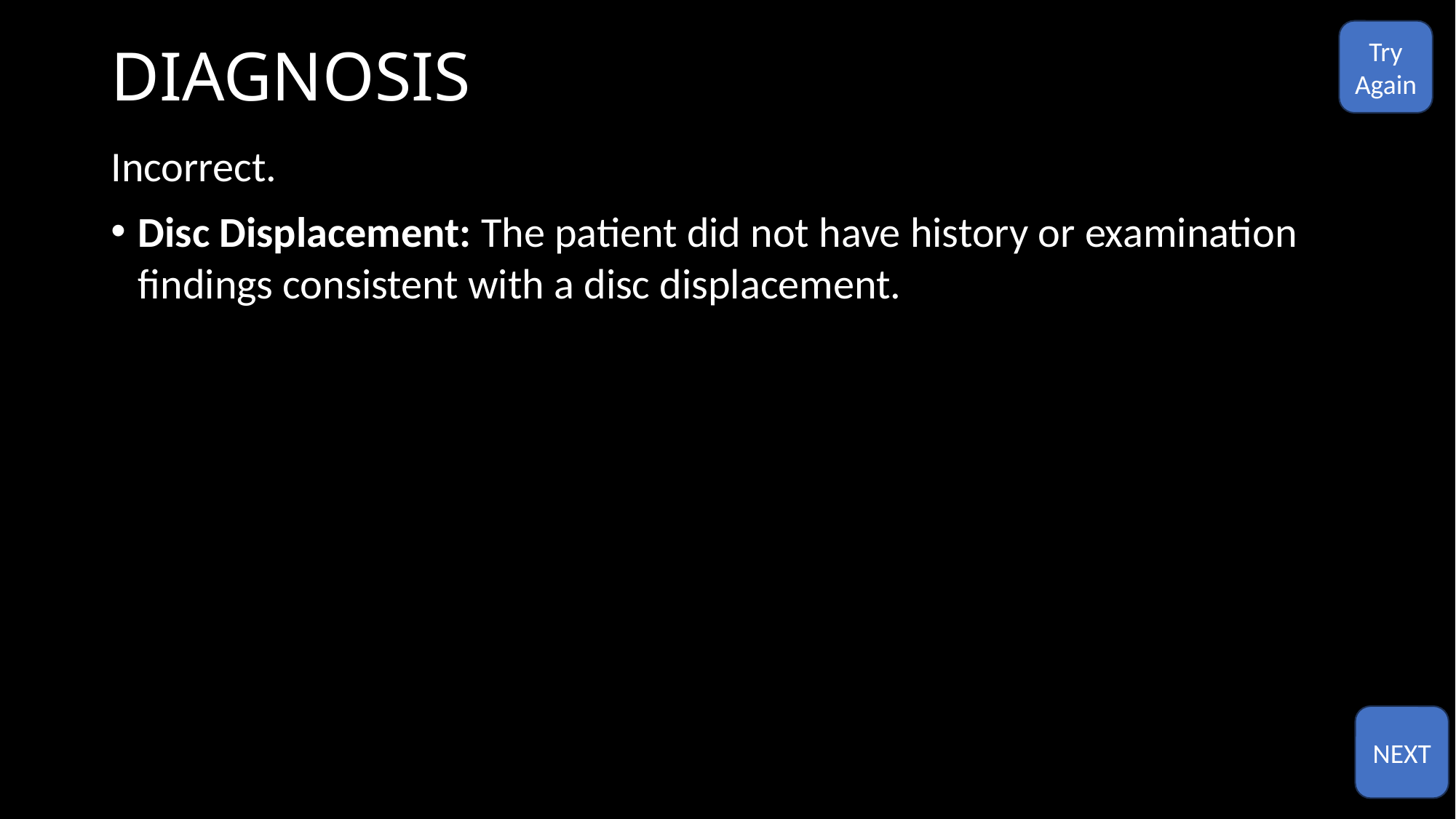

# DIAGNOSIS
Try
Again
Incorrect.
Disc Displacement: The patient did not have history or examination findings consistent with a disc displacement.
NEXT

## Slide 82
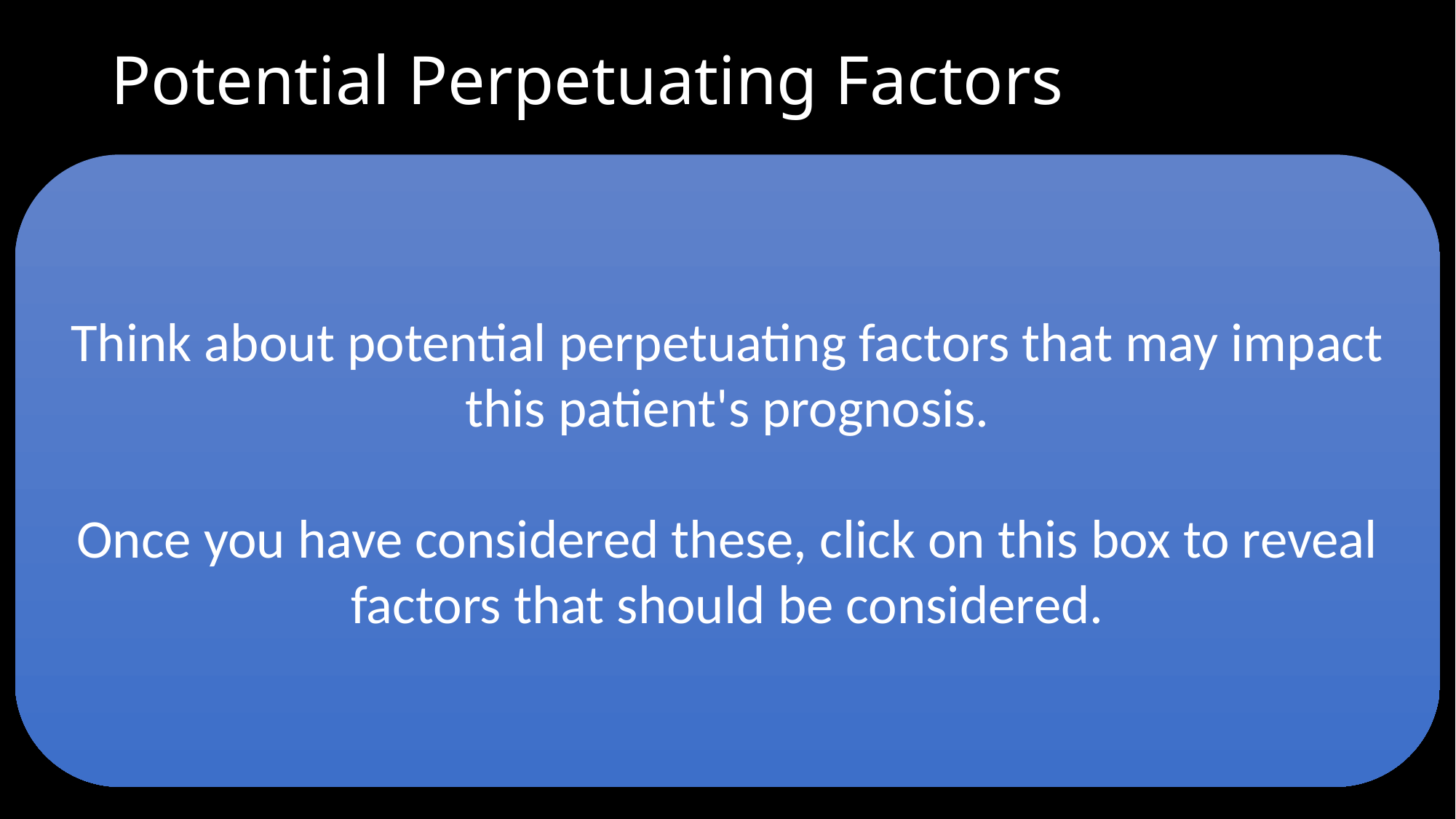

# Potential Perpetuating Factors
Nicotine use
Prolonged history of smoking
Think about potential perpetuating factors that may impact this patient's prognosis.
Once you have considered these, click on this box to reveal factors that should be considered.

## Slide 83
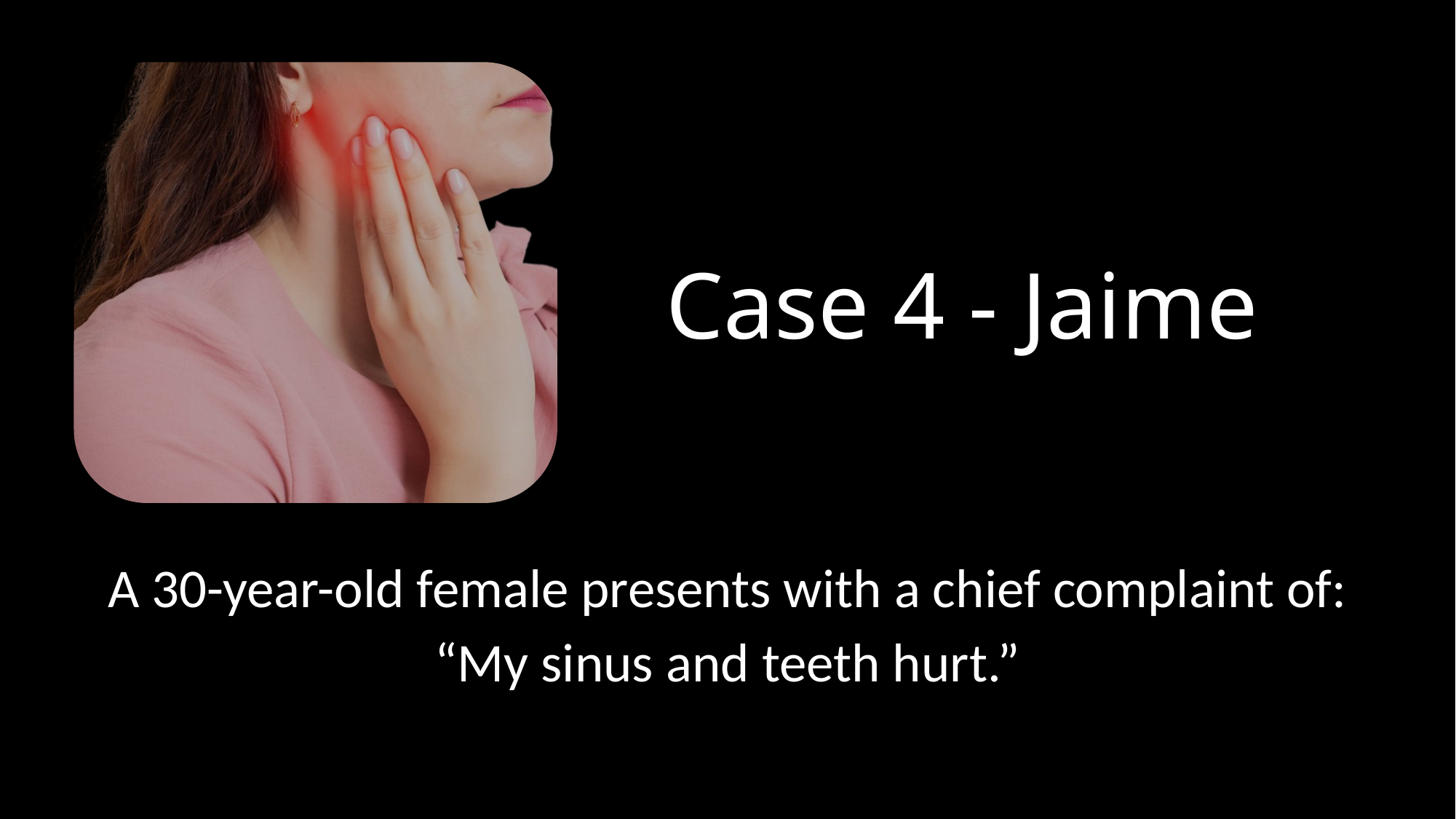

# Case 4 - Jaime
A 30-year-old female presents with a chief complaint of:
“My sinus and teeth hurt.”

## Slide 84
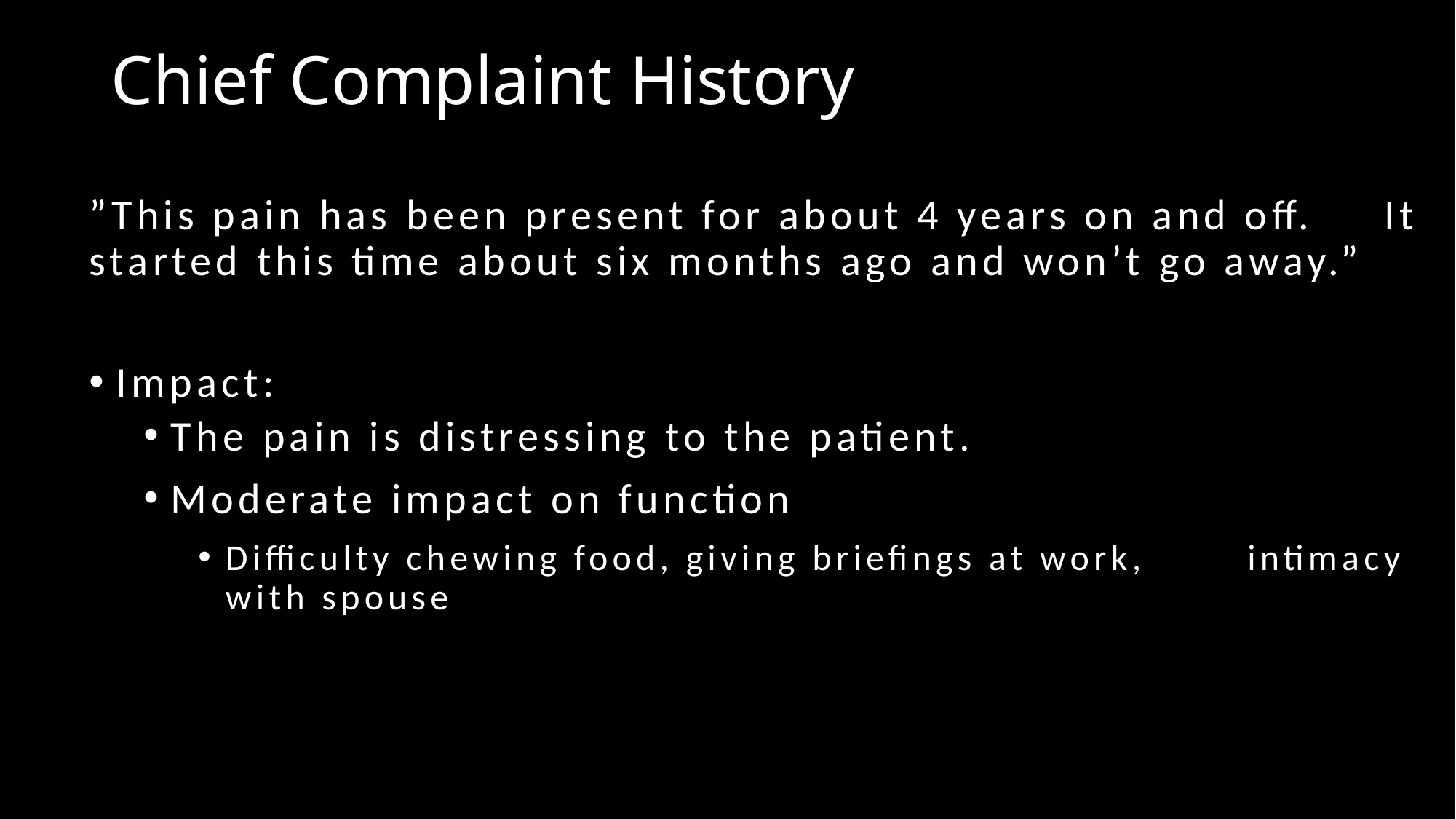

# Chief Complaint History
”This pain has been present for about 4 years on and off. It started this time about six months ago and won’t go away.”
Impact:
The pain is distressing to the patient.
Moderate impact on function
Difficulty chewing food, giving briefings at work, intimacy with spouse

## Slide 85
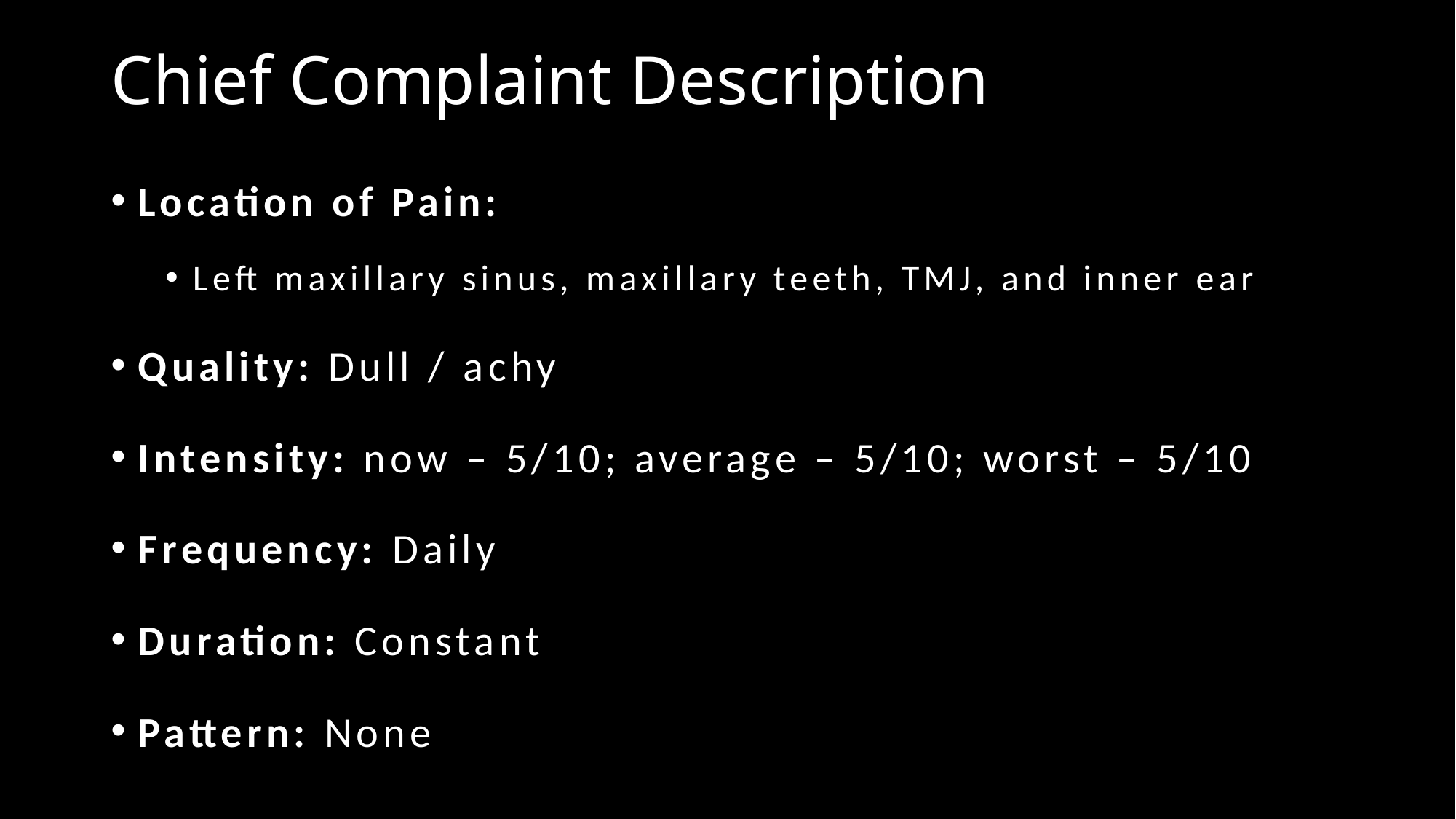

# Chief Complaint Description
Location of Pain:
Left maxillary sinus, maxillary teeth, TMJ, and inner ear
Quality: Dull / achy
Intensity: now – 5/10; average – 5/10; worst – 5/10
Frequency: Daily
Duration: Constant
Pattern: None

## Slide 86
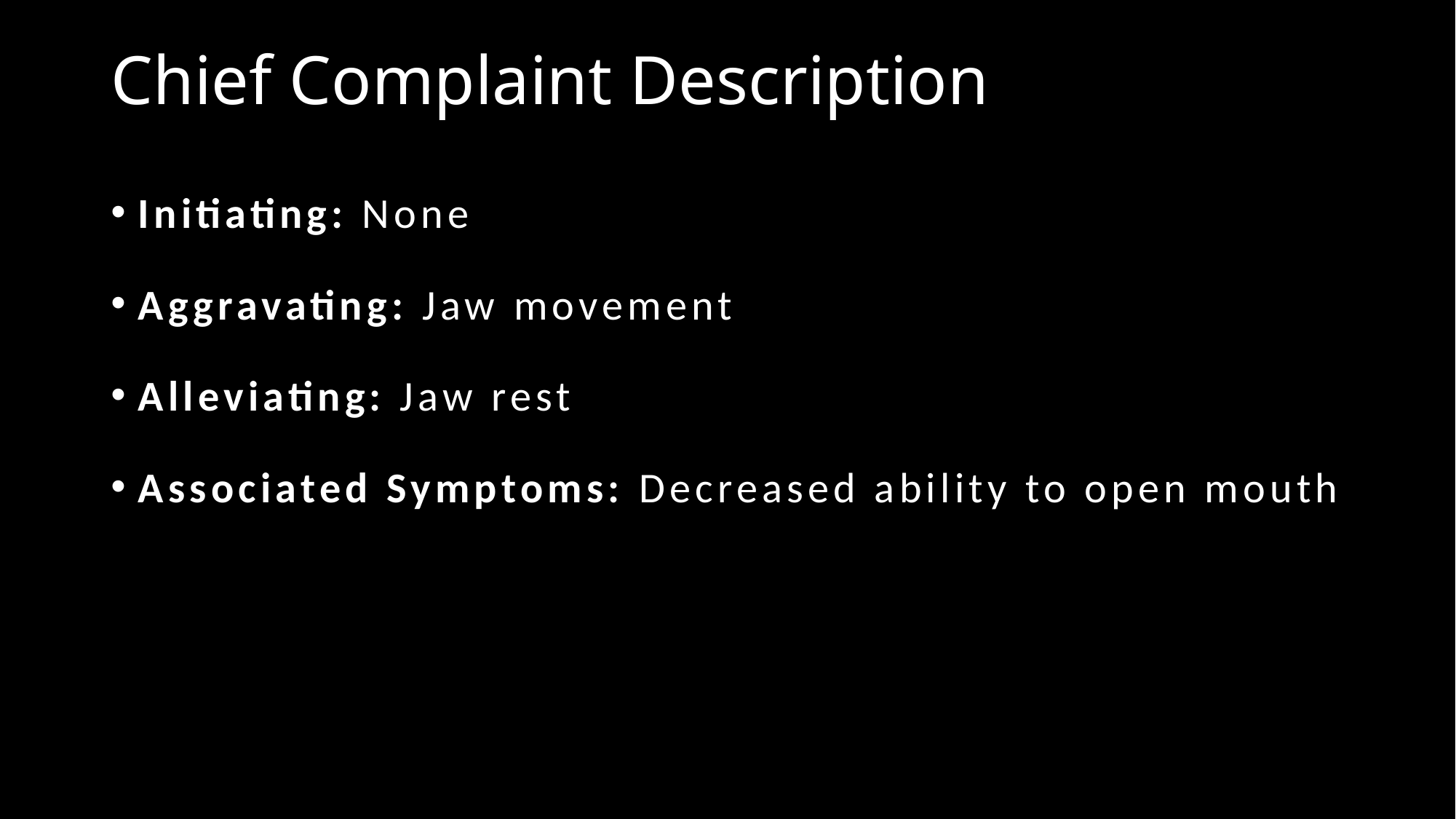

# Chief Complaint Description
Initiating: None
Aggravating: Jaw movement
Alleviating: Jaw rest
Associated Symptoms: Decreased ability to open mouth

## Slide 87
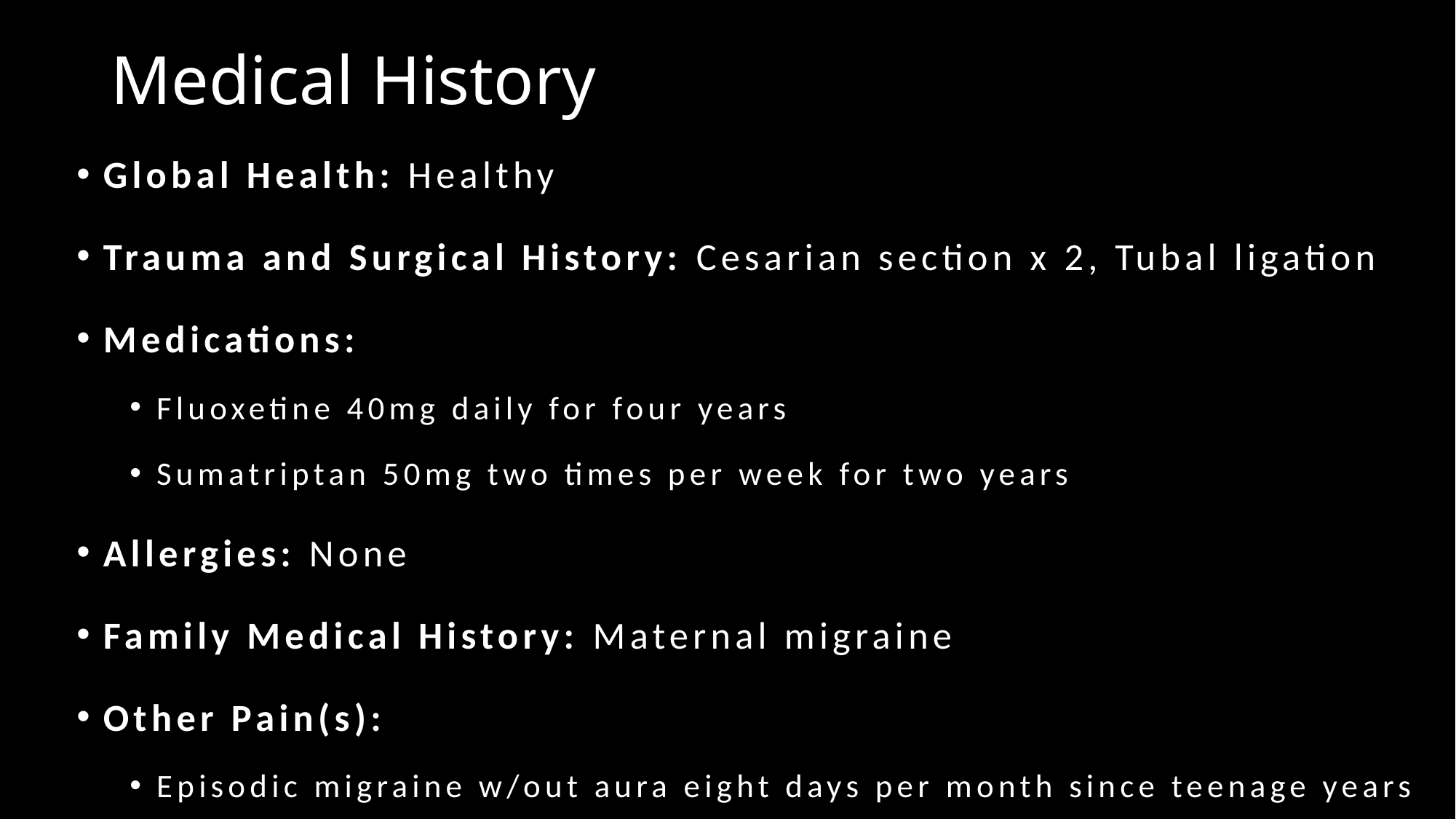

# Medical History
Global Health: Healthy
Trauma and Surgical History: Cesarian section x 2, Tubal ligation
Medications:
Fluoxetine 40mg daily for four years
Sumatriptan 50mg two times per week for two years
Allergies: None
Family Medical History: Maternal migraine
Other Pain(s):
Episodic migraine w/out aura eight days per month since teenage years

## Slide 88
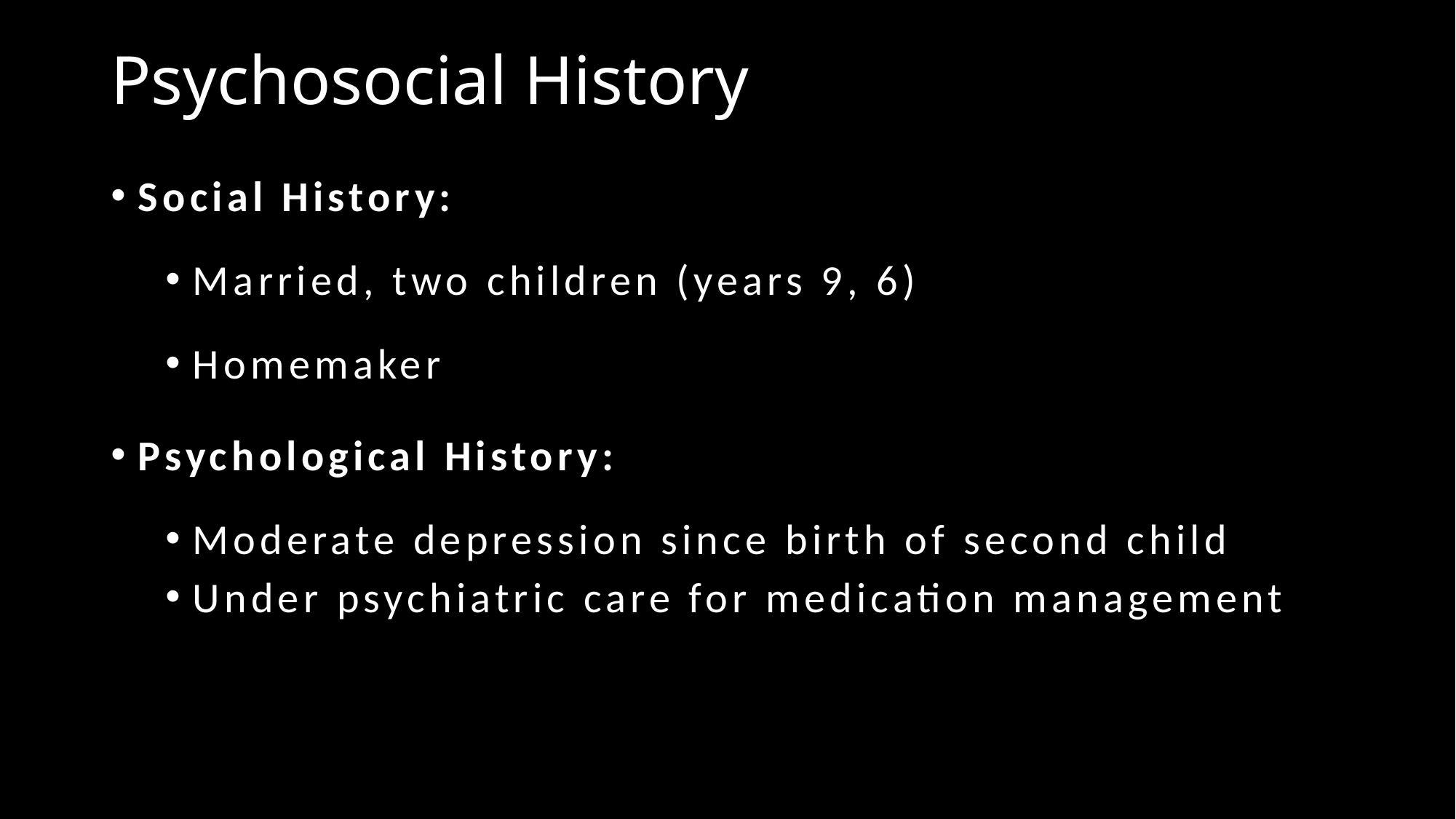

# Psychosocial History
Social History:
Married, two children (years 9, 6)
Homemaker
Psychological History:
Moderate depression since birth of second child
Under psychiatric care for medication management

## Slide 89
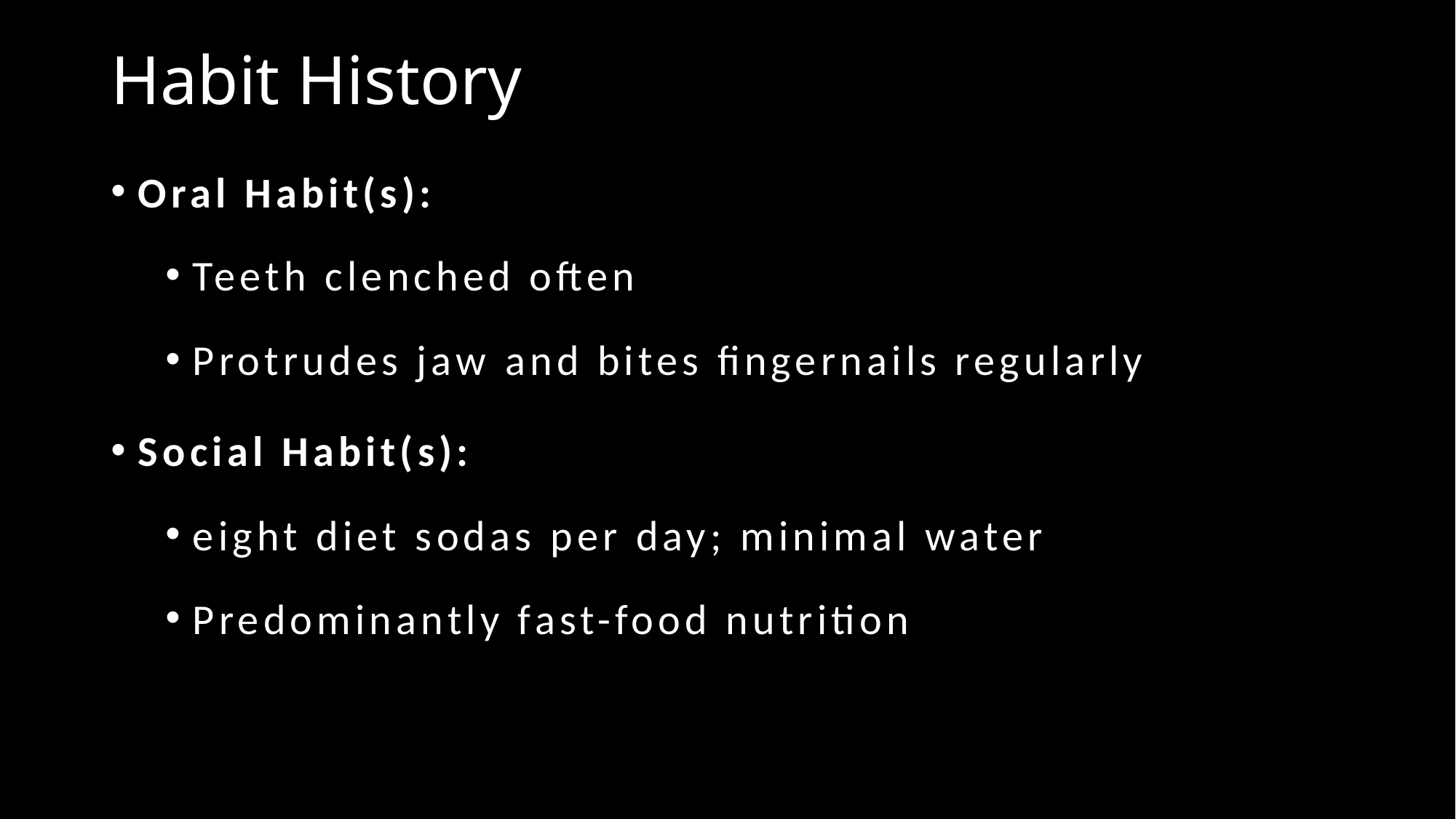

# Habit History
Oral Habit(s):
Teeth clenched often
Protrudes jaw and bites fingernails regularly
Social Habit(s):
eight diet sodas per day; minimal water
Predominantly fast-food nutrition

## Slide 90
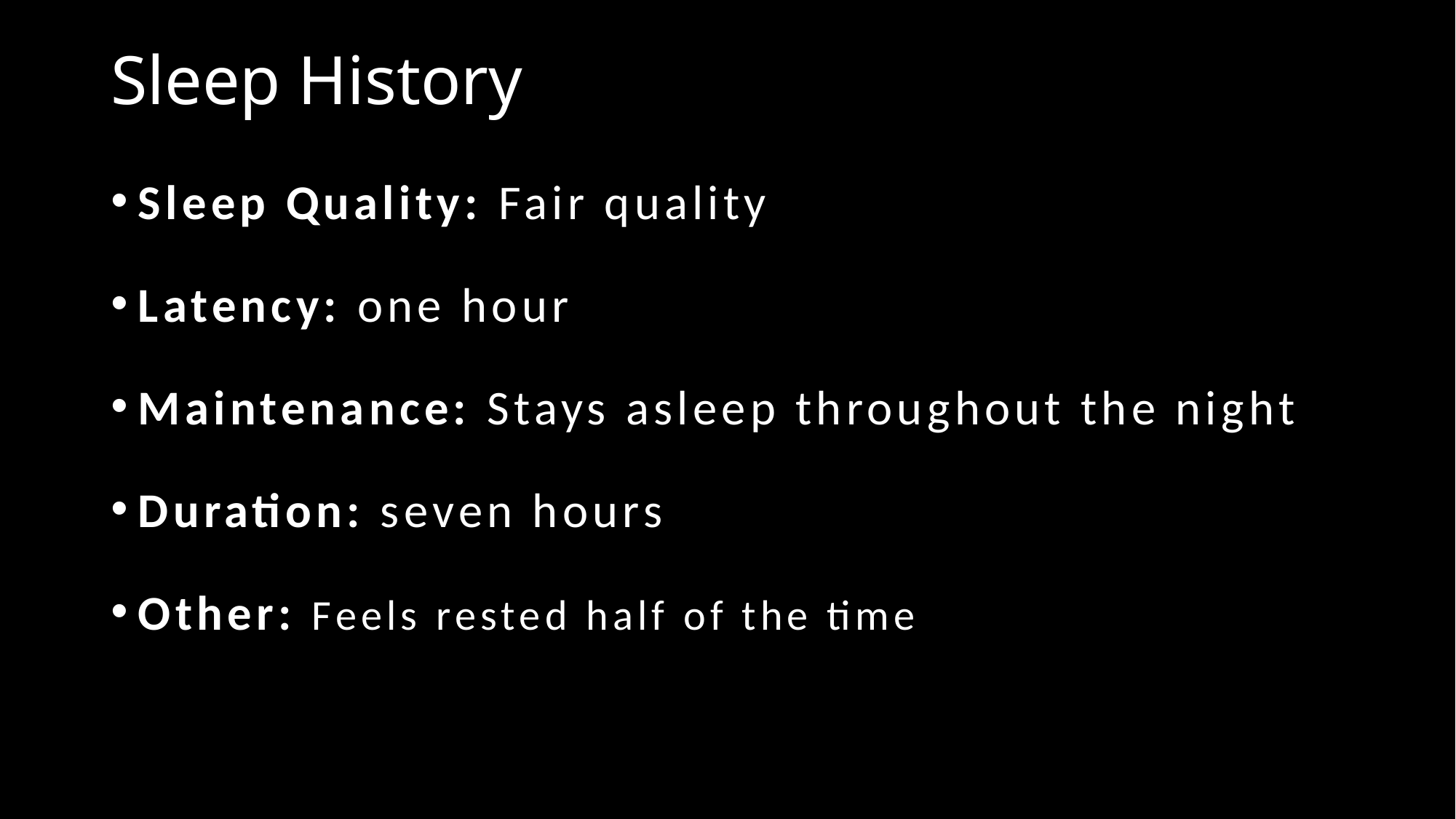

# Sleep History
Sleep Quality: Fair quality
Latency: one hour
Maintenance: Stays asleep throughout the night
Duration: seven hours
Other: Feels rested half of the time

## Slide 91
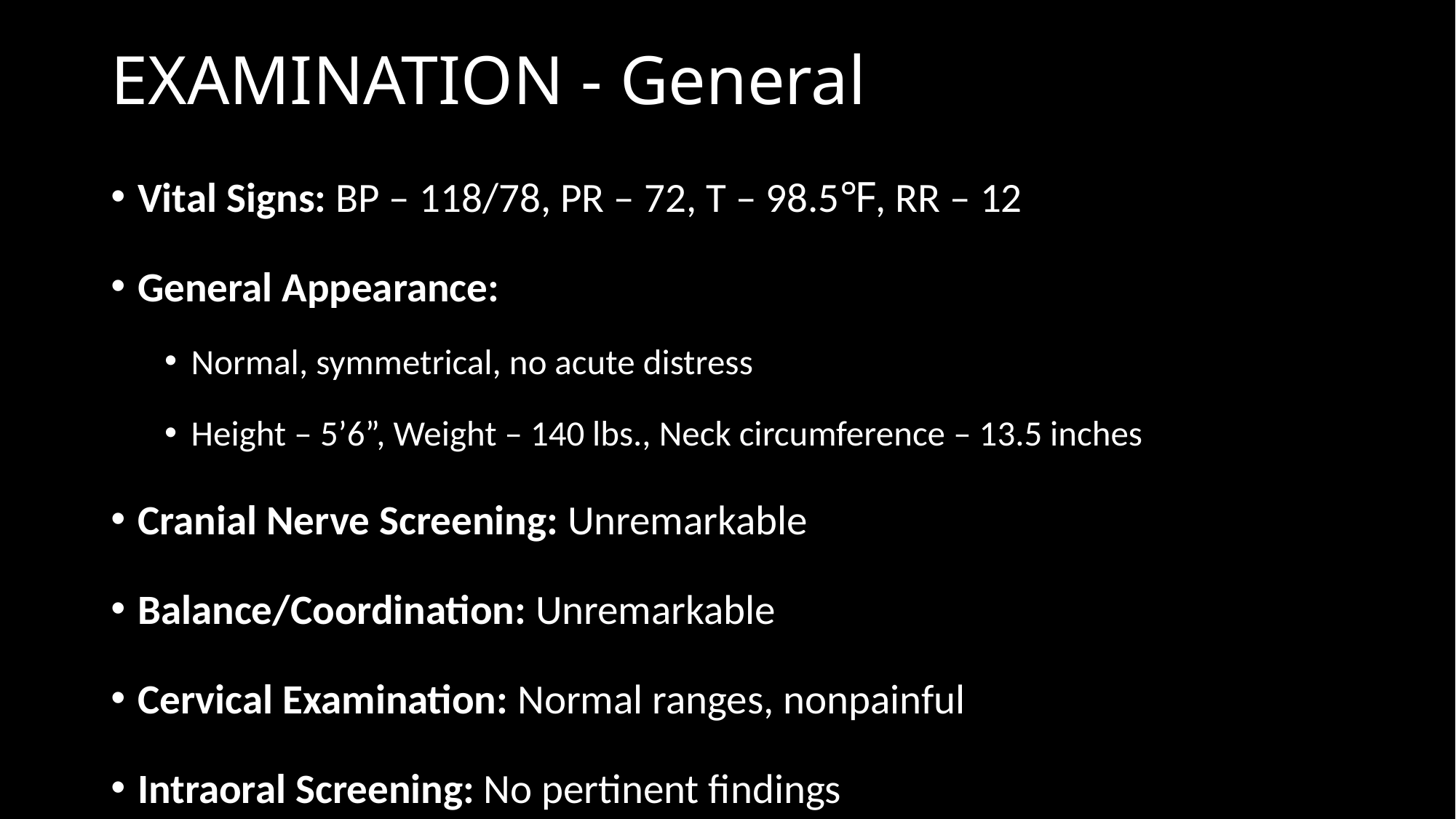

# EXAMINATION - General
Vital Signs: BP – 118/78, PR – 72, T – 98.5℉, RR – 12
General Appearance:
Normal, symmetrical, no acute distress
Height – 5’6”, Weight – 140 lbs., Neck circumference – 13.5 inches
Cranial Nerve Screening: Unremarkable
Balance/Coordination: Unremarkable
Cervical Examination: Normal ranges, nonpainful
Intraoral Screening: No pertinent findings

## Slide 92
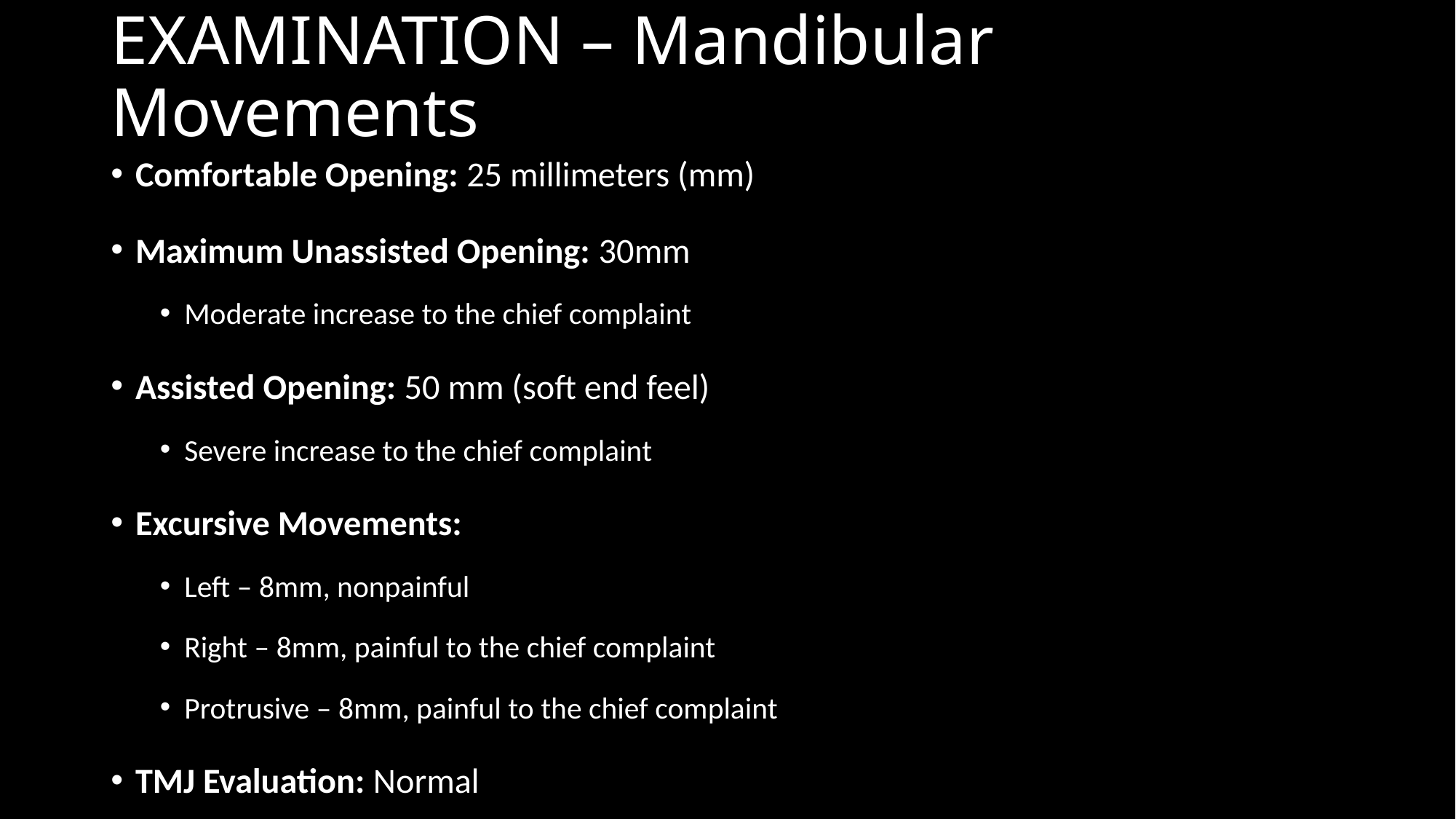

# EXAMINATION – Mandibular Movements
Comfortable Opening: 25 millimeters (mm)
Maximum Unassisted Opening: 30mm
Moderate increase to the chief complaint
Assisted Opening: 50 mm (soft end feel)
Severe increase to the chief complaint
Excursive Movements:
Left – 8mm, nonpainful
Right – 8mm, painful to the chief complaint
Protrusive – 8mm, painful to the chief complaint
TMJ Evaluation: Normal

## Slide 93
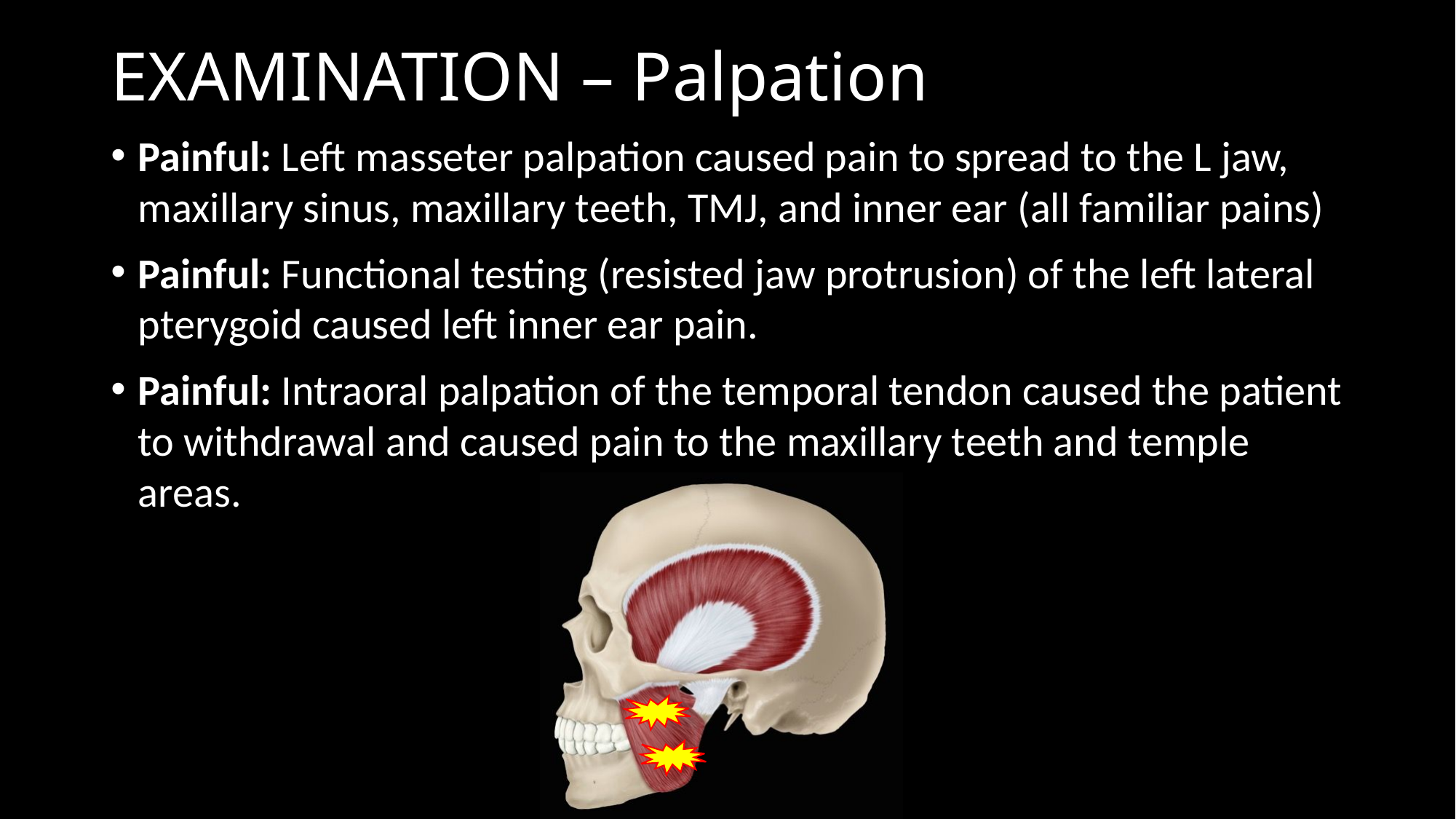

# EXAMINATION – Palpation
Painful: Left masseter palpation caused pain to spread to the L jaw, maxillary sinus, maxillary teeth, TMJ, and inner ear (all familiar pains)
Painful: Functional testing (resisted jaw protrusion) of the left lateral pterygoid caused left inner ear pain.
Painful: Intraoral palpation of the temporal tendon caused the patient to withdrawal and caused pain to the maxillary teeth and temple areas.

## Slide 94
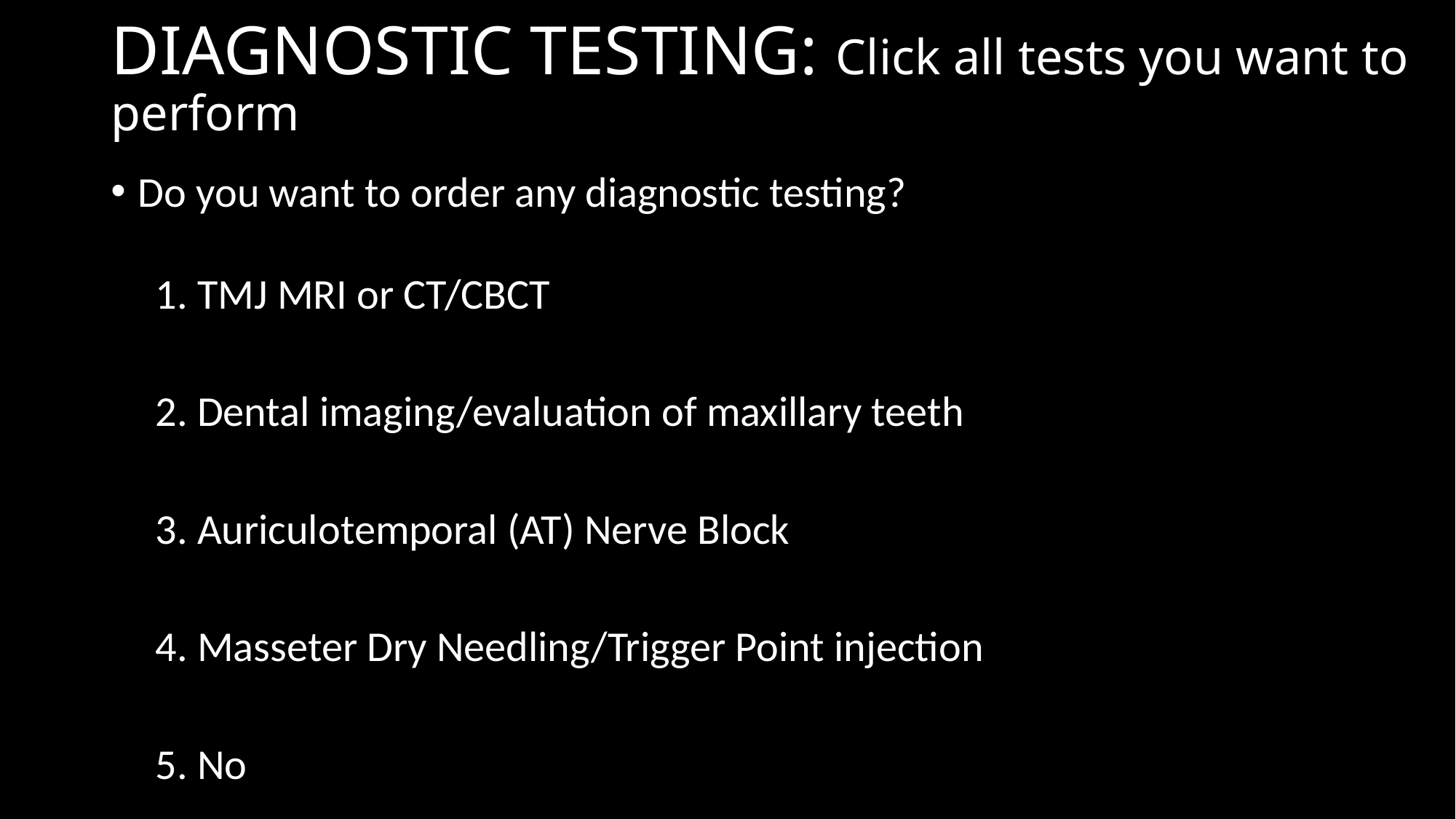

DIAGNOSTIC TESTING: Click all tests you want to perform
Do you want to order any diagnostic testing?
1. TMJ MRI or CT/CBCT
2. Dental imaging/evaluation of maxillary teeth
3. Auriculotemporal (AT) Nerve Block
4. Masseter Dry Needling/Trigger Point injection
5. No

## Slide 95
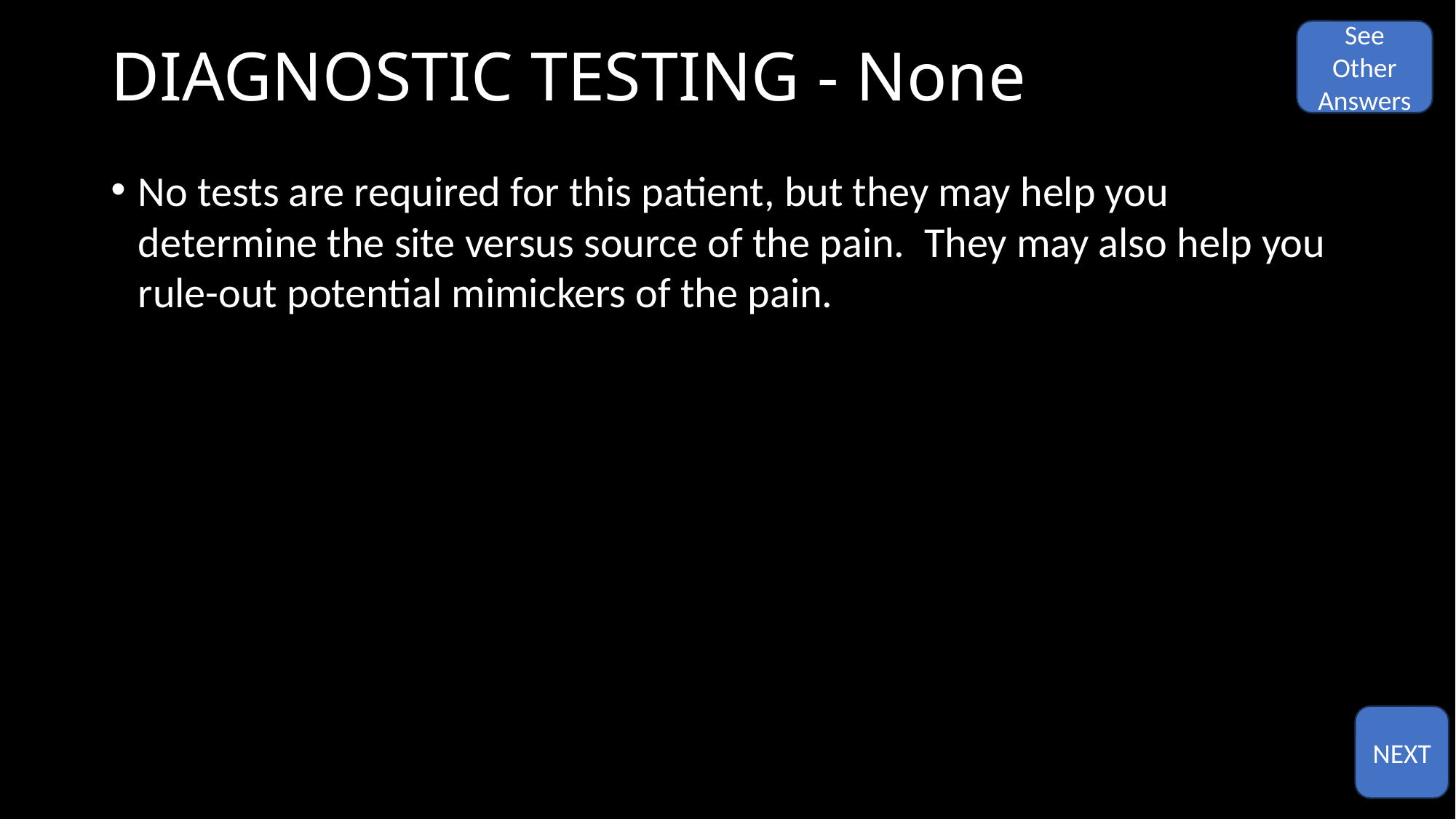

# DIAGNOSTIC TESTING - None
See Other Answers
No tests are required for this patient, but they may help you determine the site versus source of the pain. They may also help you rule-out potential mimickers of the pain.
NEXT

## Slide 96
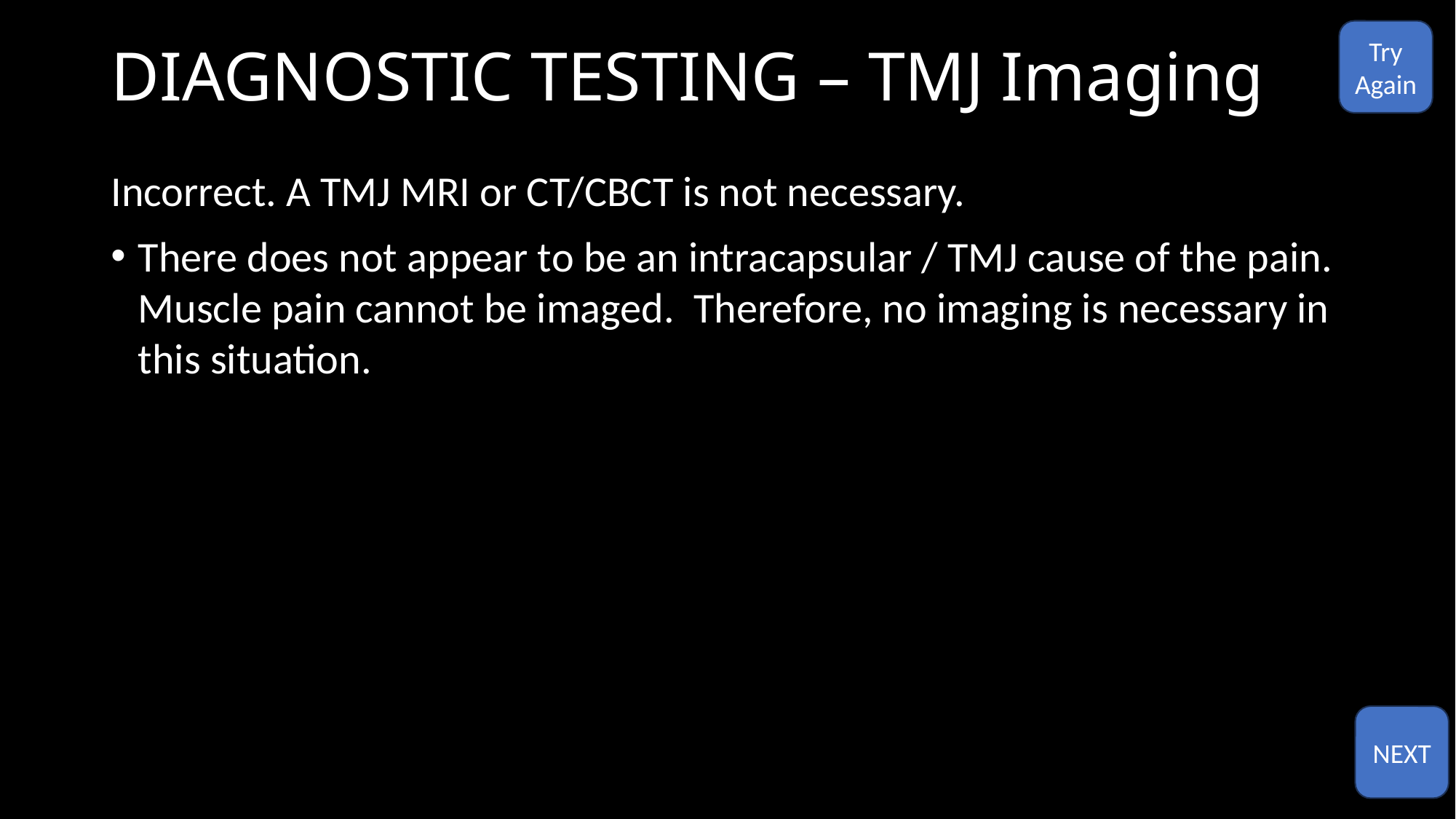

# DIAGNOSTIC TESTING – TMJ Imaging
Try
Again
Incorrect. A TMJ MRI or CT/CBCT is not necessary.
There does not appear to be an intracapsular / TMJ cause of the pain. Muscle pain cannot be imaged. Therefore, no imaging is necessary in this situation.
NEXT

## Slide 97
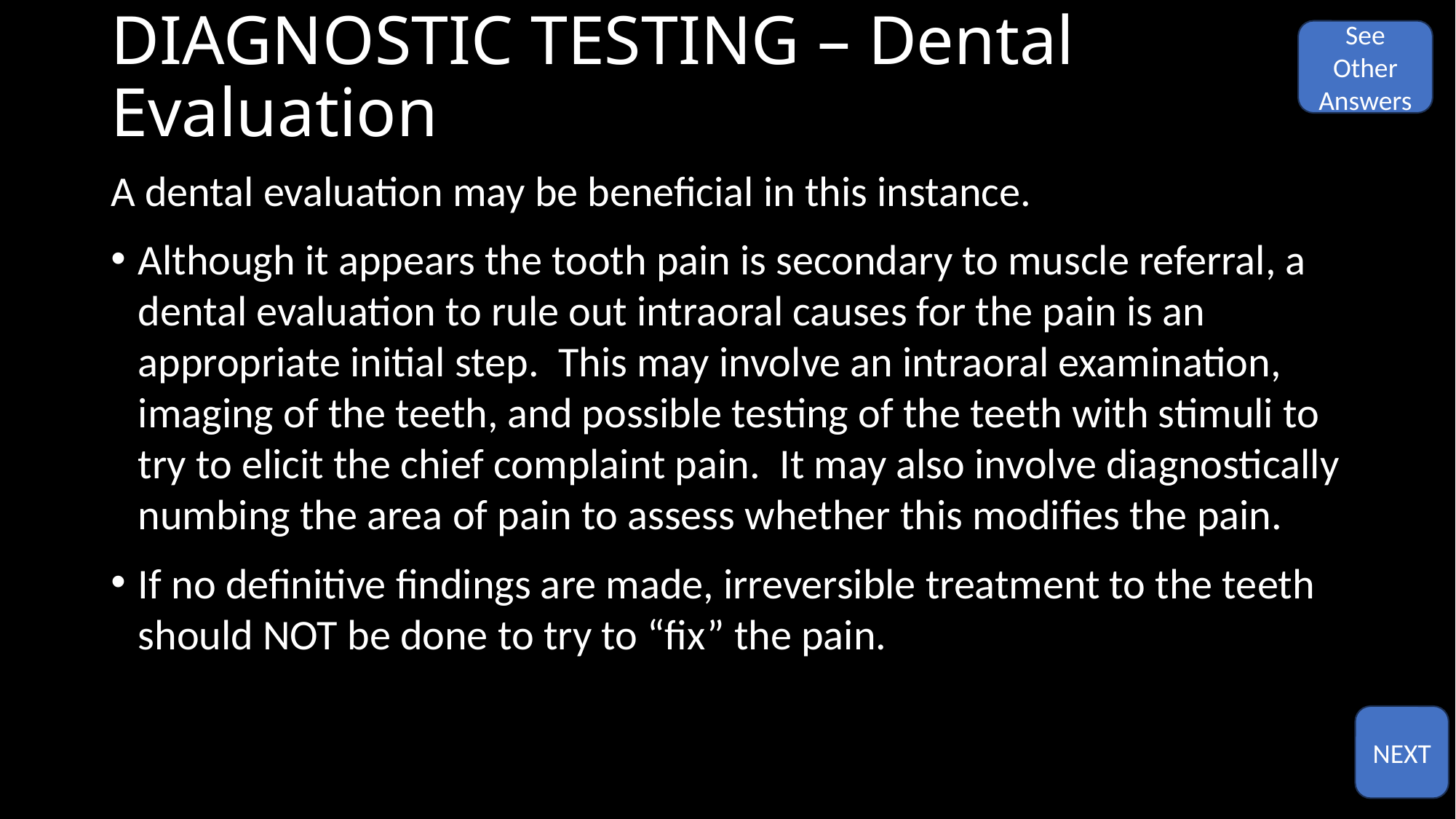

# DIAGNOSTIC TESTING – Dental Evaluation
See Other Answers
A dental evaluation may be beneficial in this instance.
Although it appears the tooth pain is secondary to muscle referral, a dental evaluation to rule out intraoral causes for the pain is an appropriate initial step. This may involve an intraoral examination, imaging of the teeth, and possible testing of the teeth with stimuli to try to elicit the chief complaint pain. It may also involve diagnostically numbing the area of pain to assess whether this modifies the pain.
If no definitive findings are made, irreversible treatment to the teeth should NOT be done to try to “fix” the pain.
NEXT

## Slide 98
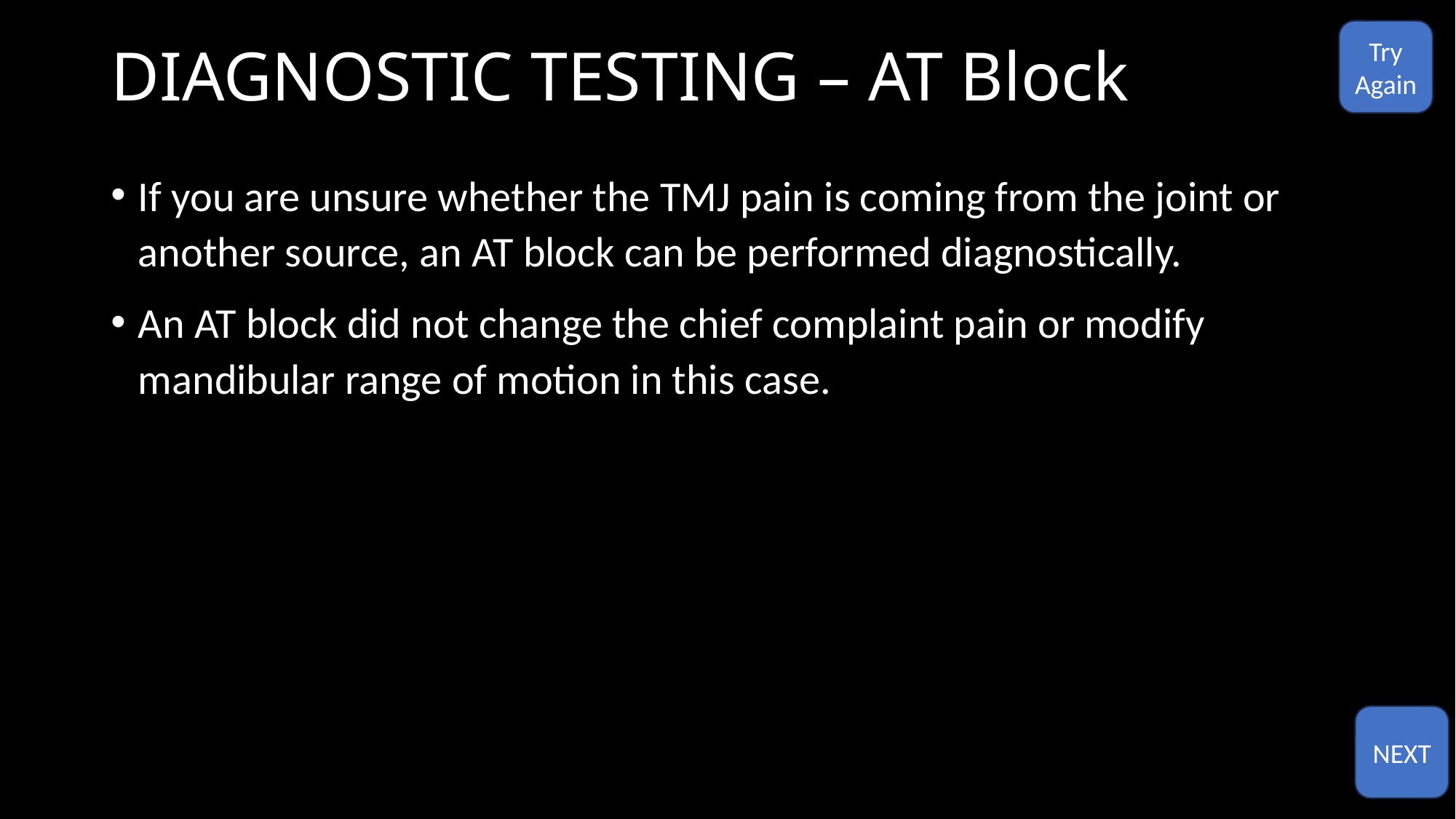

# DIAGNOSTIC TESTING – AT Block
Try
Again
If you are unsure whether the TMJ pain is coming from the joint or another source, an AT block can be performed diagnostically.
An AT block did not change the chief complaint pain or modify mandibular range of motion in this case.
NEXT

## Slide 99
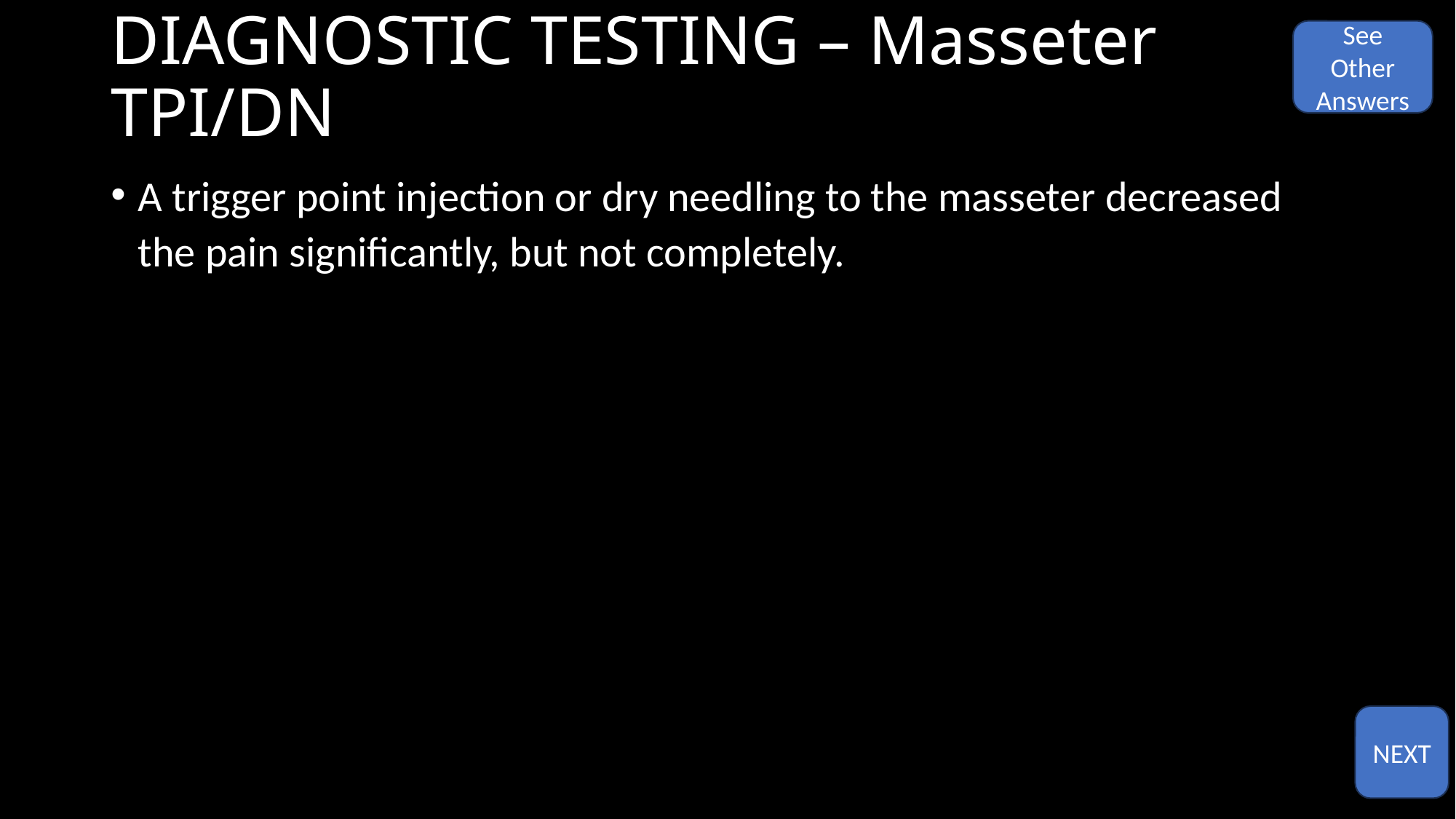

# DIAGNOSTIC TESTING – Masseter TPI/DN
See Other Answers
A trigger point injection or dry needling to the masseter decreased the pain significantly, but not completely.
NEXT

## Slide 100
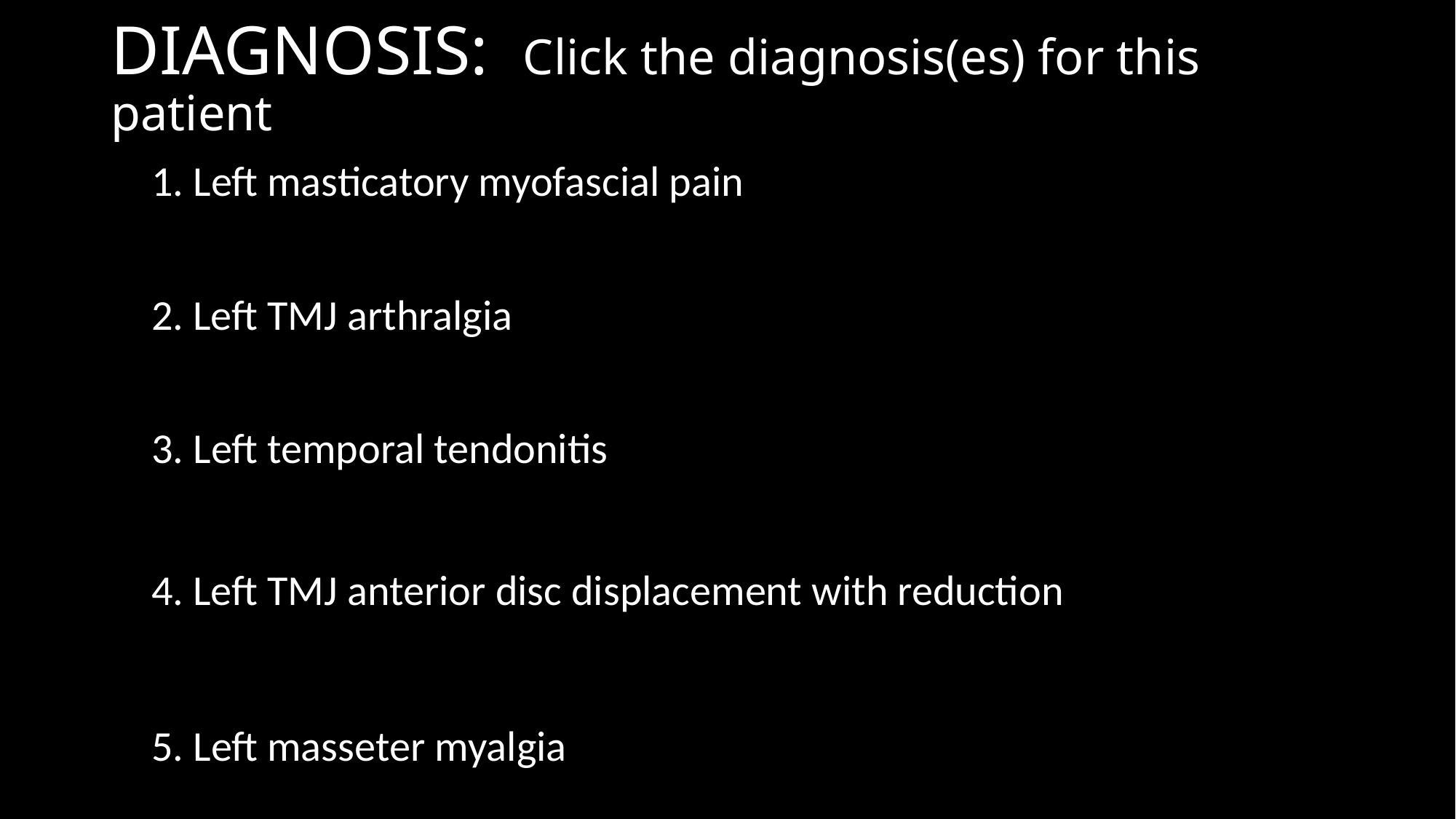

# DIAGNOSIS: Click the diagnosis(es) for this patient
1. Left masticatory myofascial pain
2. Left TMJ arthralgia
3. Left temporal tendonitis
4. Left TMJ anterior disc displacement with reduction
5. Left masseter myalgia

## Slide 101
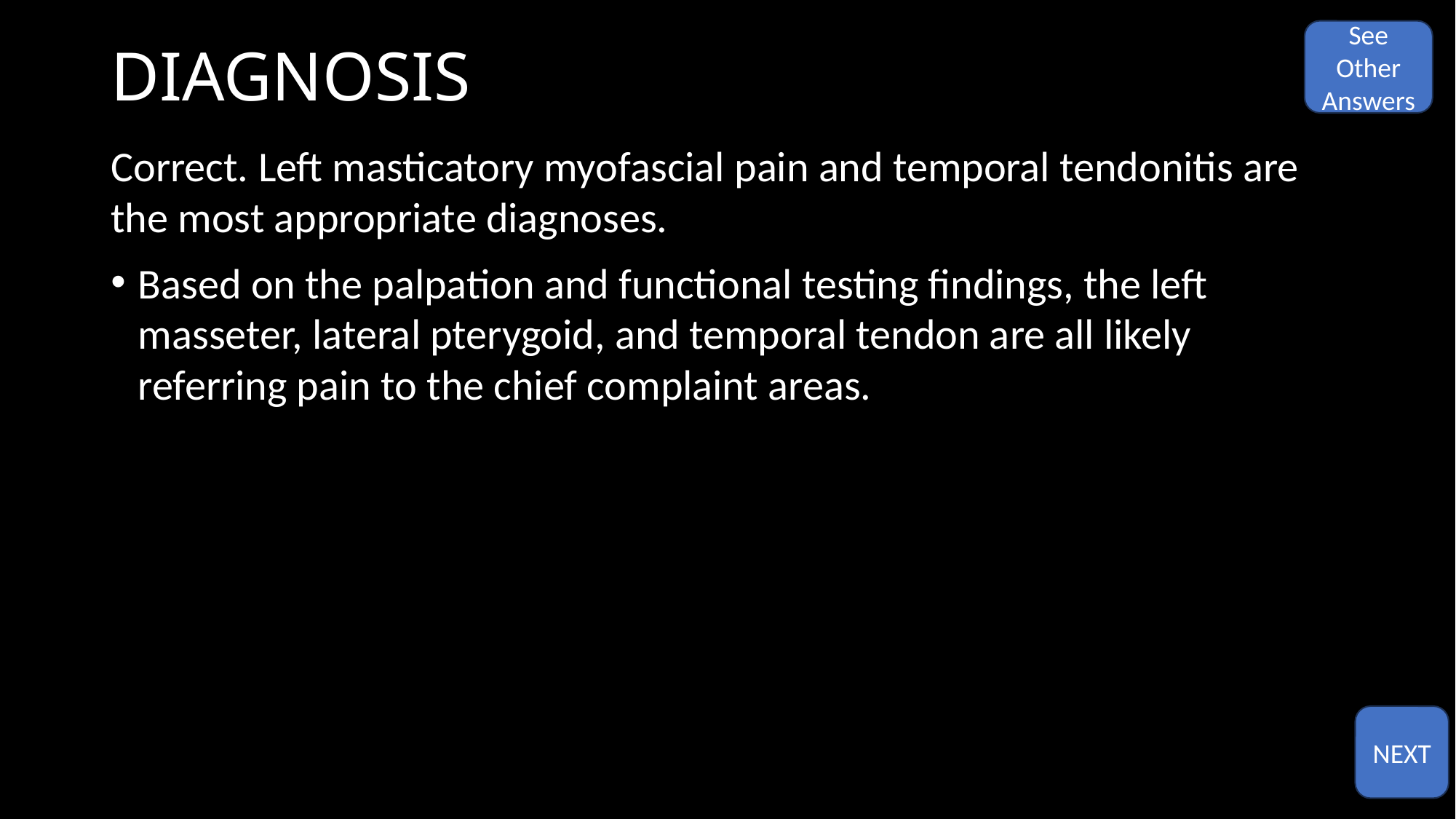

# DIAGNOSIS
See Other Answers
Correct. Left masticatory myofascial pain and temporal tendonitis are the most appropriate diagnoses.
Based on the palpation and functional testing findings, the left masseter, lateral pterygoid, and temporal tendon are all likely referring pain to the chief complaint areas.
NEXT

## Slide 102
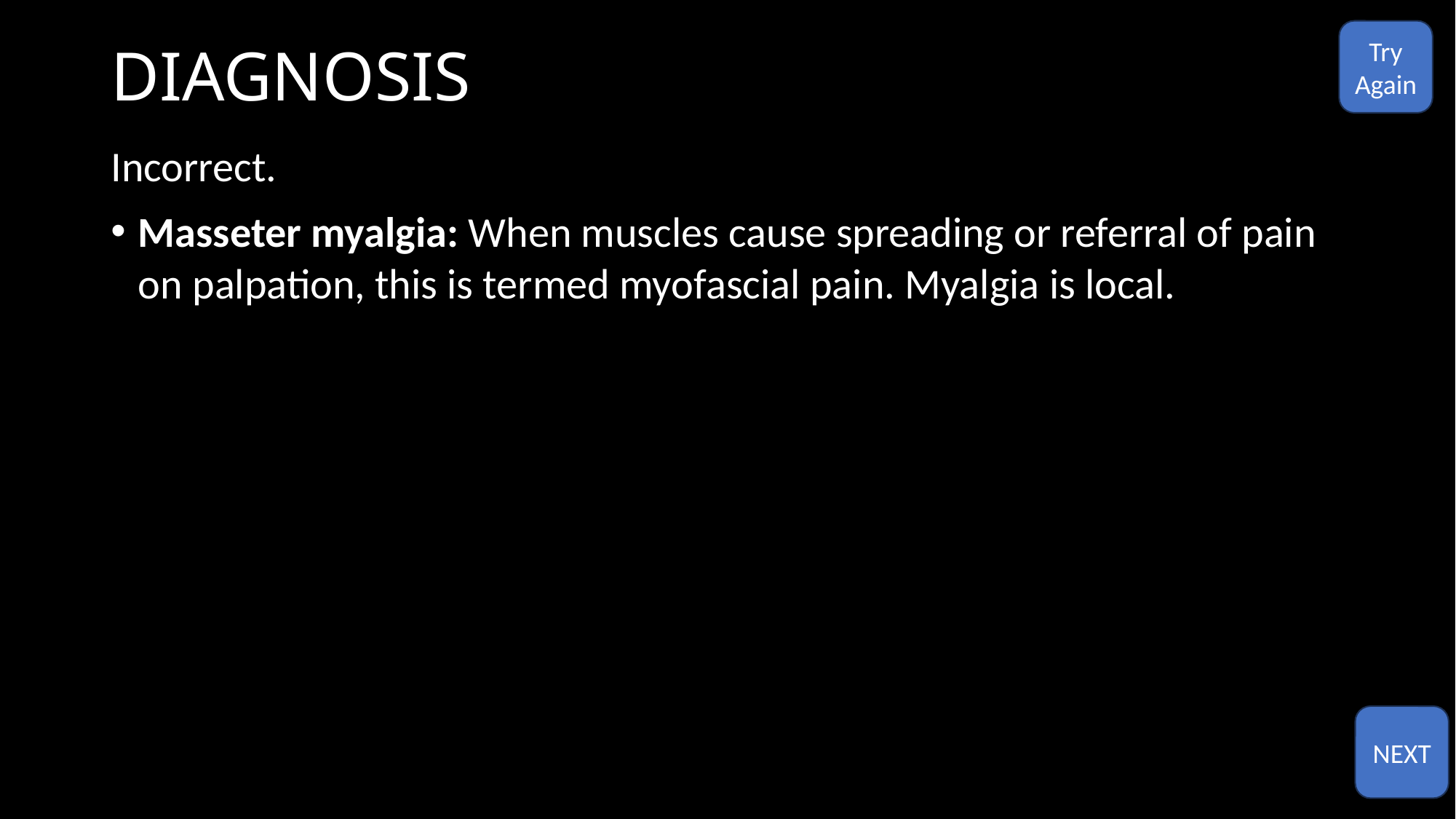

# DIAGNOSIS
Try
Again
Incorrect.
Masseter myalgia: When muscles cause spreading or referral of pain on palpation, this is termed myofascial pain. Myalgia is local.
NEXT

## Slide 103
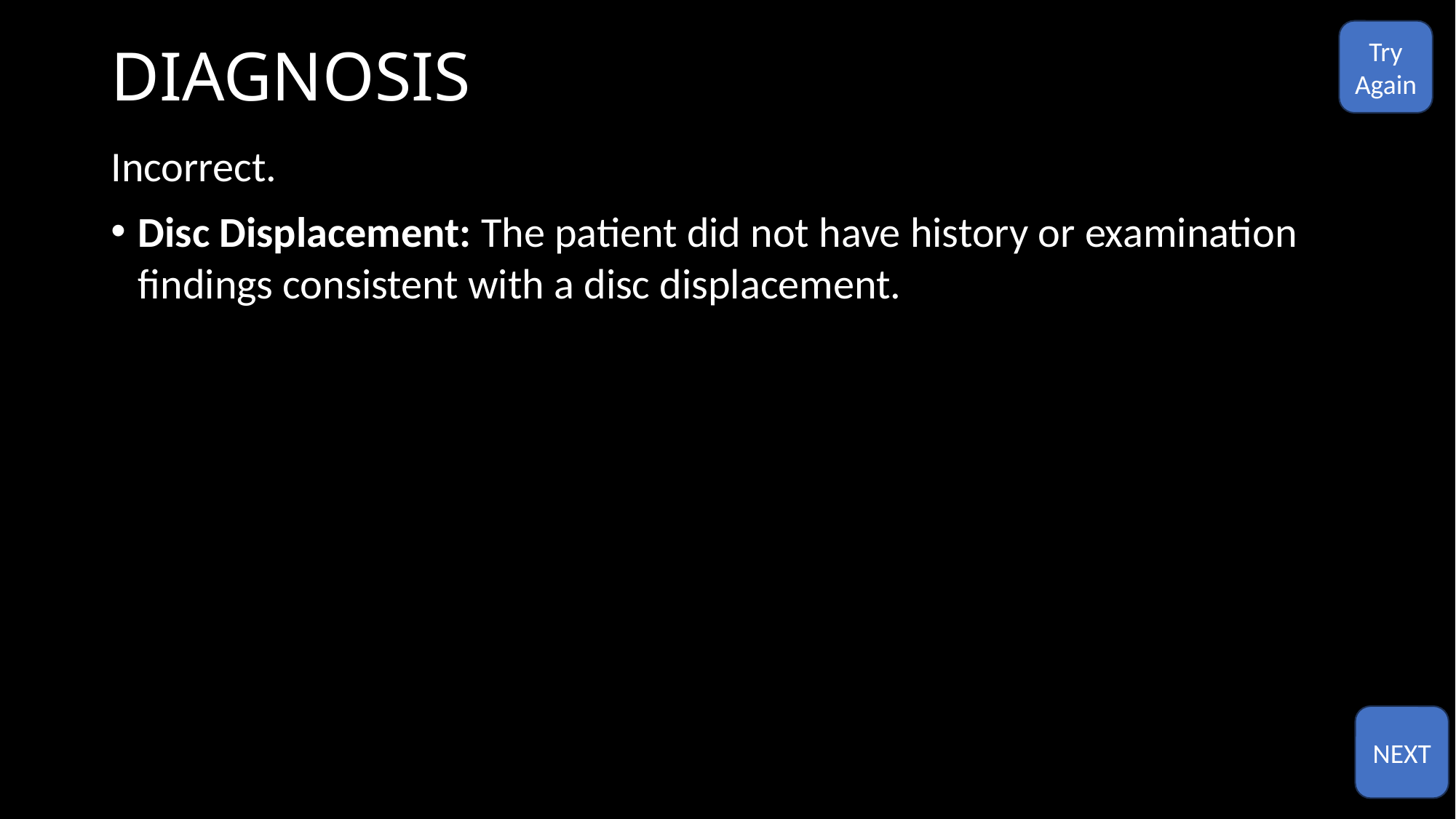

# DIAGNOSIS
Try
Again
Incorrect.
Disc Displacement: The patient did not have history or examination findings consistent with a disc displacement.
NEXT

## Slide 104
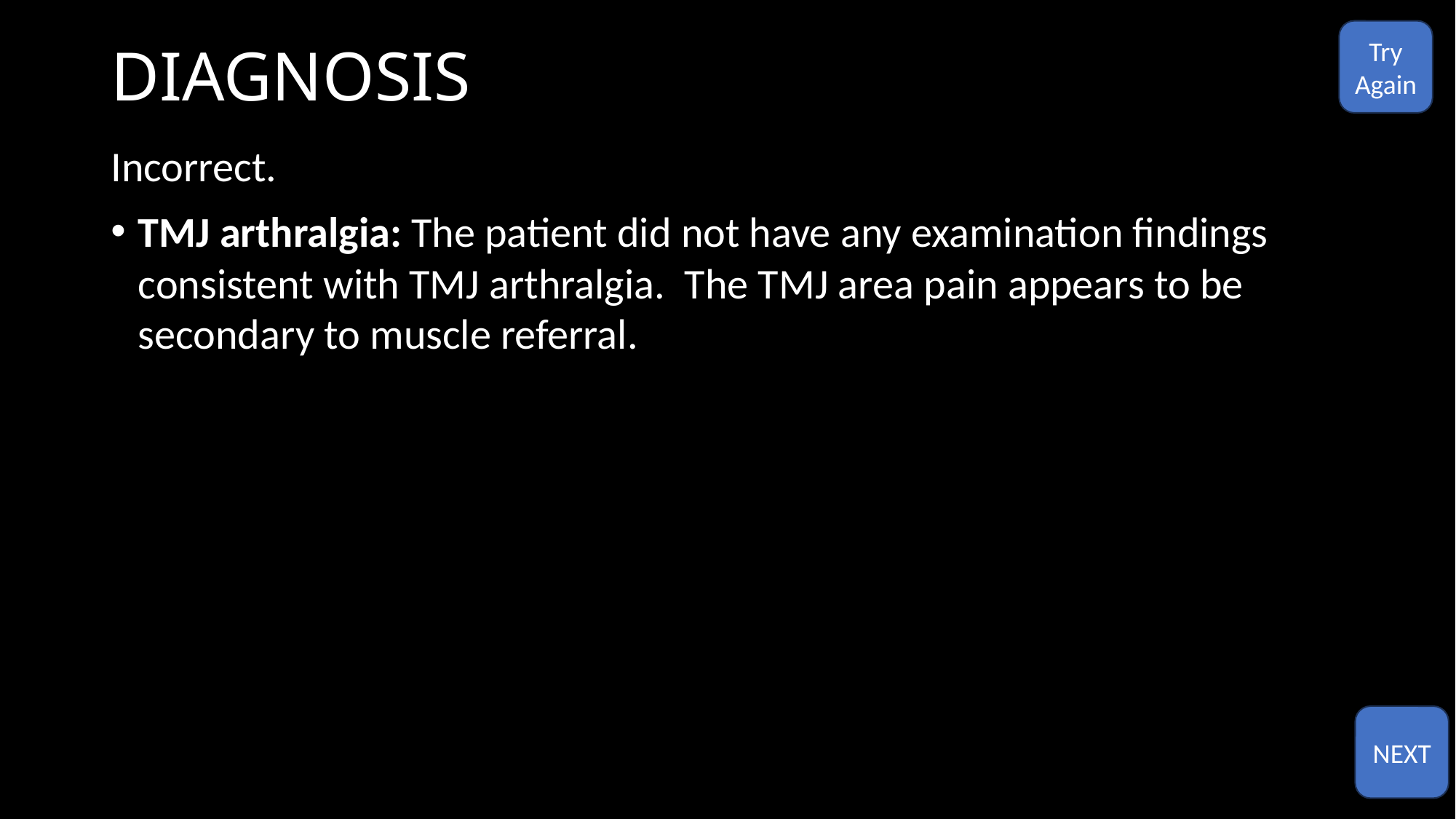

# DIAGNOSIS
Try
Again
Incorrect.
TMJ arthralgia: The patient did not have any examination findings consistent with TMJ arthralgia. The TMJ area pain appears to be secondary to muscle referral.
NEXT

## Slide 105
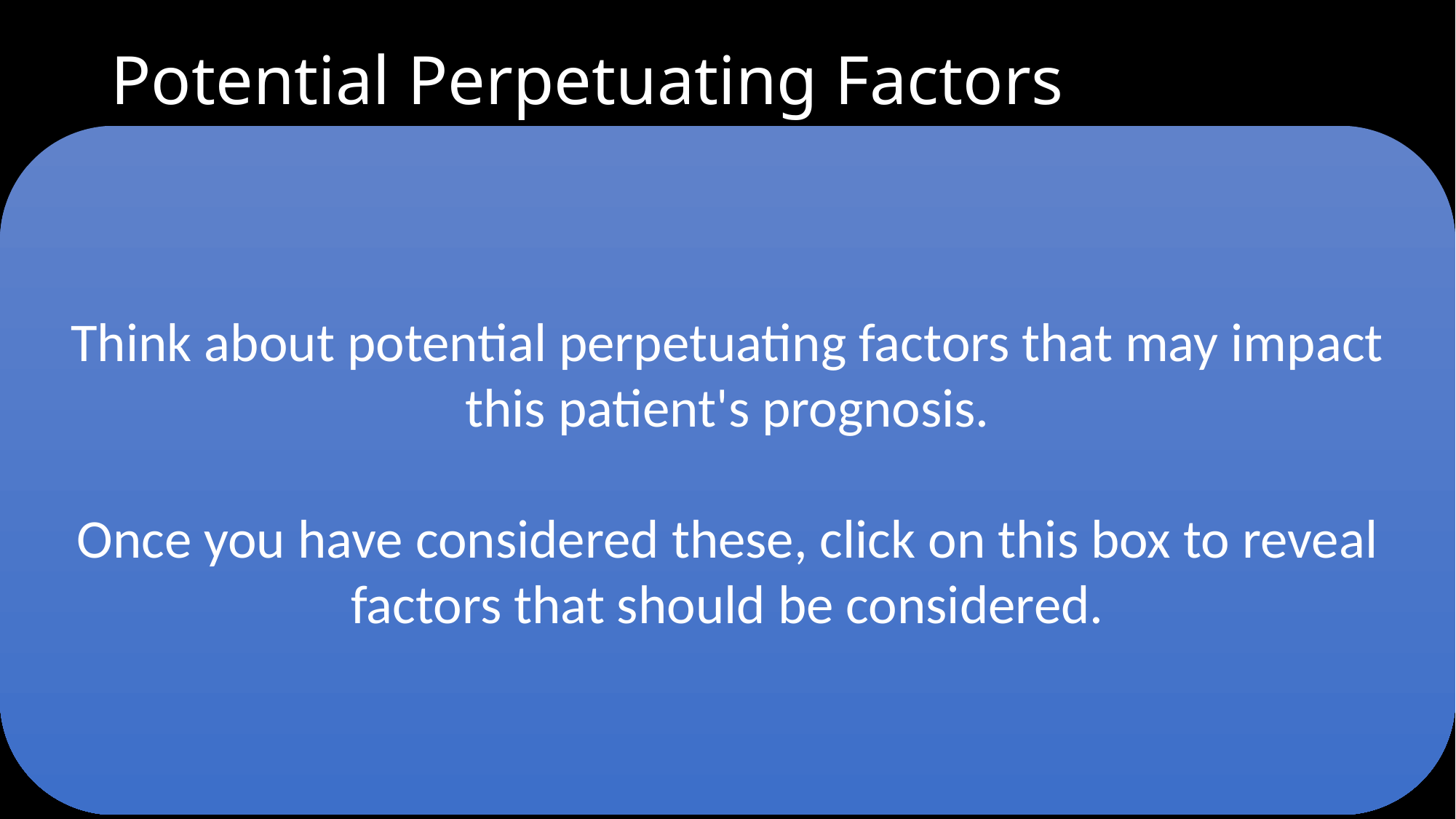

# Potential Perpetuating Factors
Think about potential perpetuating factors that may impact this patient's prognosis.
Once you have considered these, click on this box to reveal factors that should be considered.
Oral Parafunction - Clenching, jaw protrusion, fingernail biting
Depression history
Medication Use- Fluoxetine (side effect of oral parafunction?)
Headache History - Prolonged history of migraine headache
Nutrition
High soda (caffeine) consumption
Inadequate hydration
Proinflammatory food consumption

## Slide 106
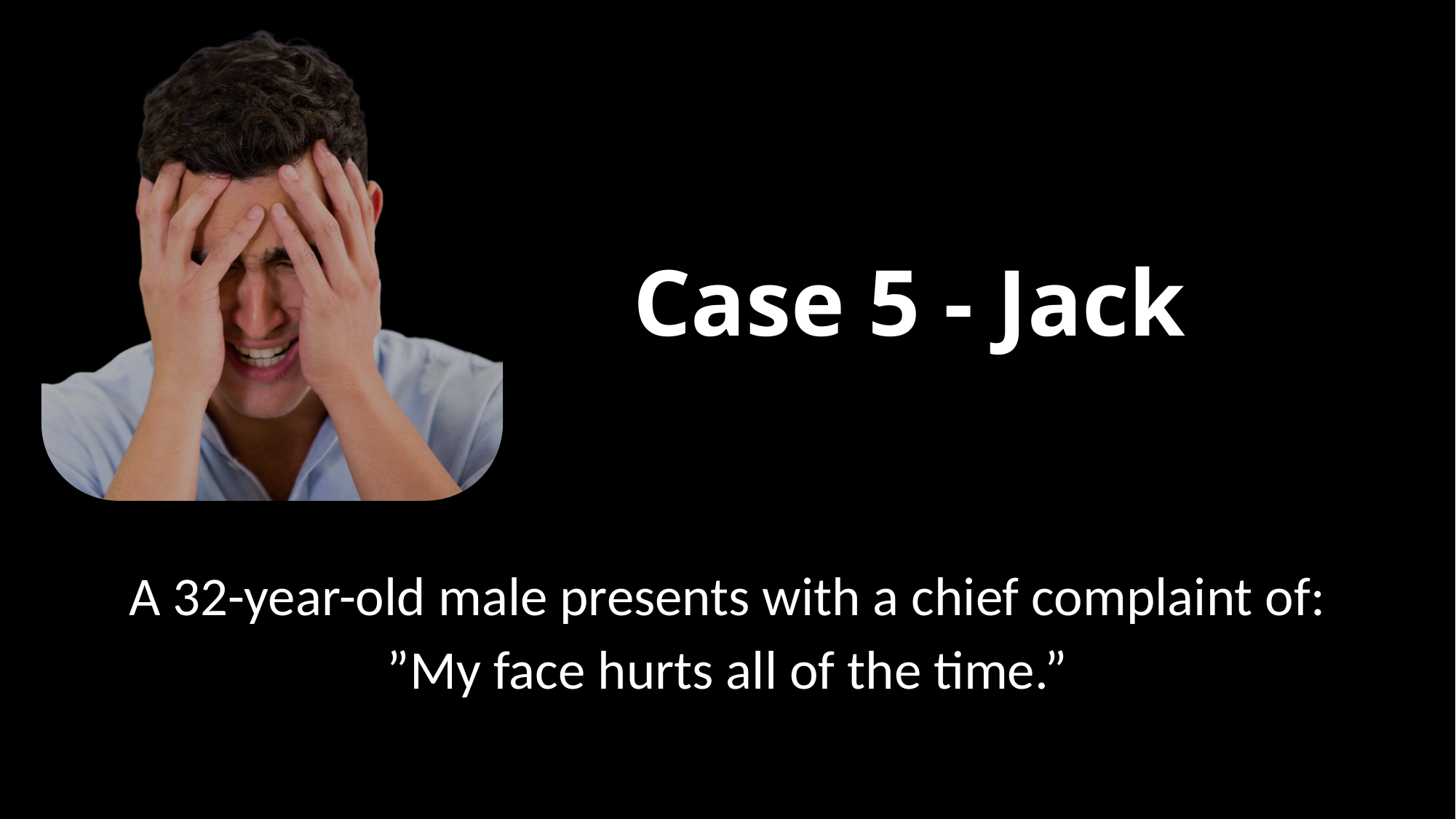

# Case 5 - Jack
A 32-year-old male presents with a chief complaint of:
”My face hurts all of the time.”

## Slide 107
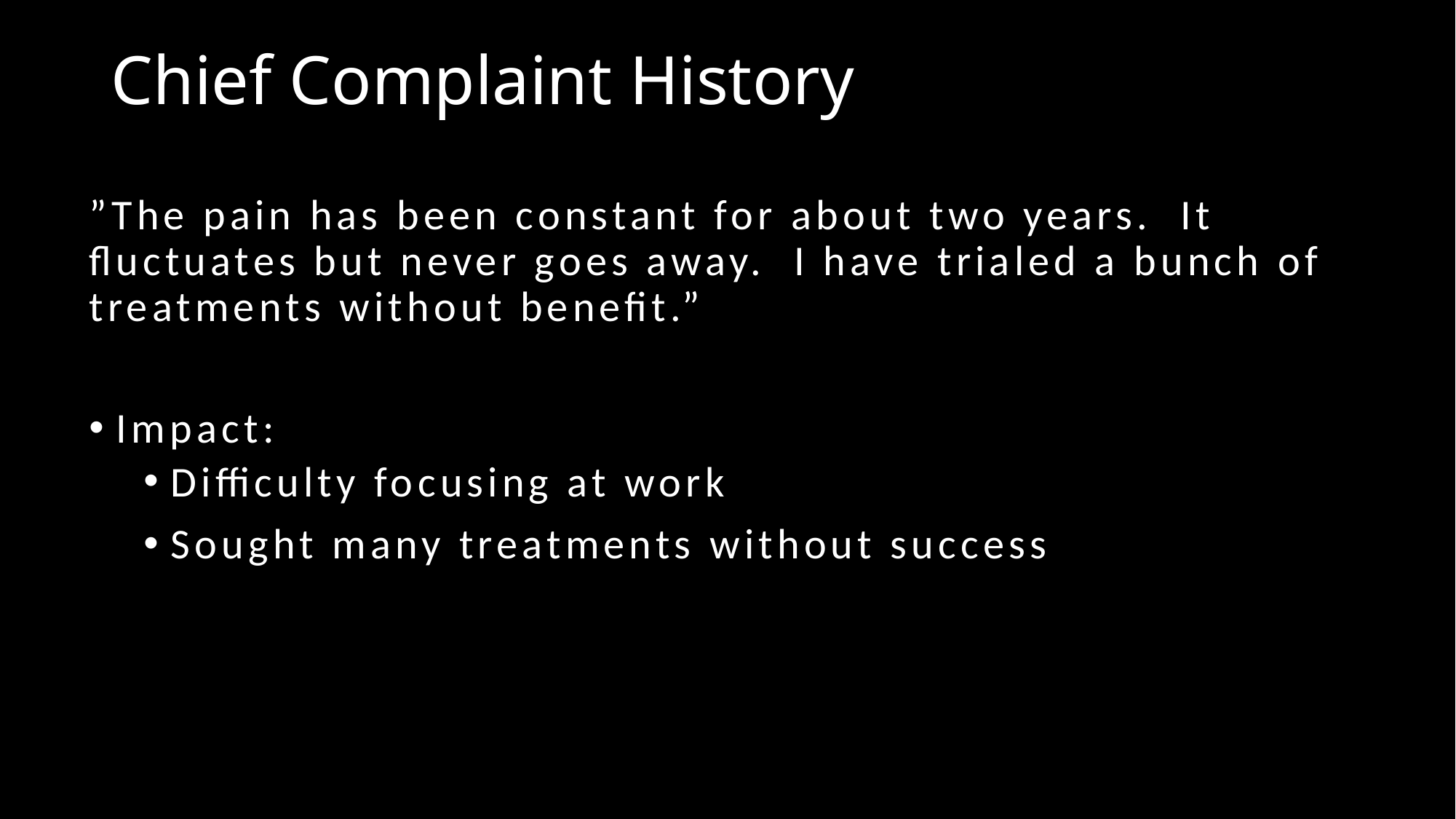

# Chief Complaint History
”The pain has been constant for about two years. It fluctuates but never goes away. I have trialed a bunch of treatments without benefit.”
Impact:
Difficulty focusing at work
Sought many treatments without success

## Slide 108
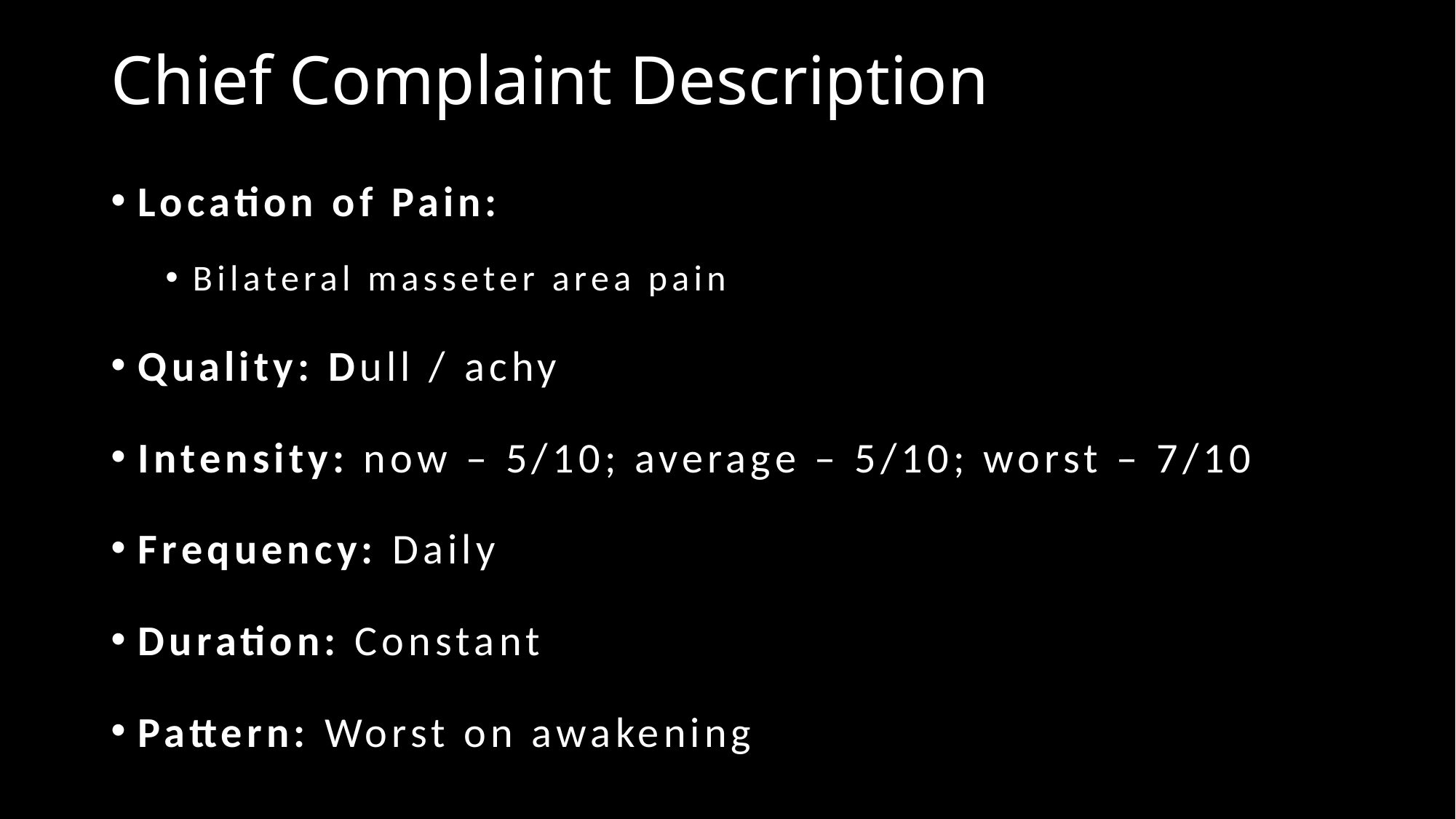

# Chief Complaint Description
Location of Pain:
Bilateral masseter area pain
Quality: Dull / achy
Intensity: now – 5/10; average – 5/10; worst – 7/10
Frequency: Daily
Duration: Constant
Pattern: Worst on awakening

## Slide 109
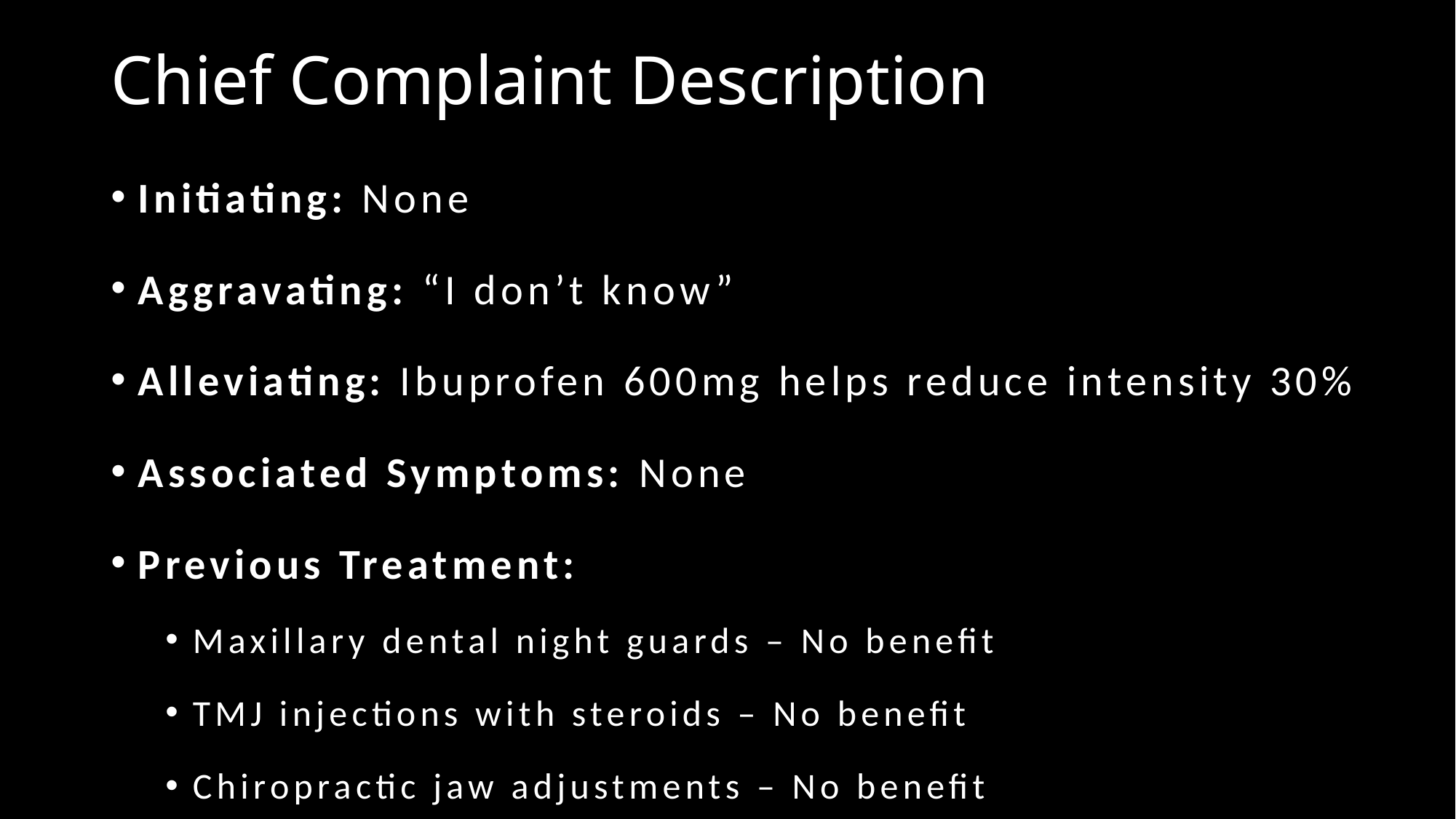

# Chief Complaint Description
Initiating: None
Aggravating: “I don’t know”
Alleviating: Ibuprofen 600mg helps reduce intensity 30%
Associated Symptoms: None
Previous Treatment:
Maxillary dental night guards – No benefit
TMJ injections with steroids – No benefit
Chiropractic jaw adjustments – No benefit

## Slide 110
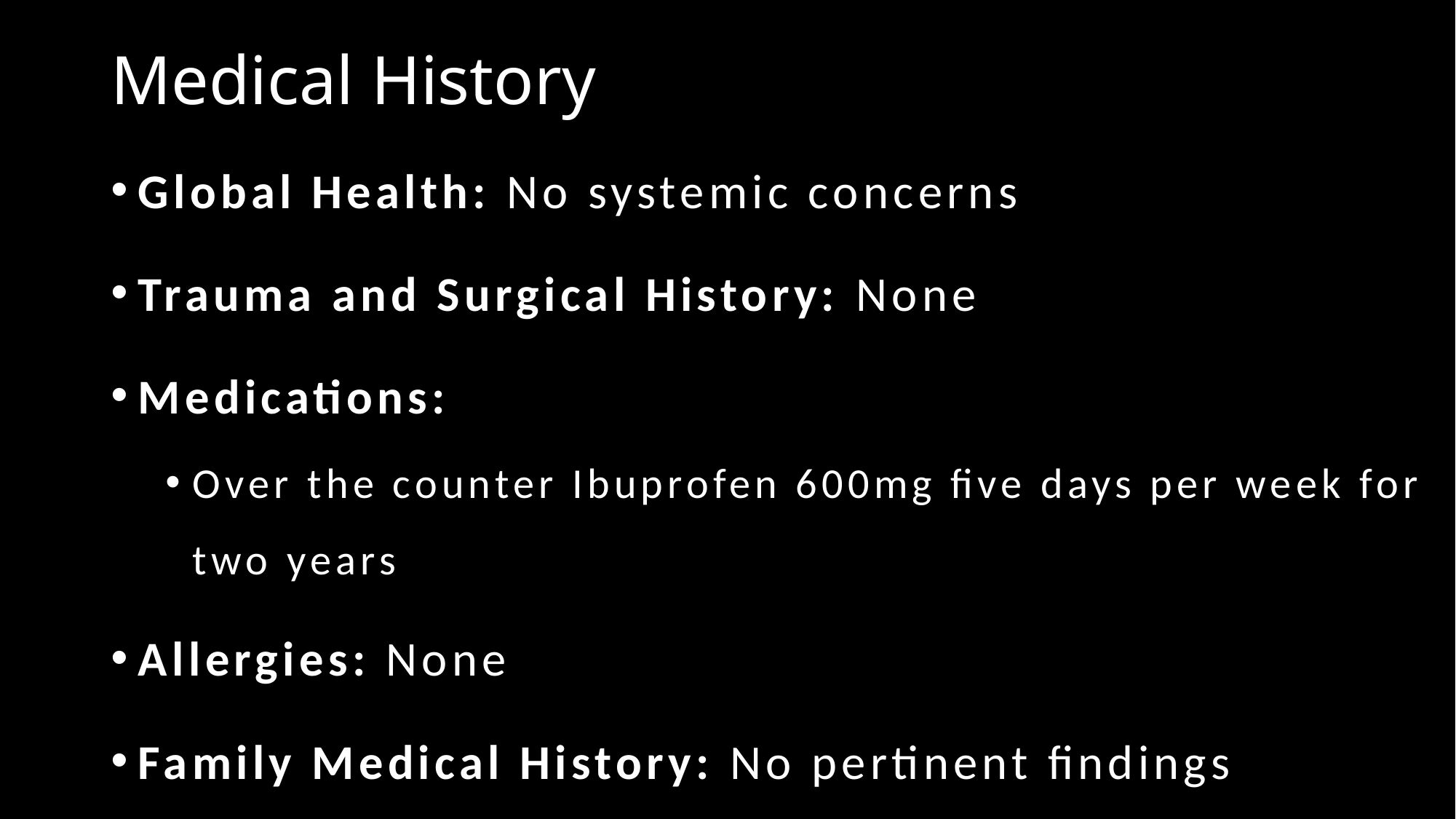

# Medical History
Global Health: No systemic concerns
Trauma and Surgical History: None
Medications:
Over the counter Ibuprofen 600mg five days per week for two years
Allergies: None
Family Medical History: No pertinent findings

## Slide 111
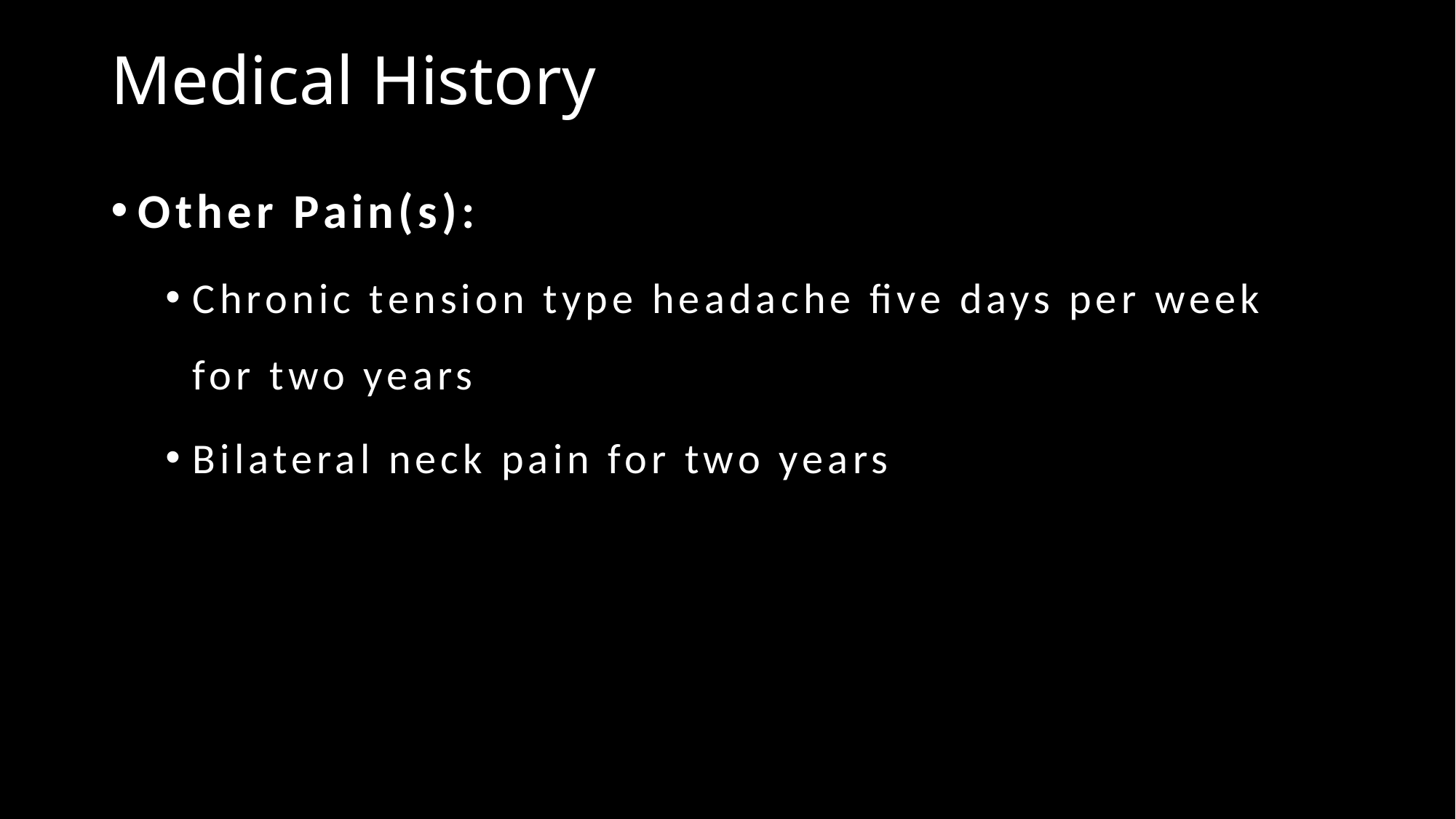

# Medical History
Other Pain(s):
Chronic tension type headache five days per week for two years
Bilateral neck pain for two years

## Slide 112
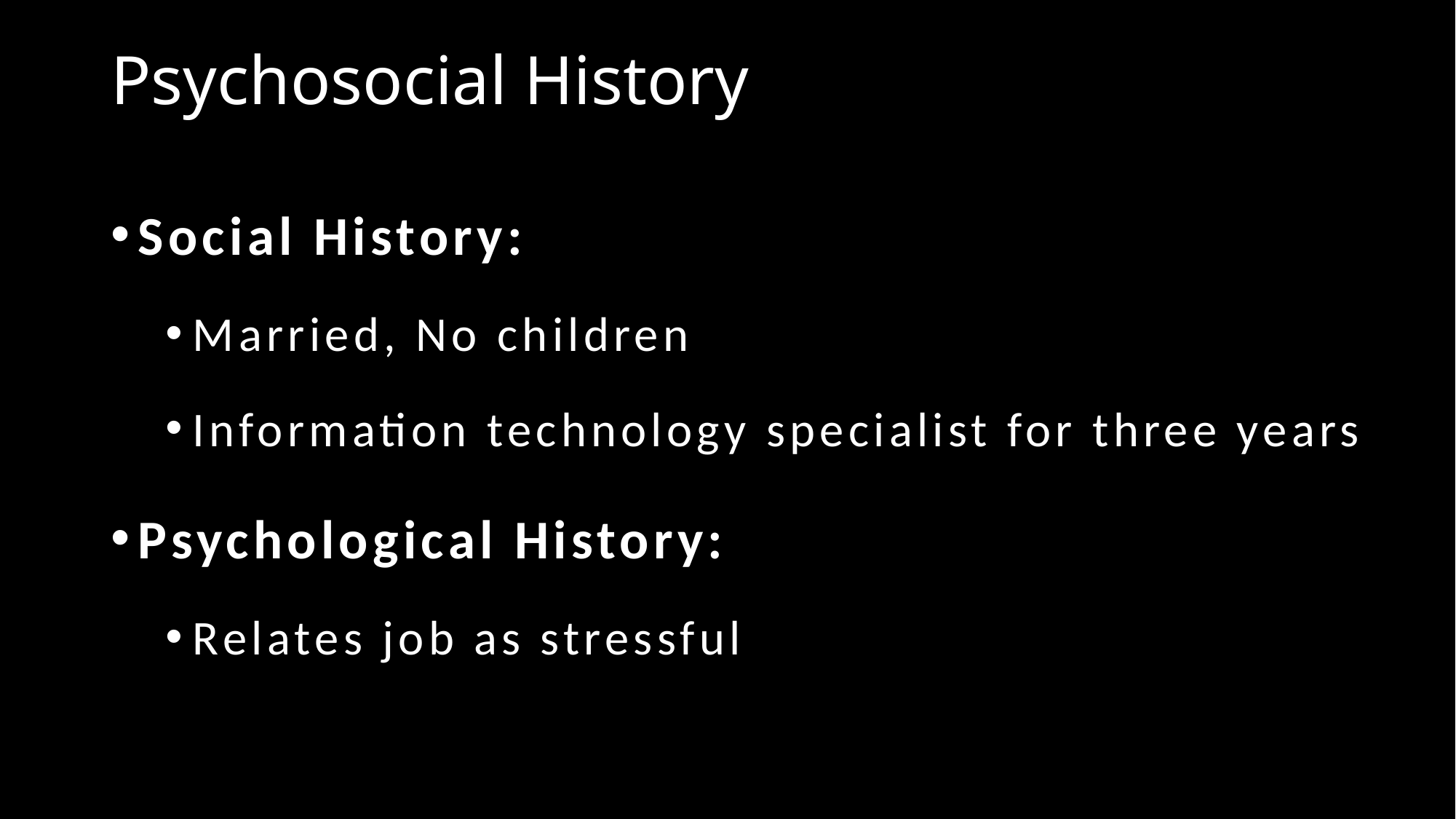

# Psychosocial History
Social History:
Married, No children
Information technology specialist for three years
Psychological History:
Relates job as stressful

## Slide 113
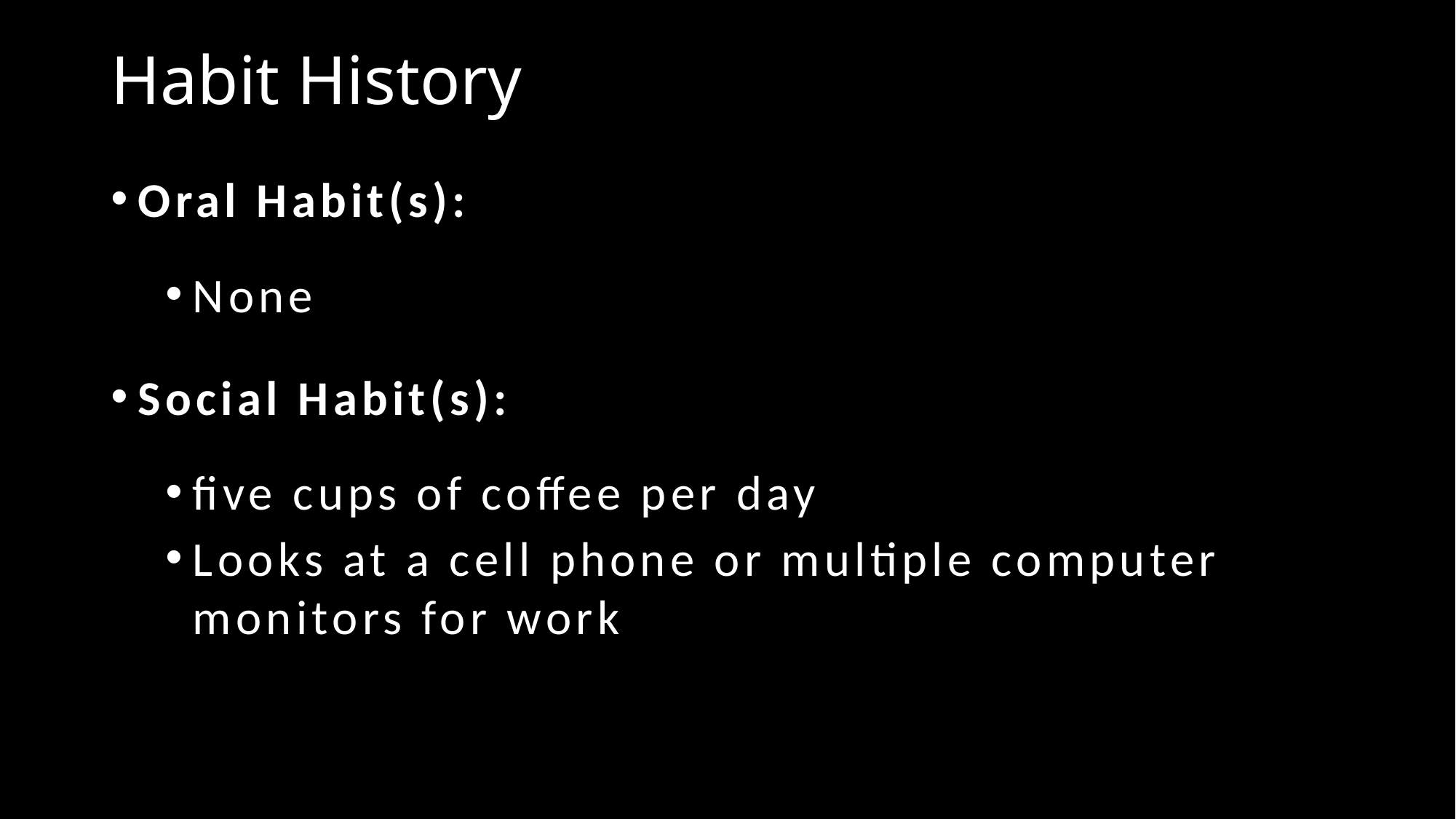

# Habit History
Oral Habit(s):
None
Social Habit(s):
five cups of coffee per day
Looks at a cell phone or multiple computer monitors for work

## Slide 114
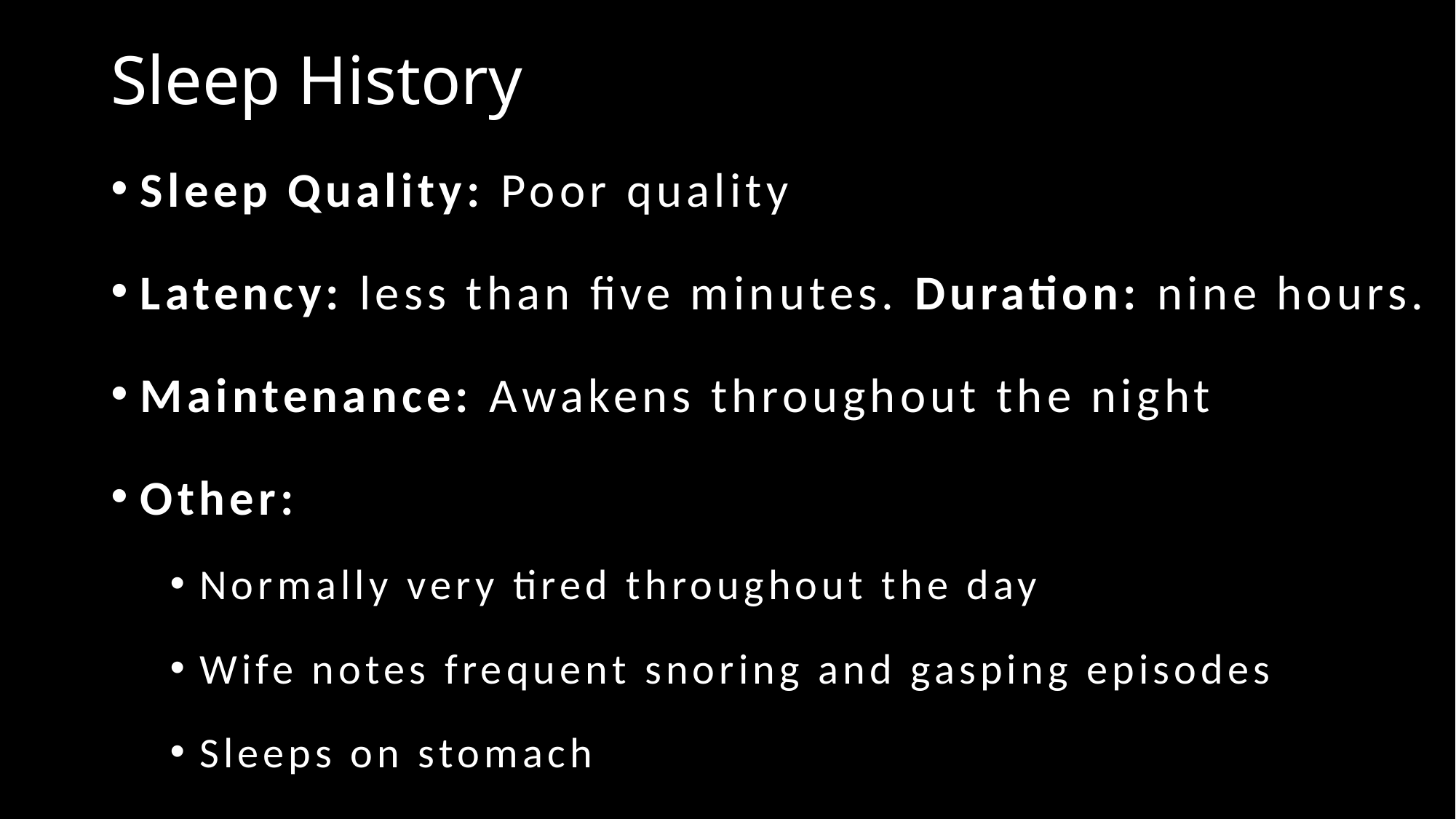

# Sleep History
Sleep Quality: Poor quality
Latency: less than five minutes. Duration: nine hours.
Maintenance: Awakens throughout the night
Other:
Normally very tired throughout the day
Wife notes frequent snoring and gasping episodes
Sleeps on stomach

## Slide 115
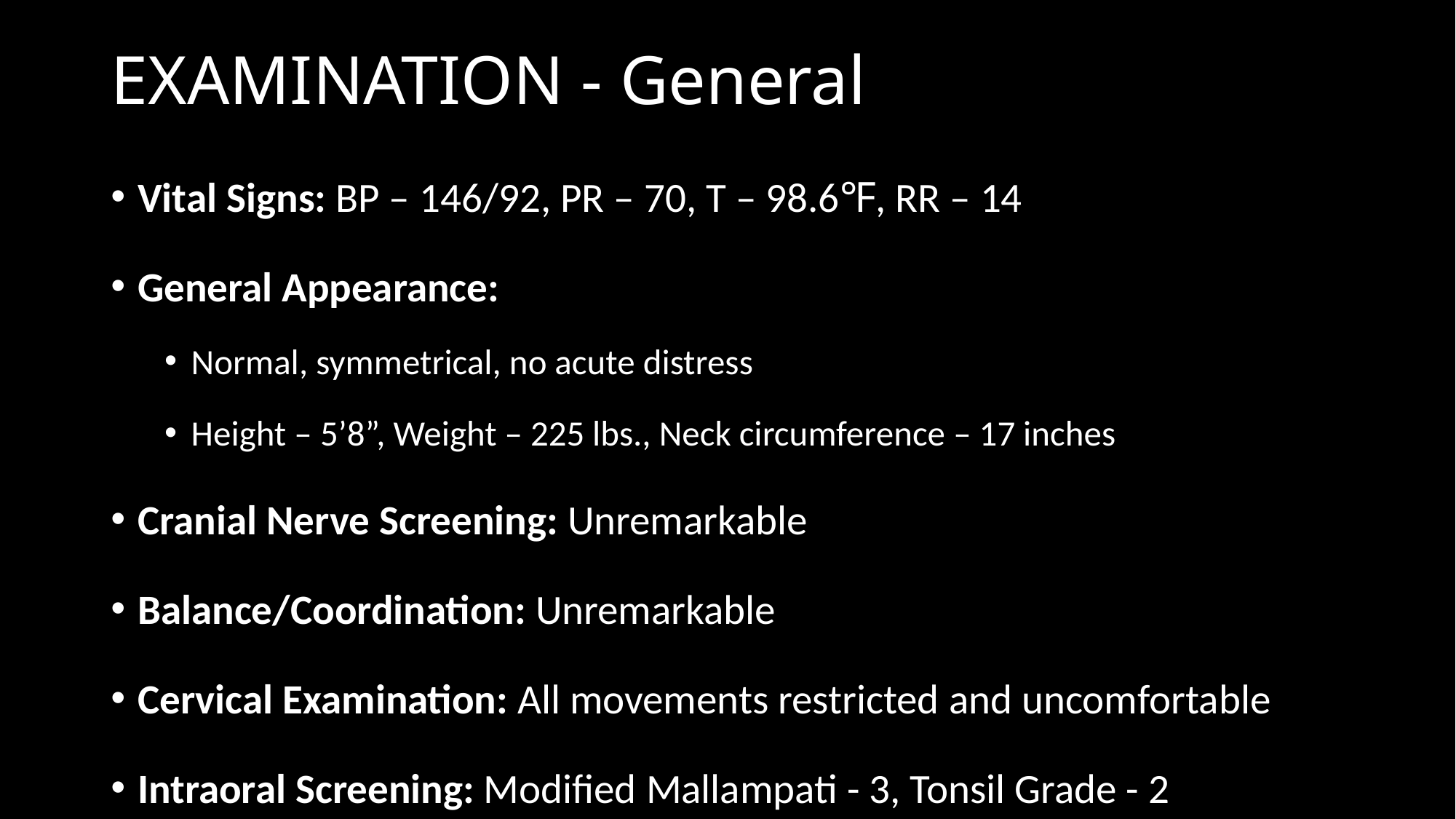

# EXAMINATION - General
Vital Signs: BP – 146/92, PR – 70, T – 98.6℉, RR – 14
General Appearance:
Normal, symmetrical, no acute distress
Height – 5’8”, Weight – 225 lbs., Neck circumference – 17 inches
Cranial Nerve Screening: Unremarkable
Balance/Coordination: Unremarkable
Cervical Examination: All movements restricted and uncomfortable
Intraoral Screening: Modified Mallampati - 3, Tonsil Grade - 2

## Slide 116
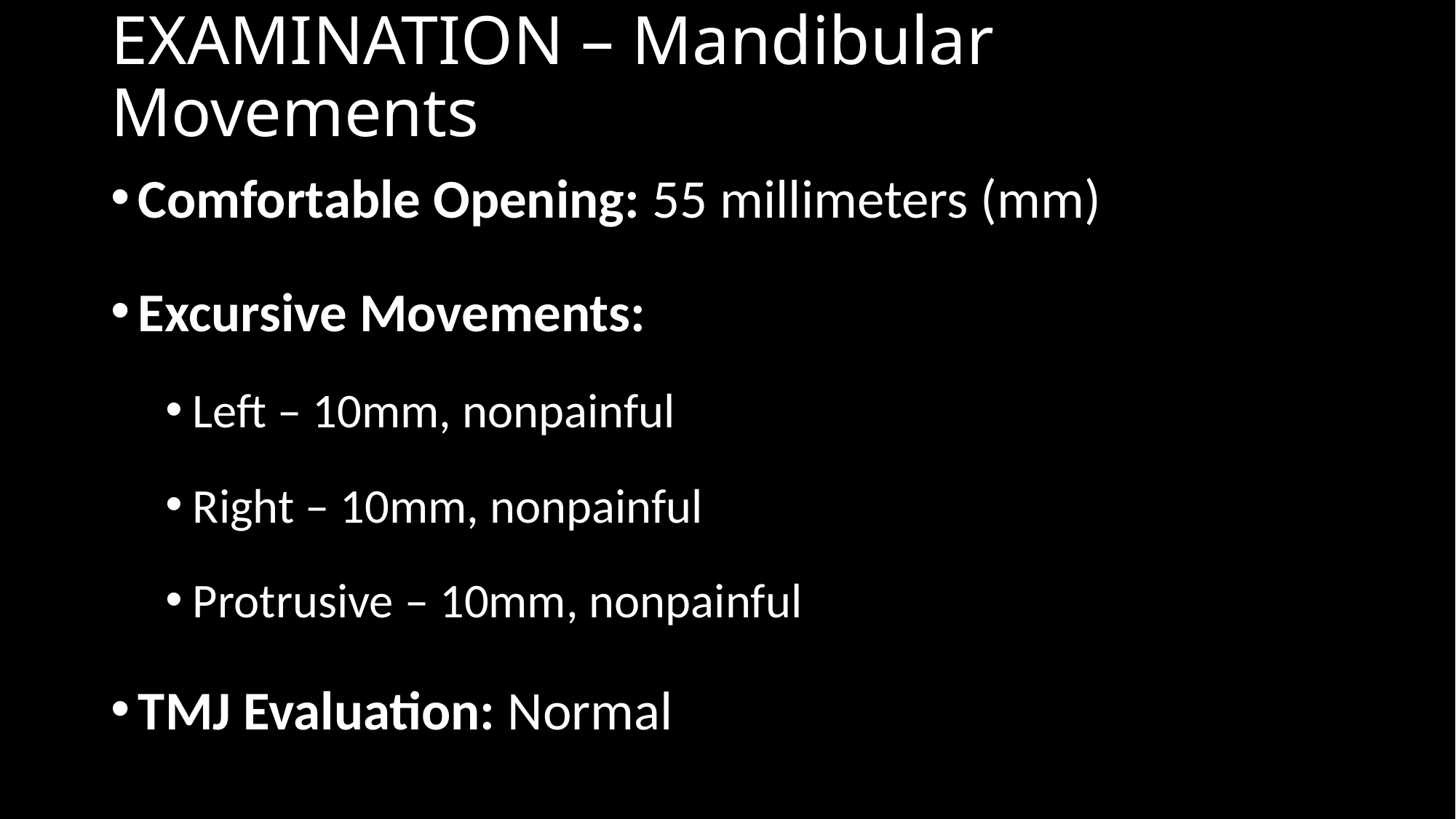

# EXAMINATION – Mandibular Movements
Comfortable Opening: 55 millimeters (mm)
Excursive Movements:
Left – 10mm, nonpainful
Right – 10mm, nonpainful
Protrusive – 10mm, nonpainful
TMJ Evaluation: Normal

## Slide 117
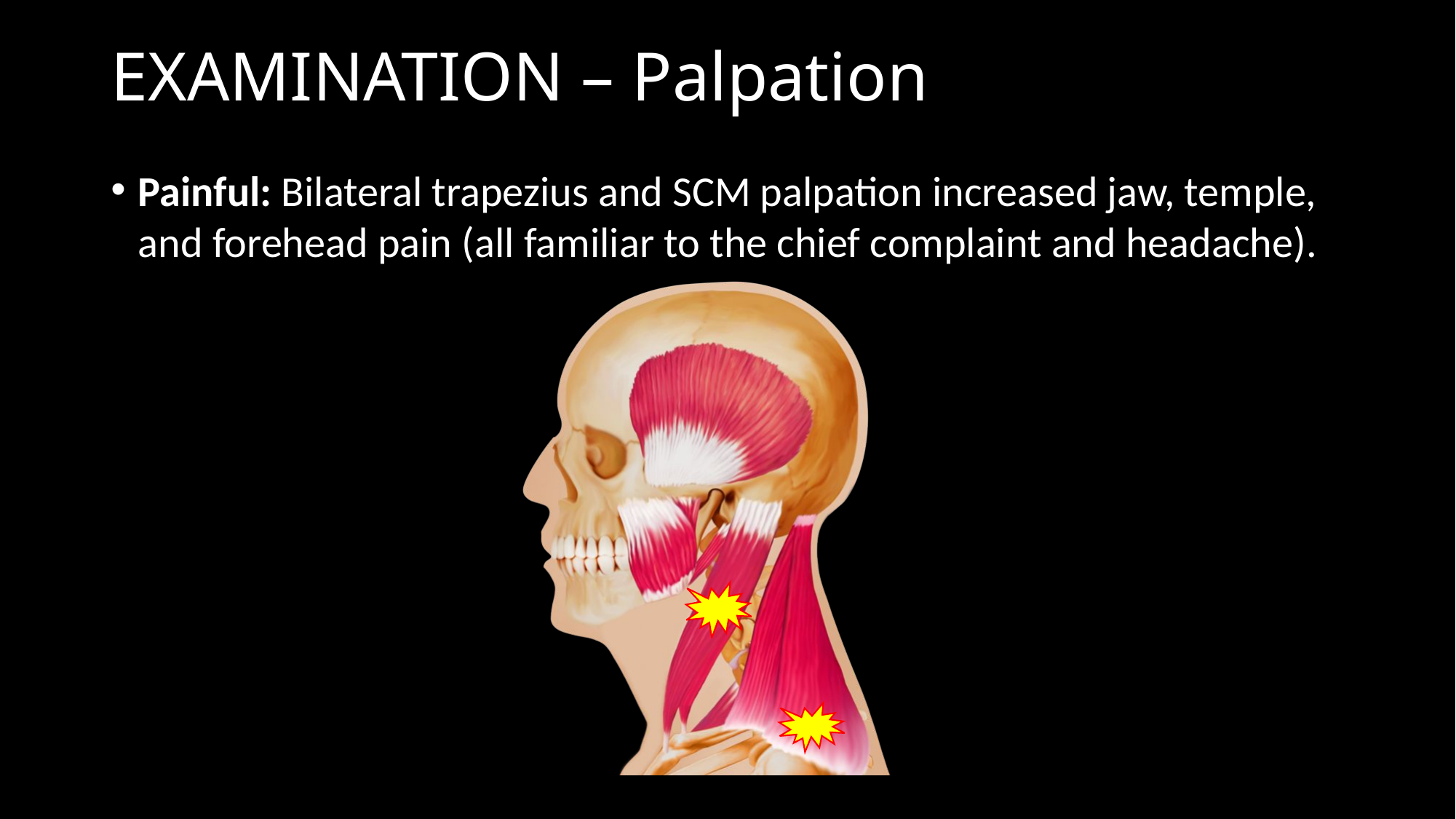

# EXAMINATION – Palpation
Painful: Bilateral trapezius and SCM palpation increased jaw, temple, and forehead pain (all familiar to the chief complaint and headache).

## Slide 118
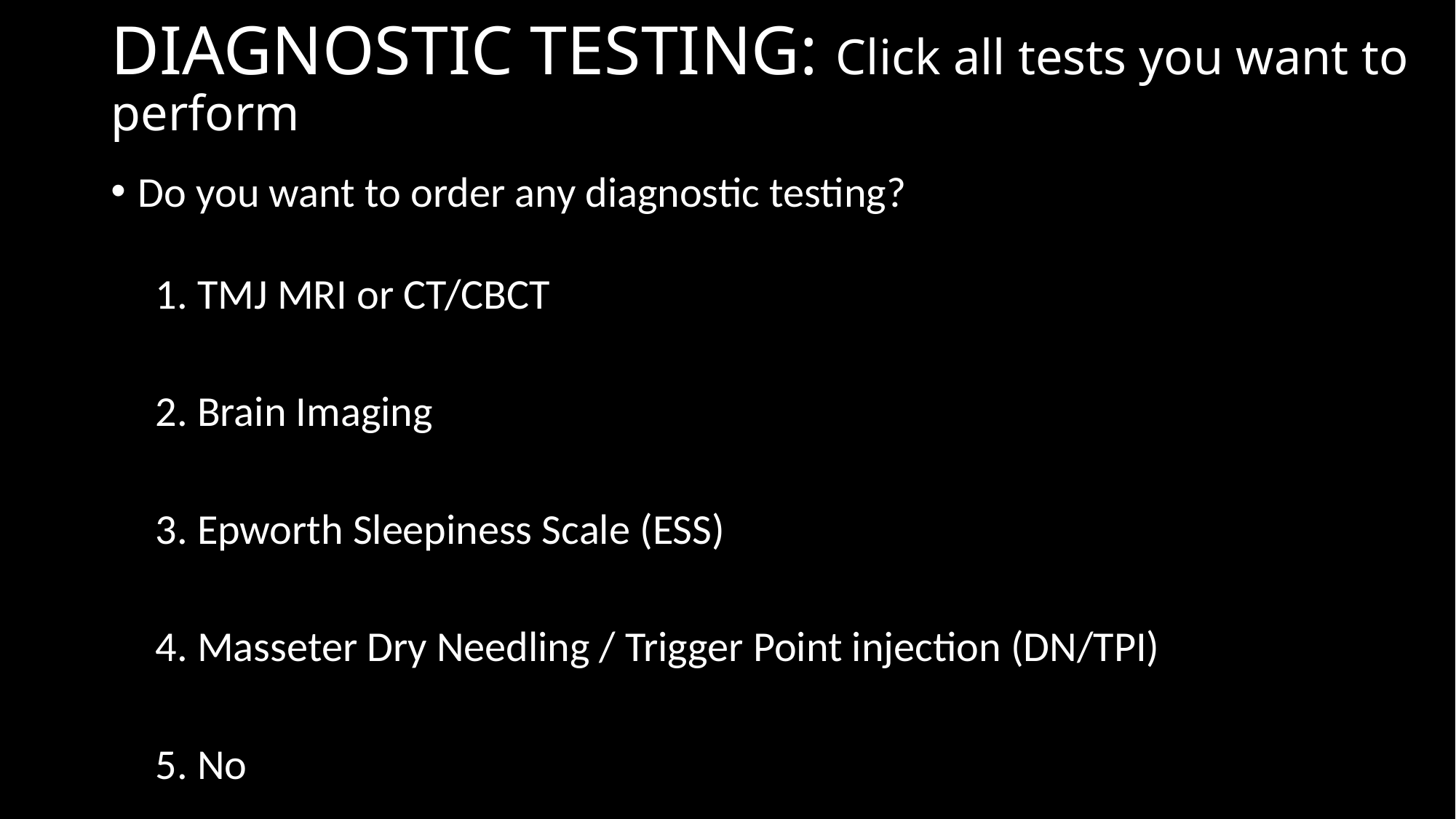

DIAGNOSTIC TESTING: Click all tests you want to perform
Do you want to order any diagnostic testing?
1. TMJ MRI or CT/CBCT
2. Brain Imaging
3. Epworth Sleepiness Scale (ESS)
4. Masseter Dry Needling / Trigger Point injection (DN/TPI)
5. No

## Slide 119
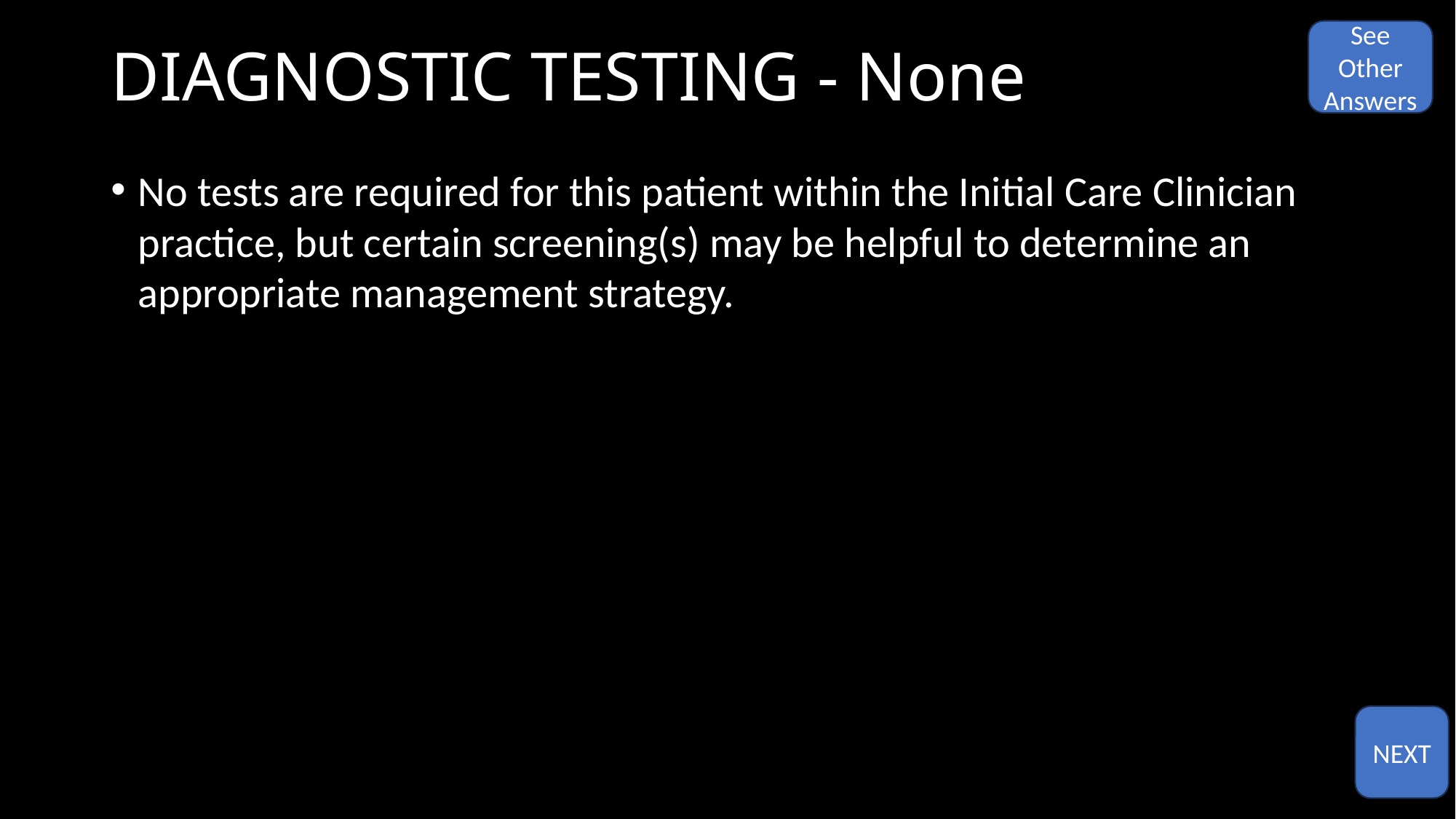

# DIAGNOSTIC TESTING - None
See Other Answers
No tests are required for this patient within the Initial Care Clinician practice, but certain screening(s) may be helpful to determine an appropriate management strategy.
NEXT

## Slide 120
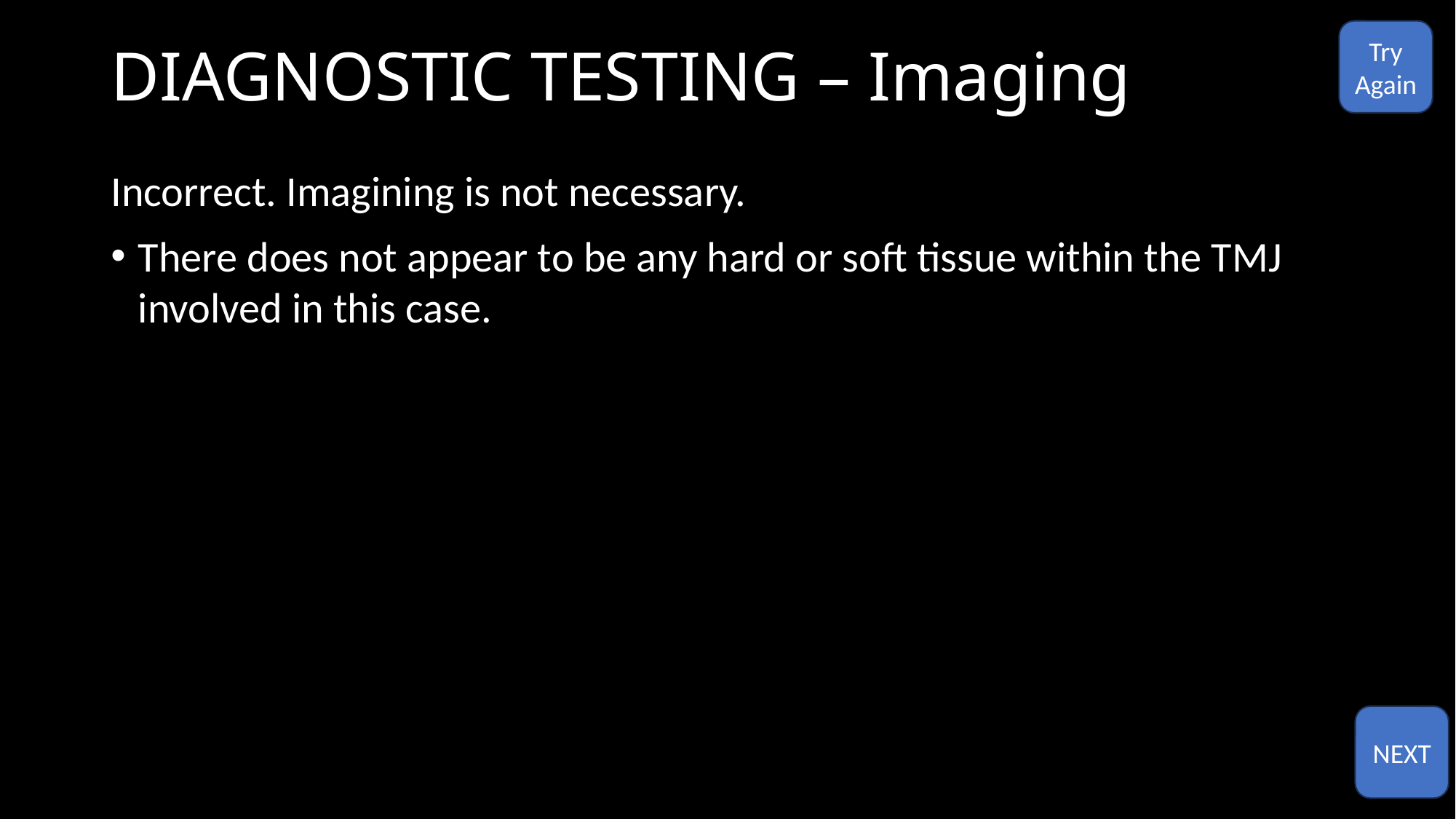

# DIAGNOSTIC TESTING – Imaging
Try
Again
Incorrect. Imagining is not necessary.
There does not appear to be any hard or soft tissue within the TMJ involved in this case.
NEXT

## Slide 121
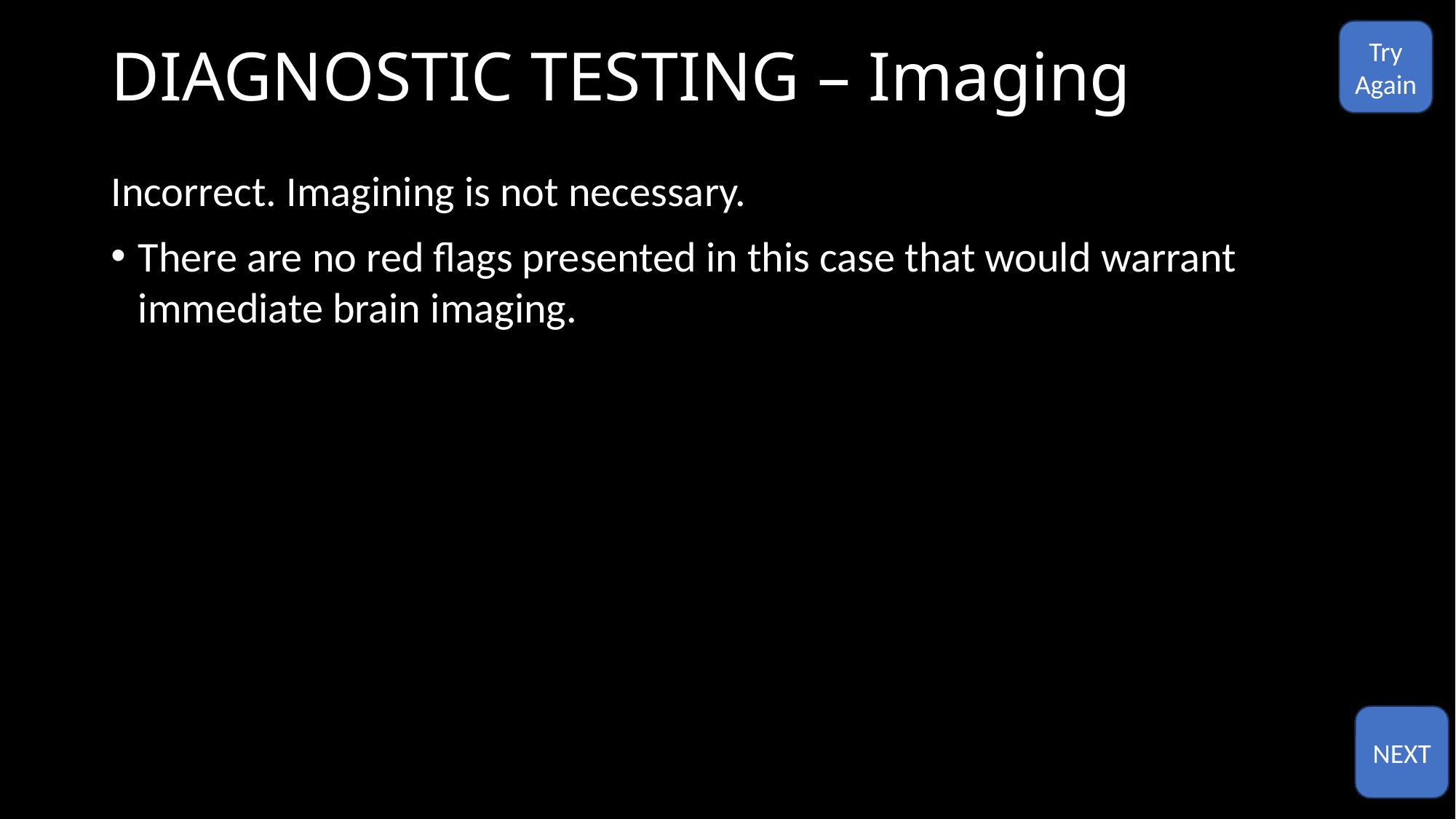

# DIAGNOSTIC TESTING – Imaging
Try
Again
Incorrect. Imagining is not necessary.
There are no red flags presented in this case that would warrant immediate brain imaging.
NEXT

## Slide 122
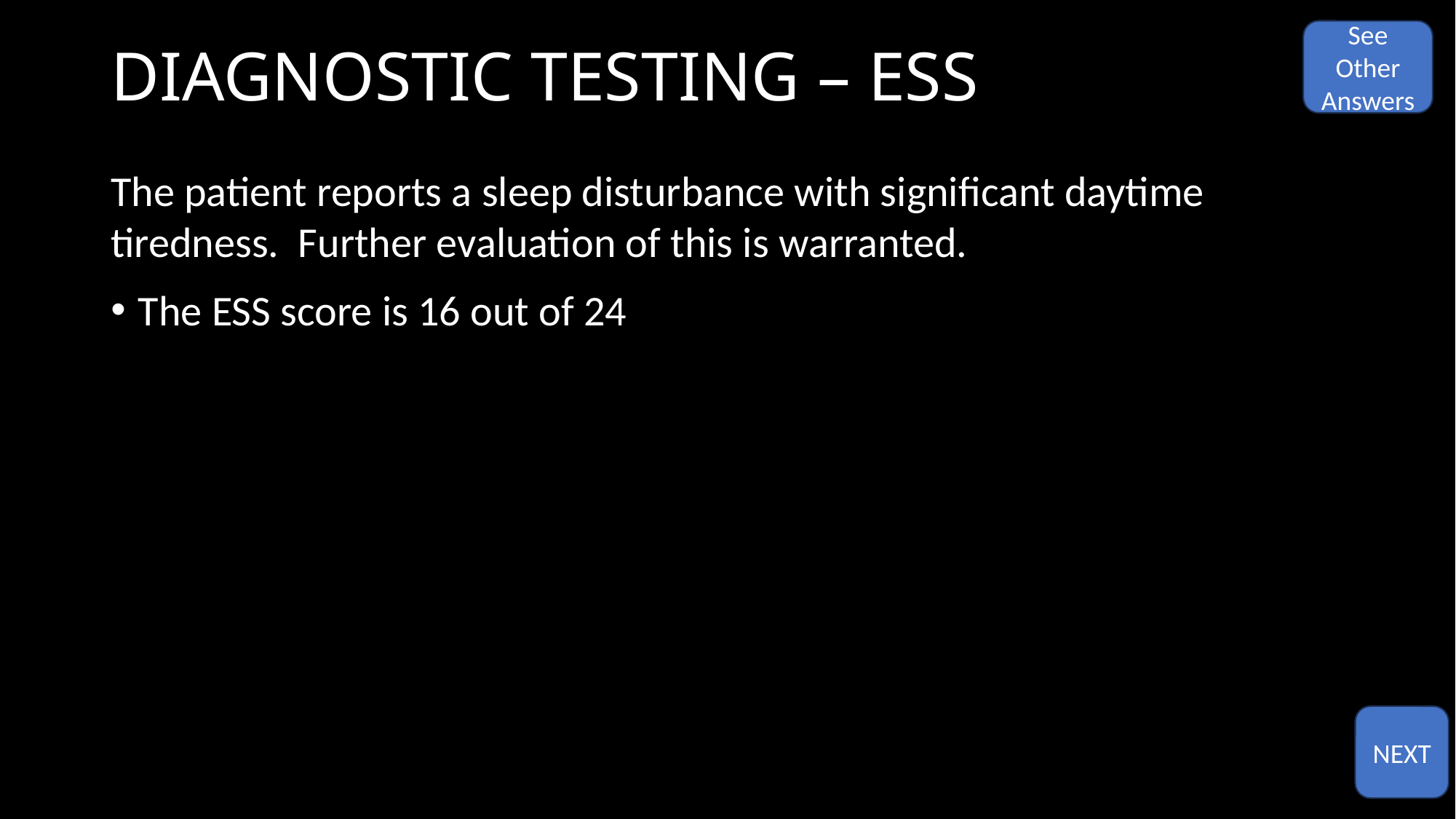

# DIAGNOSTIC TESTING – ESS
See Other Answers
The patient reports a sleep disturbance with significant daytime tiredness. Further evaluation of this is warranted.
The ESS score is 16 out of 24
NEXT

## Slide 123
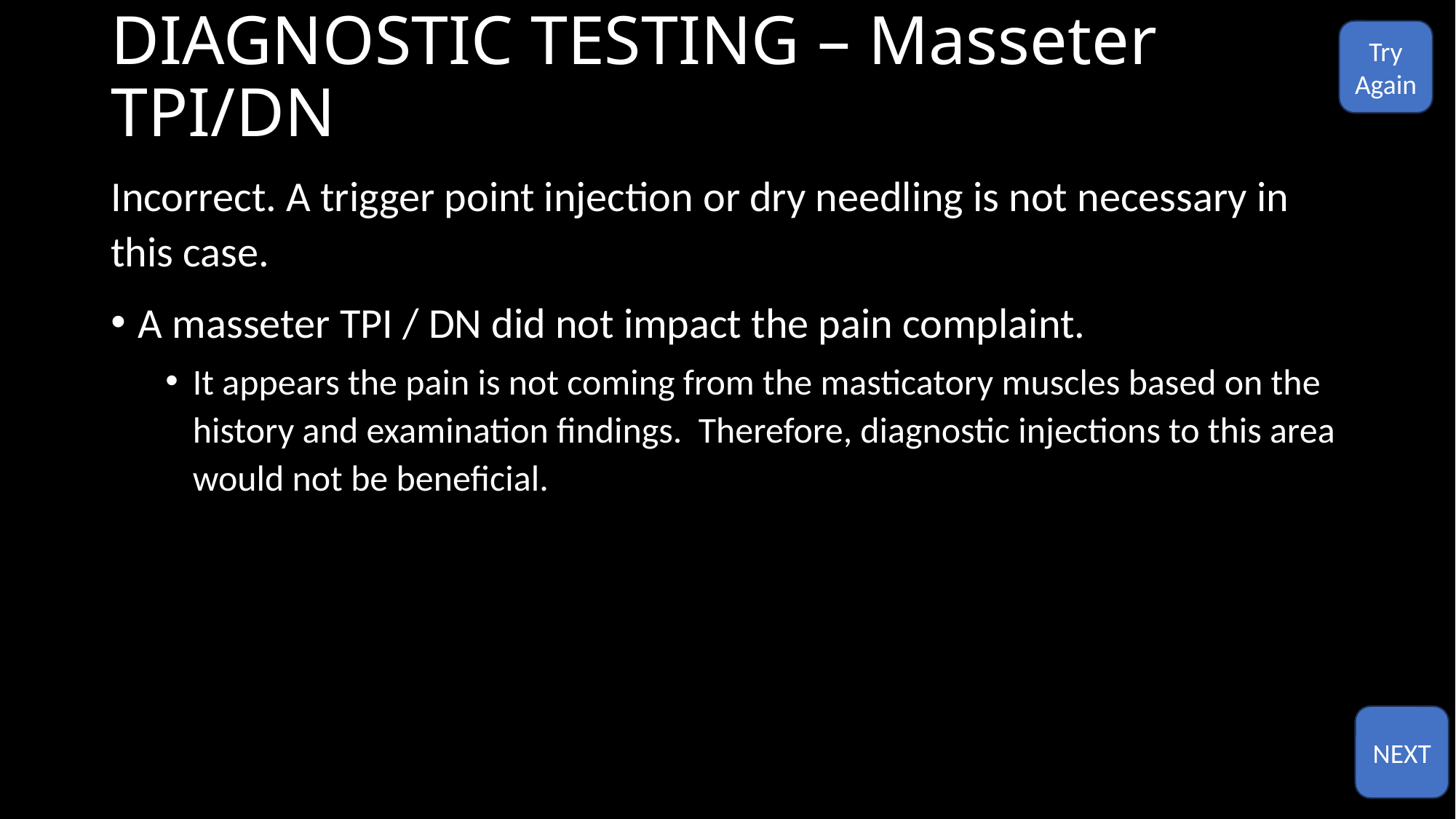

# DIAGNOSTIC TESTING – Masseter TPI/DN
Try
Again
Incorrect. A trigger point injection or dry needling is not necessary in this case.
A masseter TPI / DN did not impact the pain complaint.
It appears the pain is not coming from the masticatory muscles based on the history and examination findings. Therefore, diagnostic injections to this area would not be beneficial.
NEXT

## Slide 124
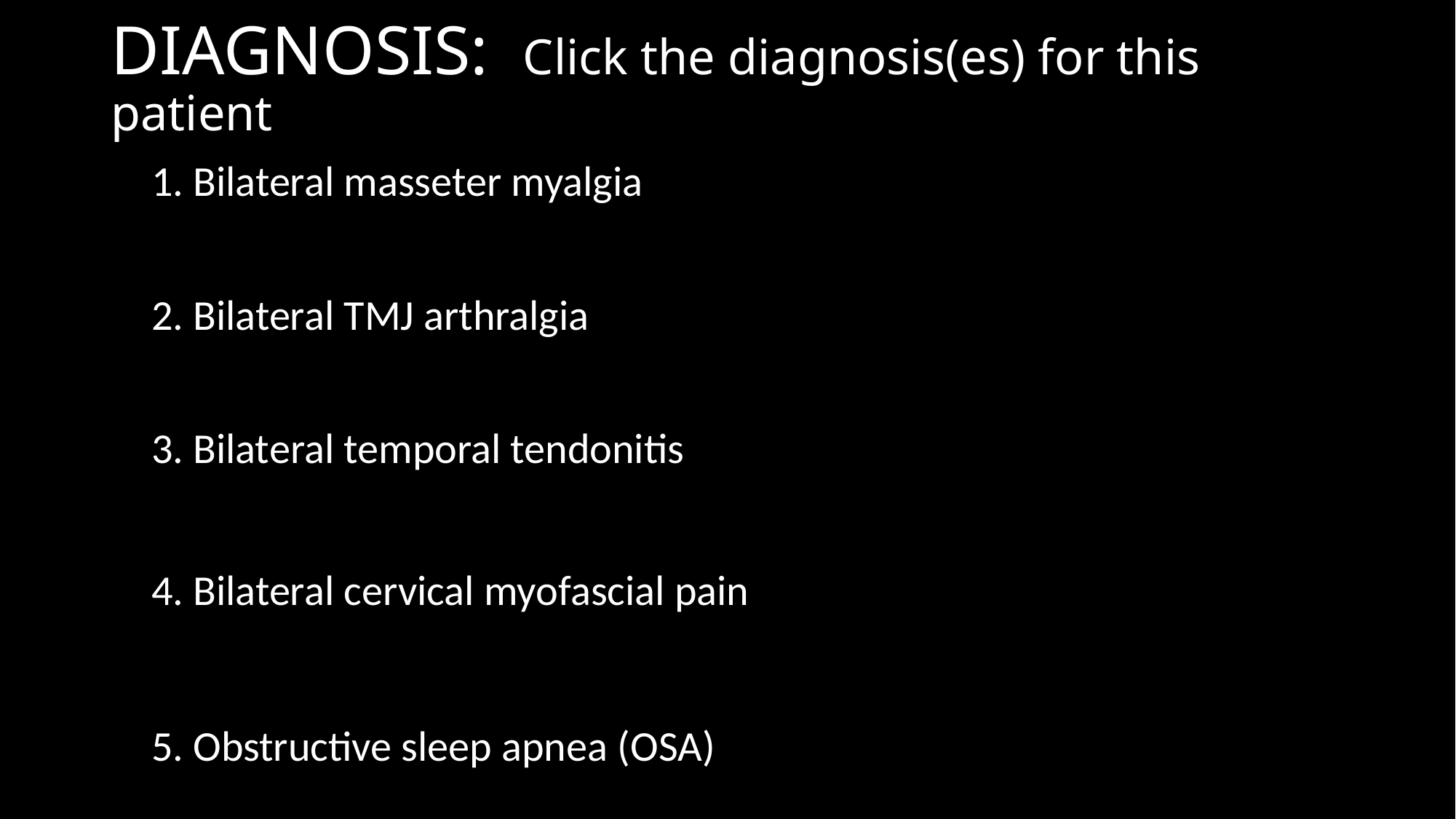

# DIAGNOSIS: Click the diagnosis(es) for this patient
1. Bilateral masseter myalgia
2. Bilateral TMJ arthralgia
3. Bilateral temporal tendonitis
4. Bilateral cervical myofascial pain
5. Obstructive sleep apnea (OSA)

## Slide 125
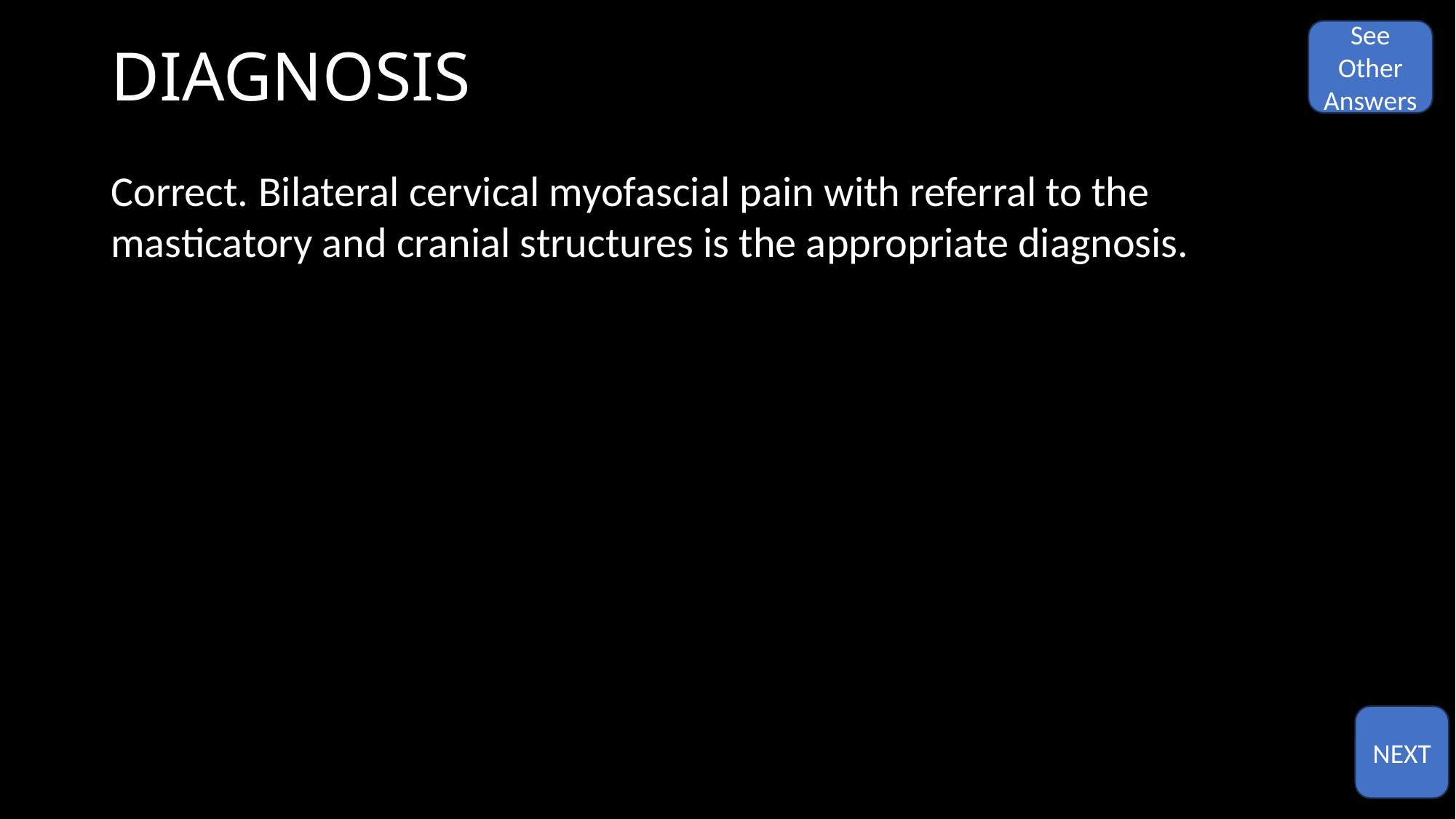

# DIAGNOSIS
See Other Answers
Correct. Bilateral cervical myofascial pain with referral to the masticatory and cranial structures is the appropriate diagnosis.
NEXT

## Slide 126
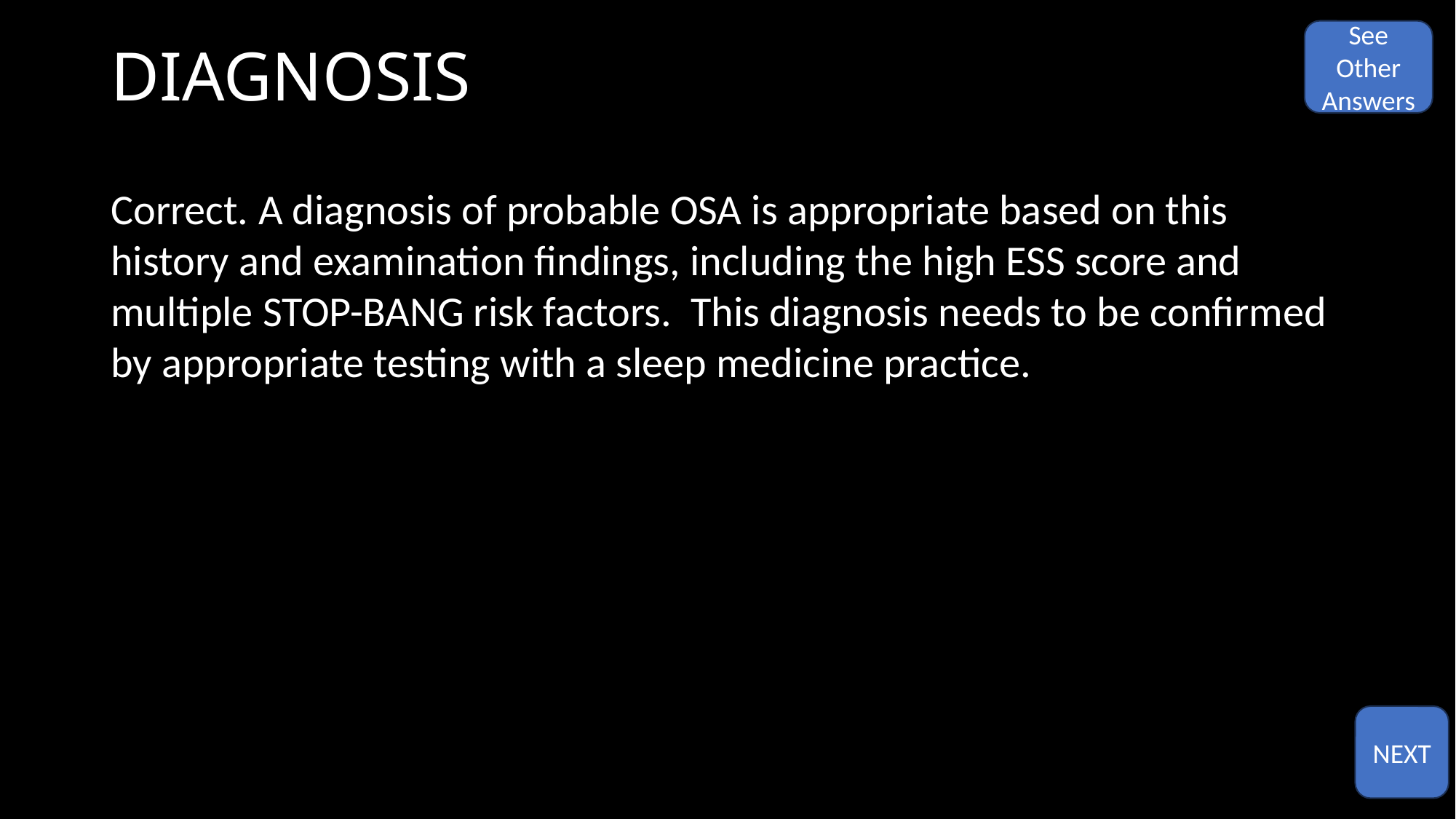

# DIAGNOSIS
See Other Answers
Correct. A diagnosis of probable OSA is appropriate based on this history and examination findings, including the high ESS score and multiple STOP-BANG risk factors. This diagnosis needs to be confirmed by appropriate testing with a sleep medicine practice.
NEXT

## Slide 127
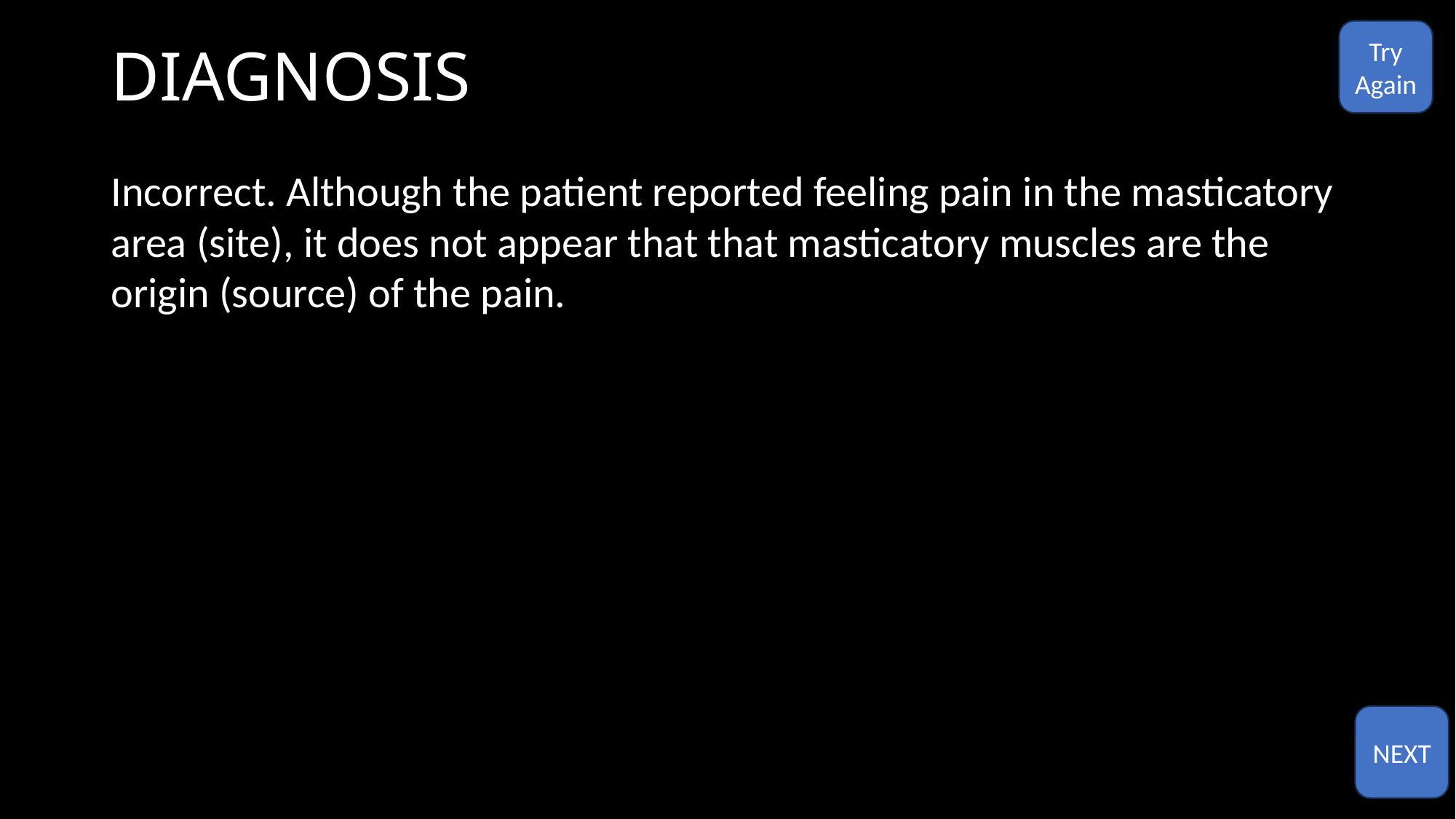

# DIAGNOSIS
Try
Again
Incorrect. Although the patient reported feeling pain in the masticatory area (site), it does not appear that that masticatory muscles are the origin (source) of the pain.
NEXT

## Slide 128
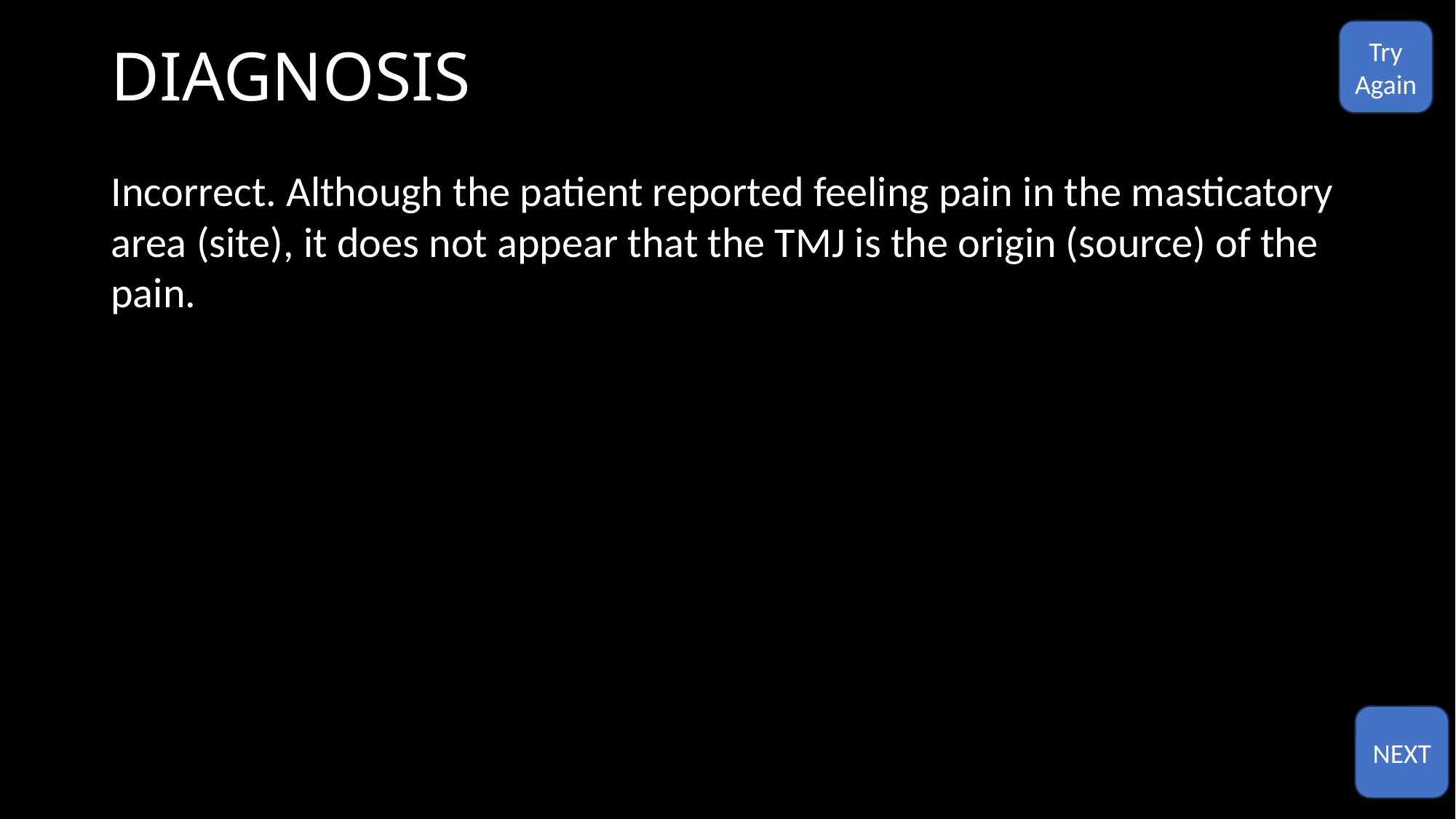

# DIAGNOSIS
Try
Again
Incorrect. Although the patient reported feeling pain in the masticatory area (site), it does not appear that the TMJ is the origin (source) of the pain.
NEXT

## Slide 129
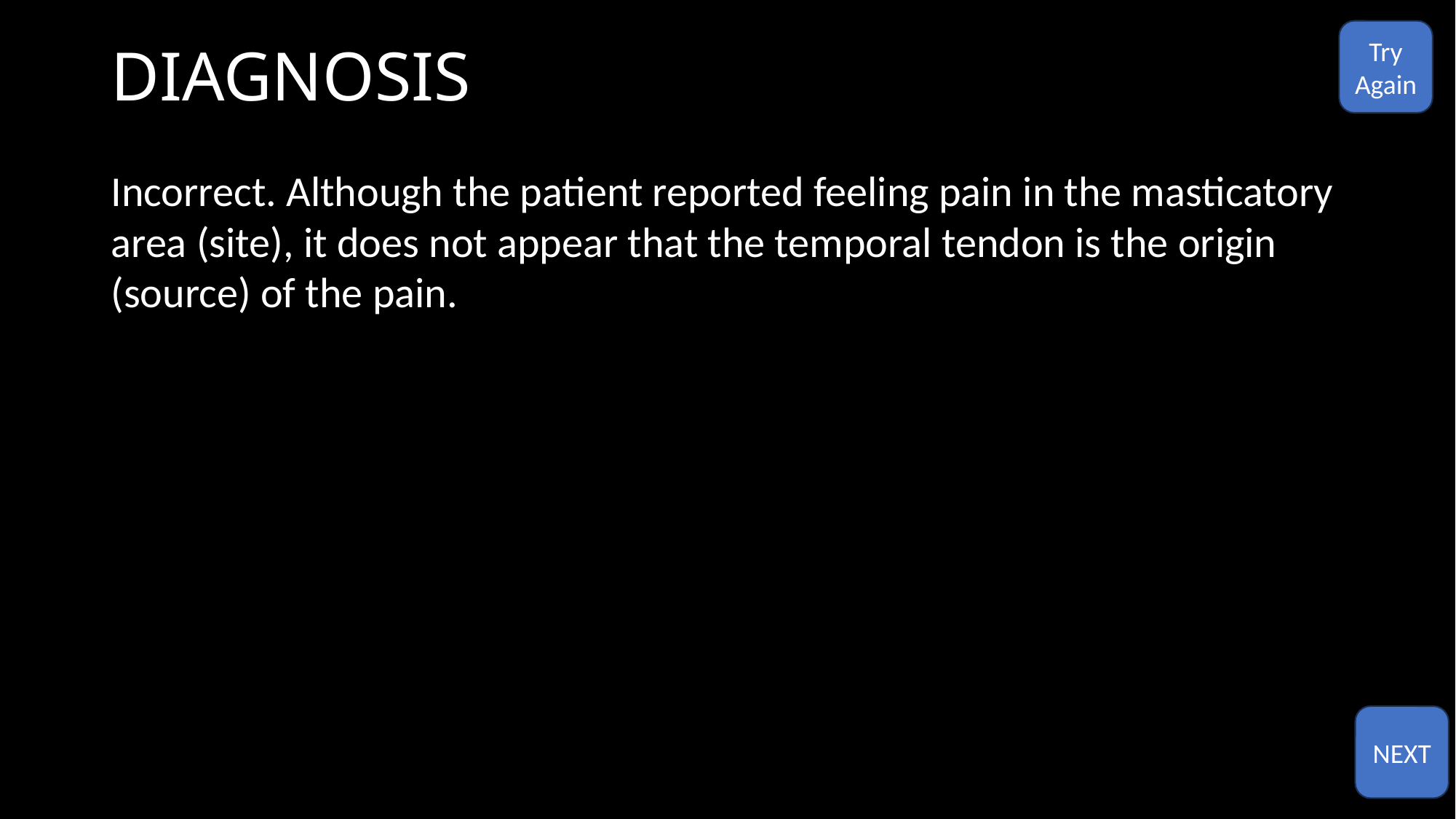

# DIAGNOSIS
Try
Again
Incorrect. Although the patient reported feeling pain in the masticatory area (site), it does not appear that the temporal tendon is the origin (source) of the pain.
NEXT

## Slide 130
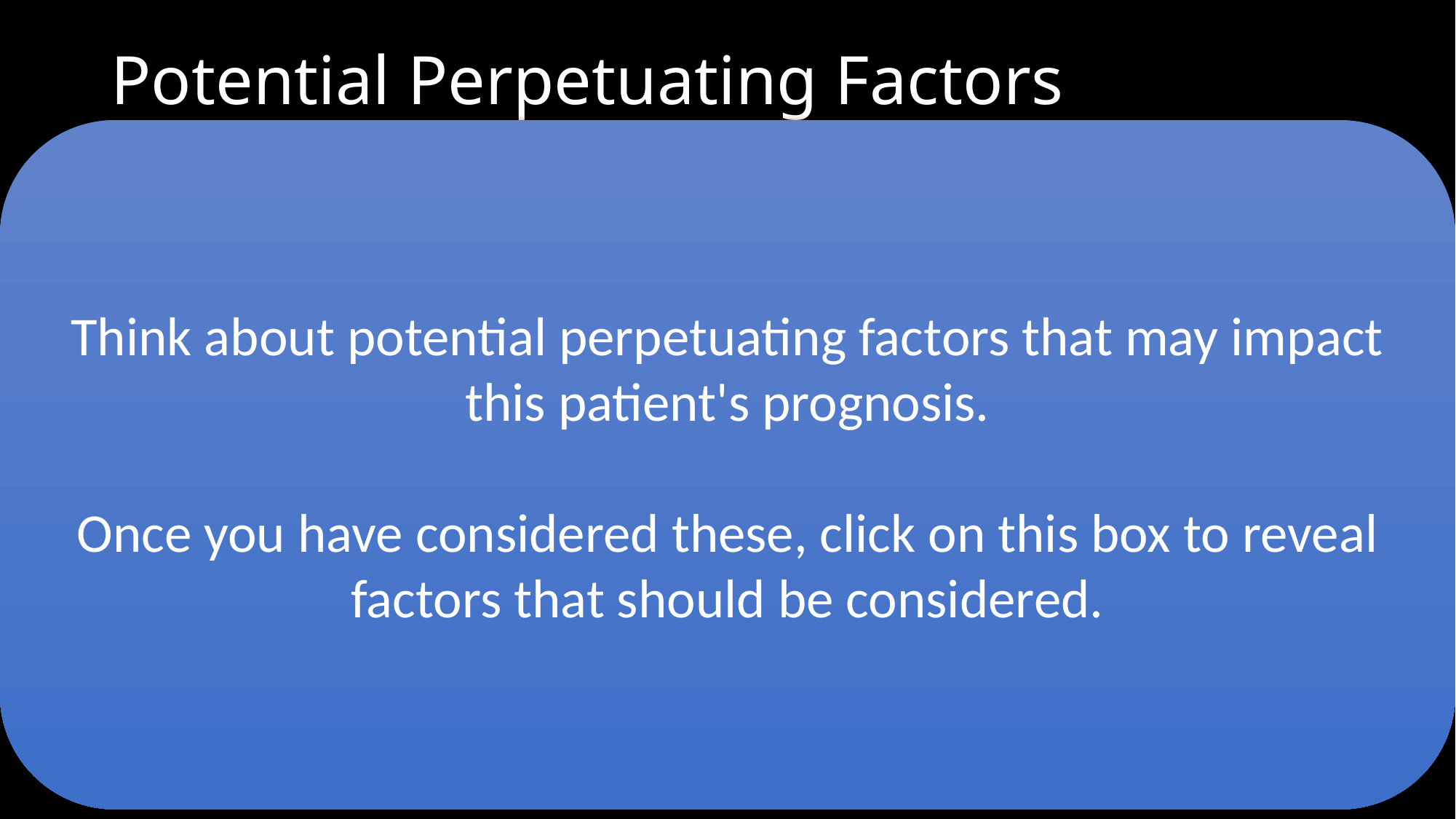

# Potential Perpetuating Factors
Cervical Parafunction
Cervical posture during work (cell phone, multiple computer monitors)
Sleep on his stomach with his head in a turned position
Probable OSA
May contribute to facial pain and headache
Medication Overuse
Near daily use of OTC Ibuprofen, which may contribute to a medication overuse headache
Nutrition
High coffee (caffeine) consumption
Think about potential perpetuating factors that may impact this patient's prognosis.
Once you have considered these, click on this box to reveal factors that should be considered.

## Slide 131
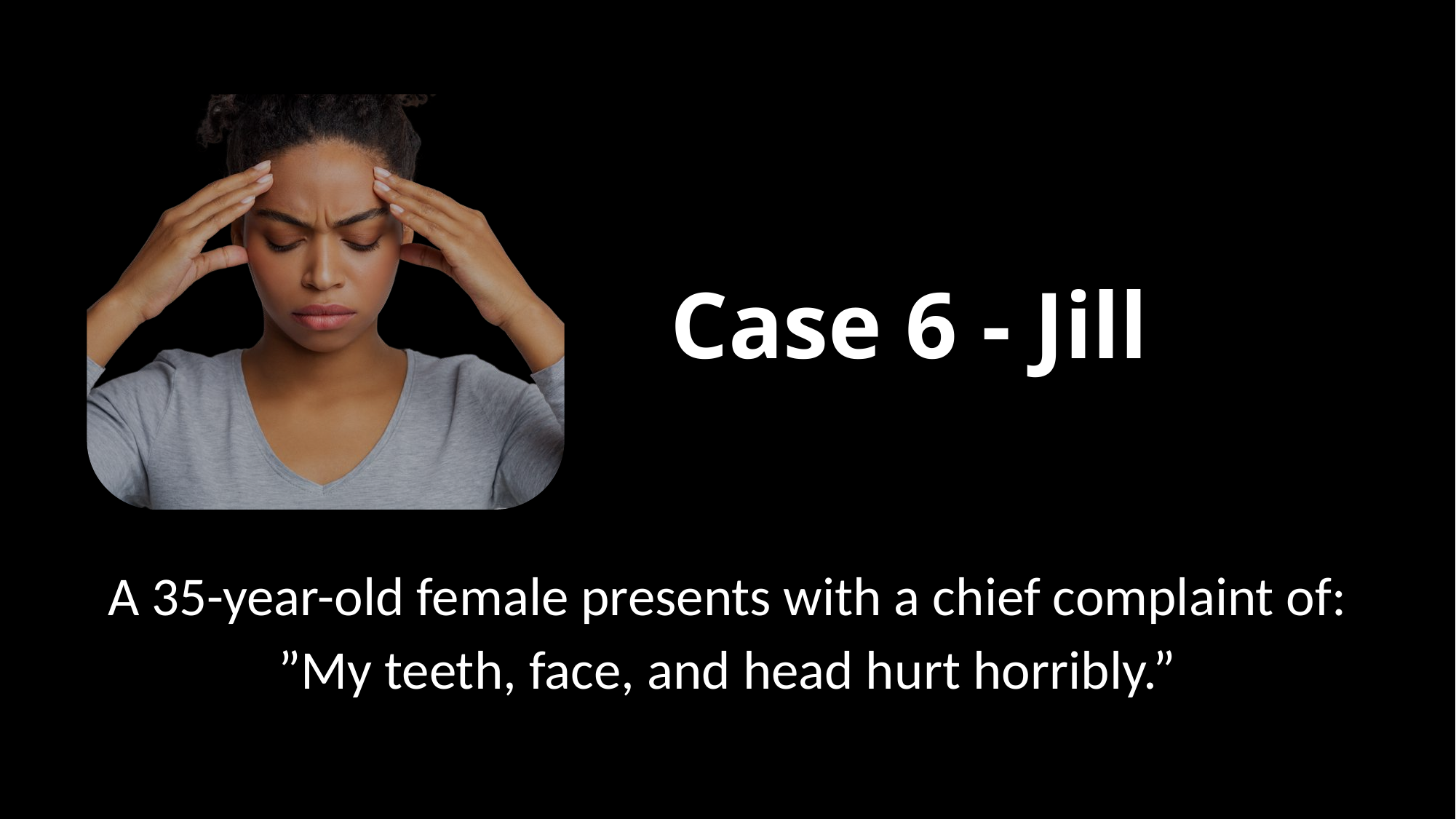

# Case 6 - Jill
A 35-year-old female presents with a chief complaint of:
”My teeth, face, and head hurt horribly.”

## Slide 132
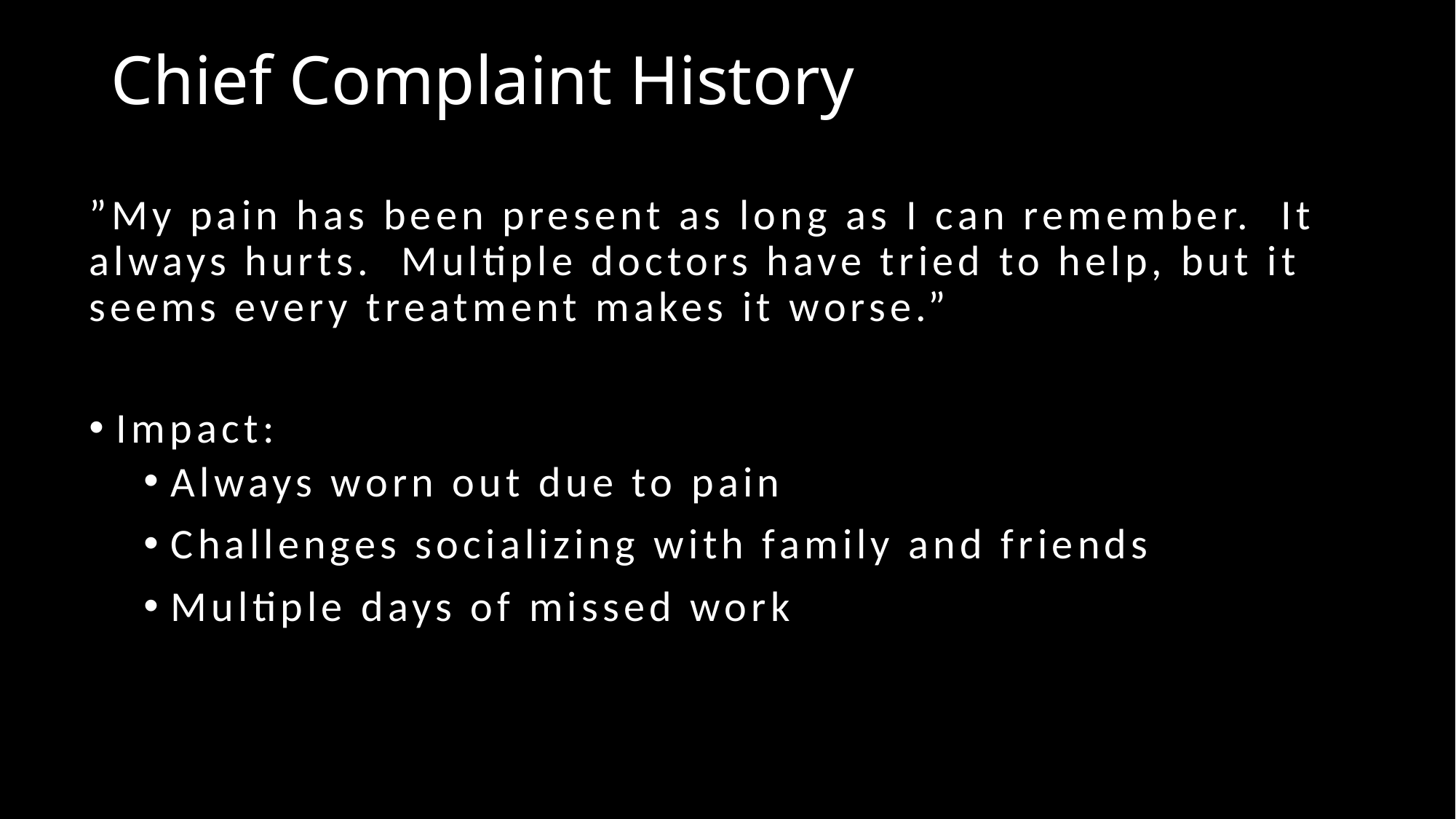

# Chief Complaint History
”My pain has been present as long as I can remember. It always hurts. Multiple doctors have tried to help, but it seems every treatment makes it worse.”
Impact:
Always worn out due to pain
Challenges socializing with family and friends
Multiple days of missed work

## Slide 133
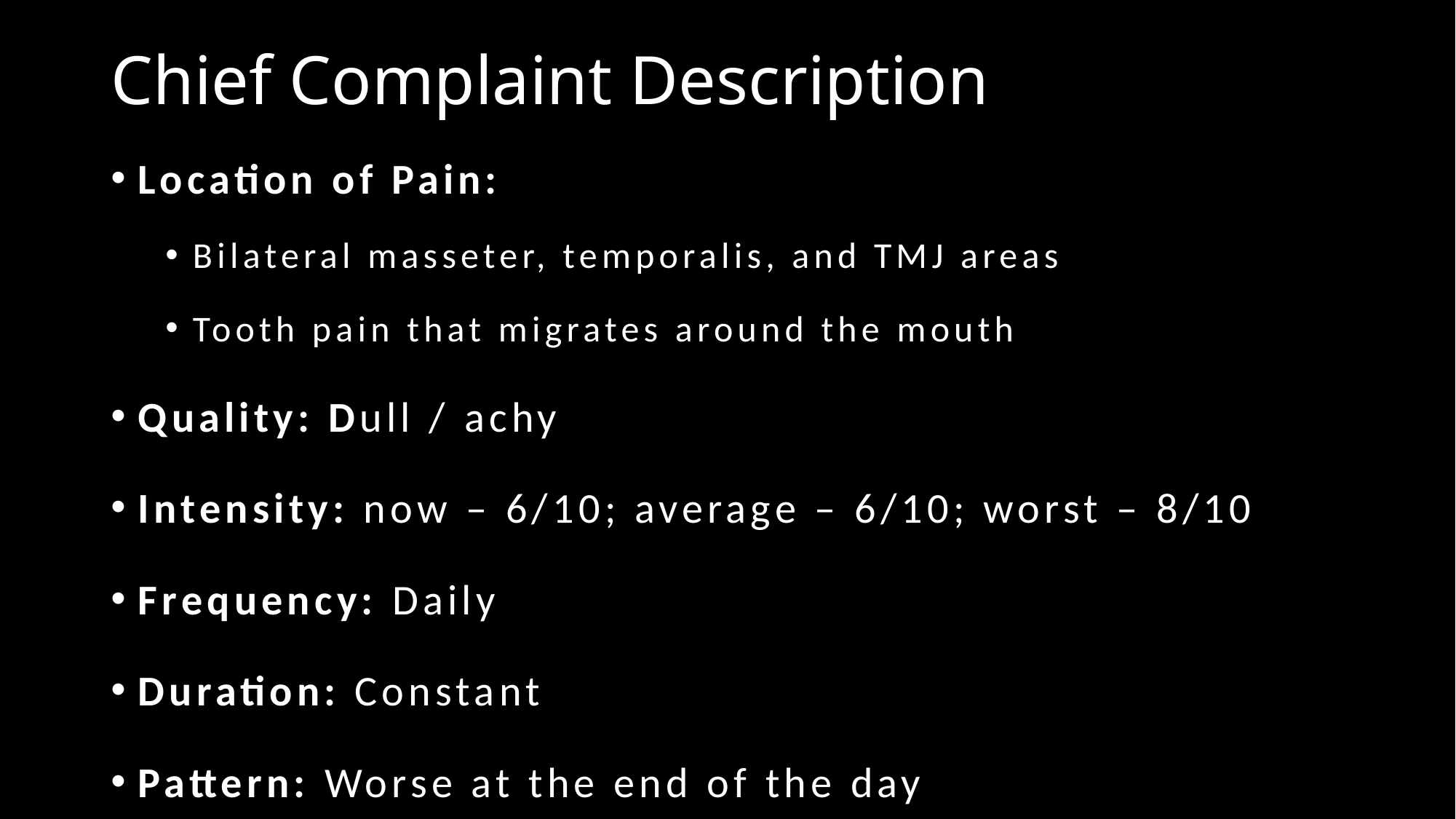

# Chief Complaint Description
Location of Pain:
Bilateral masseter, temporalis, and TMJ areas
Tooth pain that migrates around the mouth
Quality: Dull / achy
Intensity: now – 6/10; average – 6/10; worst – 8/10
Frequency: Daily
Duration: Constant
Pattern: Worse at the end of the day

## Slide 134
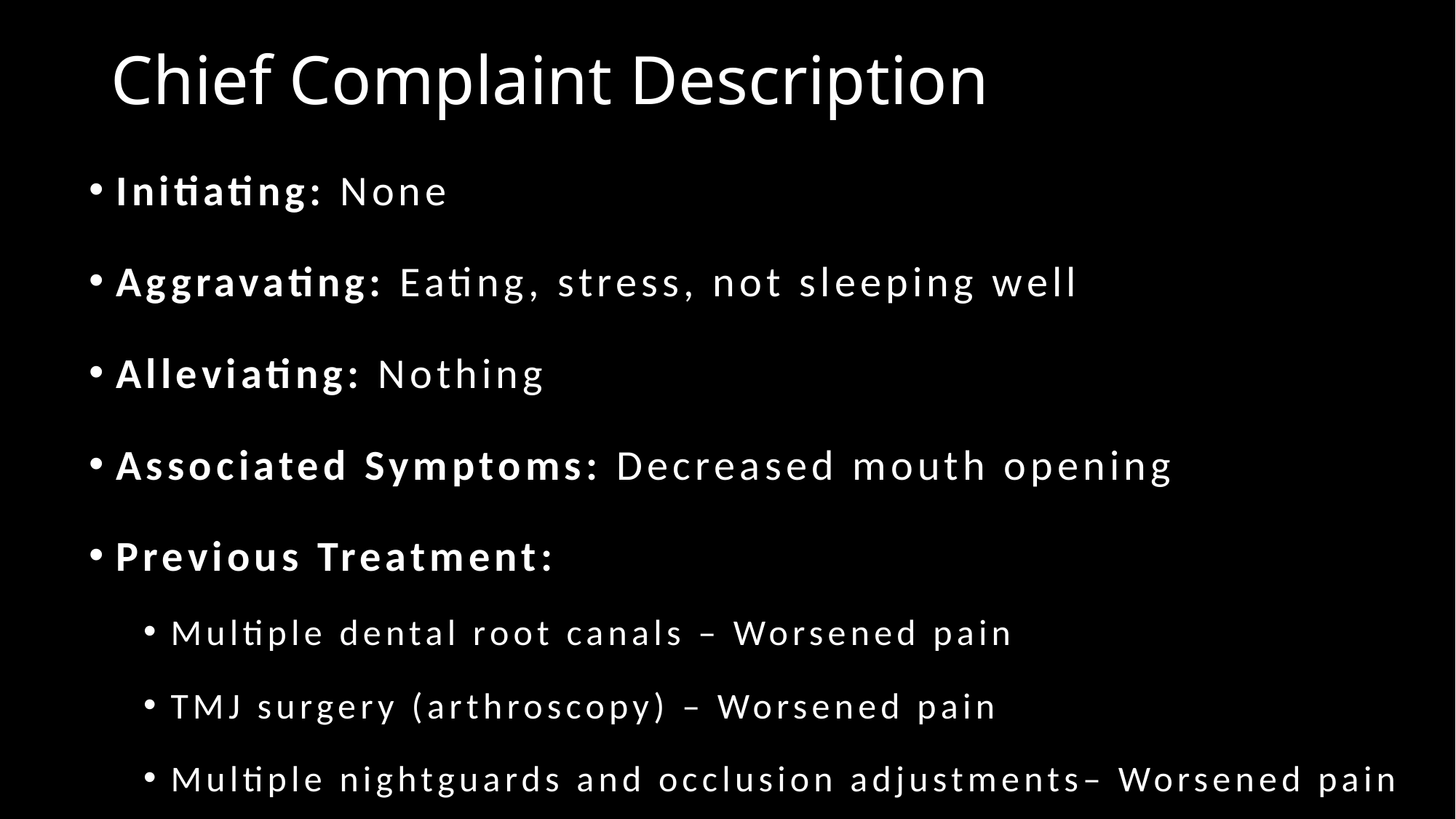

# Chief Complaint Description
Initiating: None
Aggravating: Eating, stress, not sleeping well
Alleviating: Nothing
Associated Symptoms: Decreased mouth opening
Previous Treatment:
Multiple dental root canals – Worsened pain
TMJ surgery (arthroscopy) – Worsened pain
Multiple nightguards and occlusion adjustments– Worsened pain

## Slide 135
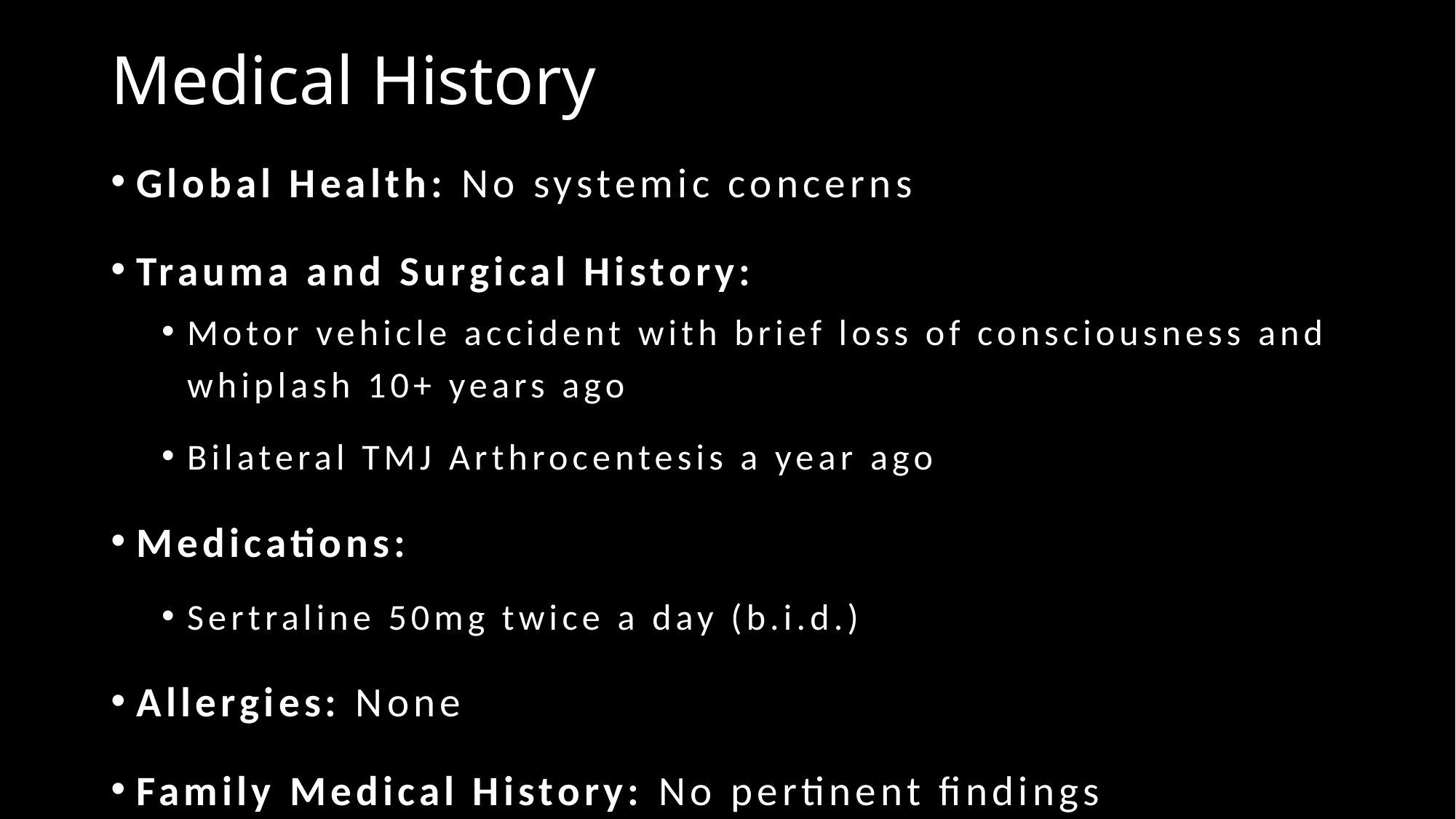

# Medical History
Global Health: No systemic concerns
Trauma and Surgical History:
Motor vehicle accident with brief loss of consciousness and whiplash 10+ years ago
Bilateral TMJ Arthrocentesis a year ago
Medications:
Sertraline 50mg twice a day (b.i.d.)
Allergies: None
Family Medical History: No pertinent findings

## Slide 136
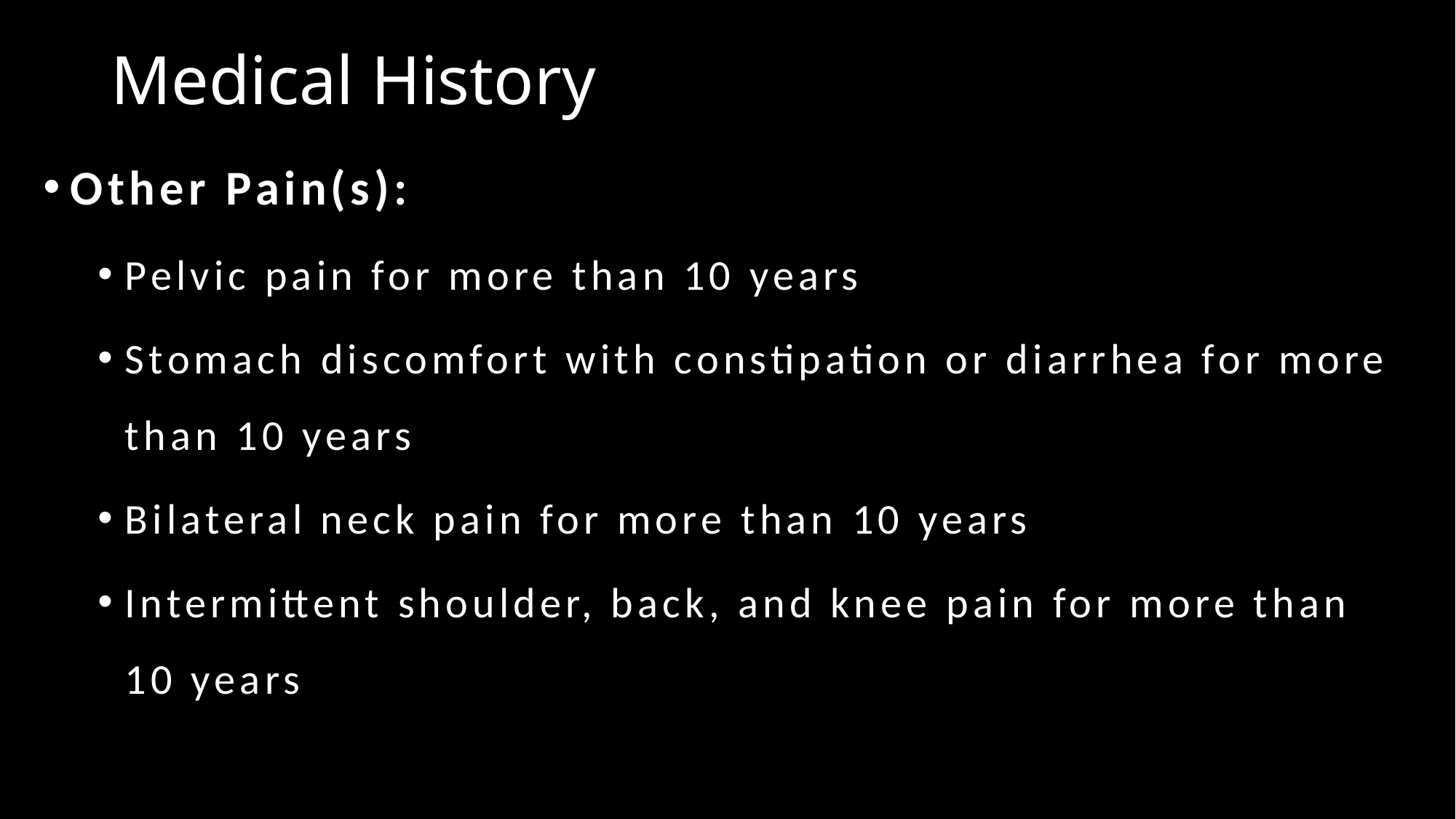

# Medical History
Other Pain(s):
Pelvic pain for more than 10 years
Stomach discomfort with constipation or diarrhea for more than 10 years
Bilateral neck pain for more than 10 years
Intermittent shoulder, back, and knee pain for more than 10 years

## Slide 137
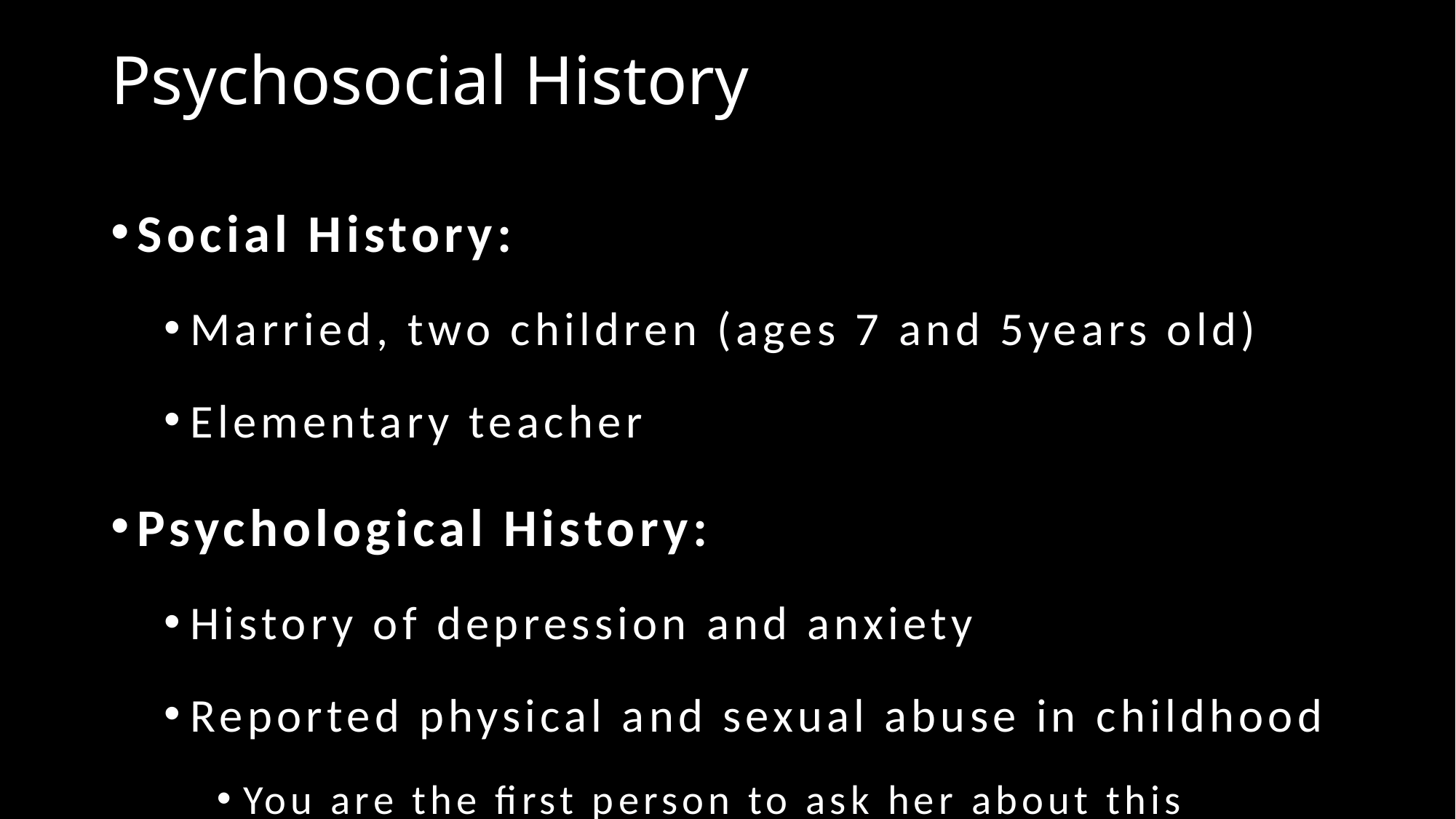

# Psychosocial History
Social History:
Married, two children (ages 7 and 5years old)
Elementary teacher
Psychological History:
History of depression and anxiety
Reported physical and sexual abuse in childhood
You are the first person to ask her about this

## Slide 138
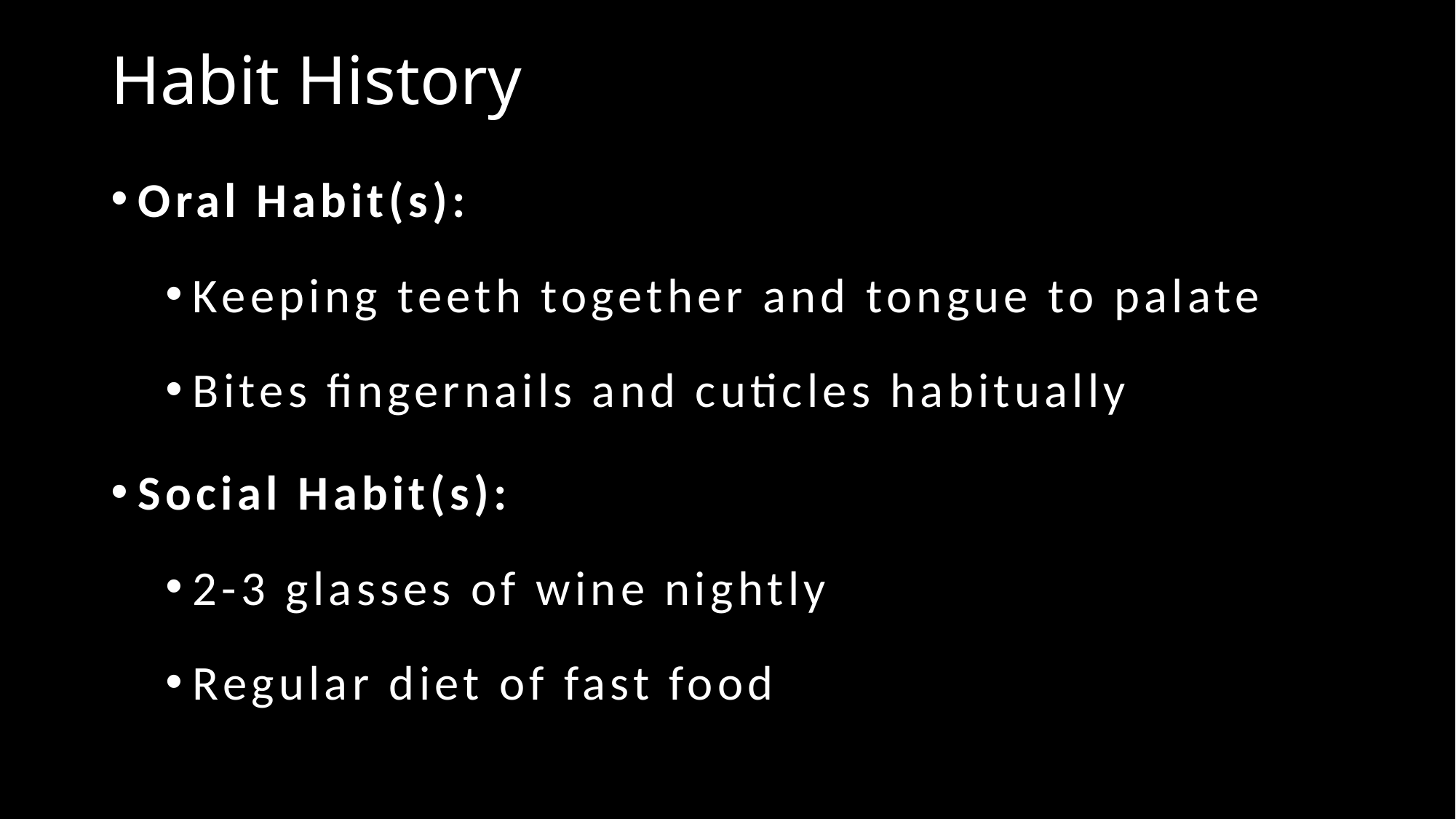

# Habit History
Oral Habit(s):
Keeping teeth together and tongue to palate
Bites fingernails and cuticles habitually
Social Habit(s):
2-3 glasses of wine nightly
Regular diet of fast food

## Slide 139
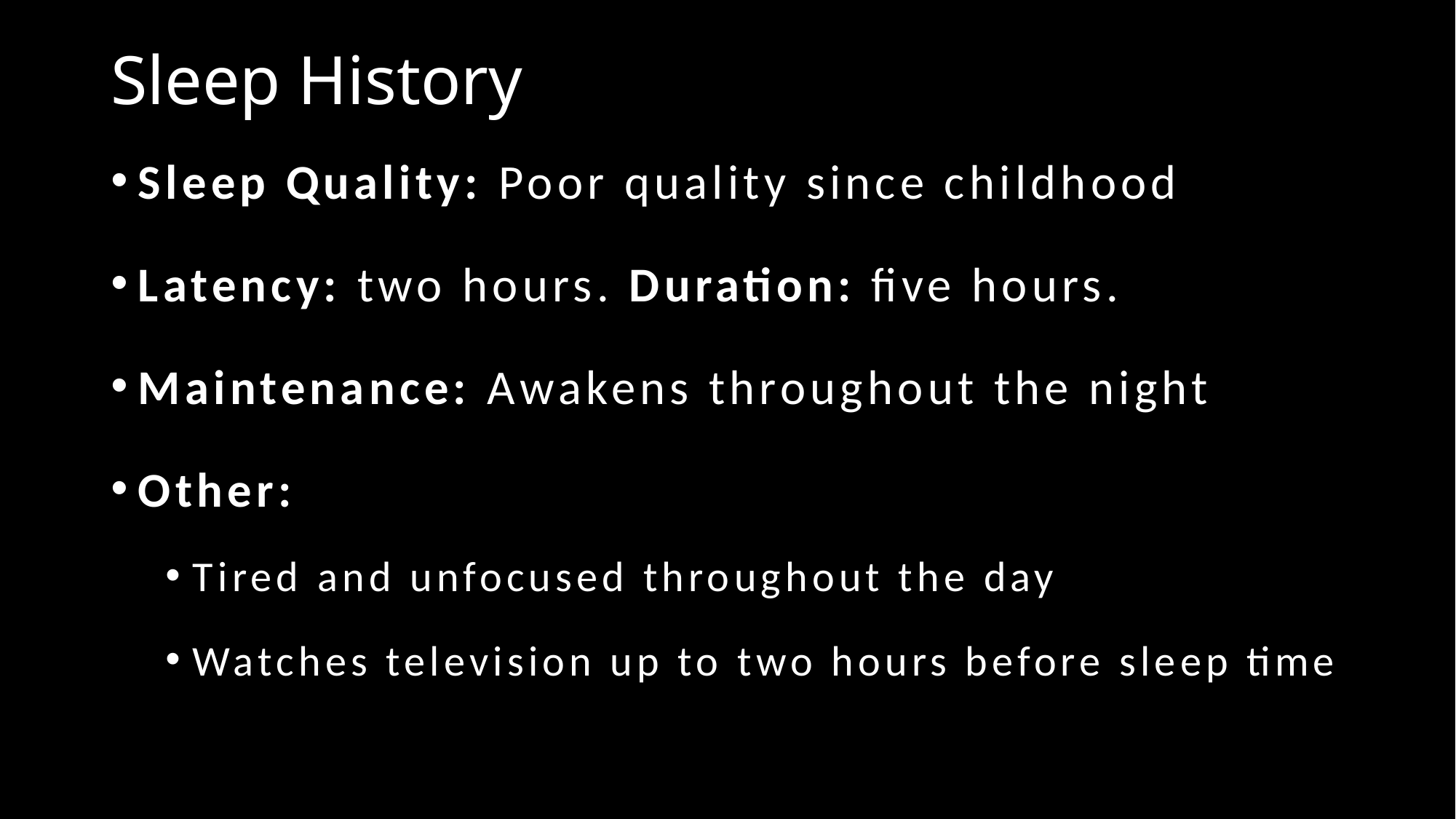

# Sleep History
Sleep Quality: Poor quality since childhood
Latency: two hours. Duration: five hours.
Maintenance: Awakens throughout the night
Other:
Tired and unfocused throughout the day
Watches television up to two hours before sleep time

## Slide 140
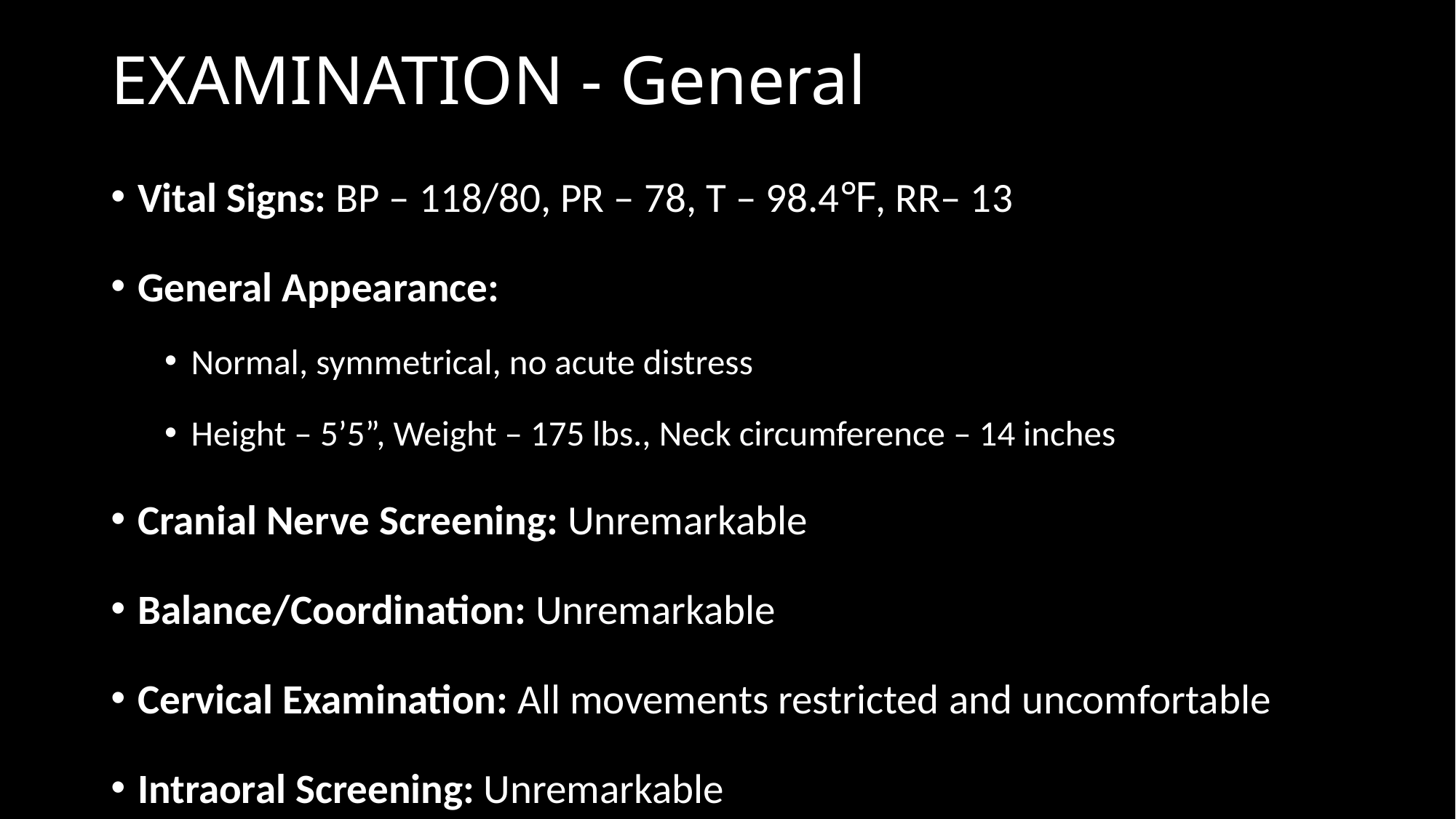

# EXAMINATION - General
Vital Signs: BP – 118/80, PR – 78, T – 98.4℉, RR– 13
General Appearance:
Normal, symmetrical, no acute distress
Height – 5’5”, Weight – 175 lbs., Neck circumference – 14 inches
Cranial Nerve Screening: Unremarkable
Balance/Coordination: Unremarkable
Cervical Examination: All movements restricted and uncomfortable
Intraoral Screening: Unremarkable

## Slide 141
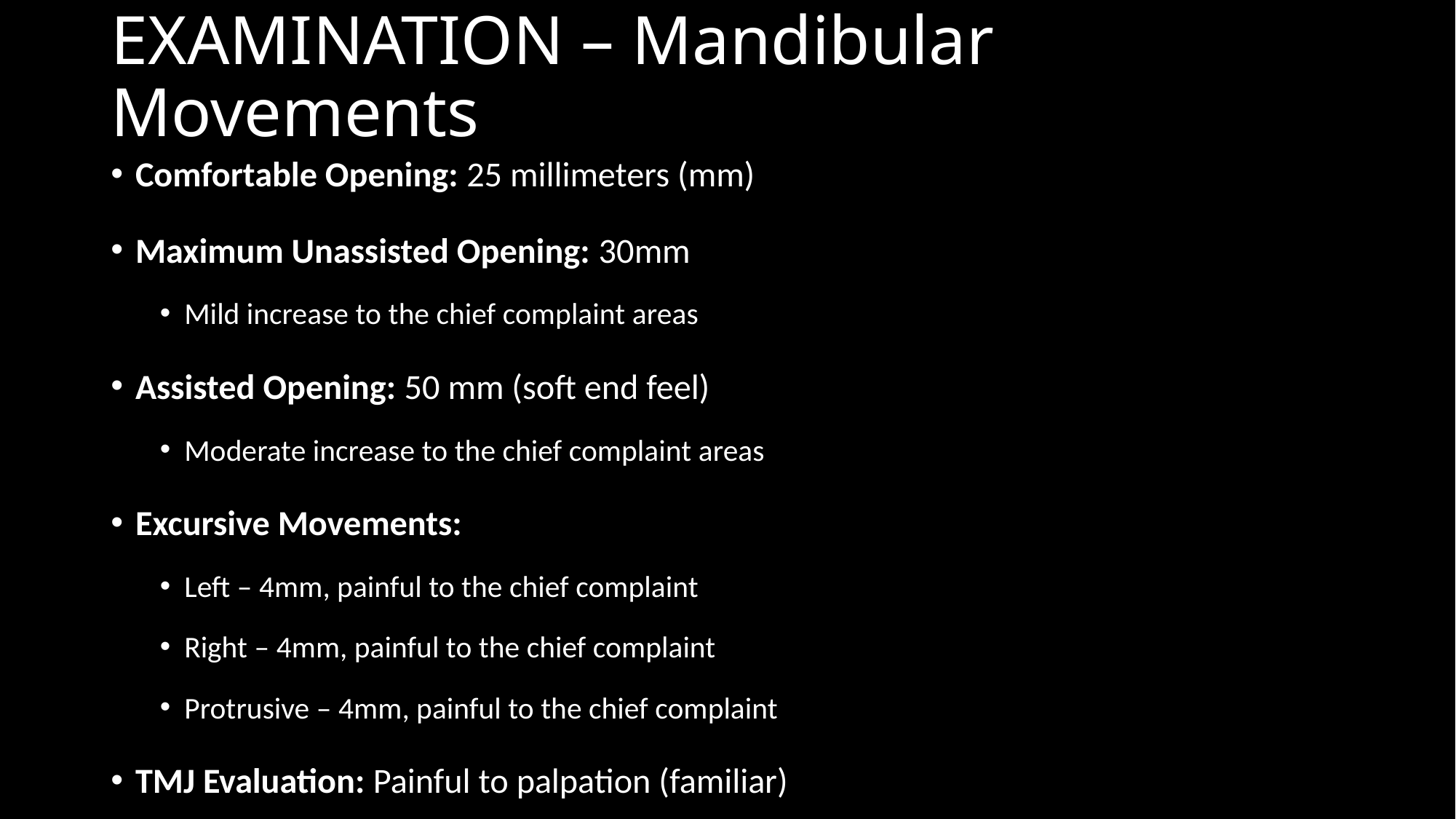

# EXAMINATION – Mandibular Movements
Comfortable Opening: 25 millimeters (mm)
Maximum Unassisted Opening: 30mm
Mild increase to the chief complaint areas
Assisted Opening: 50 mm (soft end feel)
Moderate increase to the chief complaint areas
Excursive Movements:
Left – 4mm, painful to the chief complaint
Right – 4mm, painful to the chief complaint
Protrusive – 4mm, painful to the chief complaint
TMJ Evaluation: Painful to palpation (familiar)

## Slide 142
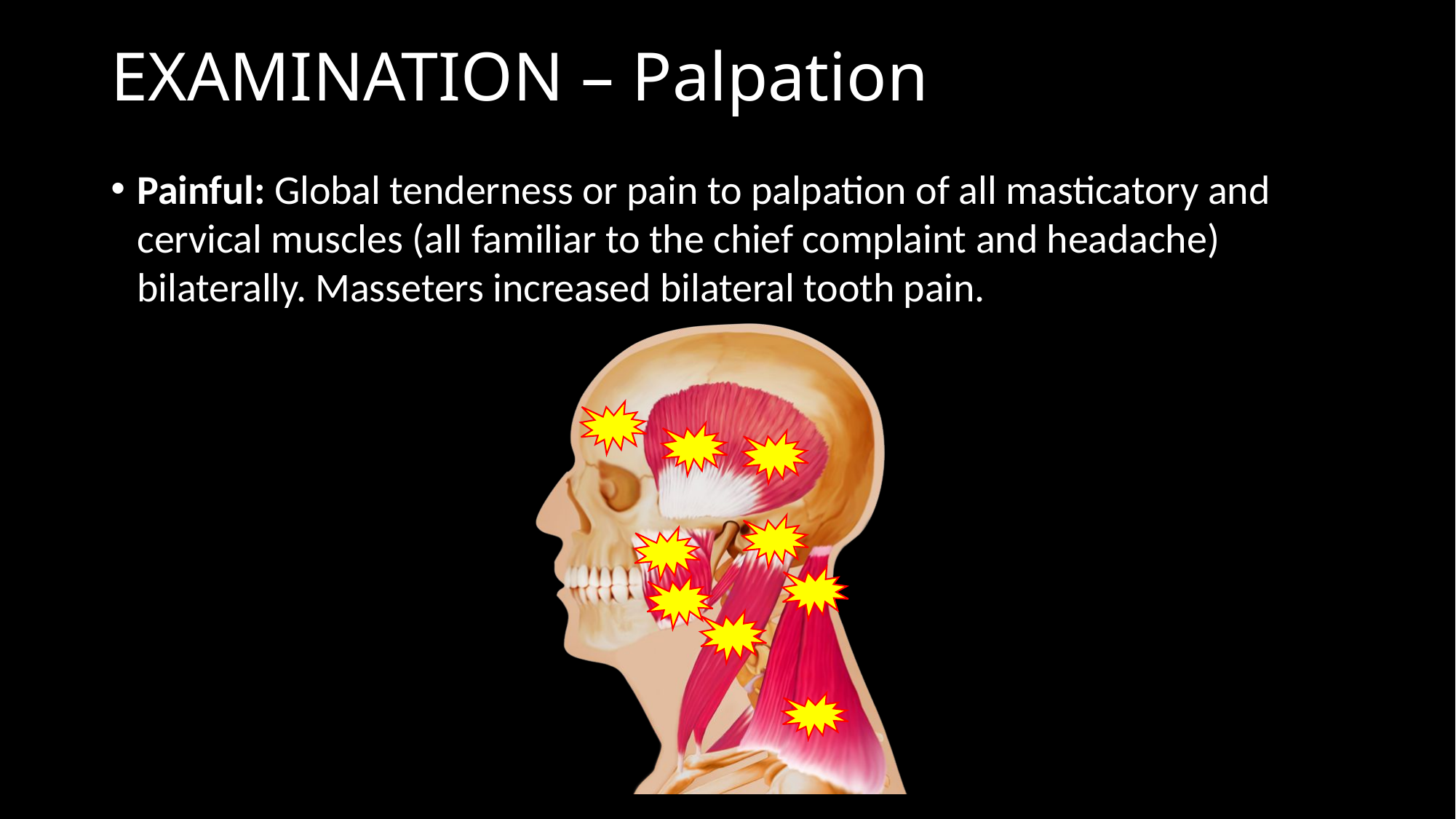

# EXAMINATION – Palpation
Painful: Global tenderness or pain to palpation of all masticatory and cervical muscles (all familiar to the chief complaint and headache) bilaterally. Masseters increased bilateral tooth pain.

## Slide 143
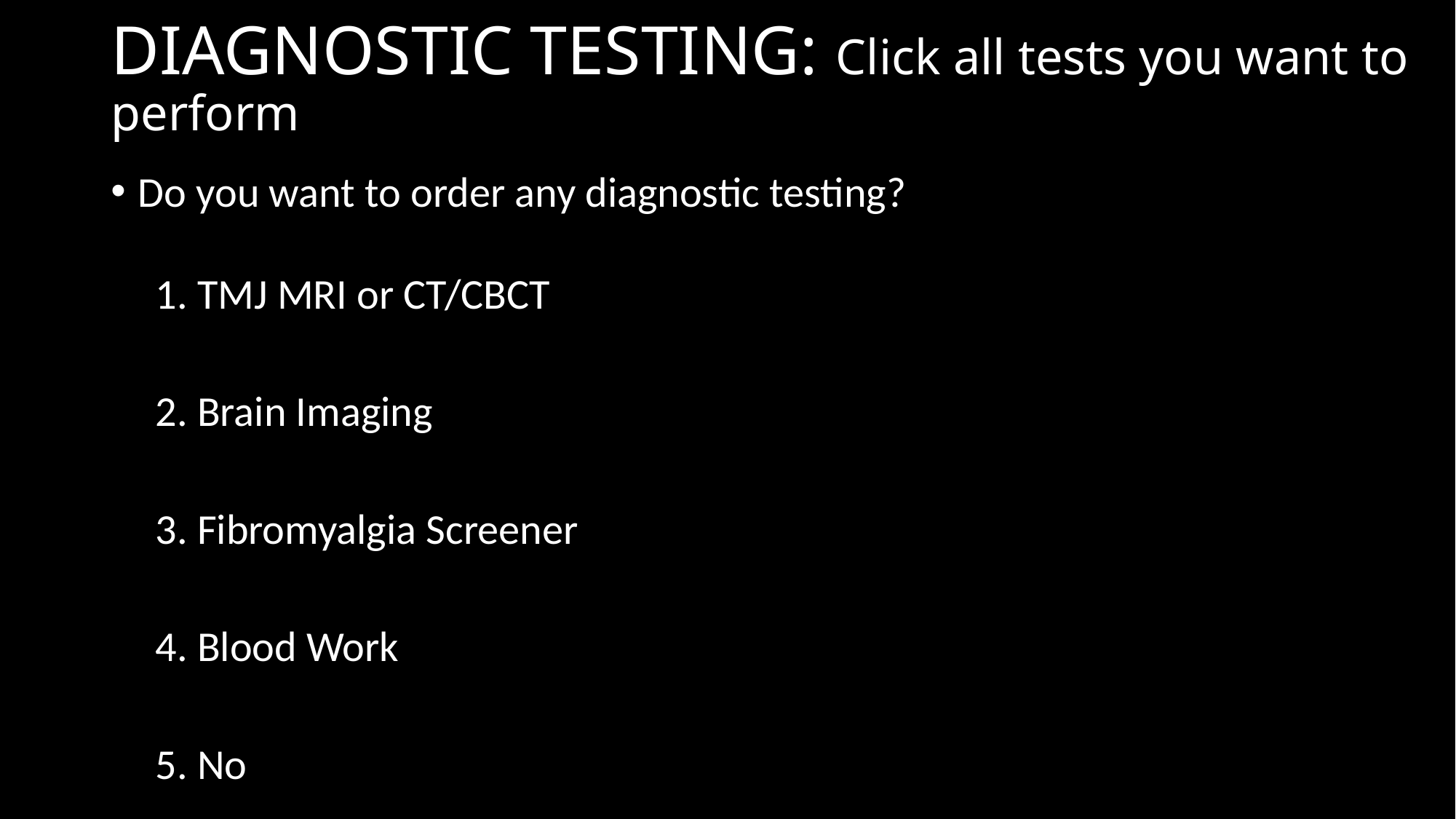

DIAGNOSTIC TESTING: Click all tests you want to perform
Do you want to order any diagnostic testing?
1. TMJ MRI or CT/CBCT
2. Brain Imaging
3. Fibromyalgia Screener
4. Blood Work
5. No

## Slide 144
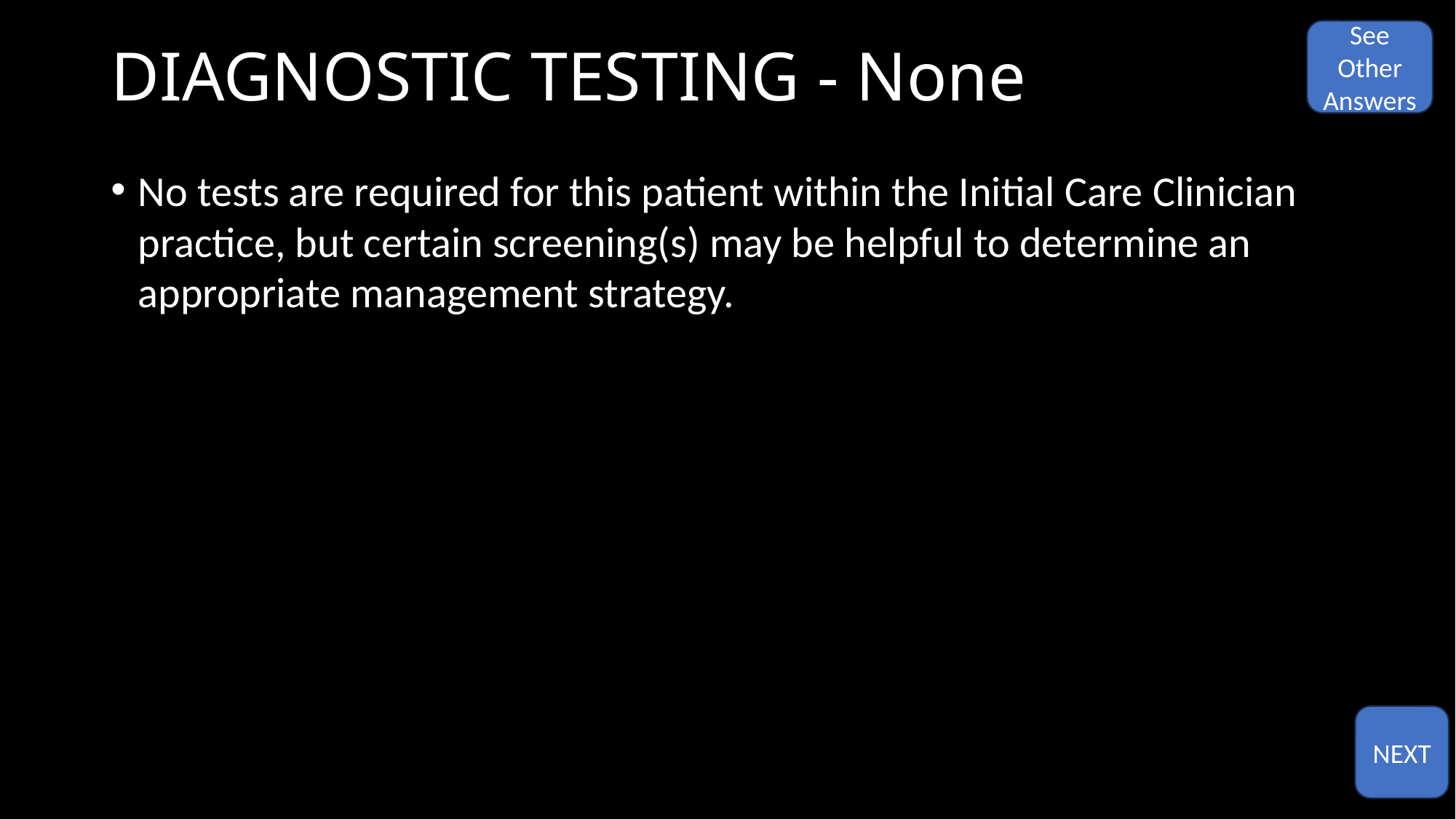

# DIAGNOSTIC TESTING - None
See Other Answers
No tests are required for this patient within the Initial Care Clinician practice, but certain screening(s) may be helpful to determine an appropriate management strategy.
NEXT

## Slide 145
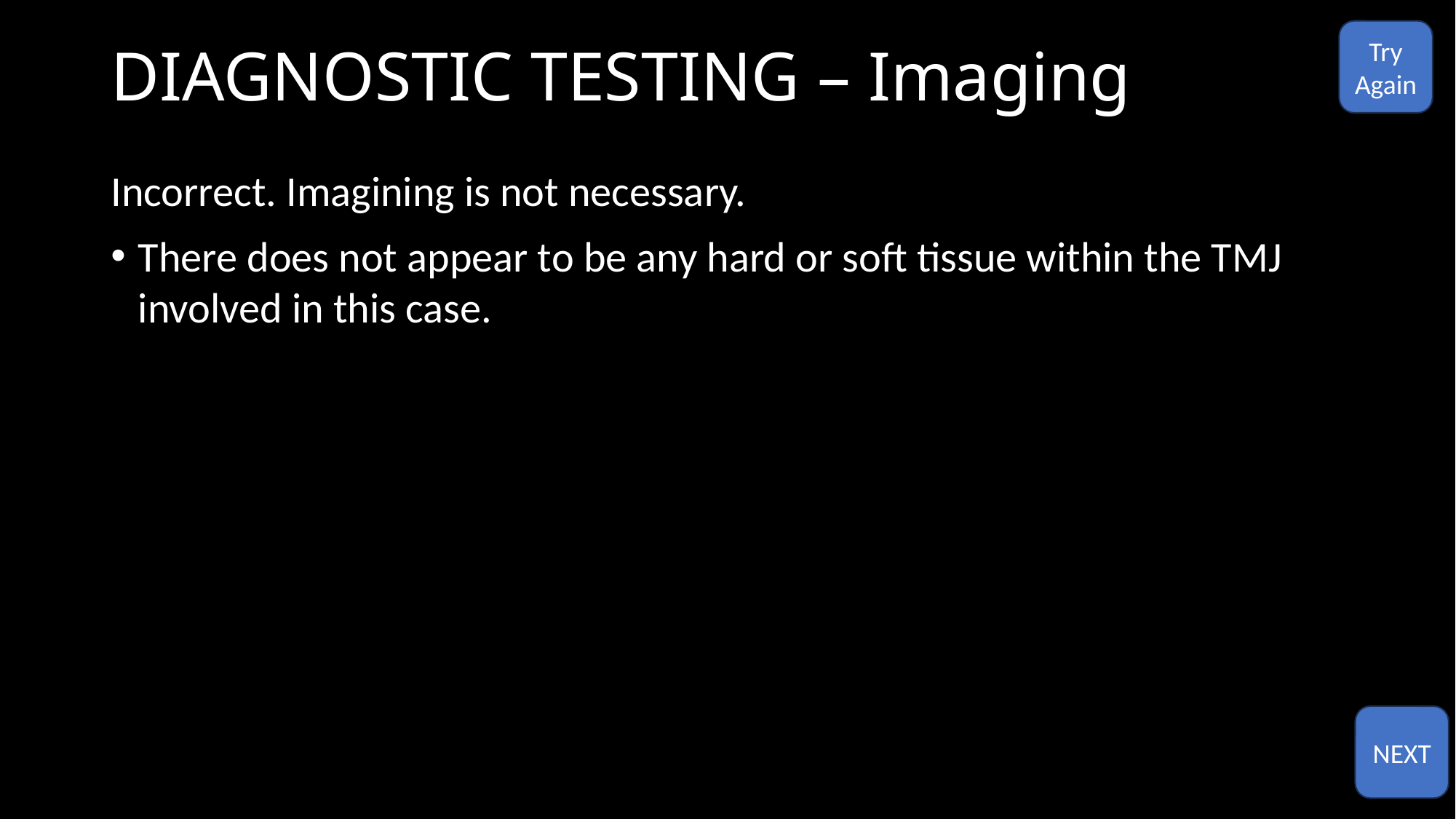

# DIAGNOSTIC TESTING – Imaging
Try
Again
Incorrect. Imagining is not necessary.
There does not appear to be any hard or soft tissue within the TMJ involved in this case.
NEXT

## Slide 146
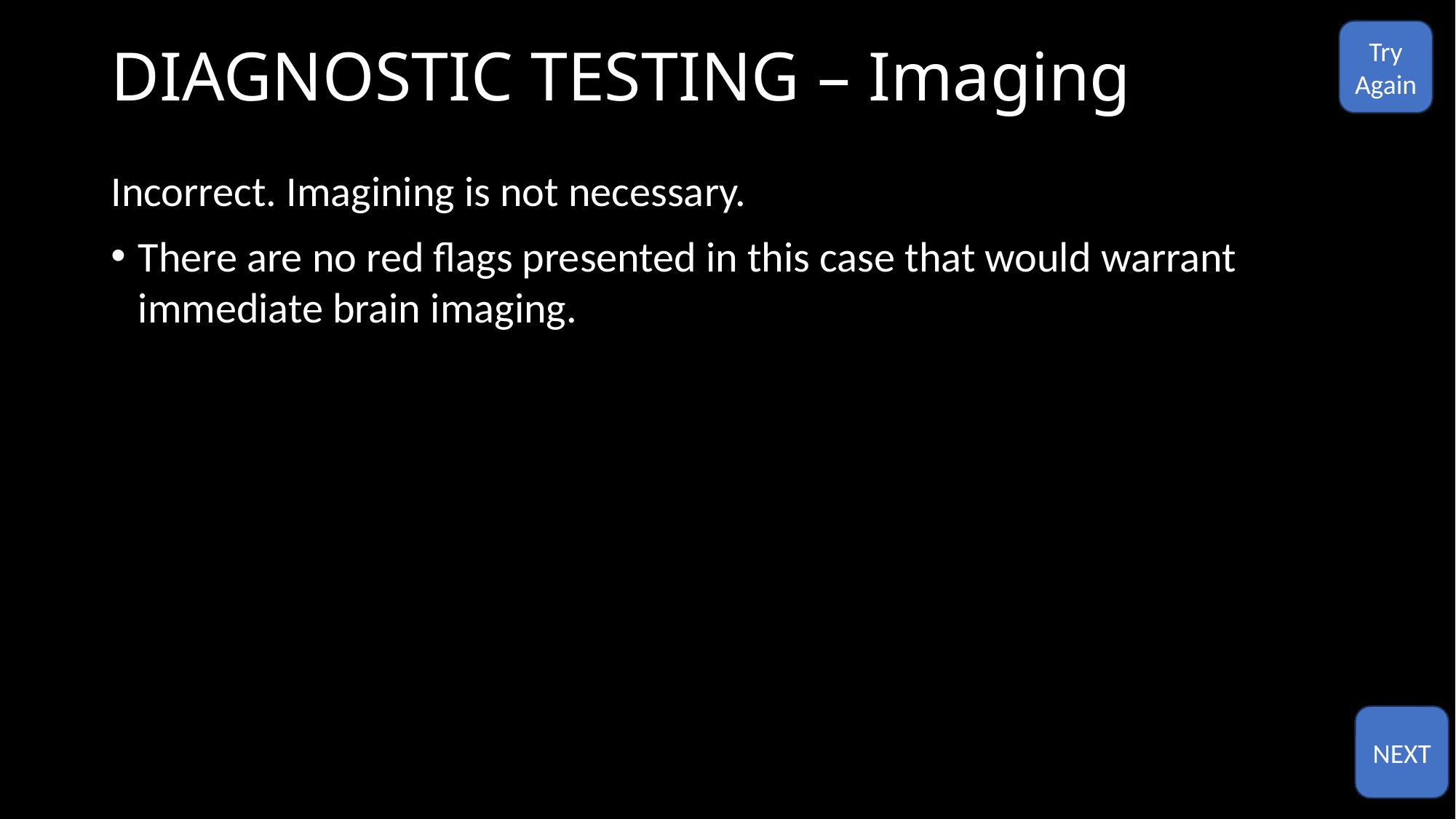

# DIAGNOSTIC TESTING – Imaging
Try
Again
Incorrect. Imagining is not necessary.
There are no red flags presented in this case that would warrant immediate brain imaging.
NEXT

## Slide 147
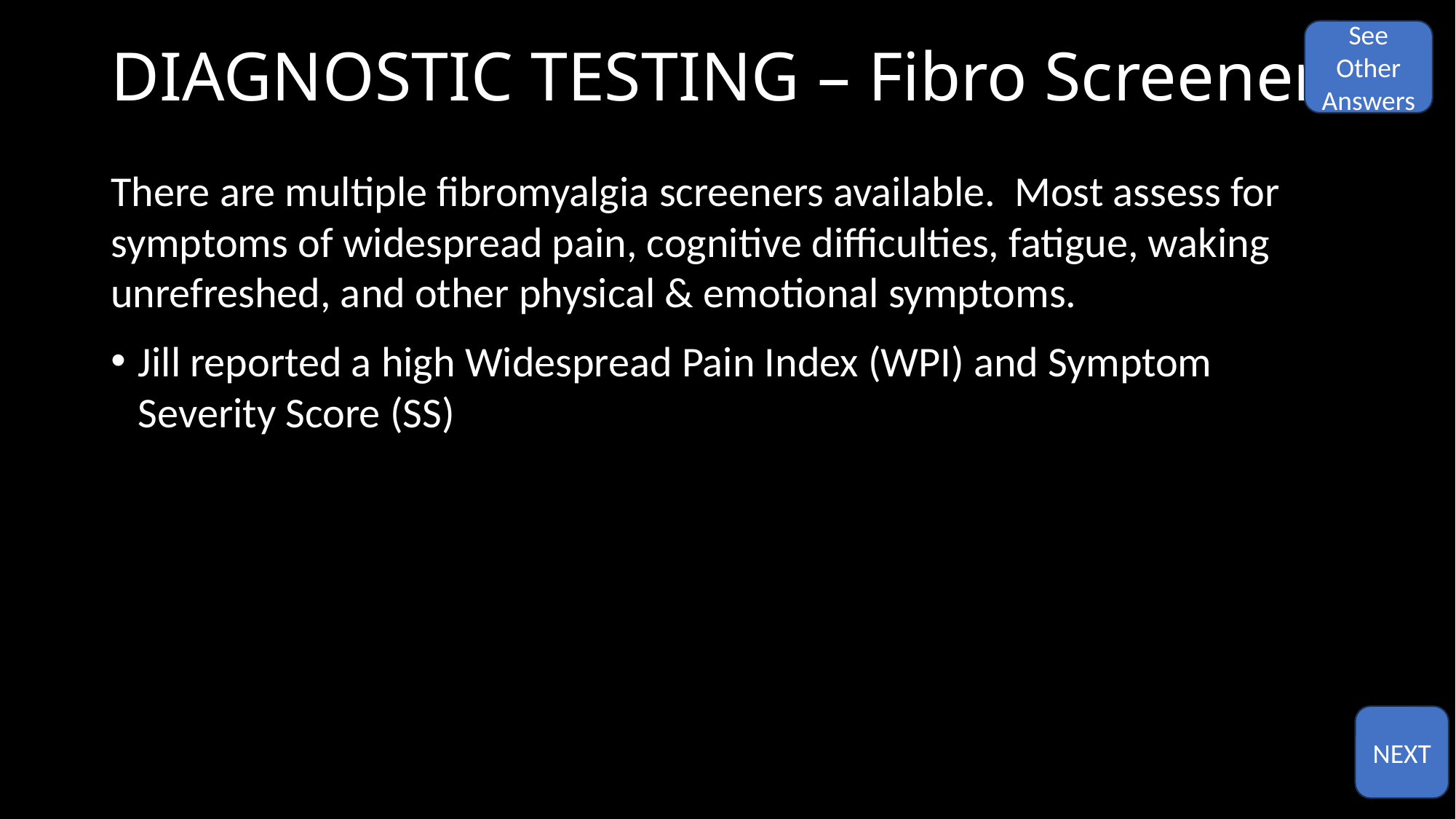

# DIAGNOSTIC TESTING – Fibro Screener
See Other Answers
There are multiple fibromyalgia screeners available. Most assess for symptoms of widespread pain, cognitive difficulties, fatigue, waking unrefreshed, and other physical & emotional symptoms.
Jill reported a high Widespread Pain Index (WPI) and Symptom Severity Score (SS)
NEXT

## Slide 148
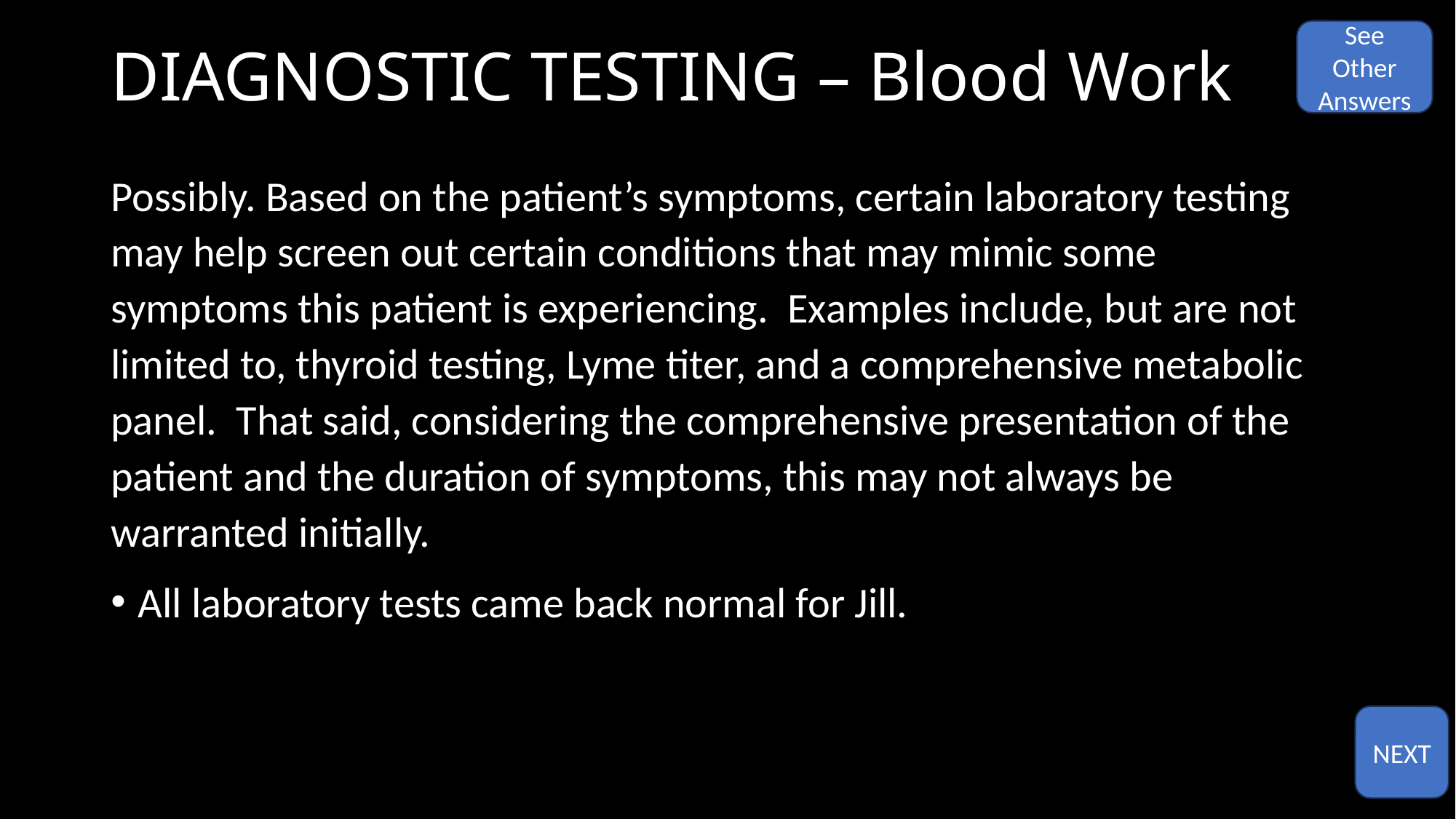

# DIAGNOSTIC TESTING – Blood Work
See Other Answers
Possibly. Based on the patient’s symptoms, certain laboratory testing may help screen out certain conditions that may mimic some symptoms this patient is experiencing. Examples include, but are not limited to, thyroid testing, Lyme titer, and a comprehensive metabolic panel. That said, considering the comprehensive presentation of the patient and the duration of symptoms, this may not always be warranted initially.
All laboratory tests came back normal for Jill.
NEXT

## Slide 149
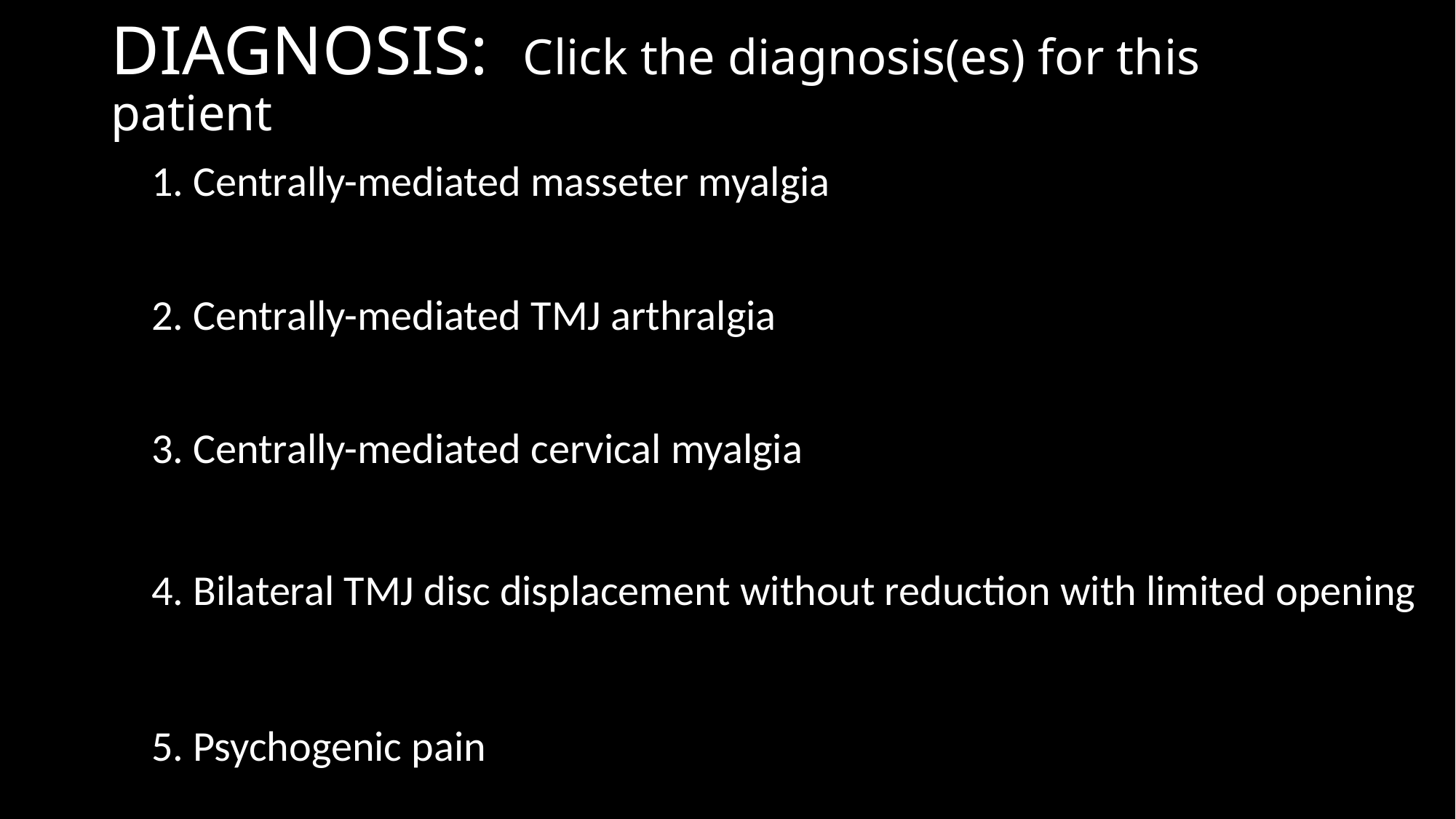

# DIAGNOSIS: Click the diagnosis(es) for this patient
1. Centrally-mediated masseter myalgia
2. Centrally-mediated TMJ arthralgia
3. Centrally-mediated cervical myalgia
4. Bilateral TMJ disc displacement without reduction with limited opening
5. Psychogenic pain

## Slide 150
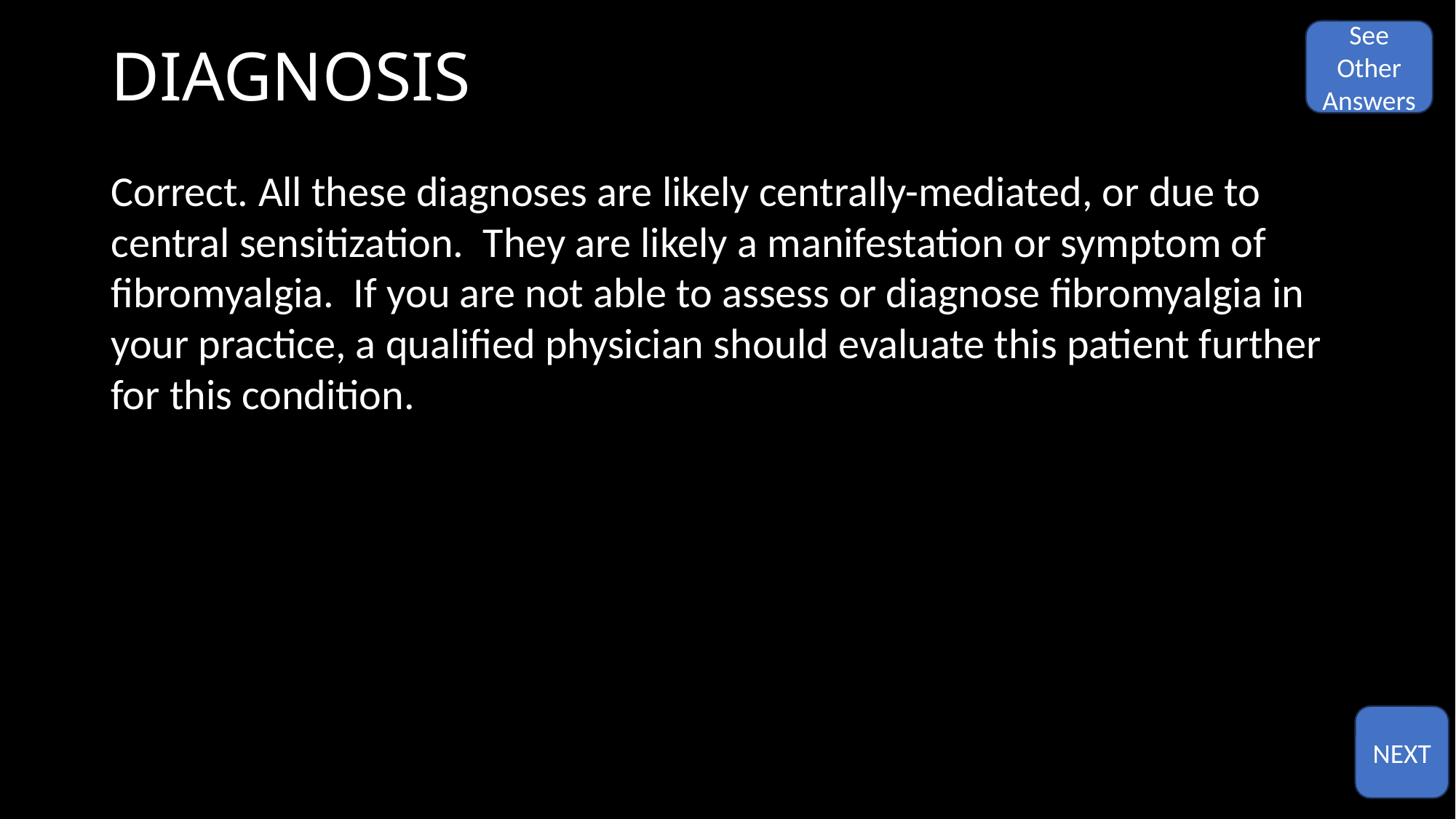

# DIAGNOSIS
See Other Answers
Correct. All these diagnoses are likely centrally-mediated, or due to central sensitization. They are likely a manifestation or symptom of fibromyalgia. If you are not able to assess or diagnose fibromyalgia in your practice, a qualified physician should evaluate this patient further for this condition.
NEXT

## Slide 151
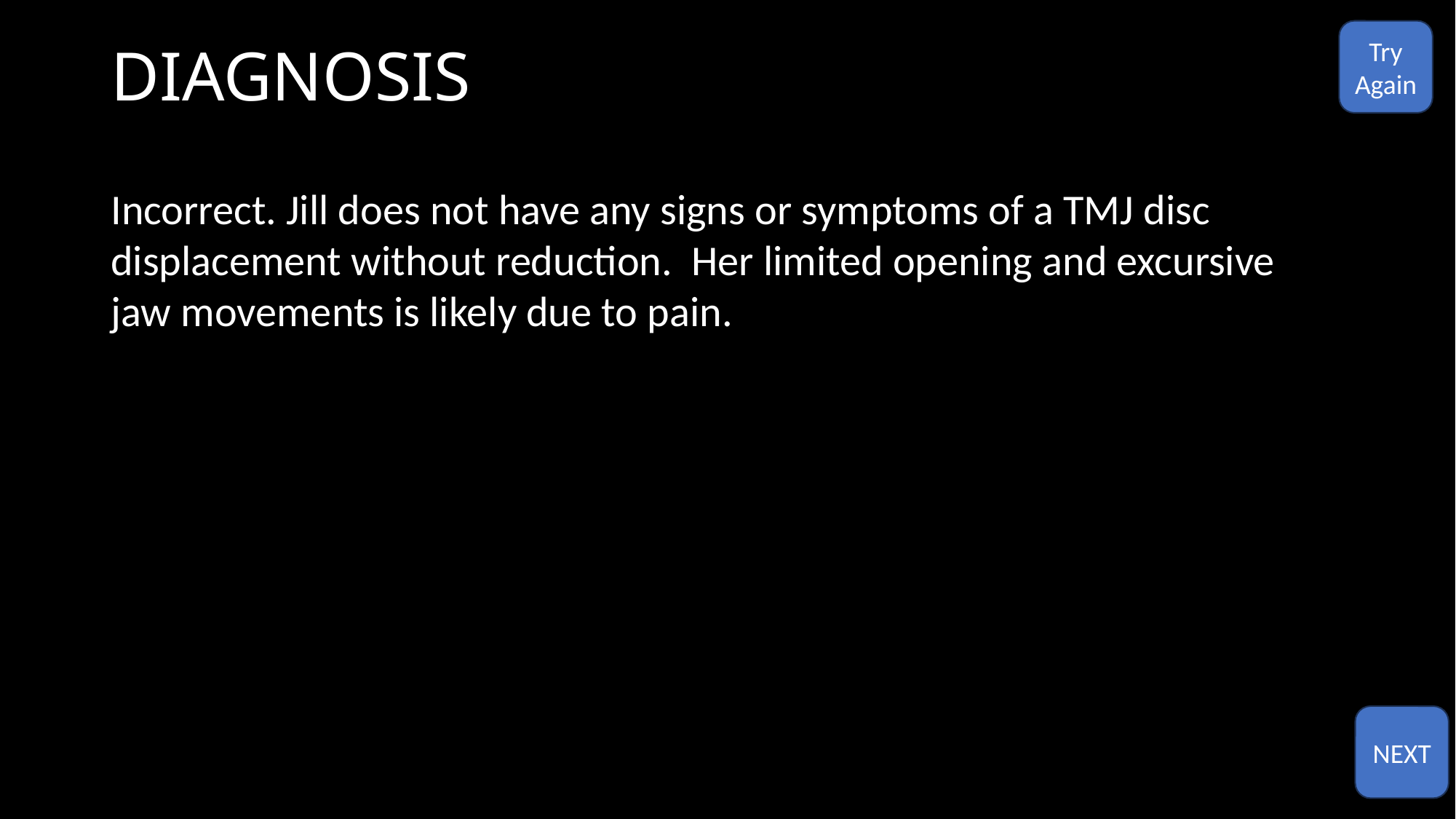

# DIAGNOSIS
Try
Again
Incorrect. Jill does not have any signs or symptoms of a TMJ disc displacement without reduction. Her limited opening and excursive jaw movements is likely due to pain.
NEXT

## Slide 152
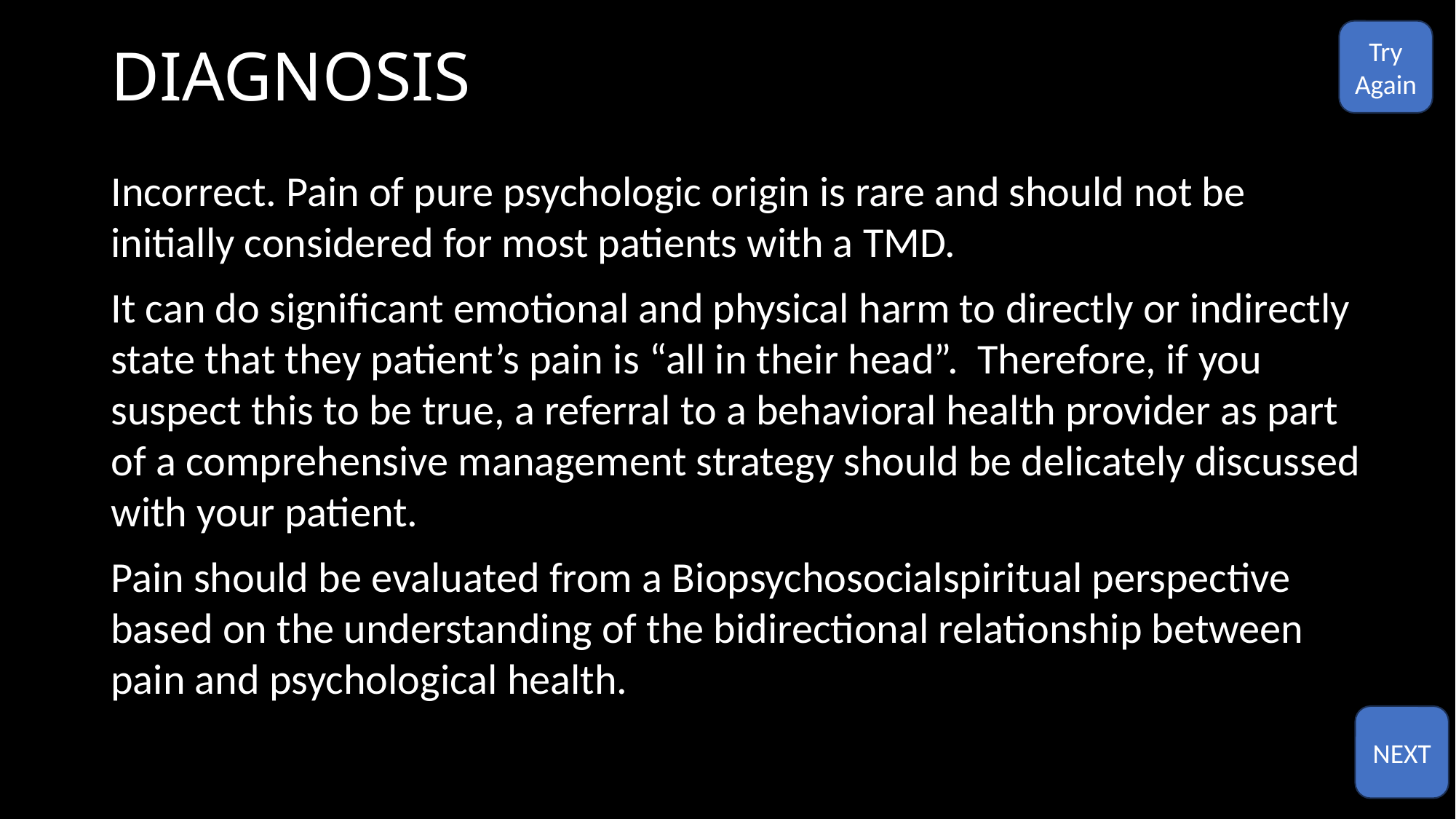

# DIAGNOSIS
Try
Again
Incorrect. Pain of pure psychologic origin is rare and should not be initially considered for most patients with a TMD.
It can do significant emotional and physical harm to directly or indirectly state that they patient’s pain is “all in their head”. Therefore, if you suspect this to be true, a referral to a behavioral health provider as part of a comprehensive management strategy should be delicately discussed with your patient.
Pain should be evaluated from a Biopsychosocialspiritual perspective based on the understanding of the bidirectional relationship between pain and psychological health.
NEXT

## Slide 153
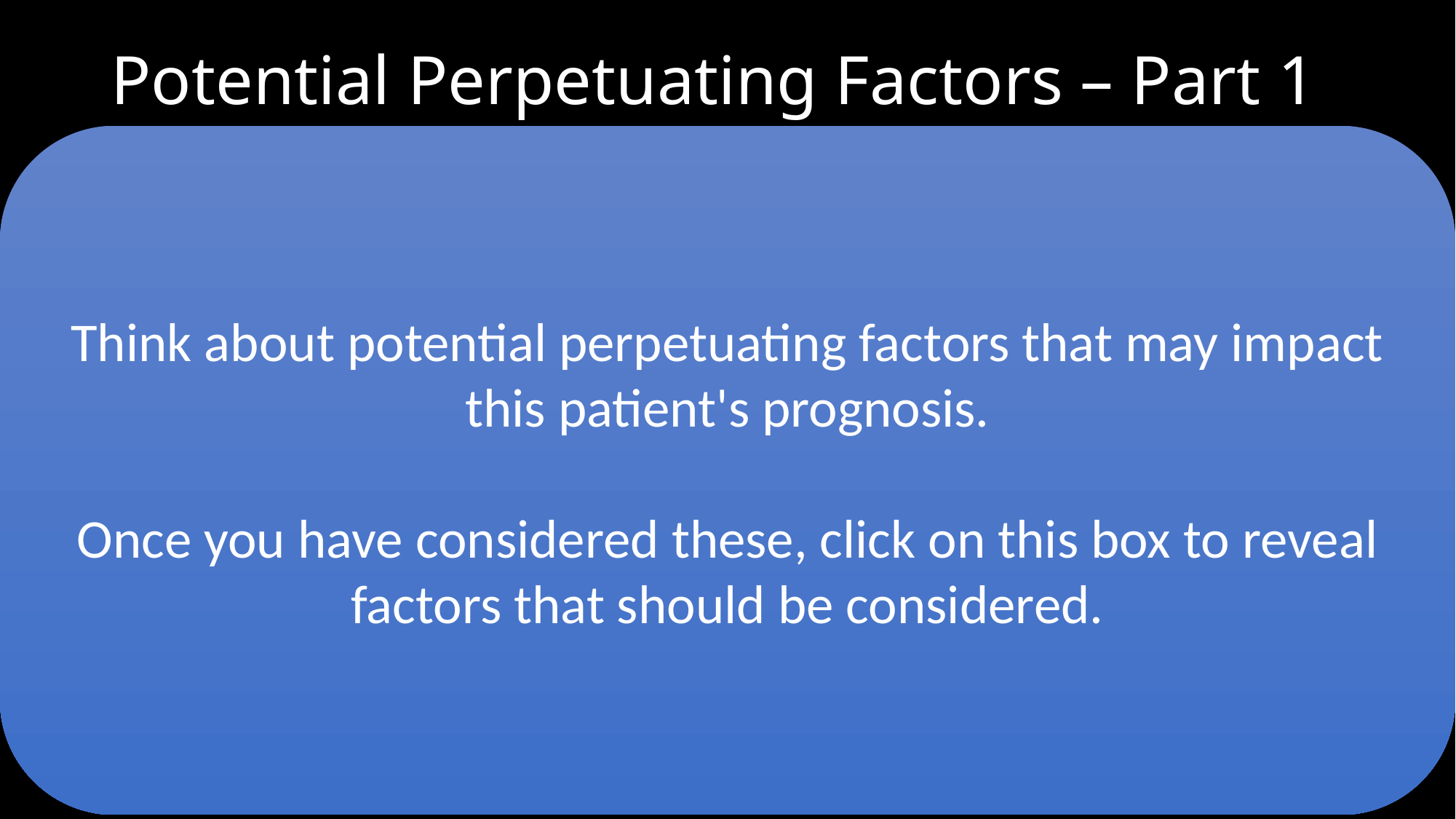

# Potential Perpetuating Factors – Part 1
Think about potential perpetuating factors that may impact this patient's prognosis.
Once you have considered these, click on this box to reveal factors that should be considered.
Oral Parafunction: Teeth together, Tongue to palate, nail biting
Poor Sleep Quality
Unhealthy sleep hygiene habits
History of Adverse Childhood Experiences (Abuse)
Has patient properly processed through these?
Psychological Health
Depression (adequately managed?)

## Slide 154
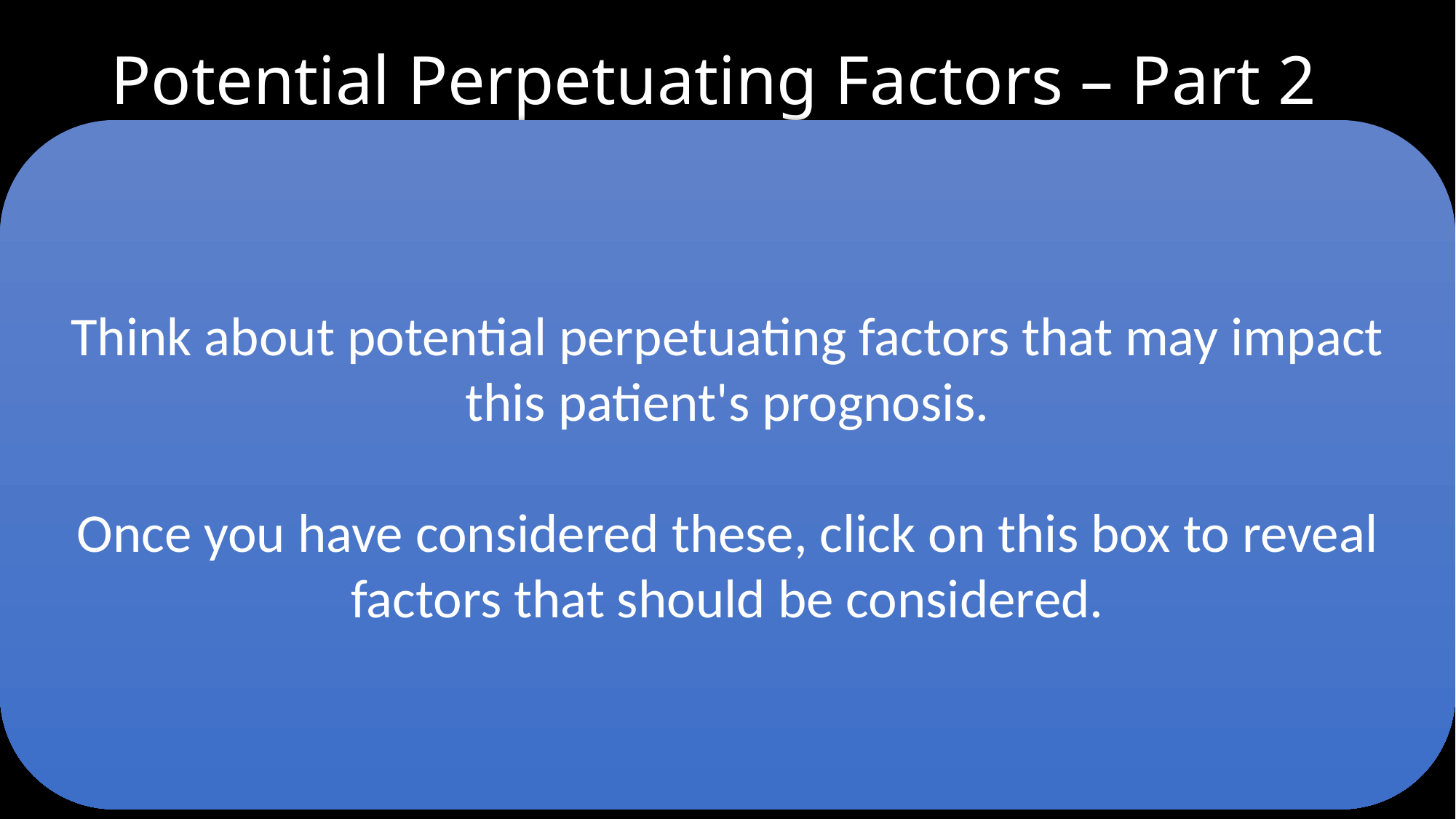

# Potential Perpetuating Factors – Part 2
History of Motor Vehicle Accident with Loss of Consciousness
Other Body Pain (Chronic Overlapping Pain Conditions)
Have these been evaluated / managed?
Nutrition
Unhealthy alcohol consumption
Unhealthy dietary intake
High BMI (overweight)
May be protective based on history of adverse childhood experience
Think about potential perpetuating factors that may impact this patient's prognosis.
Once you have considered these, click on this box to reveal factors that should be considered.

## Slide 155
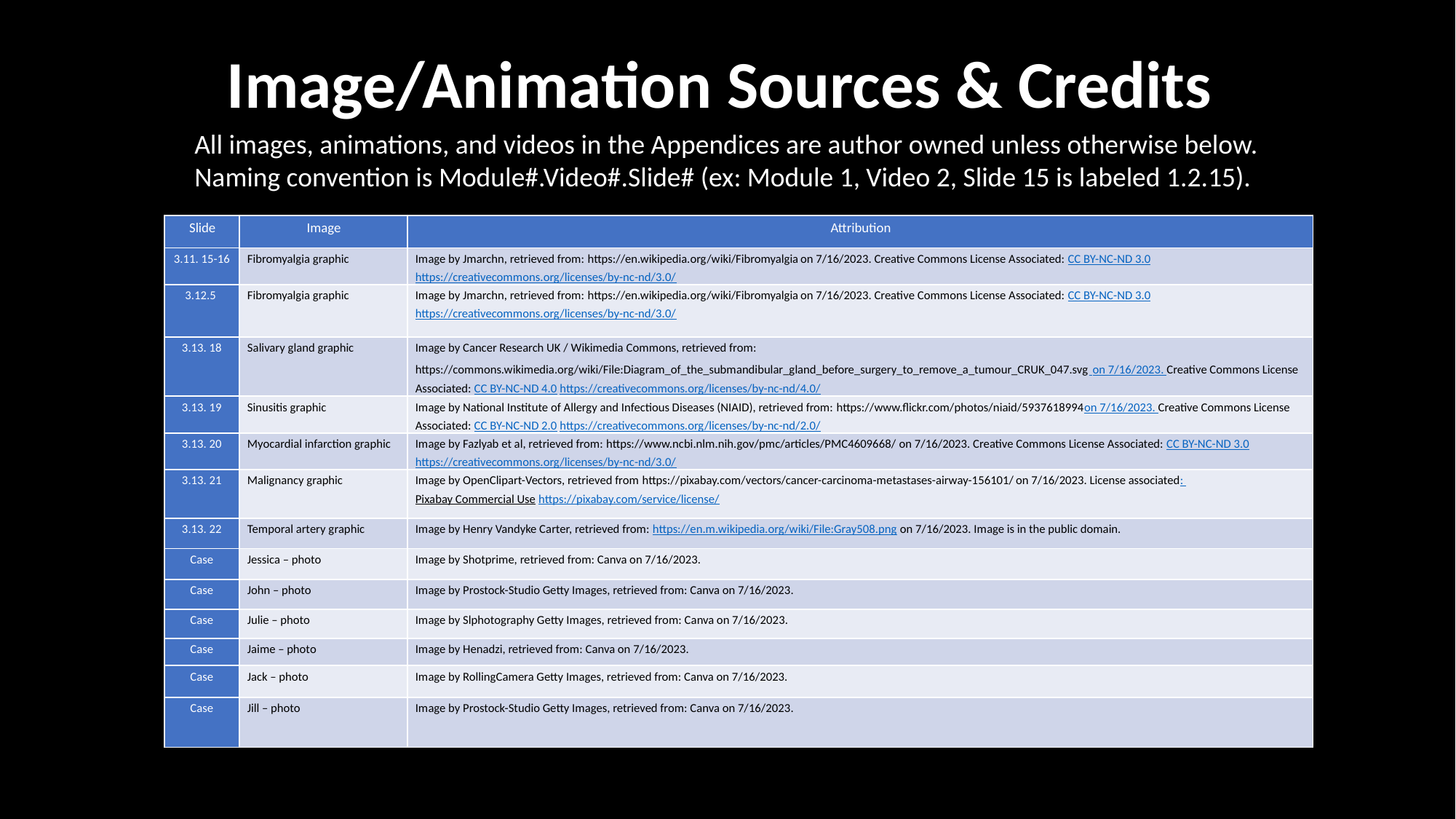

# Image/Animation Sources & Credits
All images, animations, and videos in the Appendices are author owned unless otherwise below. Naming convention is Module#.Video#.Slide# (ex: Module 1, Video 2, Slide 15 is labeled 1.2.15).
| Slide | Image | Attribution |
| --- | --- | --- |
| 3.11. 15-16 | Fibromyalgia graphic | Image by Jmarchn, retrieved from: https://en.wikipedia.org/wiki/Fibromyalgia on 7/16/2023. Creative Commons License Associated: CC BY-NC-ND 3.0 https://creativecommons.org/licenses/by-nc-nd/3.0/ |
| 3.12.5 | Fibromyalgia graphic | Image by Jmarchn, retrieved from: https://en.wikipedia.org/wiki/Fibromyalgia on 7/16/2023. Creative Commons License Associated: CC BY-NC-ND 3.0 https://creativecommons.org/licenses/by-nc-nd/3.0/ |
| 3.13. 18 | Salivary gland graphic | Image by Cancer Research UK / Wikimedia Commons, retrieved from: https://commons.wikimedia.org/wiki/File:Diagram\_of\_the\_submandibular\_gland\_before\_surgery\_to\_remove\_a\_tumour\_CRUK\_047.svg on 7/16/2023. Creative Commons License Associated: CC BY-NC-ND 4.0 https://creativecommons.org/licenses/by-nc-nd/4.0/ |
| 3.13. 19 | Sinusitis graphic | Image by National Institute of Allergy and Infectious Diseases (NIAID), retrieved from: https://www.flickr.com/photos/niaid/5937618994on 7/16/2023. Creative Commons License Associated: CC BY-NC-ND 2.0 https://creativecommons.org/licenses/by-nc-nd/2.0/ |
| 3.13. 20 | Myocardial infarction graphic | Image by Fazlyab et al, retrieved from: https://www.ncbi.nlm.nih.gov/pmc/articles/PMC4609668/ on 7/16/2023. Creative Commons License Associated: CC BY-NC-ND 3.0 https://creativecommons.org/licenses/by-nc-nd/3.0/ |
| 3.13. 21 | Malignancy graphic | Image by OpenClipart-Vectors, retrieved from https://pixabay.com/vectors/cancer-carcinoma-metastases-airway-156101/ on 7/16/2023. License associated: Pixabay Commercial Use https://pixabay.com/service/license/ |
| 3.13. 22 | Temporal artery graphic | Image by Henry Vandyke Carter, retrieved from: https://en.m.wikipedia.org/wiki/File:Gray508.png on 7/16/2023. Image is in the public domain. |
| Case | Jessica – photo | Image by Shotprime, retrieved from: Canva on 7/16/2023. |
| Case | John – photo | Image by Prostock-Studio Getty Images, retrieved from: Canva on 7/16/2023. |
| Case | Julie – photo | Image by Slphotography Getty Images, retrieved from: Canva on 7/16/2023. |
| Case | Jaime – photo | Image by Henadzi, retrieved from: Canva on 7/16/2023. |
| Case | Jack – photo | Image by RollingCamera Getty Images, retrieved from: Canva on 7/16/2023. |
| Case | Jill – photo | Image by Prostock-Studio Getty Images, retrieved from: Canva on 7/16/2023. |

## Slide 156
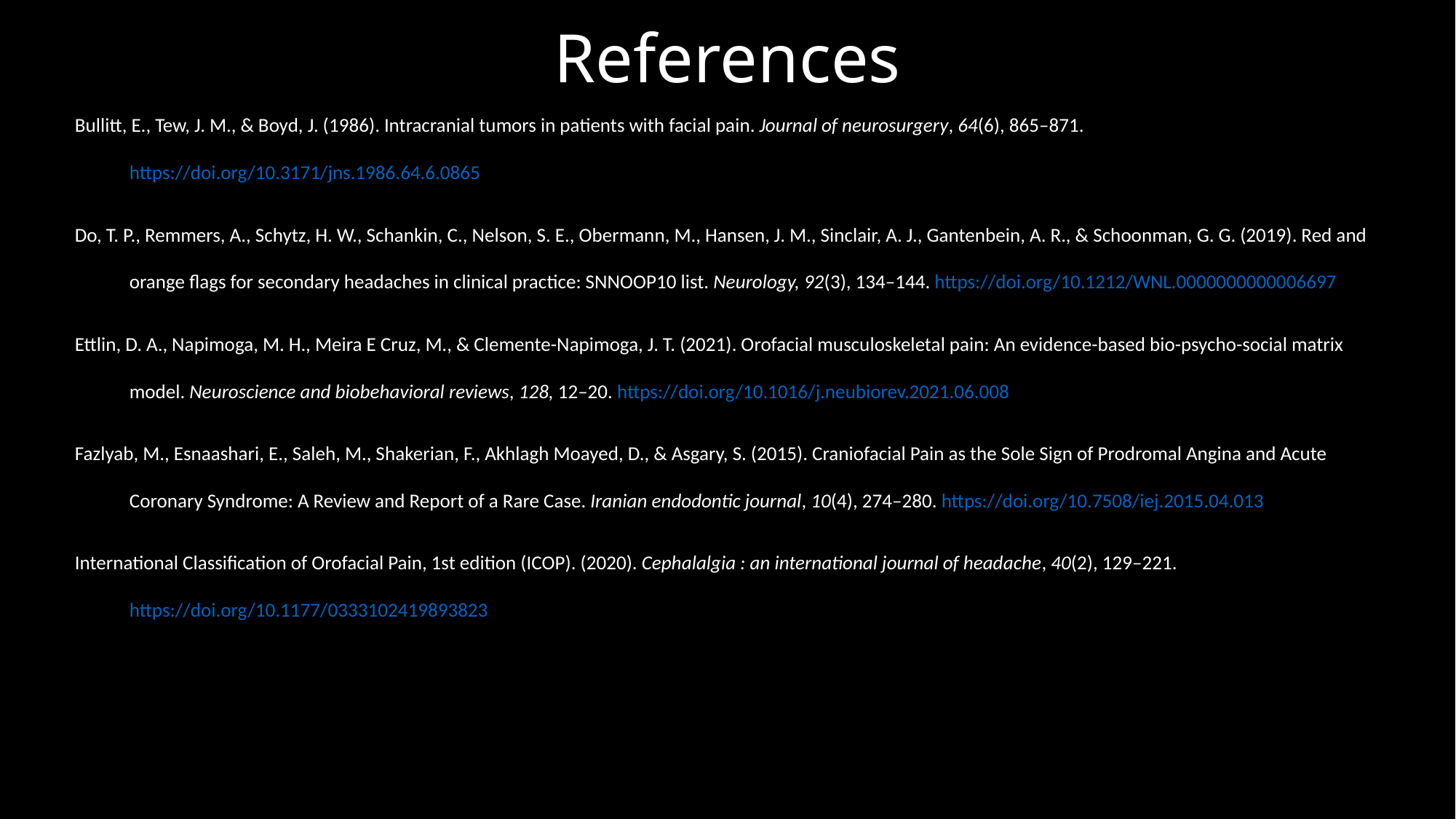

# References
Bullitt, E., Tew, J. M., & Boyd, J. (1986). Intracranial tumors in patients with facial pain. Journal of neurosurgery, 64(6), 865–871. https://doi.org/10.3171/jns.1986.64.6.0865
Do, T. P., Remmers, A., Schytz, H. W., Schankin, C., Nelson, S. E., Obermann, M., Hansen, J. M., Sinclair, A. J., Gantenbein, A. R., & Schoonman, G. G. (2019). Red and orange flags for secondary headaches in clinical practice: SNNOOP10 list. Neurology, 92(3), 134–144. https://doi.org/10.1212/WNL.0000000000006697
Ettlin, D. A., Napimoga, M. H., Meira E Cruz, M., & Clemente-Napimoga, J. T. (2021). Orofacial musculoskeletal pain: An evidence-based bio-psycho-social matrix model. Neuroscience and biobehavioral reviews, 128, 12–20. https://doi.org/10.1016/j.neubiorev.2021.06.008
Fazlyab, M., Esnaashari, E., Saleh, M., Shakerian, F., Akhlagh Moayed, D., & Asgary, S. (2015). Craniofacial Pain as the Sole Sign of Prodromal Angina and Acute Coronary Syndrome: A Review and Report of a Rare Case. Iranian endodontic journal, 10(4), 274–280. https://doi.org/10.7508/iej.2015.04.013
International Classification of Orofacial Pain, 1st edition (ICOP). (2020). Cephalalgia : an international journal of headache, 40(2), 129–221. https://doi.org/10.1177/0333102419893823

## Slide 157
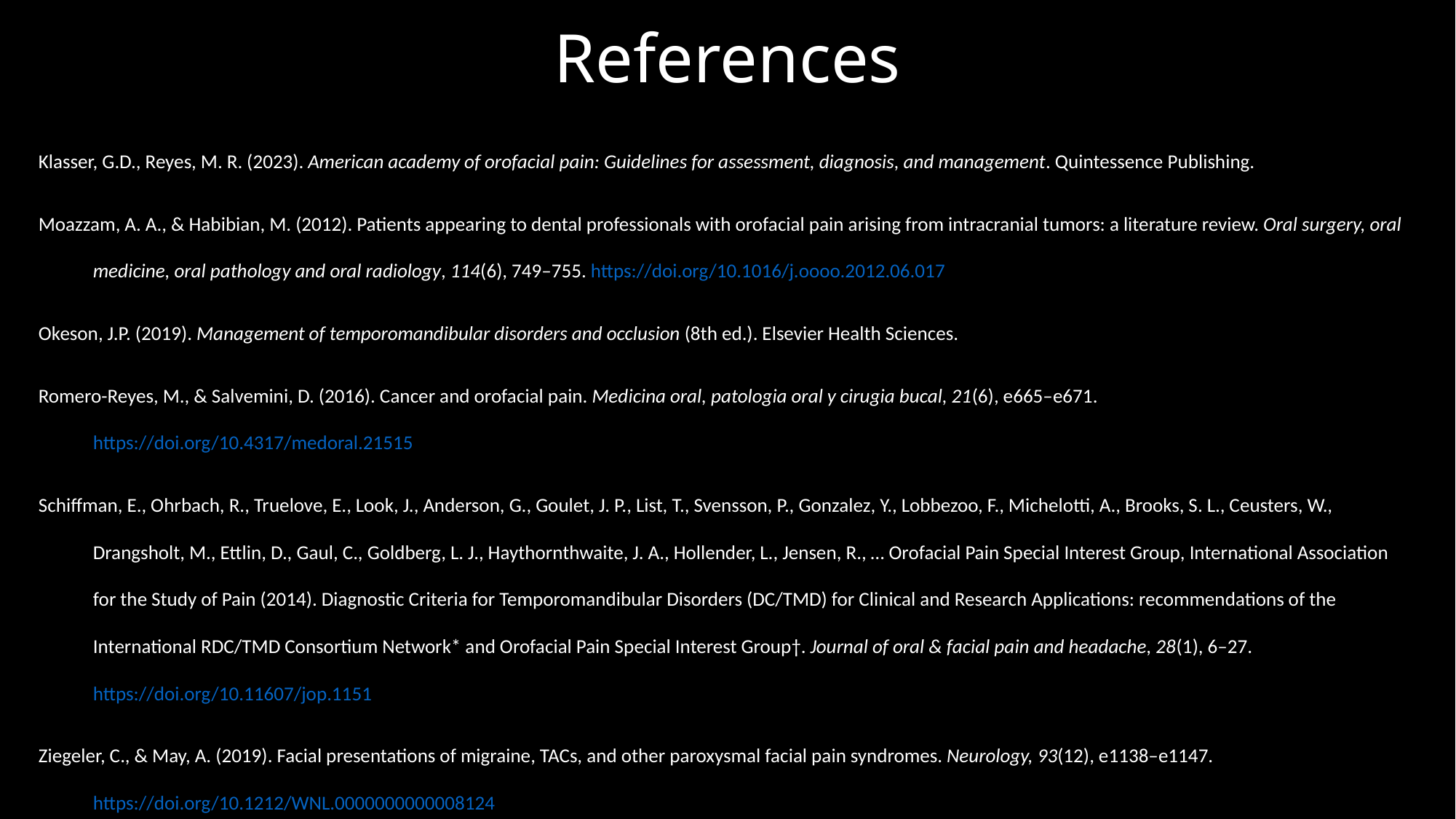

# References
Klasser, G.D., Reyes, M. R. (2023). American academy of orofacial pain: Guidelines for assessment, diagnosis, and management. Quintessence Publishing.
Moazzam, A. A., & Habibian, M. (2012). Patients appearing to dental professionals with orofacial pain arising from intracranial tumors: a literature review. Oral surgery, oral medicine, oral pathology and oral radiology, 114(6), 749–755. https://doi.org/10.1016/j.oooo.2012.06.017
Okeson, J.P. (2019). Management of temporomandibular disorders and occlusion (8th ed.). Elsevier Health Sciences.
Romero-Reyes, M., & Salvemini, D. (2016). Cancer and orofacial pain. Medicina oral, patologia oral y cirugia bucal, 21(6), e665–e671. https://doi.org/10.4317/medoral.21515
Schiffman, E., Ohrbach, R., Truelove, E., Look, J., Anderson, G., Goulet, J. P., List, T., Svensson, P., Gonzalez, Y., Lobbezoo, F., Michelotti, A., Brooks, S. L., Ceusters, W., Drangsholt, M., Ettlin, D., Gaul, C., Goldberg, L. J., Haythornthwaite, J. A., Hollender, L., Jensen, R., … Orofacial Pain Special Interest Group, International Association for the Study of Pain (2014). Diagnostic Criteria for Temporomandibular Disorders (DC/TMD) for Clinical and Research Applications: recommendations of the International RDC/TMD Consortium Network* and Orofacial Pain Special Interest Group†. Journal of oral & facial pain and headache, 28(1), 6–27. https://doi.org/10.11607/jop.1151
Ziegeler, C., & May, A. (2019). Facial presentations of migraine, TACs, and other paroxysmal facial pain syndromes. Neurology, 93(12), e1138–e1147. https://doi.org/10.1212/WNL.0000000000008124
