# Supplementary material for: Enhancing Temporomandibular Disorders Education for Initial Care Clinicians Through Interprofessional Education
Source: MedEdPORTAL. 2024 Nov 19;20:11467. doi: 10.15766/mep_2374-8265.11467 (PMC11575917; doi:10.15766/mep_2374-8265.11467)
Supplement: Supplementary file 1 — Facilitator Guide.docxLearner Guide and Clinical Tools.pdfModule 1 - TMD Pathophysiology.pptxModule 2 - TMD Assessment.pptxModule 3 - TMD Diagnosis.pptxModule 4 - TMD Management.pptxSample Patient Education Tools.pptx [file mep_2374-8265.11467-s001.zip › G. Sample Patient Education Tools.pptx]

## Slide 1
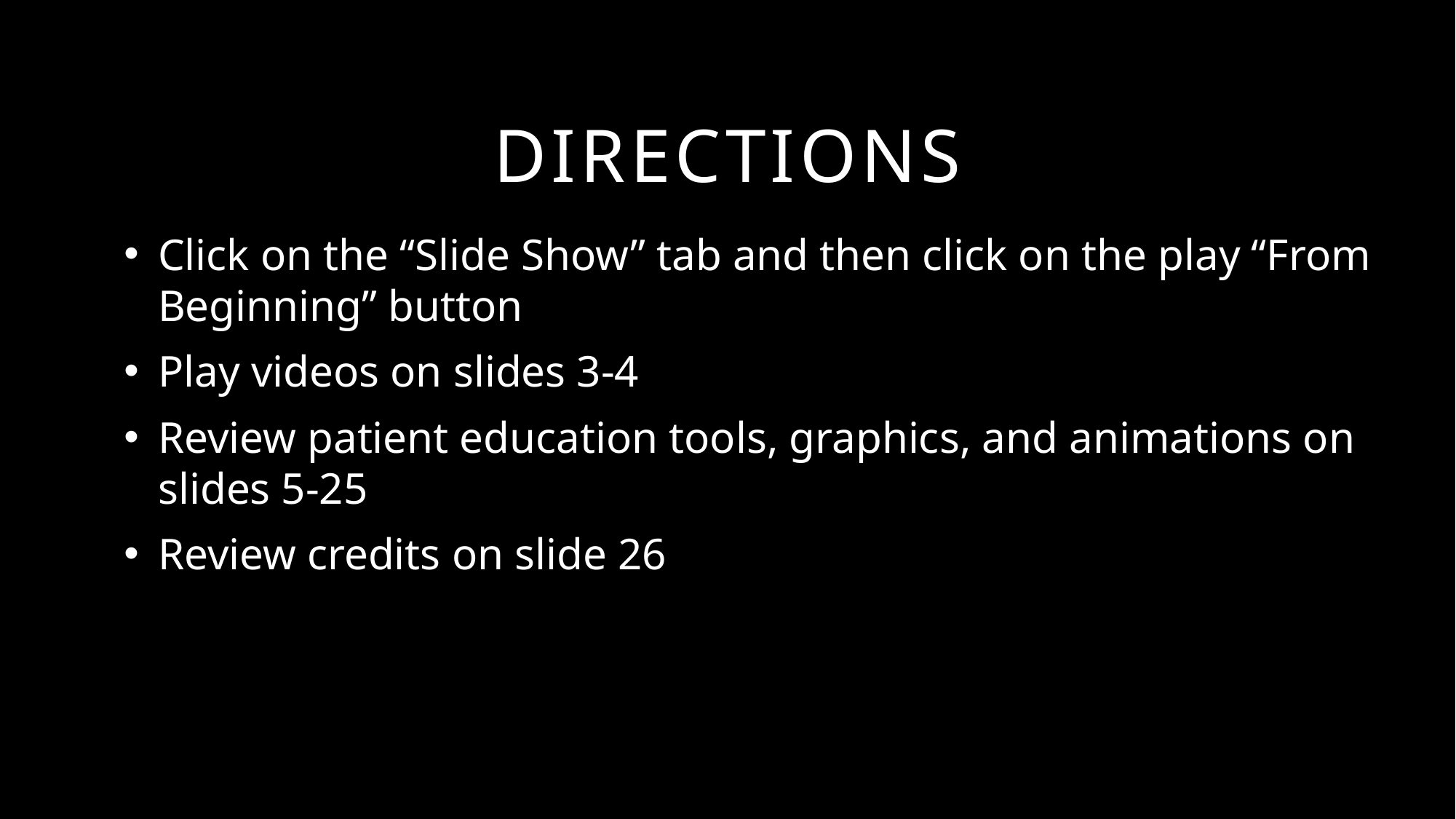

Directions
Click on the “Slide Show” tab and then click on the play “From Beginning” button
Play videos on slides 3-4
Review patient education tools, graphics, and animations on slides 5-25
Review credits on slide 26

## Slide 2
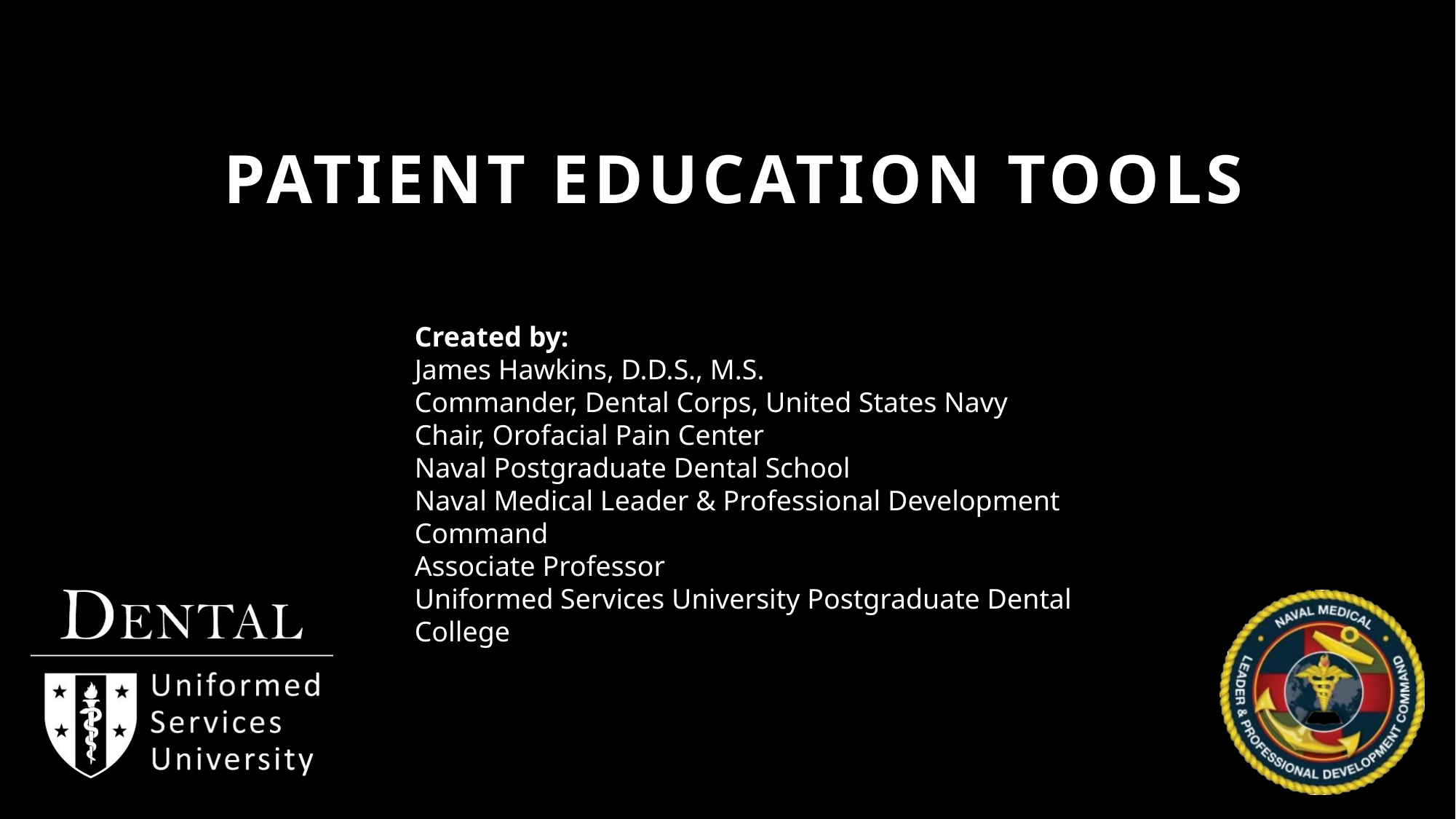

# Patient Education Tools
Created by:
James Hawkins, D.D.S., M.S.
Commander, Dental Corps, United States Navy
Chair, Orofacial Pain Center
Naval Postgraduate Dental School
Naval Medical Leader & Professional Development Command
Associate Professor
Uniformed Services University Postgraduate Dental College

## Slide 3
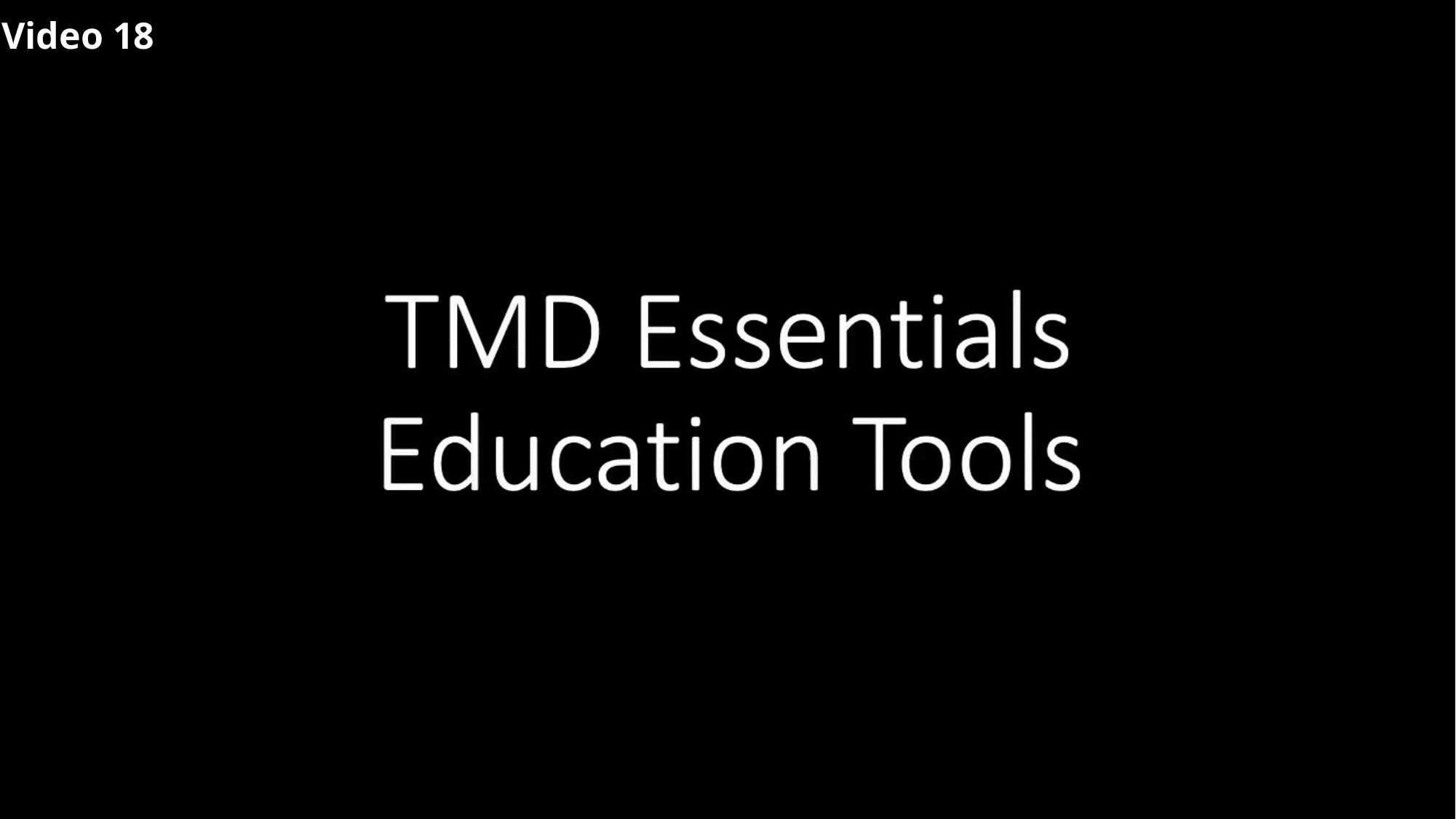

Video 18

## Slide 4
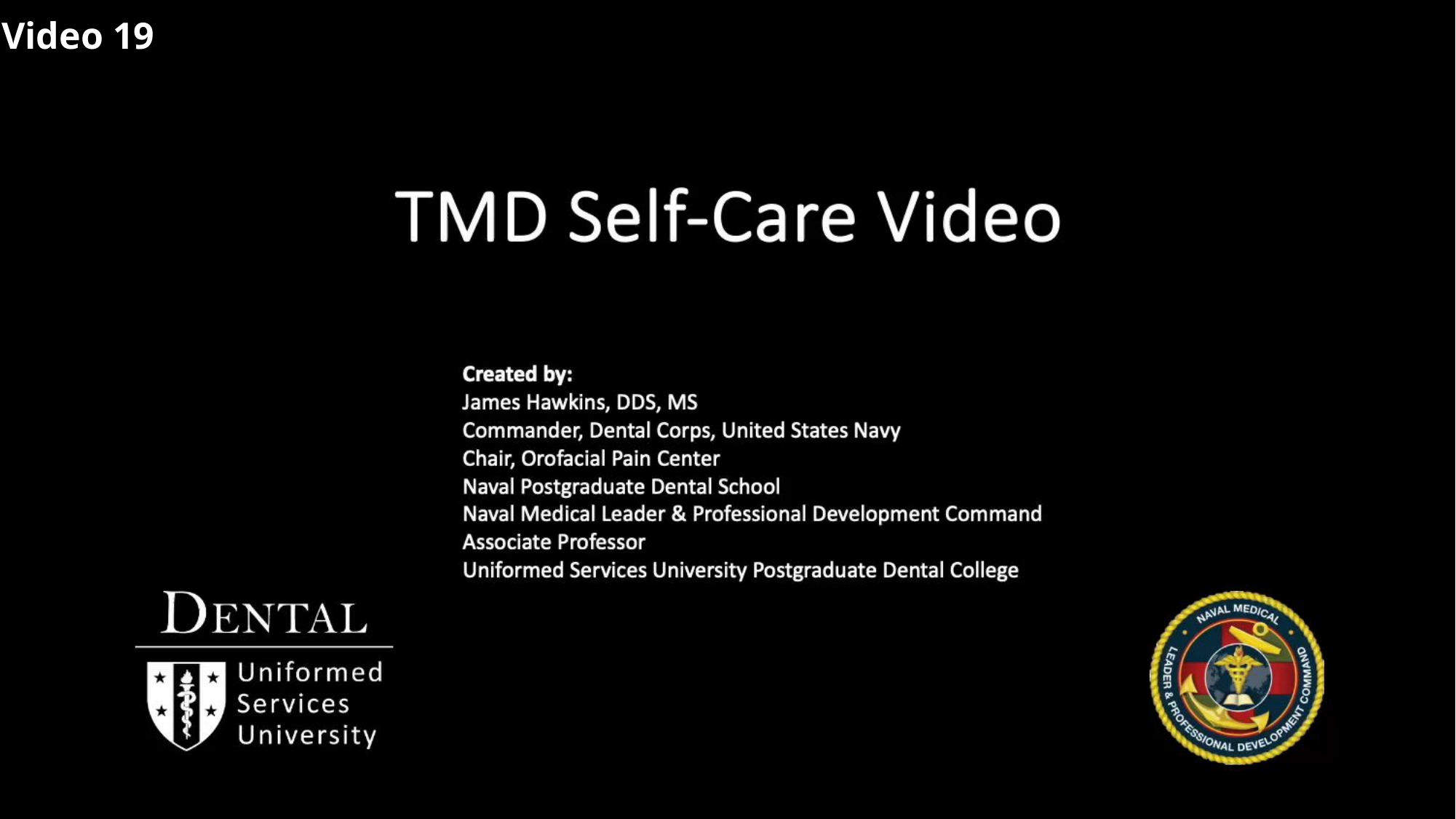

Video 19

## Slide 5
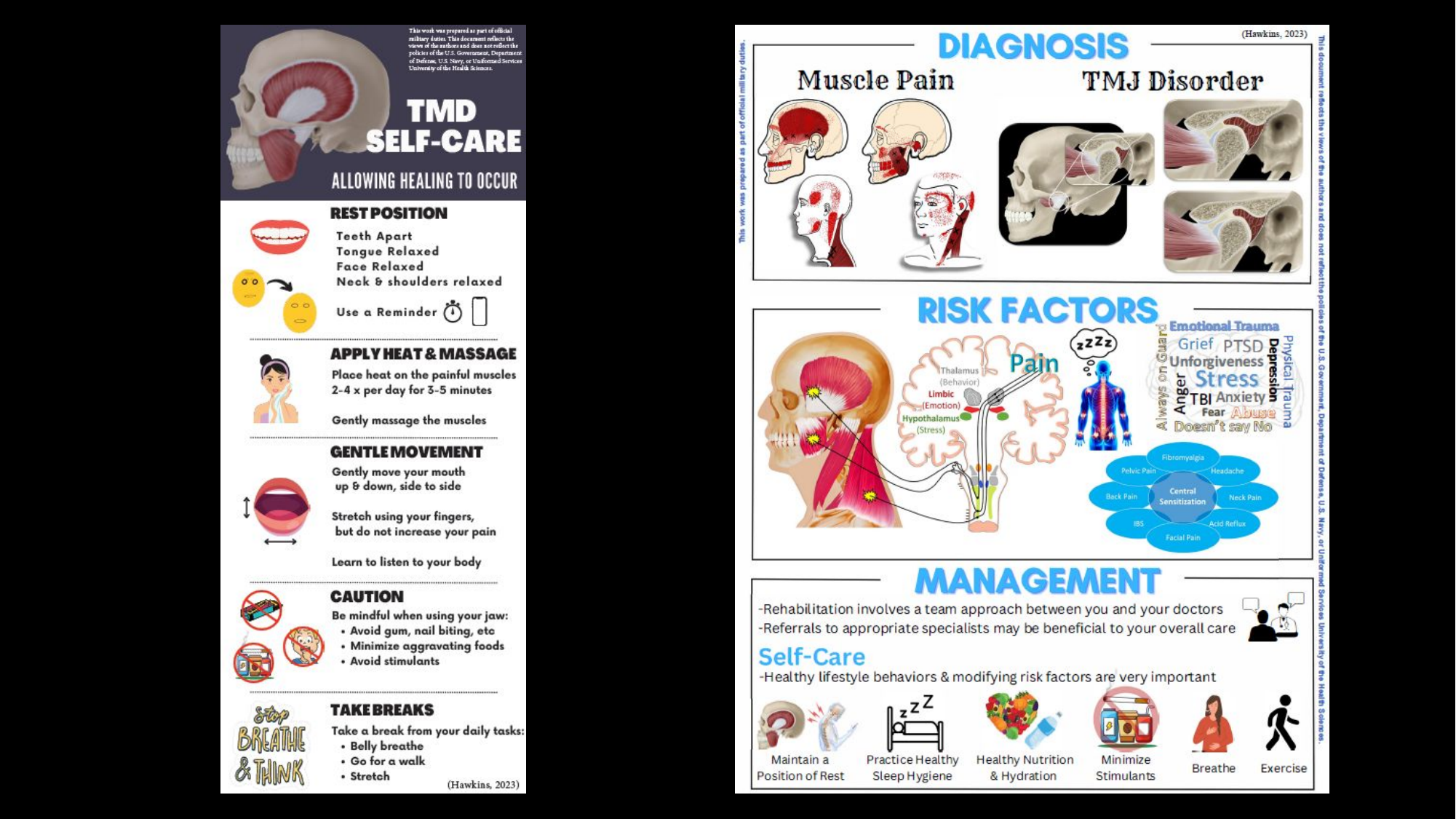

## Slide 6
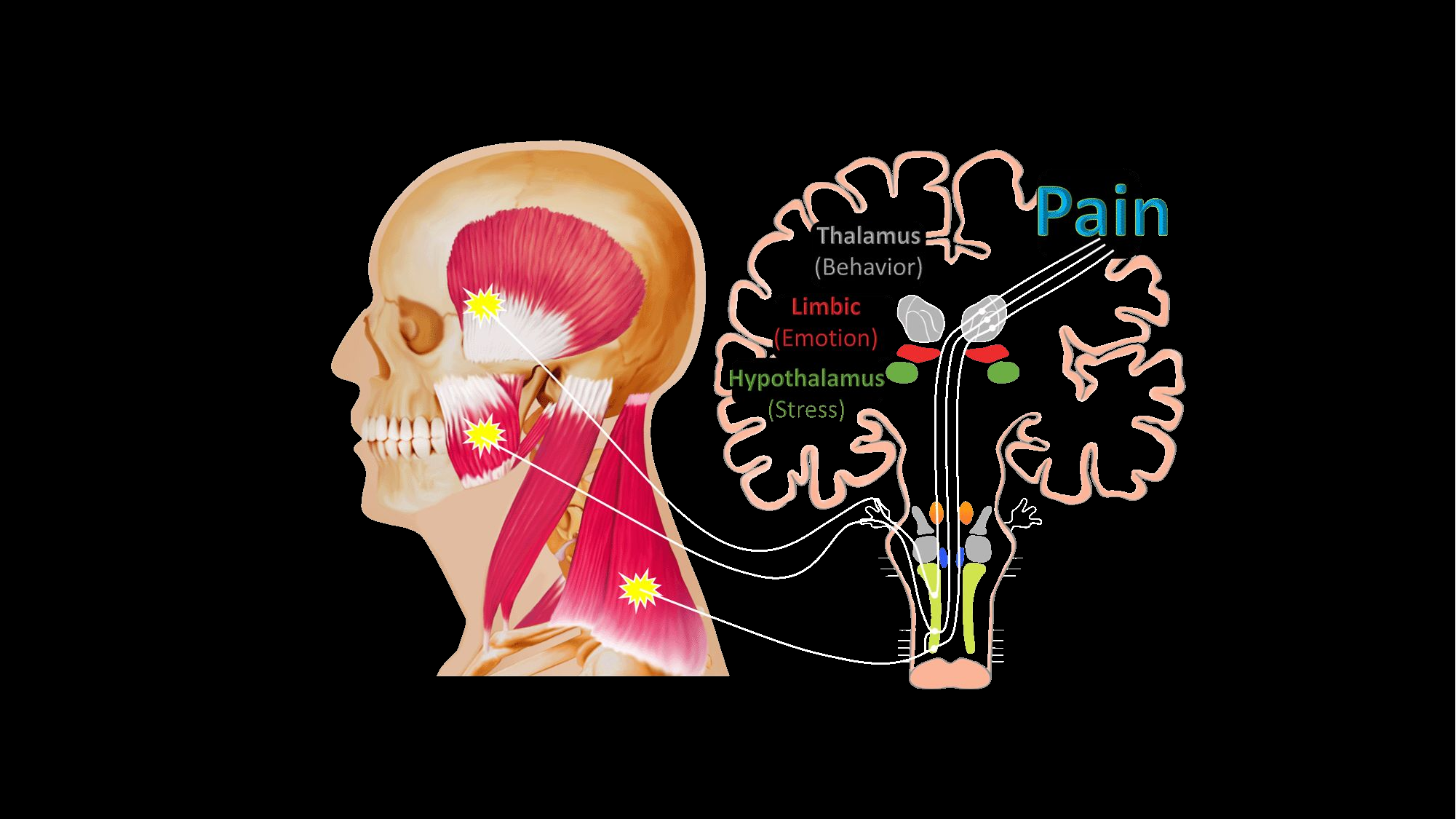

## Slide 7
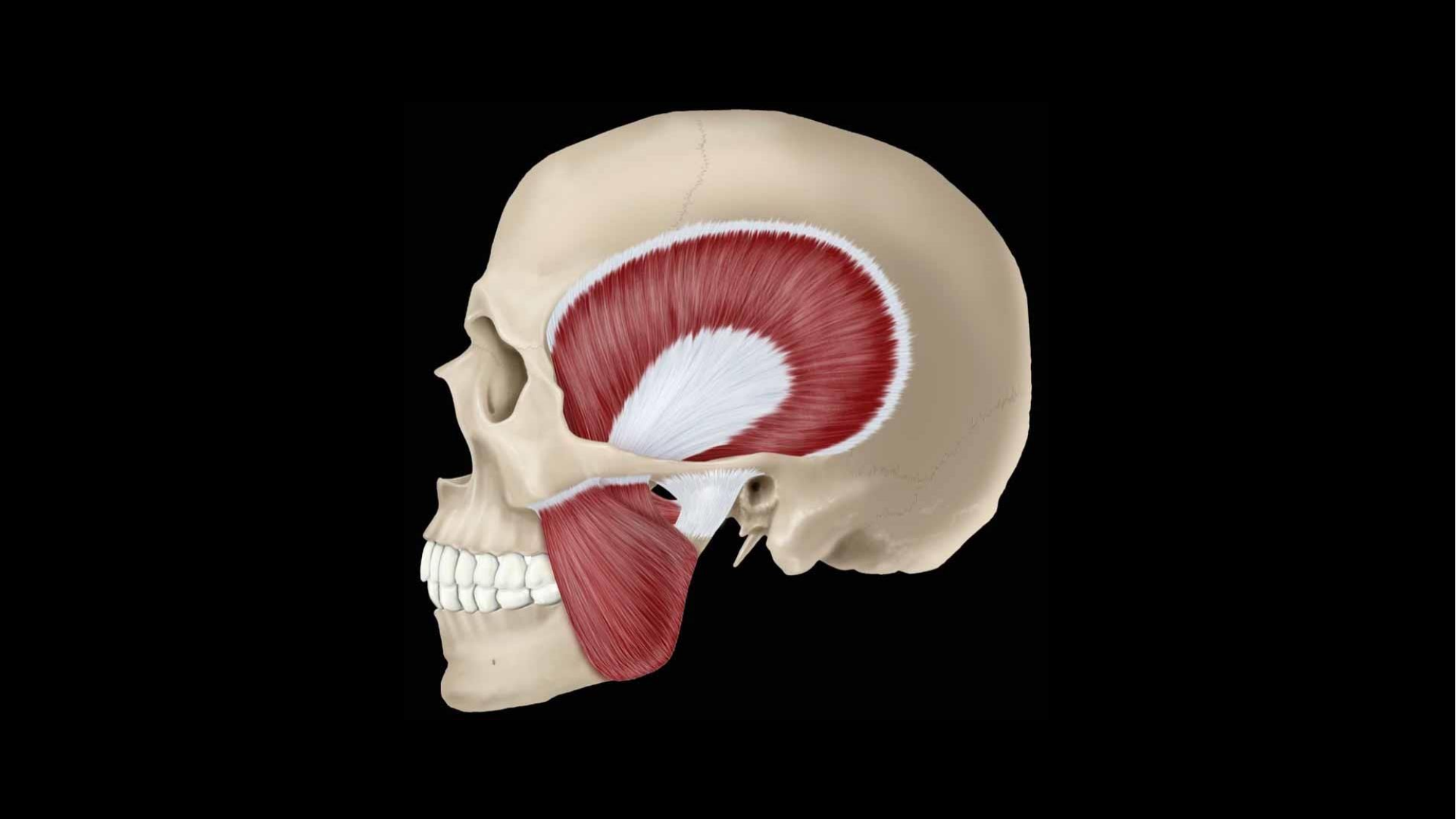

# Masticatory Muscle Disorders

## Slide 8
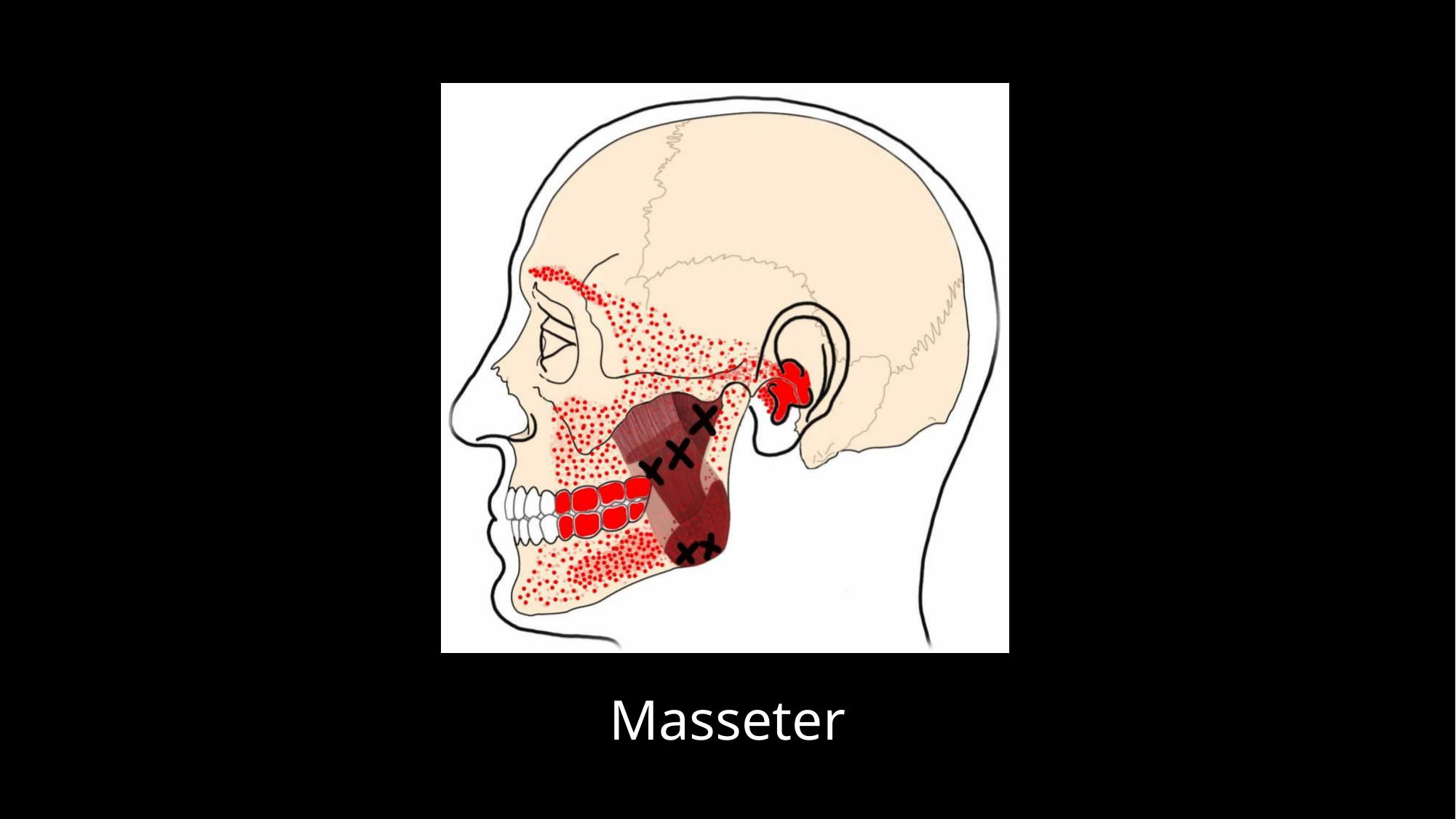

Masseter

## Slide 9
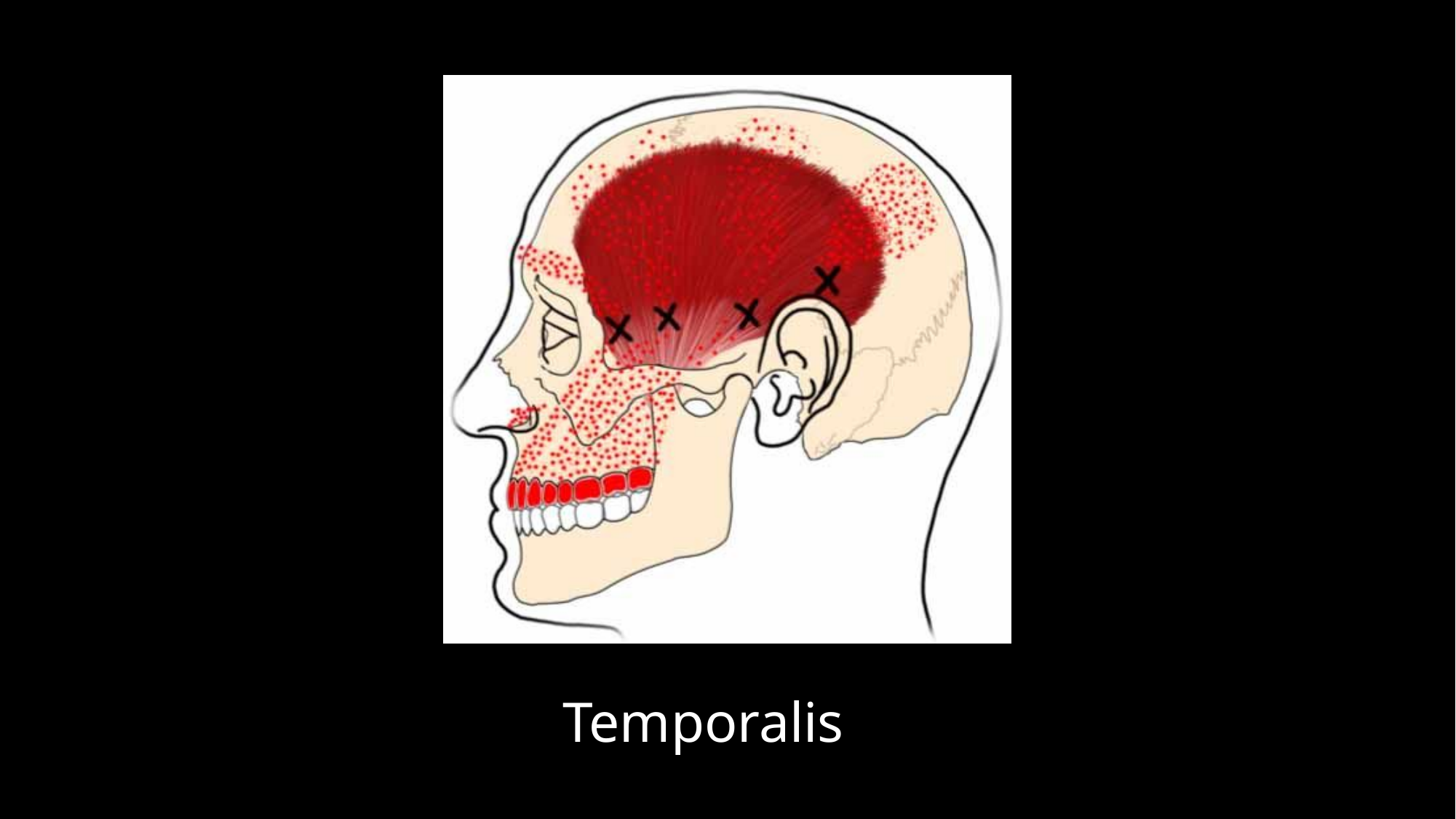

Temporalis

## Slide 10
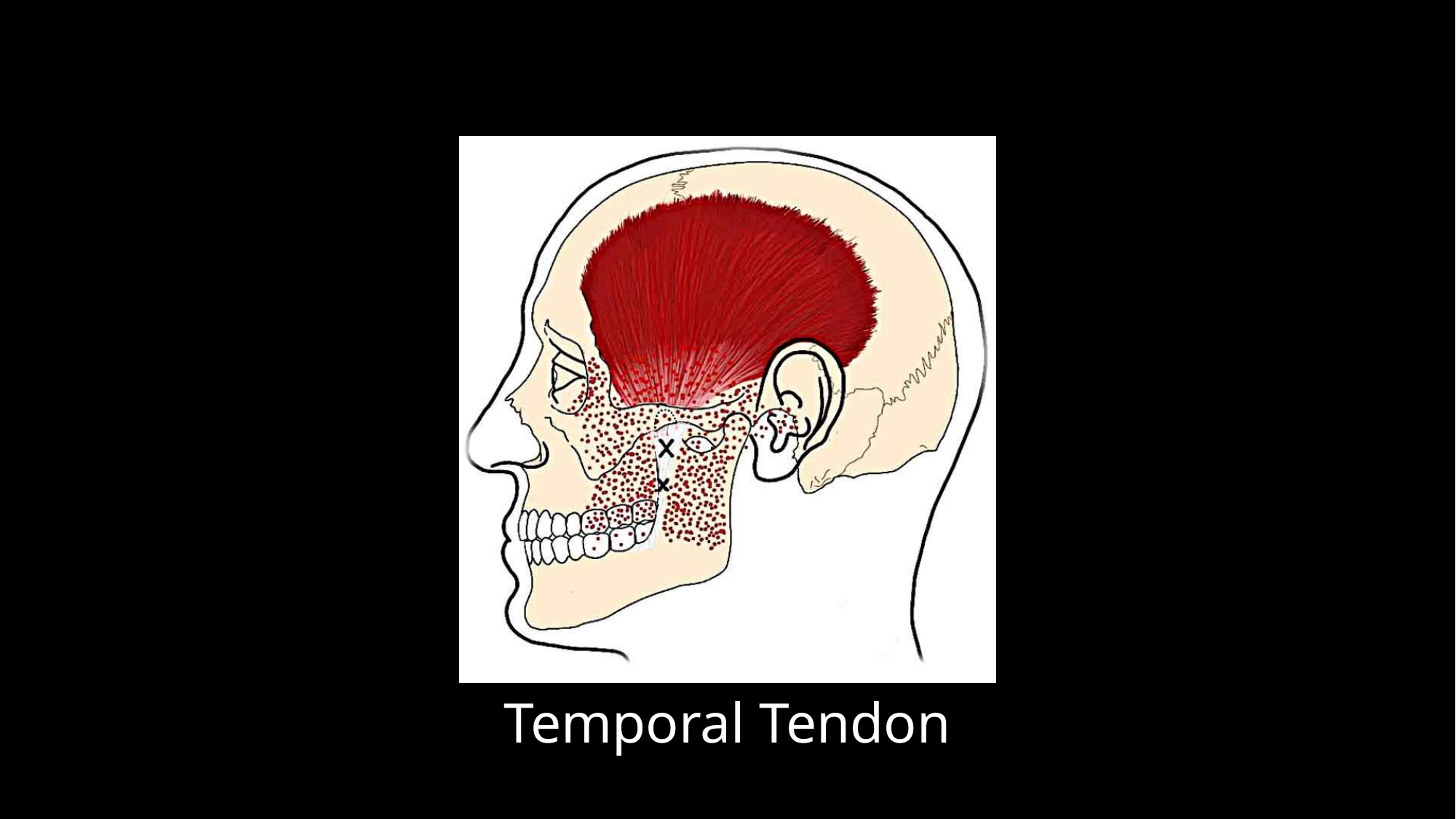

Temporal Tendon

## Slide 11
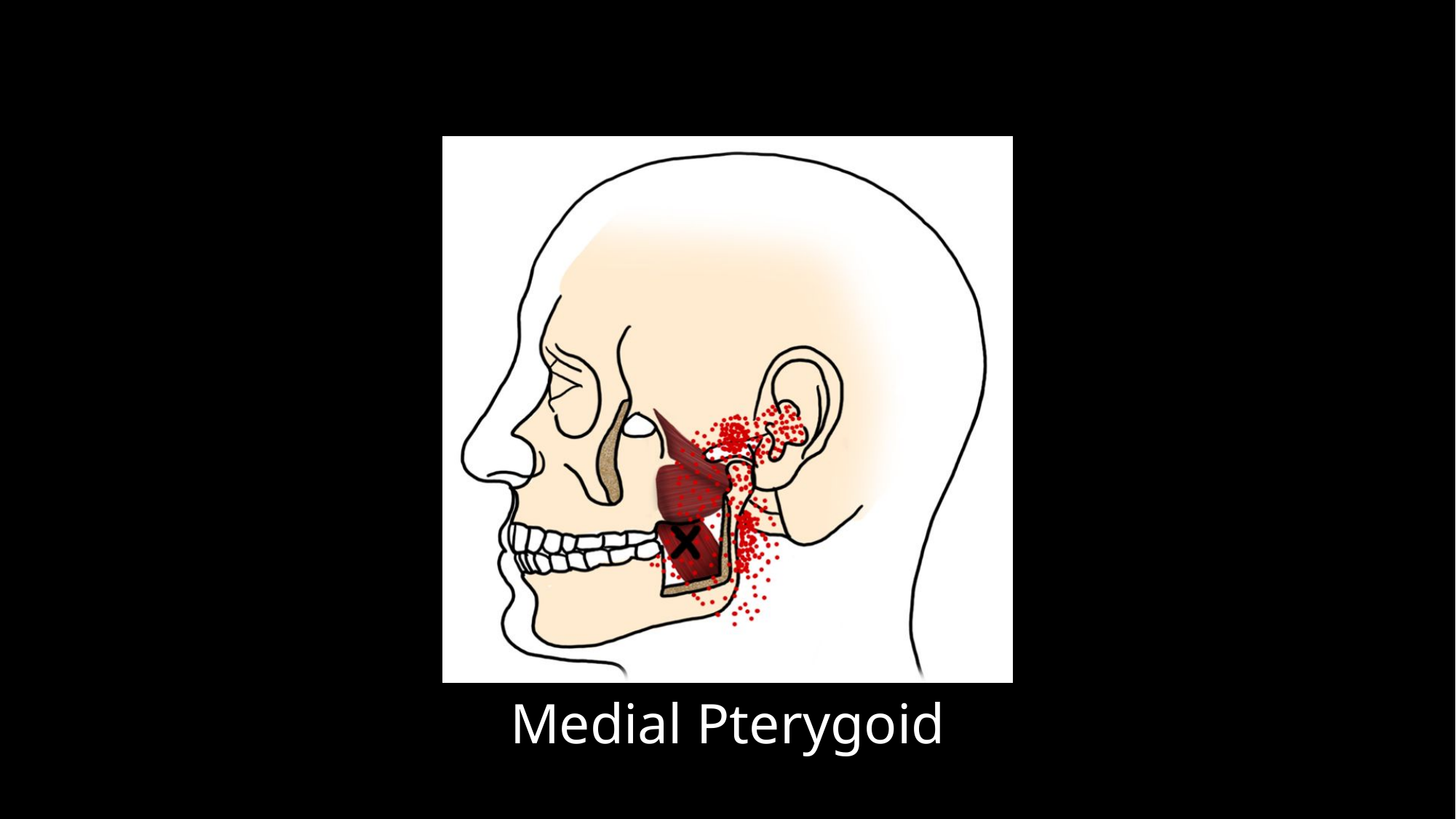

Medial Pterygoid

## Slide 12
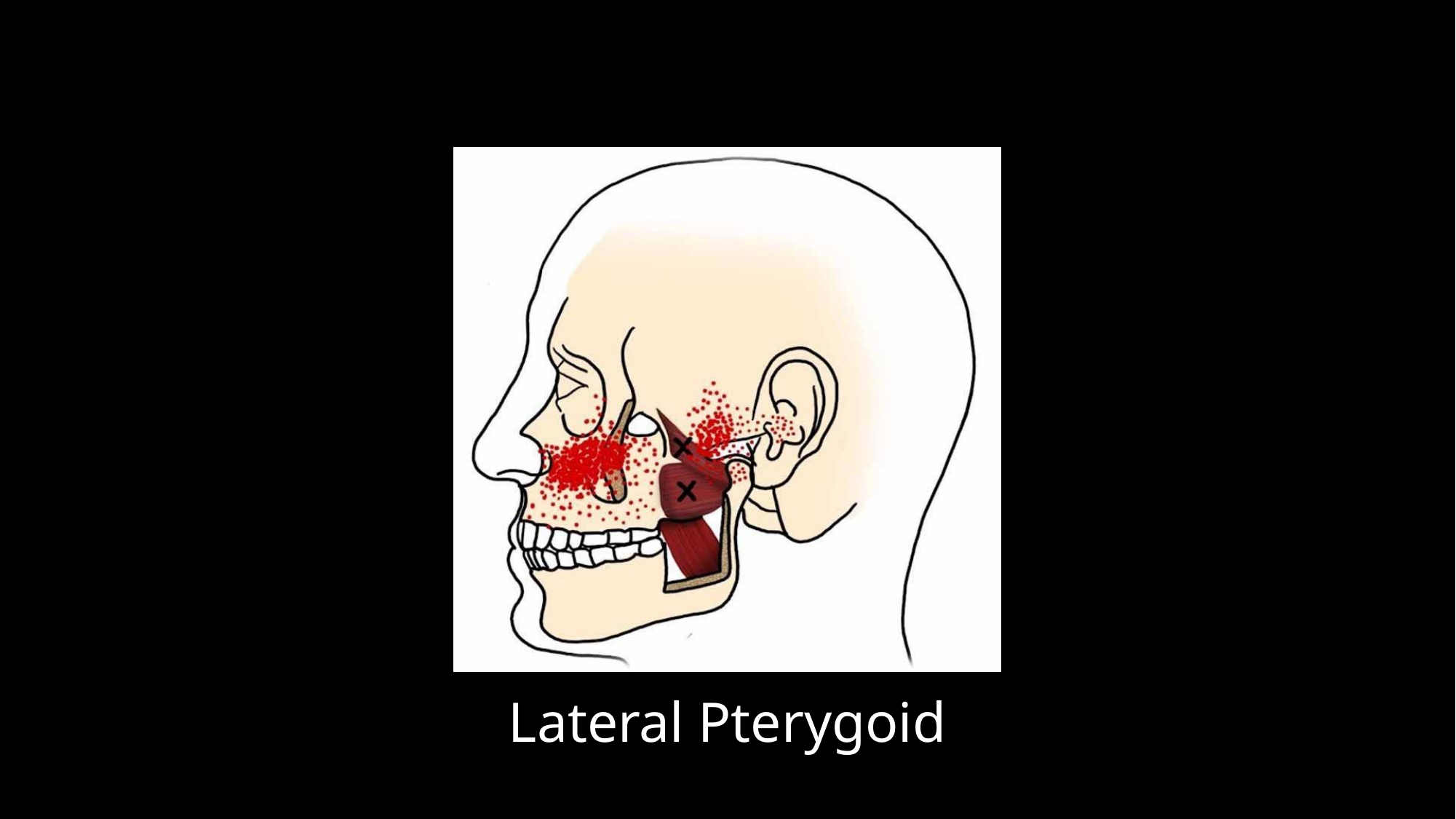

Lateral Pterygoid

## Slide 13
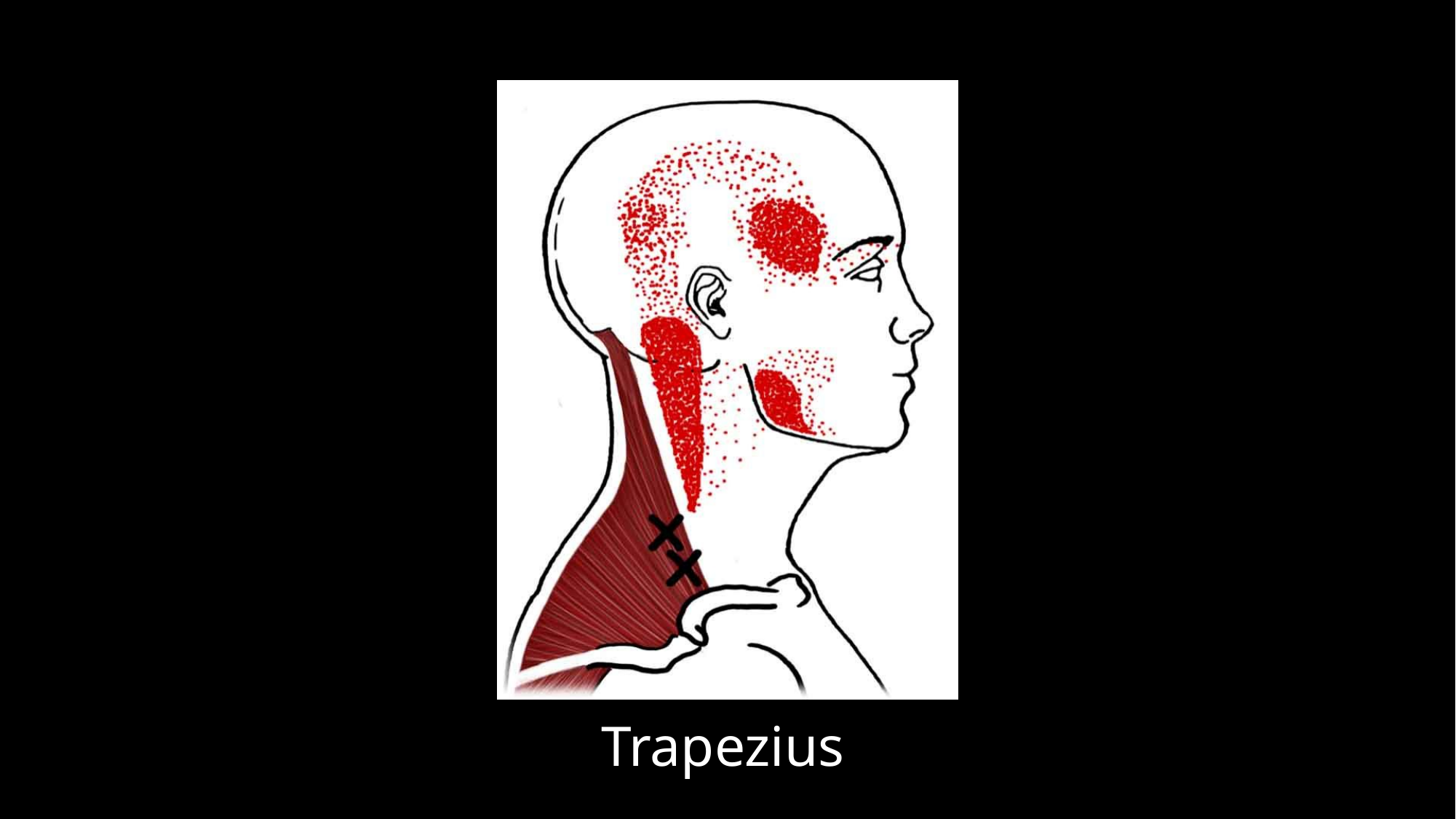

Trapezius

## Slide 14
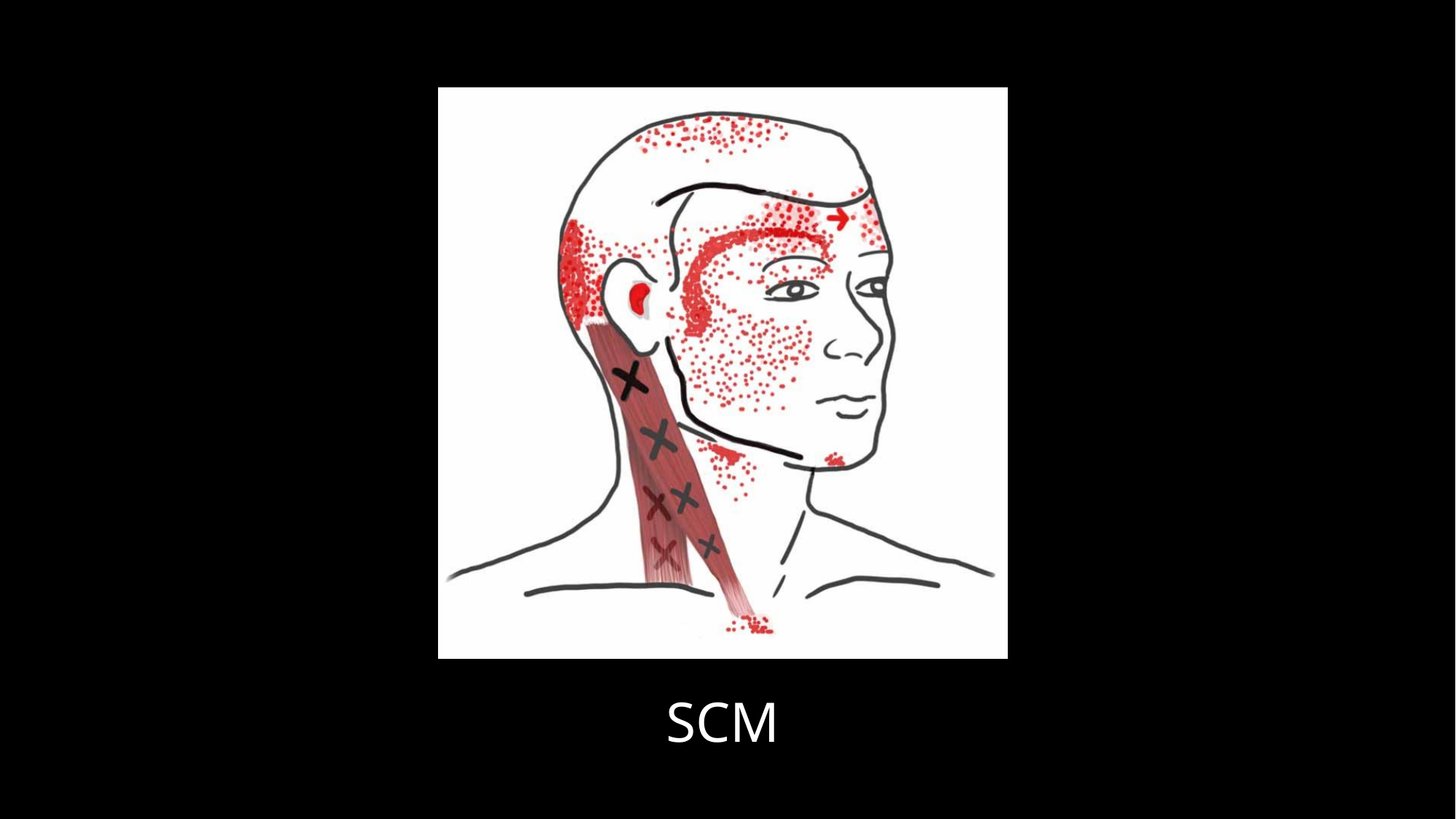

SCM

## Slide 15
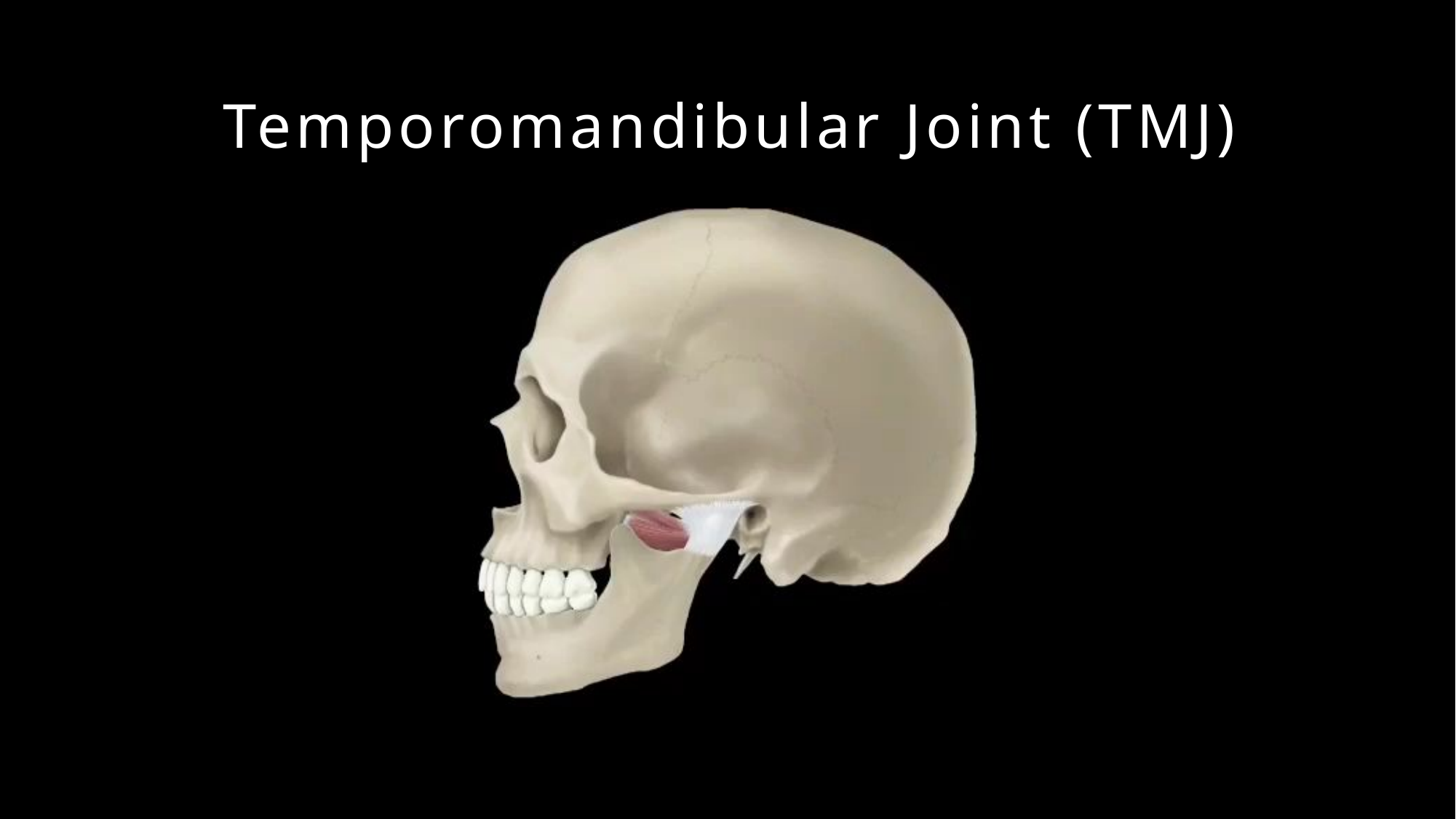

Temporomandibular Joint (TMJ)

## Slide 16
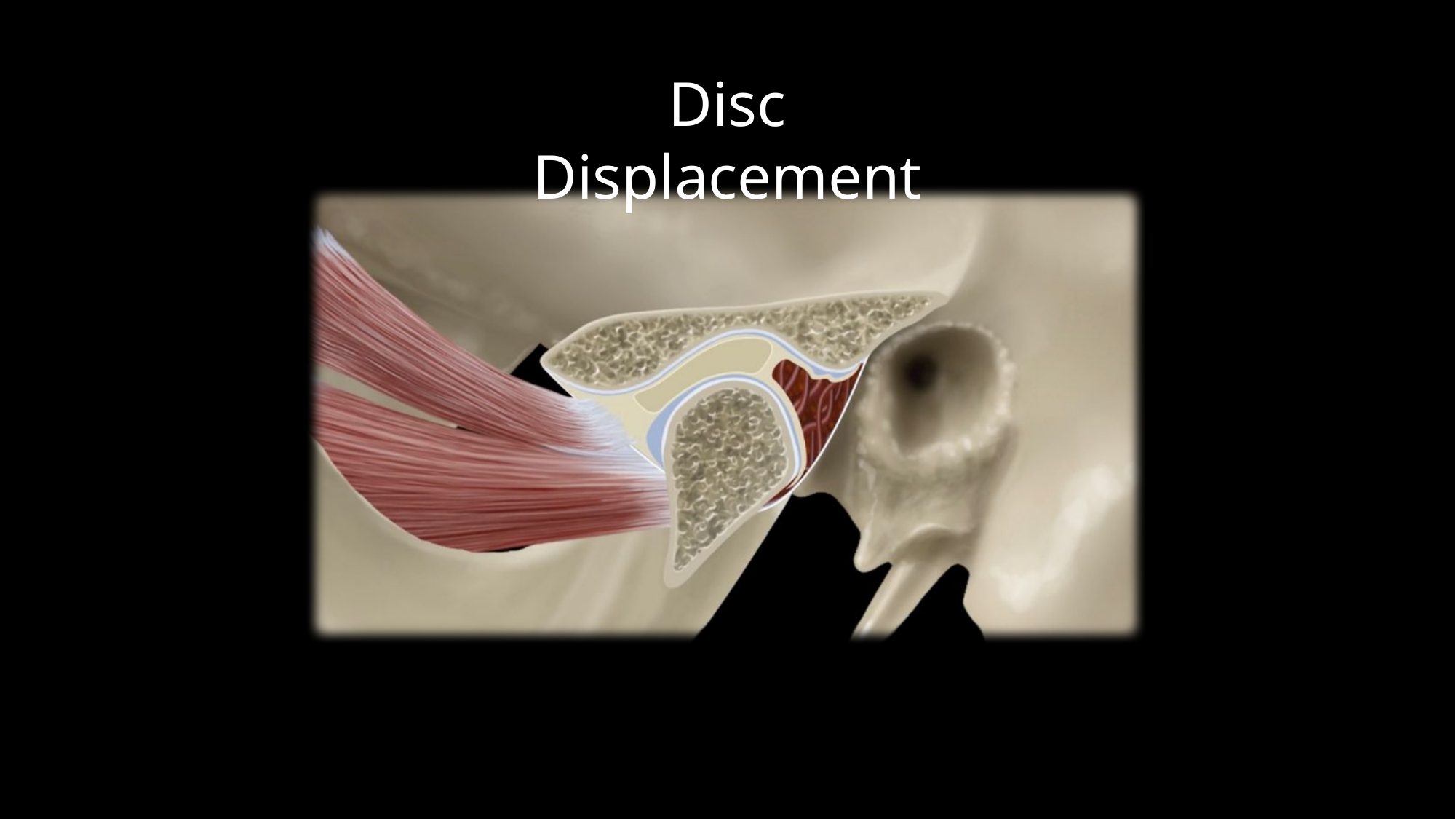

Disc Displacement

## Slide 17
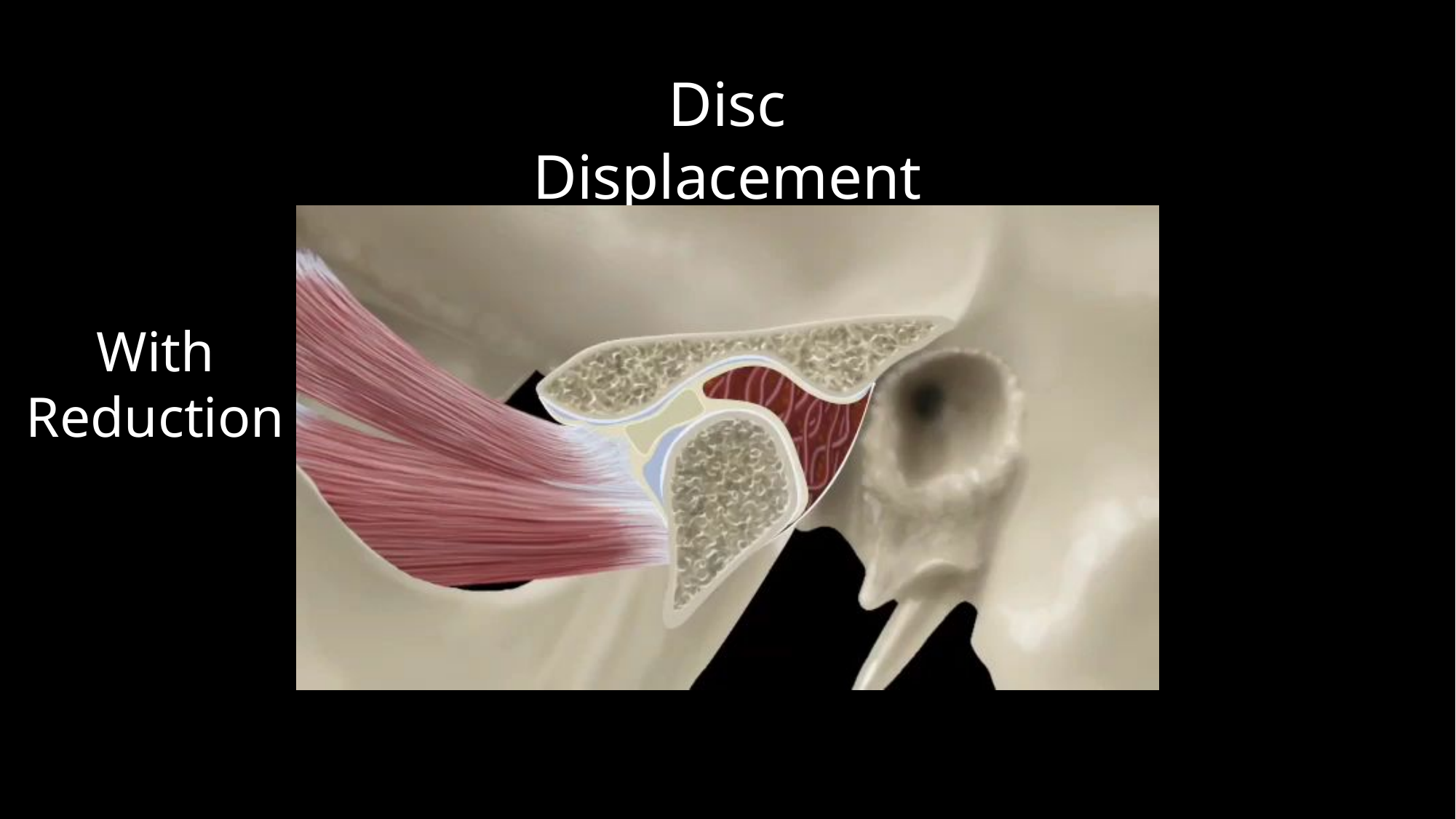

Disc Displacement
With Reduction

## Slide 18
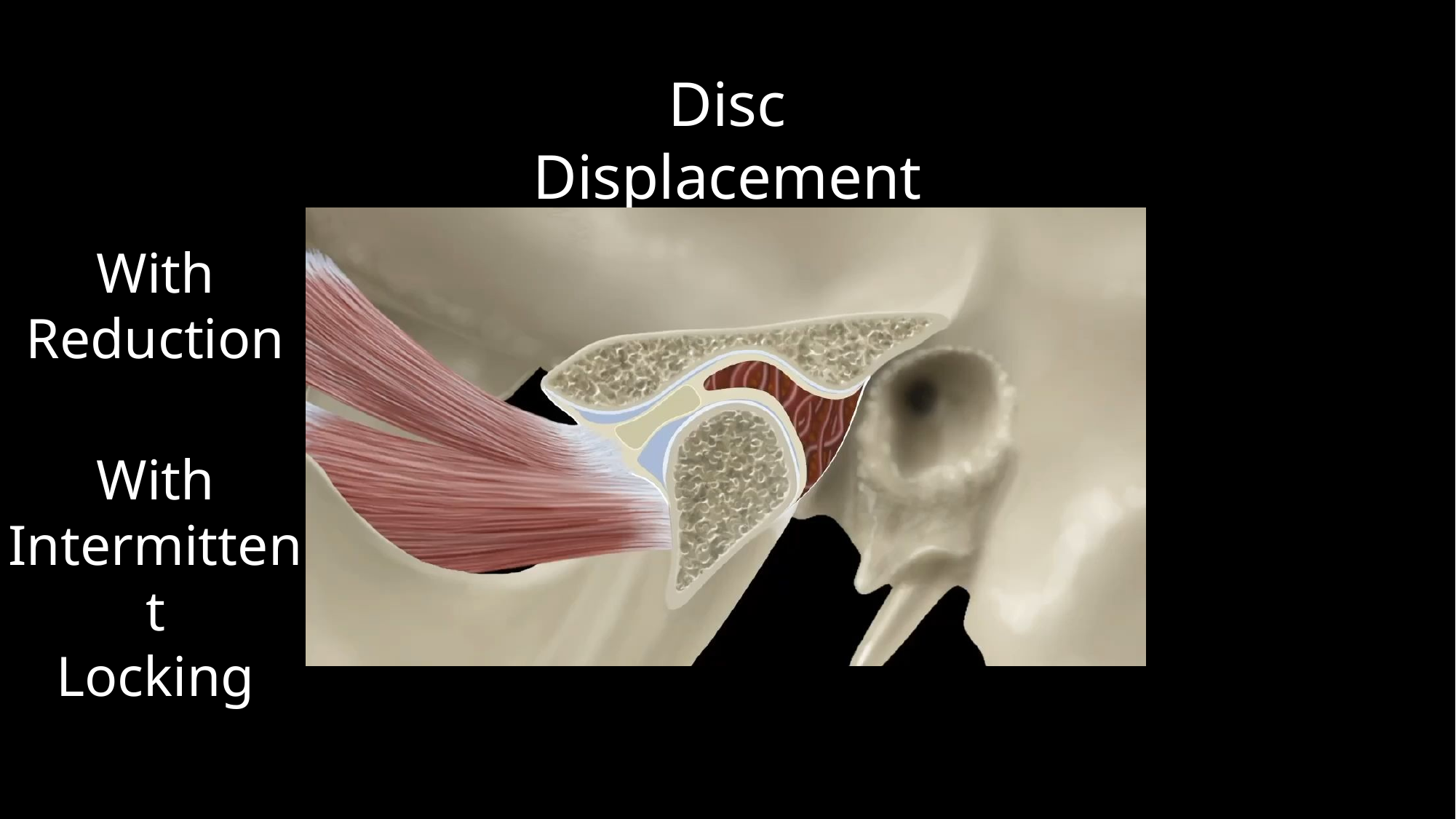

Disc Displacement
With Reduction
With
Intermittent
Locking

## Slide 19
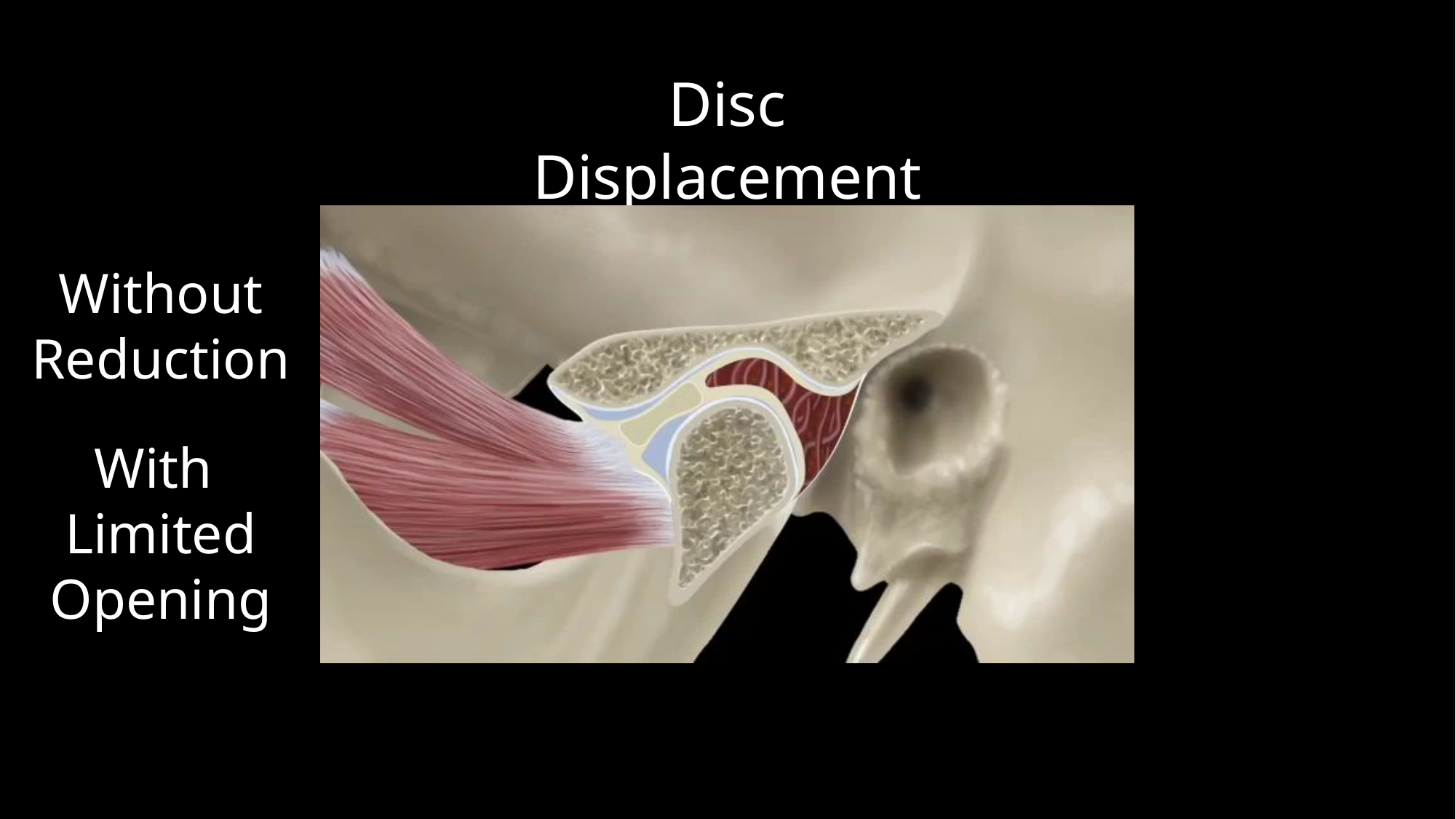

Disc Displacement
Without Reduction
With
Limited Opening

## Slide 20
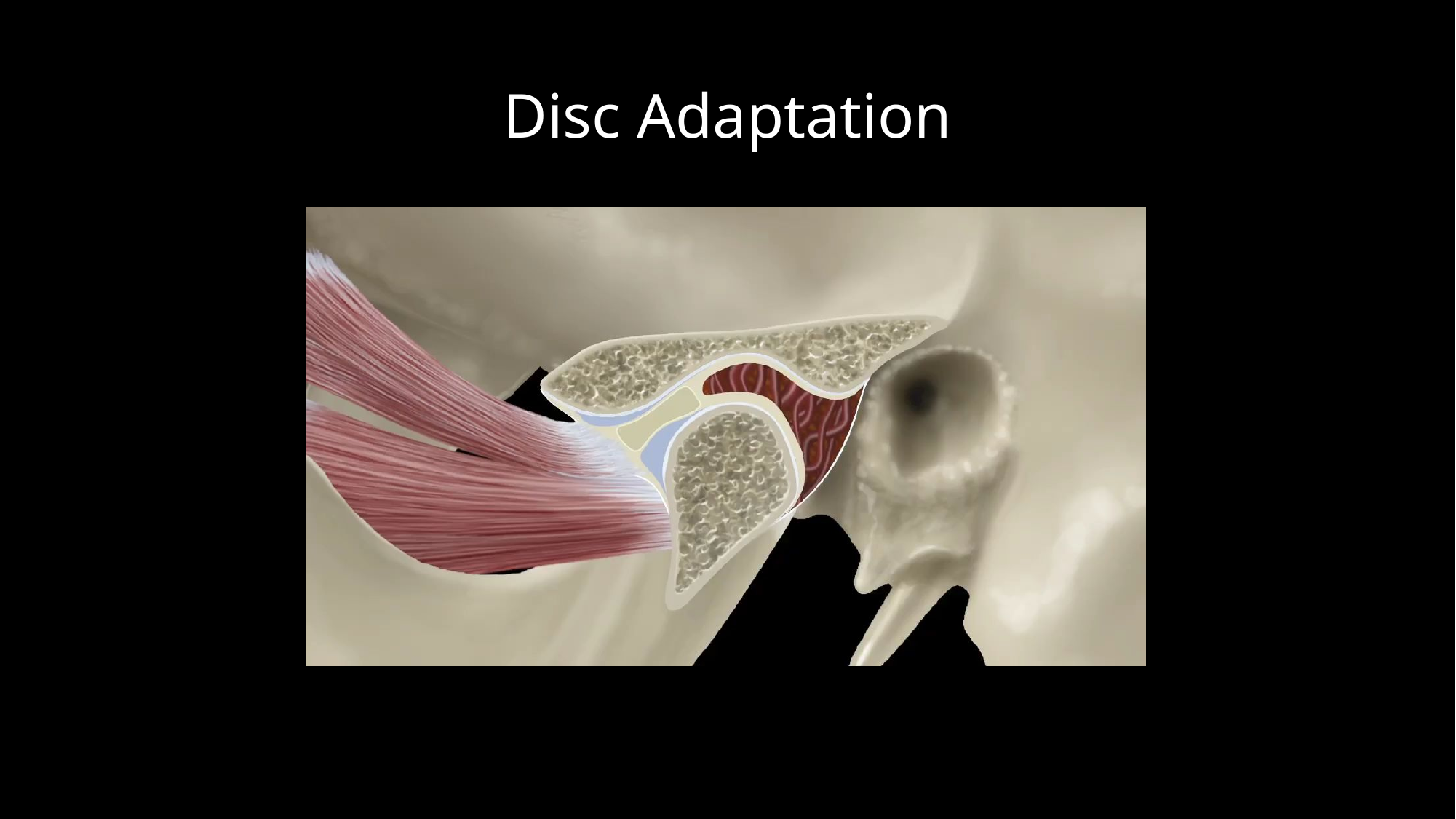

Disc Adaptation

## Slide 21
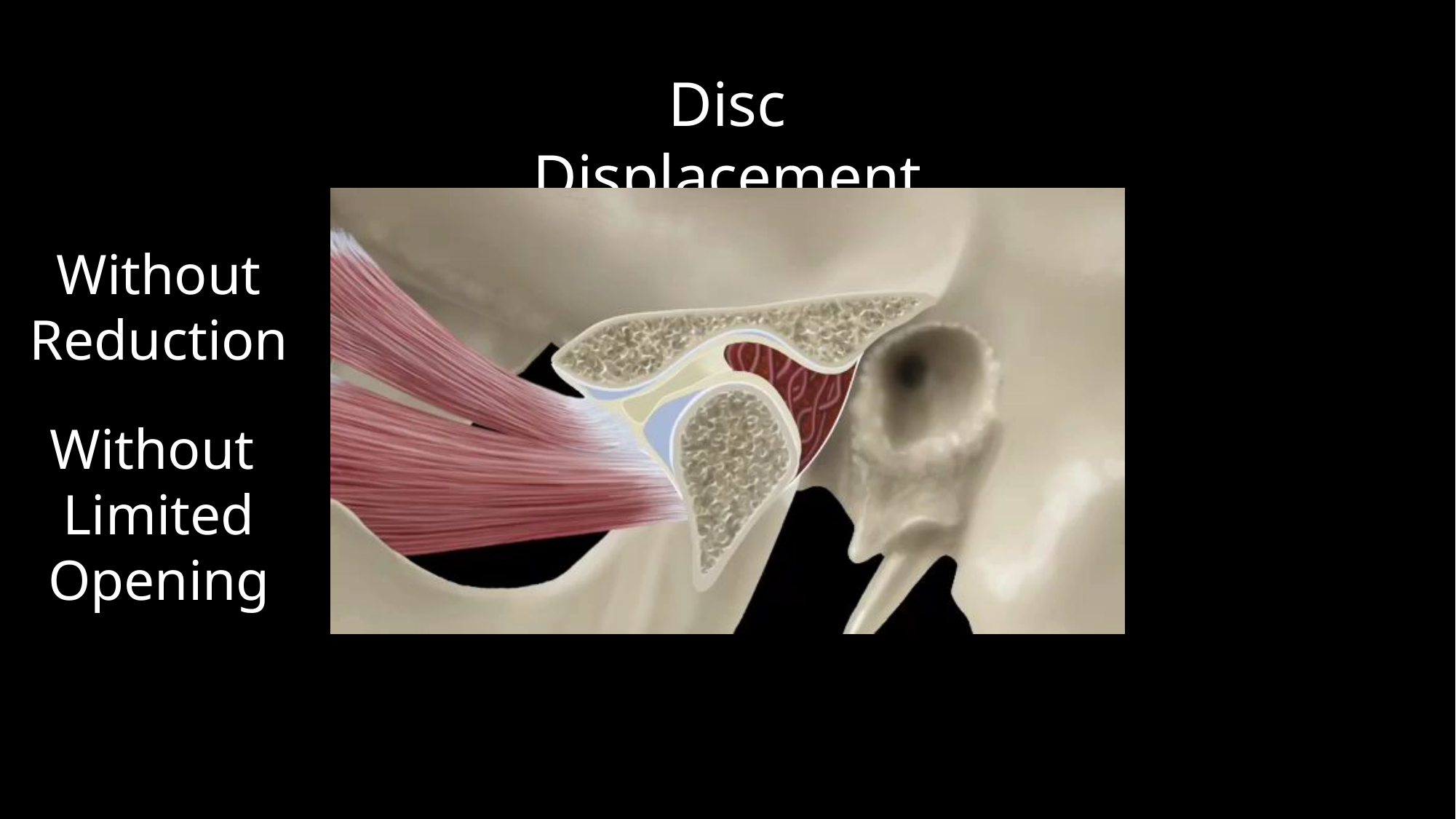

Disc Displacement
Without Reduction
Without
Limited Opening

## Slide 22
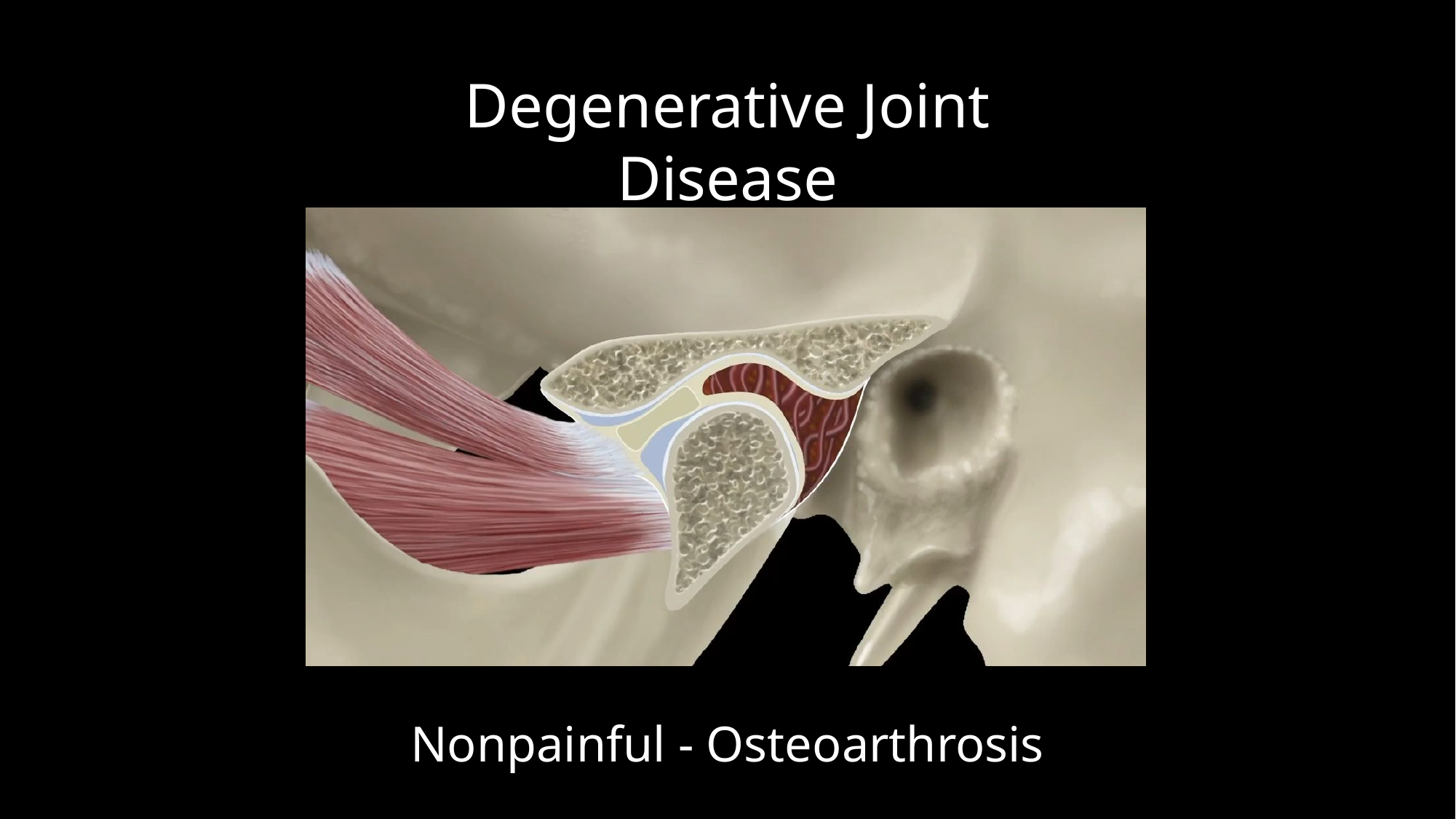

Degenerative Joint Disease
Nonpainful - Osteoarthrosis

## Slide 23
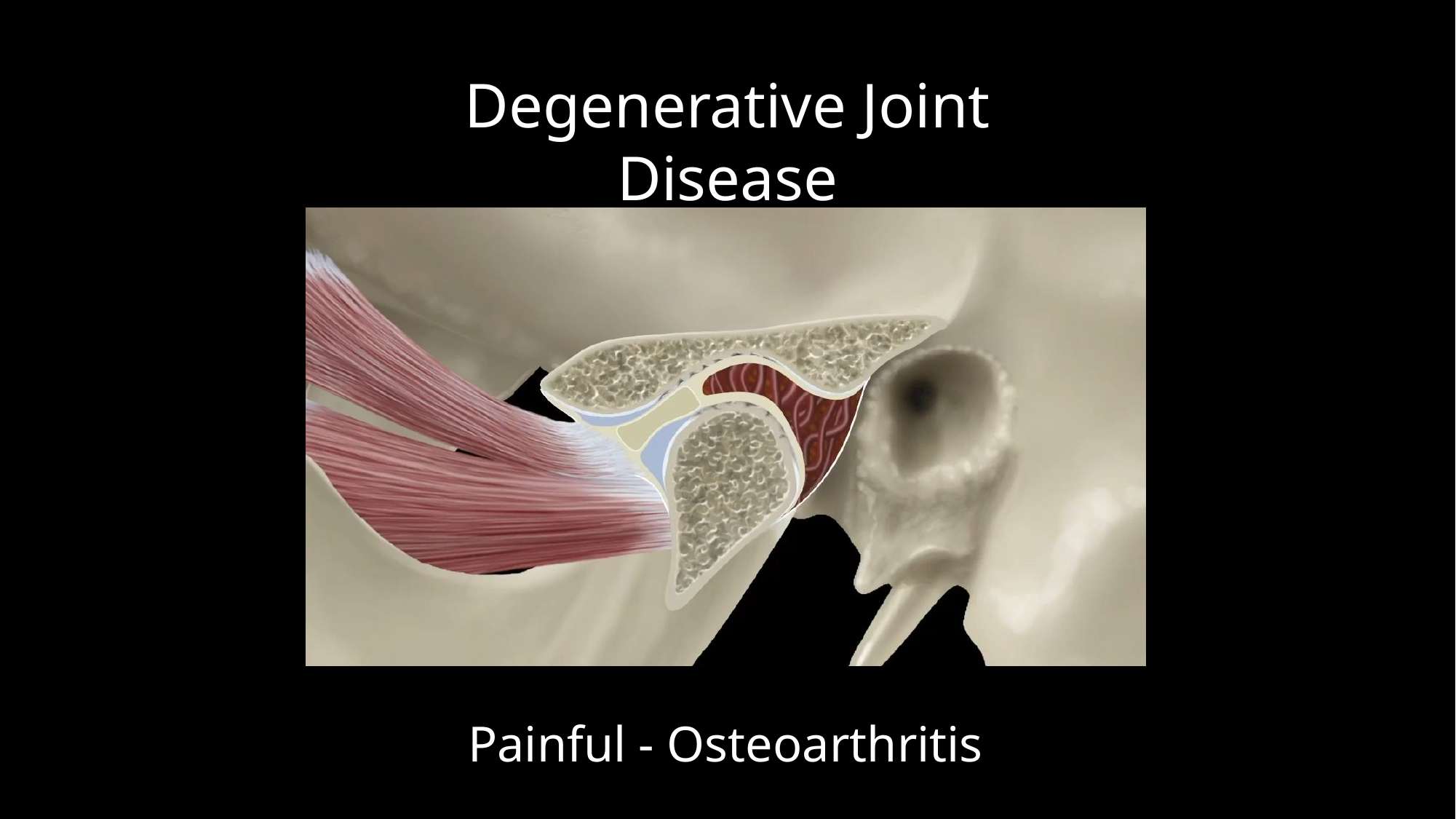

Degenerative Joint Disease
Painful - Osteoarthritis

## Slide 24
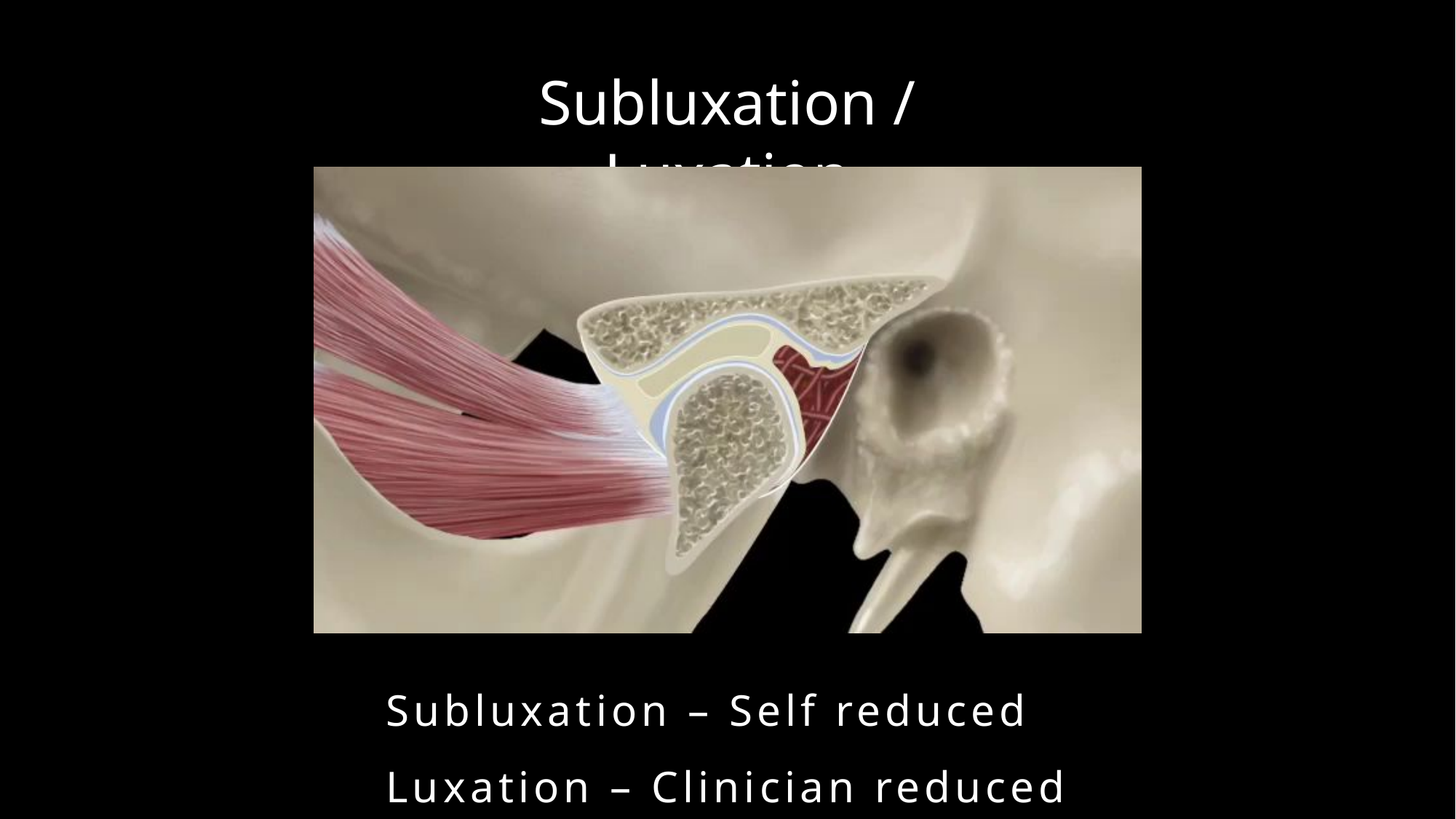

Subluxation / Luxation
Subluxation – Self reduced
Luxation – Clinician reduced

## Slide 25
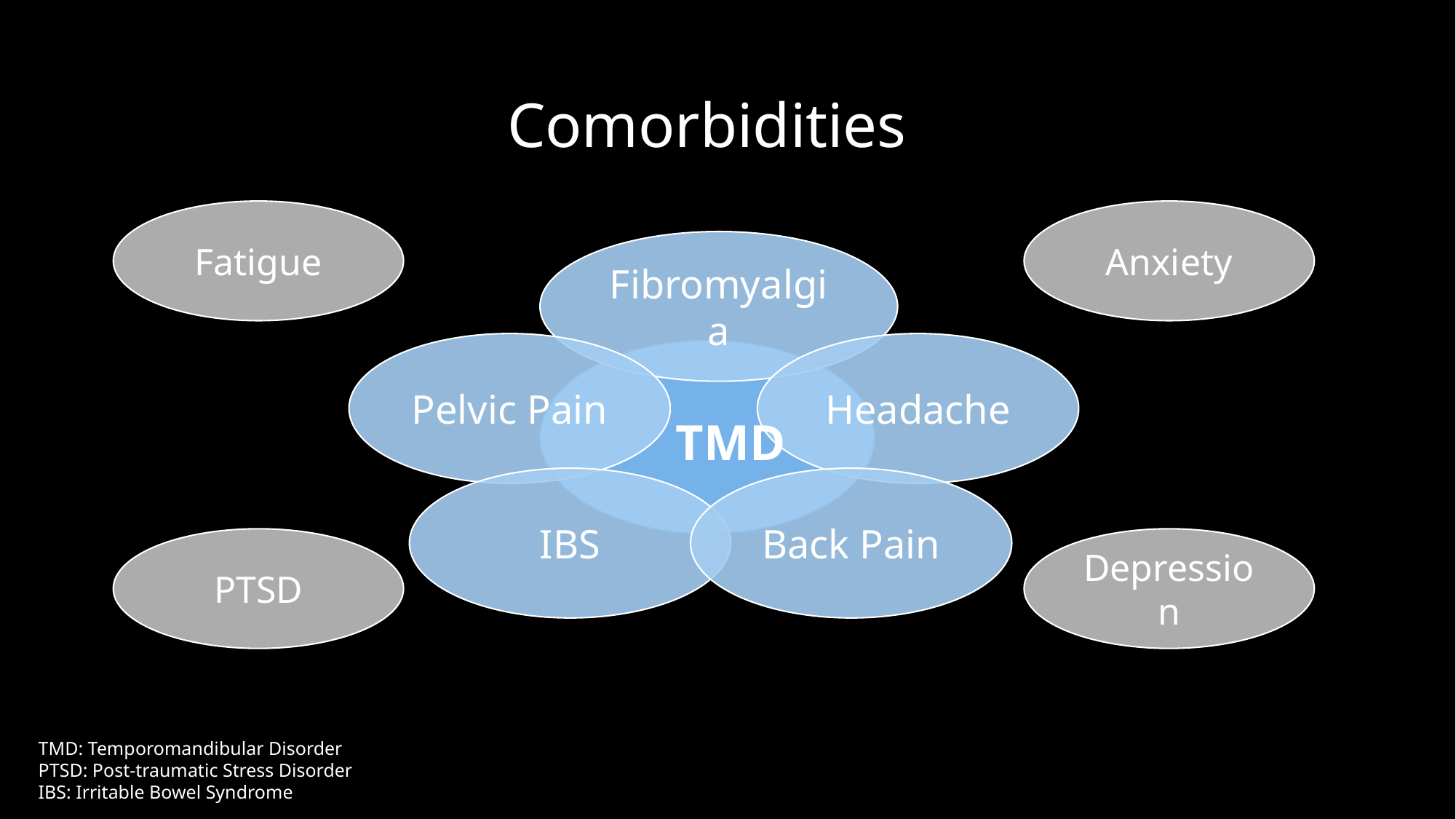

Comorbidities
Fatigue
Anxiety
Fibromyalgia
Pelvic Pain
Headache
TMD
IBS
Back Pain
PTSD
Depression
TMD: Temporomandibular Disorder
PTSD: Post-traumatic Stress Disorder
IBS: Irritable Bowel Syndrome

## Slide 26
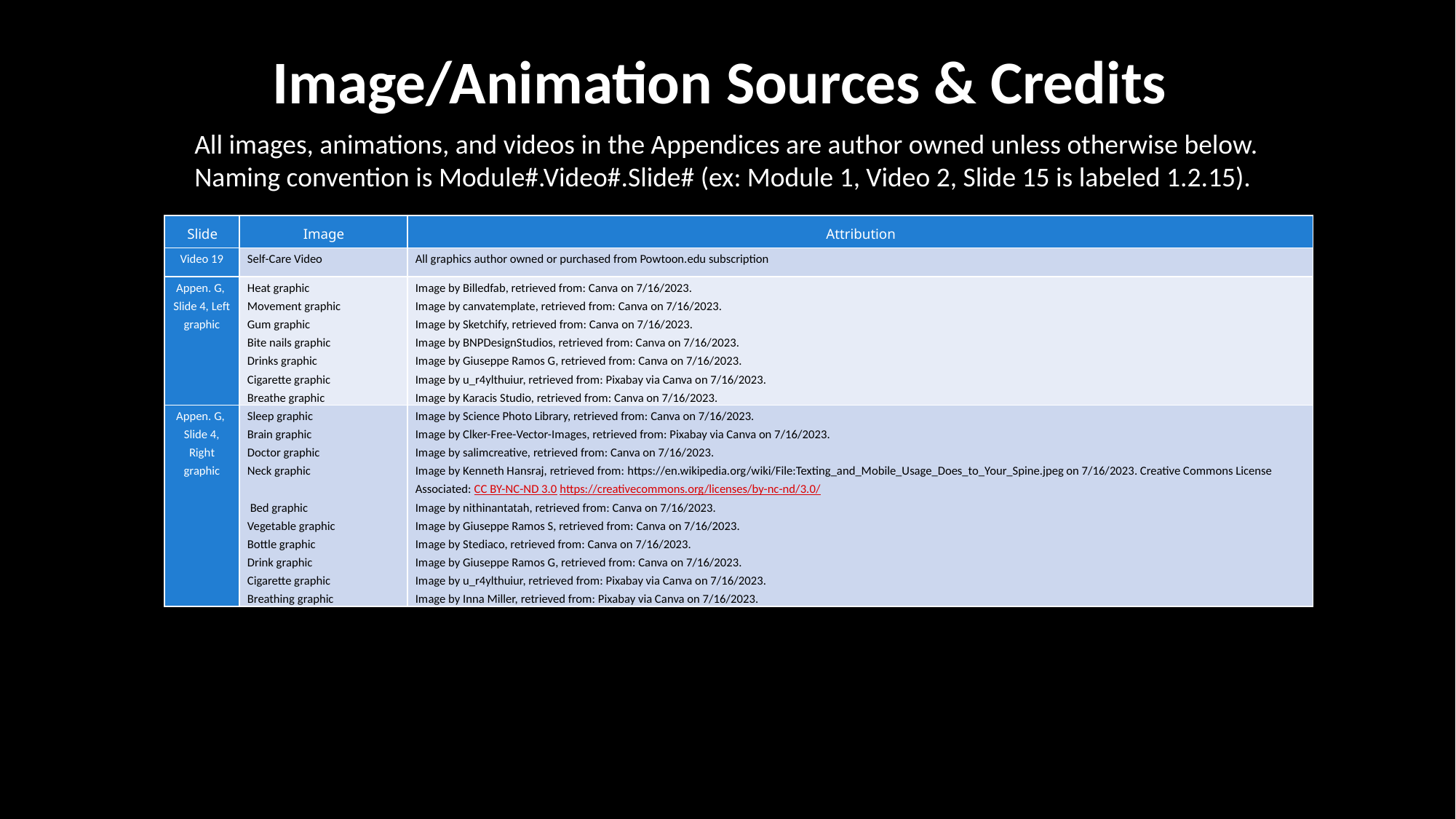

# Image/Animation Sources & Credits
All images, animations, and videos in the Appendices are author owned unless otherwise below. Naming convention is Module#.Video#.Slide# (ex: Module 1, Video 2, Slide 15 is labeled 1.2.15).
| Slide | Image | Attribution |
| --- | --- | --- |
| Video 19 | Self-Care Video | All graphics author owned or purchased from Powtoon.edu subscription |
| Appen. G, Slide 4, Left graphic | Heat graphic Movement graphic Gum graphic Bite nails graphic Drinks graphic Cigarette graphic Breathe graphic | Image by Billedfab, retrieved from: Canva on 7/16/2023. Image by canvatemplate, retrieved from: Canva on 7/16/2023. Image by Sketchify, retrieved from: Canva on 7/16/2023. Image by BNPDesignStudios, retrieved from: Canva on 7/16/2023. Image by Giuseppe Ramos G, retrieved from: Canva on 7/16/2023. Image by u\_r4ylthuiur, retrieved from: Pixabay via Canva on 7/16/2023. Image by Karacis Studio, retrieved from: Canva on 7/16/2023. |
| Appen. G, Slide 4, Right graphic | Sleep graphic Brain graphic Doctor graphic Neck graphic    Bed graphic Vegetable graphic Bottle graphic Drink graphic Cigarette graphic Breathing graphic | Image by Science Photo Library, retrieved from: Canva on 7/16/2023. Image by Clker-Free-Vector-Images, retrieved from: Pixabay via Canva on 7/16/2023. Image by salimcreative, retrieved from: Canva on 7/16/2023. Image by Kenneth Hansraj, retrieved from: https://en.wikipedia.org/wiki/File:Texting\_and\_Mobile\_Usage\_Does\_to\_Your\_Spine.jpeg on 7/16/2023. Creative Commons License Associated: CC BY-NC-ND 3.0 https://creativecommons.org/licenses/by-nc-nd/3.0/ Image by nithinantatah, retrieved from: Canva on 7/16/2023. Image by Giuseppe Ramos S, retrieved from: Canva on 7/16/2023. Image by Stediaco, retrieved from: Canva on 7/16/2023. Image by Giuseppe Ramos G, retrieved from: Canva on 7/16/2023. Image by u\_r4ylthuiur, retrieved from: Pixabay via Canva on 7/16/2023. Image by Inna Miller, retrieved from: Pixabay via Canva on 7/16/2023. |
